# Monodisperse Chemical Oligophosphorylation of Peptides via Protected Oligophosphorimidazolide Reagents

**DOI:** 10.1002/anie.202419147

**Published:** 2024-12-16

**Authors:** Kevin Qian, Björn Hanf, Christopher Cummins, Dorothea Fiedler

**Affiliations:** ^1^ Department of Chemistry Massachusetts Institute of Technology (MIT) 77 Massachusetts Ave. Cambridge MA-02139 United States of America; ^2^ Leibniz-Forschungsinstitut für Molekulare Pharmakologie (FMP) Robert-Rössle-Str. 10 13125 Berlin Germany; ^3^ Institut für Chemie Humboldt-Universität zu Berlin, Germany Brook-Taylor-Str. 2 12489 Berlin Germany

**Keywords:** Peptides and Proteins, Phosphorylation, Post-translational Modifications, Reagents, Anions

## Abstract

Protein poly‐ and oligophosphorylation are recently discovered post‐translational modifications that remain poorly characterized due to (1) the difficulty of extracting endogenously polyphosphorylated species without degradation and (2) the absence of synthetic and analytical tools to prepare and characterize poly‐ and oligophosphorylated species in biochemical contexts. Herein, we report a methodology for the selective oligophosphorylation of peptides with monodisperse phosphate chain lengths (P_n_=3–6). A library of oligophosphorimidazolide (oligoP‐imidazolide) reagents featuring benzyl and *o*‐nitrophenylethyl protecting groups was synthesized in moderate‐to‐good yields (65–93 %). These oligoP‐imidazolide reagents enabled the selective and simultaneous conjugation of multiple phosphate units to phosphoryl nucleophiles, circumventing tedious iterative processes. The generalizability of this approach is illustrated by a substrate scope study that includes several biologically relevant phosphopeptide sequences, culminating in the synthesis of >60 examples of peptide oligophosphates (P_n_=2–6). Moreover, we report the preparation of oligoP‐diimidazolides (P_n_=3–5) and discuss their application in generating unique condensed phosphate‐peptide conjugates. We also demonstrate that human phospho‐ubiquitin (pS65‐Ub) is amenable to functionalization by our reagents. Overall, we envision the methods described here will enable future studies that characterize these newly discovered but poorly understood phosphorylation modes.

## Graphical abstract

**The more the merrier**: Oligophosphorimidazolide reagents enable the concomitant conjugation of multiple phosphate units to phosphopeptides, yielding derivatives with monodisperse phosphate chain lengths (up to hexaphosphate). This simple transformation occurs chemoselectively under mild conditions. This work paves the way for future development of mass spectrometry‐based analytical methods for “oligophosphoproteomics.”

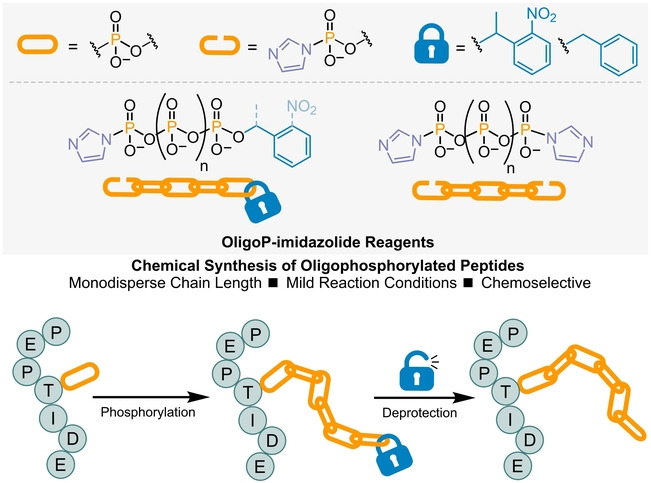

## Introduction

Post‐translational modifications (PTMs) are essential for regulating protein structure, function, localization, and activity. Many PTMs involve the addition of a small molecular fragment to a specific residue (e.g., phosphorylation, methylation, acetylation), while other PTMs entail the conjugation of larger biomolecules to the protein (e.g., glycosylation, lipidation, ubiquitination).[^1^, ^2^] Monophosphorylation is among the most abundant and extensively characterized PTMs—especially at serine, threonine, and tyrosine residues—but the phosphorylation of other side chains such as histidine and cysteine is also known.[^1^, ^3^, ^4^, ^5^] While the range of residues targeted by this PTM has been thoroughly explored, investigations into the modification of the phosphoryl group itself (i.e., via the formation of phosphoanhydride bonds) have only recently begun.

**Figure 1:**
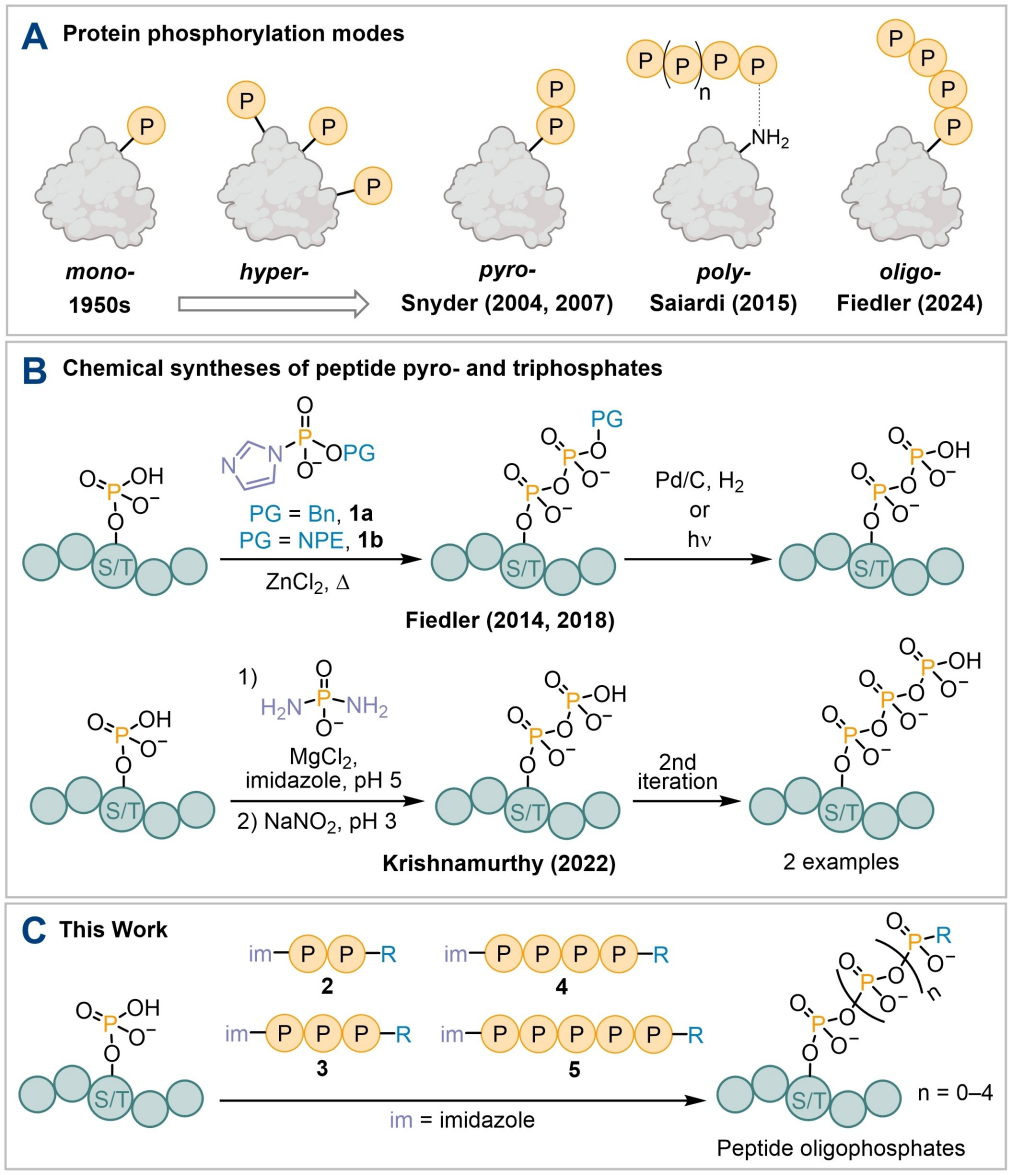
A) Discovery of different phosphorylation modes; P=phosphoryl group. (B) Reported chemical methods for preparing peptide pyro‐ and triphosphates. (C) Summary of present work with new oligophosphorylation reagents; R=OBn, O‐NPE, im.

Two landmark studies by Snyder and co‐workers demonstrated that the messenger molecule 5‐diphosphoinositol‐1,2,3,4,6‐pentakisphosphate (5PP‐InsP_5_) can transfer its high‐energy *β*‐phosphoryl group to various eukaryotic proteins.^[6]^ Interestingly, this phosphoryl transfer occurs selectively on pre‐phosphorylated side chains (predominantly phosphoserine residues), resulting in the formation of a pyrophosphate group (Figure 1A).^[7]^ Nearly a decade later, Saiardi and co‐workers discovered that two proteins in yeast, NSR1 (Nuclear localization sequence‐binding protein) and its binding partner TOP1 (DNA topoisomerase 1), are seemingly covalently modified by inorganic polyphosphate (polyP) at lysine residues in polyacidic serine‐and‐lysine‐rich (PASK) regions (Figure 1A).^[8]^ Since then, several additional proteins susceptible to polyphosphorylation have been identified, primarily in yeast and humans.[^9^, ^10^, ^11^] Most recently, it was discovered that nucleoside diphosphate kinase A (NME1), an enzyme known to be a metastasis suppressor,^[12]^ can undergo oligophosphorylation on a phosphothreonine residue in an auto‐catalytic fashion (Figure 1A).^[13]^

However, the extent of the proteome that is amenable to pyro‐, oligo‐, and polyphosphorylation, along with many questions regarding how these modifications are installed/removed and how they affect protein structure and function remain to be elucidated. The study of these PTMs is further complicated by the modifications’ high negative charge and labile nature, which renders the extraction and analysis of intact endogenous samples a considerable challenge.[^14^, ^15^, ^16^]

Mass spectrometry (MS) has emerged as a central analytical technique for phospho‐ and pyrophosphoproteomics.[^14^, ^17^] The ability to synthesize well‐defined peptide standards containing these modifications was vital for developing appropriate enrichment and high‐throughput MS‐based workflows.[^14^, ^16^, ^17^] The synthesis of pyrophosphopeptides was initially accomplished by utilizing benzyl‐protected phosphorimidazolide (P‐imidazolide) reagent **1 a** or photocaged derivative **1 b** to selectively convert phosphopeptide substrates to their pyrophosphorylated counterparts (Figure 1B).[^18^, ^19^] Alternatively, pyrophosphorylated peptides could be synthesized in an aqueous one‐pot reaction utilizing diamidophosphate (Figure 1B).^[20]^ The latter report also provided two examples of triphosphopeptides by one‐pot iterative phosphorylations.

There are no antibodies available for the detection and characterization of protein oligo‐ and polyphosphorylation, and a tailored high‐throughput MS‐based workflow has yet to be developed. This absence of analytical methods hinders the community's ability to probe these unusual modifications.^[15]^ In fact, whether polyphosphorylation at lysine is a covalent modification has been subject to recent debate,[^21^, ^22^] and settling these open questions will require more sophisticated analytical tools. Although several methods exist for the synthesis of nucleoside oligophosphates (nucleoside oligoPs),[^23^, ^24^] there are currently no reported strategies for the conjugation of longer condensed phosphates with peptides/proteins, let alone with a defined phosphate chain length.^[25]^ To that end, we now report the development of a set of P(V) phosphorylation reagents (**2**–**5**) that selectively transform phosphopeptides into their corresponding oligophosphorylated derivatives (Figure 1C).

## Results and Discussion

**Scheme 1:**
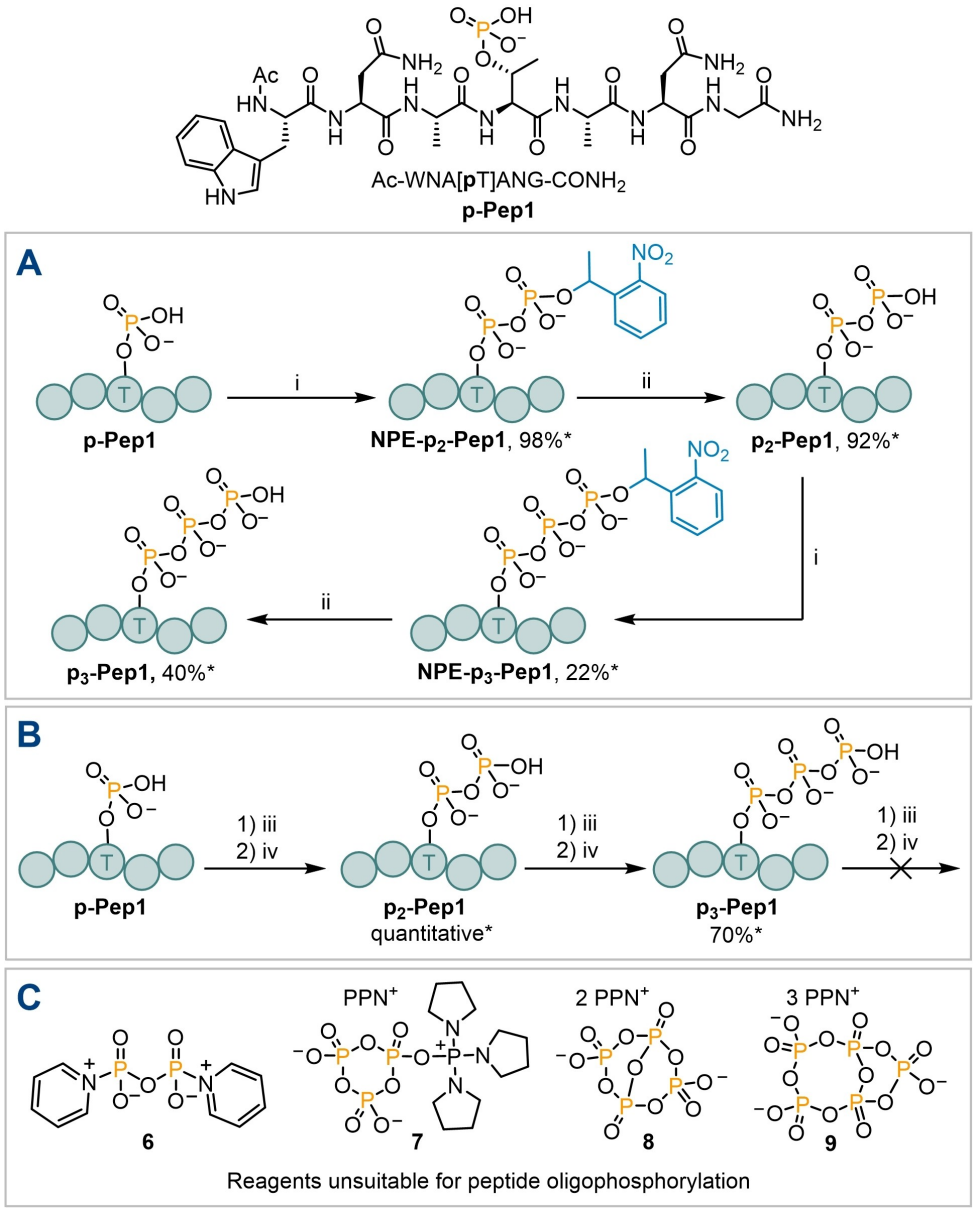
Initial attempts to synthesize oligophosphorylated peptides from model substrate **p‐Pep1** with (A) reagent **1 b**, (B) diamidophosphate, and (C) reagents **6**–**9**. Conditions: i) 3 equiv. **1 b**, 8 equiv. ZnCl_2_, 9 : 1 DMA/H_2_O, 1.5 h, 45 °C. ii) 365 nm LED, 0.1 M aq. NH_4_HCO_3_. iii) 8 equiv. diamidophosphate, 2 equiv. MgCl_2_, 2 equiv. imidazole, pH 5.5, 20 h, −20 °C. iv) 8 equiv. NaNO_2_, pH 3, 8 h, −20 °C. (*RP‐HPLC yield).

**Iterative phosphorylation sequence proved inefficient**: We initially attempted to prepare peptide oligoPs via iterative phosphorylations of a model phosphopeptide substrate **p‐Pep1** utilizing the aforementioned reagents published by the groups of Fiedler and Krishnamurthy.[^18^, ^19^, ^20^] Following previously optimized conditions,^[19]^ **p‐Pep1** was treated with 3 equiv. of reagent **1 b** in the presence of ZnCl_2_, affording the protected peptide pyrophosphate with 98 % conversion as determined by reverse‐phase high‐performance liquid chromatography (RP‐HPLC) (Scheme 1A). Deprotection of the photolabile *o*‐nitrophenylethyl (NPE) group by ultra‐violet (UV) irradiation at λ=365 nm yielded pyrophosphopeptide **p_2_‐Pep1**. Unfortunately, much‐diminished yields (22 %) were observed for the subsequent phosphorylation reaction of **p_2_‐Pep1** with **1 b**. This synthesis also proved to be exceptionally time‐consuming, requiring the RP‐HPLC purification of intermediates after each phosphorylation step. Similar results were obtained utilizing diamidophosphate in a one‐pot reaction (Scheme 1B).^[20]^ While the generation of **p_2_‐Pep1** proceeded quantitatively, the subsequent iteration led to only partial conversion to the triphosphate **p_3_‐Pep1**. Attempts at further phosphorylation led to the generation of unidentified decomposition products and no observed formation of the targeted tetraphosphorylated peptide. The latter method also suffers from slow kinetics, with reactions carried out over the course of several days. These issues render the synthesis of peptide oligoPs with chain lengths >3 an infeasible task for iterative phosphorylation methodologies.

A general and efficient synthetic method for oligophosphorylated peptides would require the introduction of multiple phosphate units in a single synthetic step. The Cummins group recently reported several reagents (**6**–**9**) effective for the synthesis of oligoPs of uniform chain length, including nucleotide derivatives.[^26^, ^27^, ^28^, ^29^] Unfortunately, no compatible conditions were found utilizing these reagents on peptide substrates (Scheme 1C); these reactions failed due to a combination of solvent compatibility issues, poor tolerance for water, and insufficient chemoselectivity towards the various nucleophilic side chains.

**Design and Synthesis of OligoP‐imidazolides**: While compounds **6**–**9** proved to be ineffective for phosphorylating peptidic substrates, we speculated that their derivatives might still be potent reagents for peptide oligophosphorylation. We revisited the chemistry of P‐imidazolides, a notable class of P(V) electrophiles that have been investigated for their role in the prebiotic synthesis of oligonucleotides.[^30^, ^31^, ^32^, ^33^, ^34^, ^35^] Developed over the past several decades, P‐imidazolides have emerged as a powerful synthetic tool for forming phosphoanhydride bonds in the preparation of nucleotide derivatives.[^36^, ^37^] The applications of these reagents have recently been broadened beyond the domain of nucleotides to also encompass peptides and proteins.[^18^, ^19^]

We therefore aimed to synthesize a range of oligophosphorimidazolides (oligoP‐imidazolides) featuring benzyl and NPE protecting groups. By leveraging the privileged reactivity exhibited by this class of P(V) electrophiles, these reagents were envisioned to facilitate the conversion of phosphopeptide substrates into peptide oligoPs through a two‐step conjugation/deprotection process.

**Scheme 2:**
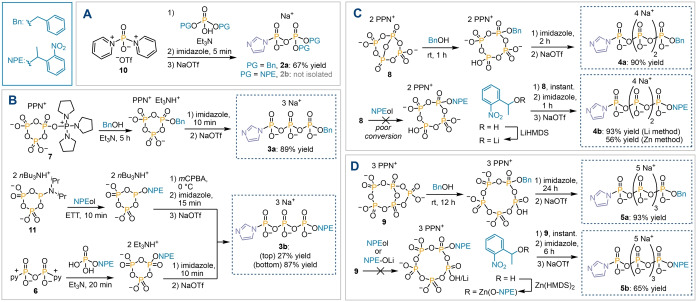
Synthesis of benzyl‐ and *o*‐nitrophenylethyl substituted oligoP‐imidazolide (A) diphosphorylation, (B) triphosphorylation, (C) tetraphosphorylation, and (D) pentaphosphorylation reagents. See Supporting Information for details regarding synthesis and characterization.

P‐imidazolides are typically prepared from phosphate esters by treatment with PPh_3_, 2,2’‐dithiodipyridine, and imidazole or by reaction with carbonyl diimidazole.[^38^, ^39^, ^40^, ^41^] However, the scarcity of commercially available functionalized condensed phosphates (beyond nucleotides) and the propensity for longer‐chain condensed phosphates to undergo rearrangement reactions rendered the synthesis of the proposed oligoP‐imidazolides less feasible by these traditional routes. Inspired by recent work from Jessen demonstrating the linearization of azidothymidine‐substituted trimetaphosphate (triMP) with imidazole,^[42]^ we postulated that the phosphorylation reagents **6**–**9**, which consist of cyclic metaphosphates (MPs) or produce functionalized MPs as intermediates, would serve as convenient precursors for oligoP‐imidazolides. The general synthetic strategies employed to produce the set of oligoP‐imidazolide reagents are summarized in Scheme 2.

The preparation of many pyrophosphates involves the coupling of two monophosphoryl precursors.[^43^, ^44^] We resorted to a similar strategy to prepare diphosphorylation reagents **2 a** and **2 b** (Scheme 2A). Weigand and co‐workers recently published a direct synthesis of precursor **10** from phosphoric acid and triflic anhydride.^[45]^ Sequential additions of the corresponding phosphate diesters and imidazole to **10** afforded compounds **2 a** and **2 b**. Reagent **2 a** was obtained in 67 % yield and isolated in high purity as the sodium salt following counter‐ion exchange with sodium triflate (NaOTf). Although **2 b** could be cleanly generated in situ, the NPE groups confer higher lipophilicity than benzyl groups, preventing the convenient isolation of **2 b** by precipitation as the sodium salt. We also observed the gradual decomposition of this compound upon concentration. Due to the lack of long‐term stability, the reactivity of **2 b** was not explored in the present study.

The phosphorylation of benzyl alcohol (BnOH) with reagent **7** produces benzyl triMP,^[27]^ which was ring‐opened with excess imidazole to yield **3 a** (Scheme 2B). Unfortunately, the NPE‐derivative **3 b** was not accessible via the same method due to the increased steric bulk of *o*‐nitrophenylethanol (NPEol) compared to BnOH. Instead, **3 b** was synthesized by two alternative routes: (1) Utilizing the more reactive cyclic pyrophosphoryl P‐amidite **11** previously published by Jessen and co‐workers,[^42^, ^46^] the reaction with NPEol formed a mixed P(III)–P(V) cyclic intermediate. Oxidation to the triMP with *meta*‐chloroperoxybenzoic acid (*m*CPBA) and ring‐opening with imidazole afforded **3 b** in moderate 27 % yield. (2) The same triMP intermediate could also be generated quantitatively by treating NPE‐phosphate monoester with diphosphorylation reagent **6**,^[26]^ resulting in the isolation of pure **3 b** in 87 % yield after linearization.

As previously published,^[28]^ compound **8** is a competent tetraphosphorylation reagent that reacts with BnOH to generate benzyl tetraMP (Scheme 2C). The linearization of the larger eight‐membered tetraMP ring by imidazole (to afford pure **4 a** in 90 % yield) was noticeably slower than that of triMP derivatives due to decreased ring strain. Again, the increased steric demand of NPEol complicated the synthesis of the NPE‐derivative **4 b**. Even at elevated temperatures for multiple days, the conversion to NPE‐tetraMP from **8** was low and accompanied by significant byproduct formation. To proceed, it was necessary to convert the substrate alcohol to the more nucleophilic lithium benzyl‐oxo salt (NPE‐OLi). The lithium salt reacted instantaneously with **8** to give a binary mixture of isomeric intermediates (SI section 2.3.2), which were linearized to product **4 b**, isolated in 93 % yield.

Compound **9** is a recently published—and first—example of a pentaphosphorylation reagent.^[29]^ Product **5 a** was synthesized from **9** in a similar fashion as **3 a** and **4 a** (Scheme 2D). Consistent with the trend, the kinetics of ring‐opening of benzyl pentaMP by imidazole was considerably slower than in the smaller‐ringed systems, requiring a 24‐hour reaction. Once again, the synthesis of the bulkier NPE‐substituted **5 b** was slightly more complicated than that of the benzyl derivative. There was no observed reaction between **9** and NPEol, and employing NPE‐OLi resulted in a complex mixture of products. We eventually discovered that the milder zinc derivative Zn(O‐NPE)_2_ was a suitable substrate for **9** to give the desired product **5 b**. The same Zn(O‐NPE)_2_ salt was also used to prepare **4 b** from **8**, although with slightly diminished yields compared to the lithium method.

In summary, we obtained a series of oligoP‐imidazolide reagents (P_n_=2–5) featuring terminal benzyl and NPE protecting groups. All compounds were afforded in moderate‐to‐good yields and did not require chromatographic purification.

**Reaction Optimization with Model Phosphopeptide**: To validate the utility of these newly generated oligoP‐imidazolide reagents, conditions were screened for the optimization of the reaction between a model phosphopeptide **p‐Pep1** and reagent **4 a** (Table 1). These reactants were chosen due to their relative ease of preparation and scale‐up, and because **p‐Pep1** features a tryptophan residue enabling convenient detection and quantification of reactants and products by UV/Vis. No conversion to the product was observed when only **p‐Pep1** and **4 a** were combined (Table 1, entry 1), consistent with previous observations from Sawai that divalent metal ions are necessary to activate P‐imidazolides.[^31^, ^47^] For this particular reaction, both ZnCl_2_ and MgCl_2_ proved to be effective Lewis‐acid promoters, although ZnCl_2_ gave marginally better yields. Reaction rates were found to be highly dependent on solvent; polar organic solvents such as dimethylacetamide (DMA), dimethylformamide (DMF), and dimethyl sulfoxide (DMSO) were all tolerated (Table 1, entries 5–7). A 9 : 1 binary mixture of DMA and water gave the overall highest yields (Table 1, entry 9), but conversion to the product was vastly diminished when the percent composition of water was increased (Table 1, entry 10). Under completely aqueous conditions, the reaction conversion amounted to only 4 % (Table 1, entry 11), presumably impeded by the competing hydrolysis of reagent **4 a** and the formation of a solvation shell around the peptide phosphoryl group and the metal ion promoter.[^48^, ^49^, ^50^] The kinetics of oligoP‐imidazolide hydrolysis are discussed in the Supporting Information (SI section 9). Yields for reactions conducted in water improved significantly—up to a moderate yield of 45 %—with increases in total reaction concentration and relative amounts of **4 a** and ZnCl_2_ (Table 1, entries 12–13).

**Table 1:** Optimization of conditions for oligophosphorylation of pThr model peptide **p‐Pep1**.

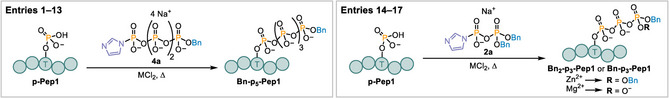						
entry	reagent (equiv.)	M^2+^ (equiv.)	solvent	temp. (°C)	time (h)	% conversion^c^
1^a^	**4 a** (3)	–	DMA	rt (~22)	3	0
2^a^	**4 a** (3)	Zn^2+^ (10)	DMA	rt	5	11
3^a^	**4 a** (10)	Zn^2+^ (10)	DMA	rt	5	14
4^a^	**4 a** (10)	Zn^2+^ (10)	DMA	45	6	49
5^a^	**4 a** (10)	Zn^2+^ (50)	DMA	45	1.5, 6	50, 71
6^a^	**4 a** (10)	Zn^2+^ (50)	DMF	45	1.5	85
7^a^	**4 a** (10)	Zn^2+^ (50)	DMSO	45	1.5	21
8^a^	**4 a** (10)	Mg^2+^ (50)	DMA	45	1.5	45
9^a^	**4 a** (10)	Zn^2+^ (50)	DMA/H_2_O (9 : 1)	45	1.5	92
10^a^	**4 a** (10)	Zn^2+^ (50)	DMA/H_2_O (1 : 1)	45	1	23
11^a^	**4 a** (10)	Zn^2+^ (50)	H_2_O	45	1	4
12^b^	**4 a** (10)	Zn^2+^ (50)	H_2_O	45	1.5	22
13^b^	**4 a** (30)	Zn^2+^ (150)	H_2_O	45	1.5	45
14^a^	**2 a** (10)	Zn^2+^ (50)	DMA	45	1	52 (Bn_2_)
15^a^	**2 a** (10)	Zn^2+^ (50)	DMF	45	1	51 (Bn_2_)
16^a^	**2 a** (10)	Mg^2+^ (50)	DMA	45	1	72 (Bn)
17^a^	**2 a** (10)	Mg^2+^ (50)	DMF	45	1	98 (Bn)

^a^Standard conditions: 1.2 mM substrate. ^b^6 mM of substrate. ^c^Conversion determined by analytical RP‐HPLC.

Reaction conditions were also optimized for the phosphorylation of **p‐Pep1** with **2 a** (Table 1, entries 14–17). Reagent **2 a** features a terminal phosphate diester and exhibits slightly different reactivity. The reactions proceeded under similar conditions as those with **4 a**, forming benzylated peptide triphosphates. Curiously, using ZnCl_2_ as an additive provided the expected dibenzyl product **Bn_2_‐p_3_‐Pep1** as the major species after one hour, while MgCl_2_ inexplicably led to the loss of a single benzyl group to produce **Bn‐p_3_‐Pep1** with 98 % conversion. Currently, we are unable to rationalize this discrepancy between these Lewis acids, but given the ultimate objective is the deprotection of the benzyl groups, this outcome with Mg^2+^ was unexpected but fortuitous.

**Figure 2:**
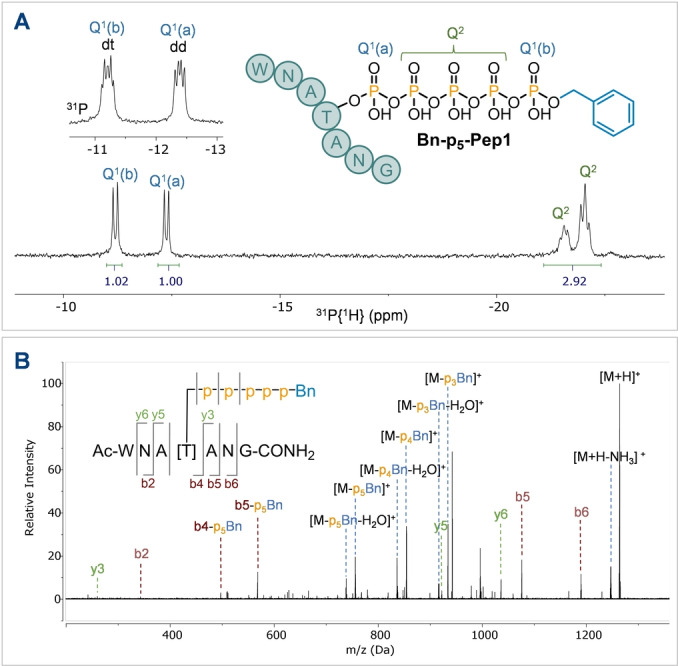
(A) ^31^P{^1^H} and ^31^P NMR spectra and (B) CID MS/MS of isolated benzyl‐pentaphosphopeptide **Bn‐p_5_‐Pep1**.

To verify the formation of a new phosphoanhydride bond, **Bn‐p_5_‐Pep1** was synthesized and isolated on a preparative scale and characterized by ^31^P nuclear magnetic resonance (NMR) spectroscopy (Figure 2A). The 1D NMR spectrum of isolated **Bn‐p_5_‐Pep1** unambiguously corresponds to a disubstituted linear pentaphosphate. Additional ^1^H NMR and 2D ^31^P‐^31^P COSY spectra corroborate this assignment (SI section 8). Similarly, full ^31^P NMR characterization of **NPE‐p_4_‐Pep2** illustrates the formation of the linear tetraphosphate (SI section 5.1).

Tandem MS/MS has been employed previously to characterize the pyrophosphorylation of peptides and proteins.[^14^, ^20^] We likewise utilized this analytical technique to verify both the formation of a pentaphosphate chain and the site‐selectivity of the modification (Figure 2B). **Bn‐p_5_‐Pep1** was characterized by positive‐mode collision‐induced dissociation (CID) MS/MS. Various daughter ions resulting from the fragmentation along both the peptide and pentaphosphate chains were detected; the observed b‐ions and y‐ions confirmed the peptide sequence, demonstrating that the oligoP chain is attached to threonine.

**Figure 3:**
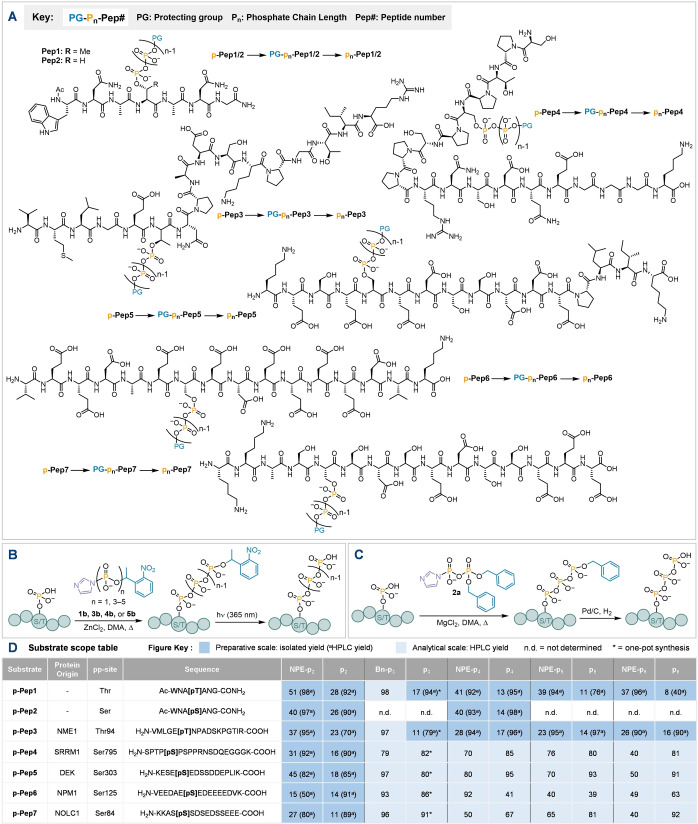
(A) Summary Scheme of oligoP‐peptides included in substrate scope. (B) Peptide oligophosphorylation with photocaged reagents. (C) Synthesis of peptide triphosphates with benzyl‐protected **2 a**. (D) Substrate scope table. *Yields for peptide triphosphates reported over two steps carried out in a one‐pot synthesis.

**Functionally diverse peptides can undergo chemical oligophosphorylation**: Having established a general protocol for the oligophosphorylation of the model phosphopeptide **p‐Pep1**, we next sought to demonstrate the utility of the new oligophosphorylation reagents by applying them to a range of phosphopeptide substrates (Figure 3A). Included in the substrate scope are two model peptides, **p‐Pep1** and **p‐Pep2**, for illustrating that the oligoP‐imidazolide reagents are compatible with both phosphoserine and phosphothreonine‐containing sequences. The other peptide sequences were chosen because they have been characterized to carry a pyrophosphate group at the indicated site,^[14]^ and many of these sites localize to PASK domains, which in turn have been linked to lysine modification by inorganic polyP in yeast cells.[^8^, ^51^] **Pep3** is a tryptic peptide sequence of nucleoside diphosphate kinase A (NME1). In a recent study, Celik et al. disclosed the non‐enzymatic auto‐oligophosphorylation of this enzyme at Thr94, and MS/MS data indicated that the extent of oligophosphorylation reaches a chain length of up to a hexaphosphate in vitro.^[13]^ Derived from serine/arginine repetitive matrix protein 1 (SRRM1), which is involved in pre‐mRNA processing, **Pep4** is a tryptic sequence previously found to be pyrophosphorylated at Ser795.^[52]^ **Pep5** is another tryptic peptide sequence from the human DEK protein, and a recent study revealed that its cytoplasmic localization was affected by supposed polyphosphorylation.^[53]^ **Pep6** and **Pep7** are non‐tryptic sequences derived from nucleophosmin (NPM1) and nucleolar and coiled‐body phosphoprotein 1 (NOLC1), respectively, and both were previously found to be pyrophosphorylated at the indicated position.^[14]^

The phosphopeptides (**p‐Peps**) were obtained by SPPS, with sequences ranging in length from six to nineteen amino acids (SI section 3). To establish the feasibility of our approach, we first pursued the synthesis of the corresponding pyrophosphopeptides using NPE‐P‐imidazolide **1 b** following reported conditions (SI section 4).^[18]^ Reagent **1 b** selectively transformed the phosphopeptides to their corresponding NPE‐substituted pyrophosphorylated intermediates (**NPE‐p_2_‐Peps**, Figure 3D). Norrish type II photodecomposition induced by irradiation at 365 nm yielded the unprotected pyrophosphopeptides (**p_2_‐Peps**). Unsurprisingly, **Peps3**–**7** were more challenging sequences than the model peptides **Pep1** and **Pep2**—especially in regard to their HPLC purification—but the desired pyrophosphopeptides were all successfully isolated and purified in good yields.

We subsequently investigated the triphosphorylation reaction of the tryptic NME1 peptide (**p‐Pep3**) with reagent **3 b** (SI section 5.1); the results of this screening were in good agreement with the optimization study performed for the oligophosphorylation of model peptide **p‐Pep1** (Table 1). Compared to the analogous phosphorylation reaction with **1 b**, a greater excess of **3 b** and ZnCl_2_ was necessary to achieve efficient conversion of **p‐Pep3** to the tetraphosphorylated derivative (**NPE‐p_4_‐Pep3**). A larger reagent‐to‐zinc ratio (1 : 8) helped to further increase the solubility of **3 b** in pure DMA, and elevating the reaction temperature to 55 °C produced higher yields.

This optimized set of conditions was applied to the remaining combinations of phosphopeptide substrates and phosphorylation reagents. Over 60 examples of peptides modified with di‐, tri‐, tetra, penta‐ or hexaphosphate groups were synthesized (Figure 3D). These reactions proceeded with moderate‐to‐good conversions, ranging from 40–95 % (SI sections 4–6). **Pep7**, a highly functionalized peptide sequence, proved to be the most challenging substrate due to its poor solubility in DMA and possibly increased charge repulsion caused by the high density of acidic side chains. Furthermore, reactions with reagent **5 b** afforded the lowest conversions. This can likely be attributed to the diminished solubility of **5 b** compared to its shorter homologs; **5 b** contains the most phosphoryl groups and consequently the highest degree of “anionicity.” This trend extends to phosphorylation reactions with **4 b**, which gave lower yields compared to those of **3 b**. Further optimization with the extended oligoP‐imidazolides will be performed in future studies.

Photodeprotection of the NPE groups was achieved by irradiation with a UV‐LED within 5–10 min, independently of the phosphate chain length and with no significant side product formation for peptide sequences **Peps3**–**7** (Figure 3B). Interestingly, during photodeprotection of the shorter model peptides **NPE‐p_5_‐Pep1** and **NPE‐p_6_‐Pep1**, partial degradation of the phosphate chain was observed, resulting in the formation of the respective unprotected tetra‐, tri‐, pyro‐, and monophosphopeptides (Figure S78). The products from this distribution were separable by RP‐HPLC and identified by liquid chromatography–mass spectrometry (LC–MS). Currently, we are unable to rationalize this unusual observation.

To access peptide triphosphates, we utilized benzyl‐protected **2 a** (Figure 3C). In the presence of Mg^2+^, this diphosphorylation reagent readily converted phosphopeptides to mono‐benzyl peptide triphosphates (**Bn‐p_3_‐Peps**). Following typical hydrogenolysis conditions with 1 atm of H_2_ and 10 wt % Pd/C, the benzyl groups were removed to give peptide triphosphates (**p_3_‐Peps**). These two reactions can be performed sequentially in one pot.

Curiously, in both the crude reaction mixtures and purified products, we frequently noticed Al^3+^ and Fe^3+^ adducts as the most prominent species during LC–MS measurements—independent of the peptide sequence, protecting group, and phosphate chain length (P_n_=3–6). These ions likely stem from the HPLC and MS instruments.^[54]^ In most cases, two separate peaks were observed by RP‐HPLC, which could be characterized as either the aluminum or the iron species. The propensity of these condensed phosphates to interact so strongly with trivalent metal ions may be exploited in future enrichment strategies for oligophosphorylated peptides and proteins.[^55^, ^56^]

Altogether, a set of oligophosphorylated peptides with defined chain lengths was synthesized and isolated by standard RP‐HPLC methods. The functional diversity of the phosphopeptide substrates underscores the effectiveness of the reported oligoP‐imidazolide reagents.

**Figure 4:**
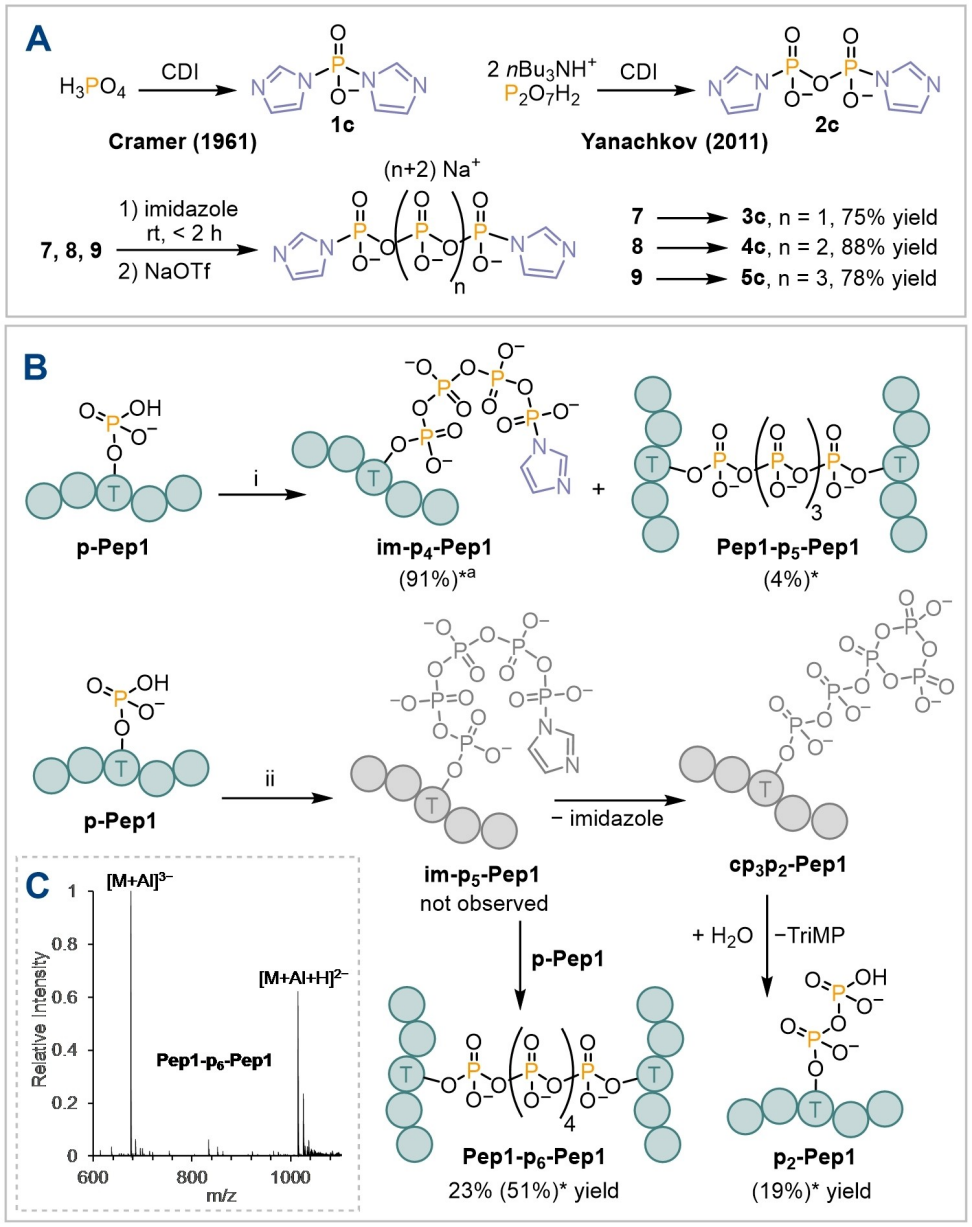
(A) Synthesis of oligoP‐diimidazolide reagents. (B) Reactivity of **3 c** and **4 c** with phosphopeptide **p‐Pep1**. (C, inset) High‐resolution ESI mass spectrum of **Pep1‐p_6_‐Pep1**. Conditions: i) 8 equiv. **3 c**, 9 : 1 DMA/H_2_O, 40 min, 45 °C. ii) 8 equiv. **4 c**, 9 : 1 DMA/H_2_O, 1 h, 45 °C. (*RP‐HPLC yield). ^a^Conversion to **im‐p_4_‐Pep1** was determined by quantifying the hydrolysis product **p_4_‐Pep1** as a proxy.

**Synthesis and Reactivity of OligoP‐diimidazolides**: In tandem with our investigation in the application of asymmetric oligoP‐imidazolides, we briefly explored the reactivity of symmetric oligoP‐diimidazolides; condensed phosphates flanked by two imidazoles (Figure 4). In 1961, Cramer synthesized the first phosphoryl diimidazolide, **1 c**, from phosphoric acid and carbonyl diimidazolide (CDI).^[40]^ Fifty years later, **2 c** was prepared similarly from pyrophosphate.^[41]^ Roy et al. recently showcased the convenient mechanochemical syntheses and one‐pot reactivity of both **1 c** and **2 c**.^[57]^ These two diimidazolides have proven to be useful reagents for the preparation of dinucleoside polyphosphates (Np_n_Ns), a class of signaling molecules implicated in numerous biological processes such as blood coagulation, ocular physiology, and neurotransmission.[^41^, ^57^, ^58^, ^59^]

Here, we now demonstrate that extended oligoP‐diimidazolides (**3 c**, **4 c**, and **5 c**) are obtained easily from precursors **7**, **8**, and **9** by the addition of excess imidazole (Figure 4A). Such bis‐electrophilic species are likely to be useful for the synthesis of Np_n_Ns, but in the context of this study, we sought to explore their reactivity with phosphopeptide substrates (Figure 4B). Subjecting **p‐Pep1** to standard reaction conditions with a stoichiometric amount of **3 c** yielded only a trace amount of the cross‐linked pentaphosphate **Pep1‐p_5_‐Pep1**. The use of excess **3 c** led to the formation of **im‐p_4_‐Pep1** as the major product, as detected by LC–MS. However, attempts to isolate **im‐p_4_‐Pep1** by preparative RP‐HPLC were unsuccessful, as the acidic mobile phase (0.1 % TFA) eventually led to its complete hydrolysis to the tetraphosphate **p_4_‐Pep1**. Curiously, we observed slightly different reactivity with reagent **4 c**, possibly a consequence of its reduced solubility compared to **3 c**. In the reaction between **p‐Pep1** and **4 c**, the formation of **im‐p_5_‐Pep1** was not detected. Instead, we obtained the cross‐linked hexaphosphate **Pep1‐p_6_‐Pep1** as the major product, which was isolated and subsequently characterized by electrospray ionization (ESI)‐MS (Figure 4C, inset). Interestingly, **p_2_‐Pep1** was also generated as a byproduct in this reaction. We propose that **p_2_‐Pep1** results from the intramolecular condensation of unobserved intermediate **im‐p_5_‐Pep1** to cyclic **cp_3_p_2_‐Pep1** and the subsequent hydrolytic dissociation of triMP.

**Pep1‐p_6_‐Pep1** is a novel peptide construct bearing resemblance to materials prepared by the Morrissey group; polyP_n_ (n≈75 or 1000) was activated by 1‐ethyl‐3‐(3‐dimethylaminopropyl)carbodiimide (EDAC) and subsequently “end‐labeled” by various amines, including the *N*‐termini and lysine residues of a few short peptides.[^60^, ^61^] Several examples of end‐labeled condensed phosphates have been utilized as probes in biochemical studies on the activity of bio‐polyP.[^10^, ^62^] It is thus conceivable that peptide‐oligoPs similar to **Pep1‐p_6_‐Pep1** might find similar applications in the future. Additionally, the formation of **p_2_‐Pep1** implies the intermediacy of **cp_3_p_2_‐Pep1**, an example of a cyclic ultraphosphate.^[24]^ Although bio‐polyP is canonically depicted as a linear polymer, cyclic and branched polyPs have been the subject of recent discussion in the literature.[^63^, ^64^, ^65^] A recent study confirmed the coexistence of linear and cyclic polyPs in granular extracts of *Xanthobacter autotrophicus* by solid‐state ^31^P NMR spectroscopy.^[66]^ Studying structures akin to **cp_3_p_2_‐Pep1** may provide insights into the reactivity and potential biological roles of this class of underexplored condensed phosphates.

**Oligophosphorylation of a Full‐length Protein**: To further test the compatibility of this methodology with complex substrates, we next evaluated the reactivity of an oligoP‐imidazolide reagent with a full‐length phosphoprotein. Hitherto, no protein featuring an extended condensed phosphate (P>2) with uniform chain length has been reported.

**Figure 5:**
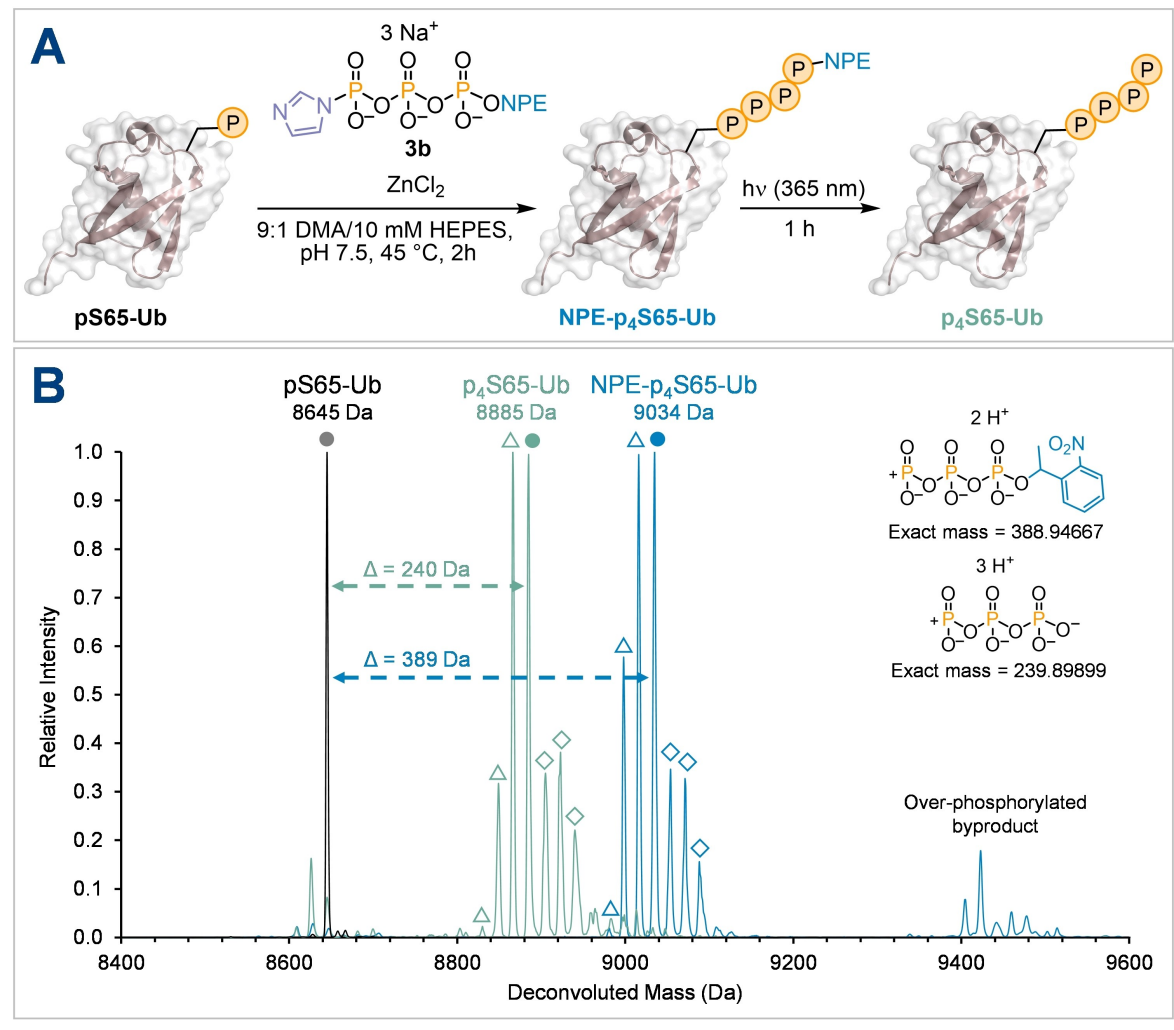
(A) Triphosphorylation of **pS65‐Ub** with **3 b** and subsequent photodeprotection. (B) Overlaid deconvoluted (ESI+) mass spectra of **pS65‐Ub**, **NPE‐p_4_S65‐Ub**, and **p_4_S65‐Ub**. Peak labels: (•) signals of the expected products, (▵) species resulting from the dehydration of said products, and (◊) signals assigned as the adducts to metal ions (Na^+^, K^+^, and Fe^3+^).

**pS65‐Ub**—ubiquitin phosphorylated at Ser65—is a well‐studied and readily available modified protein that was chosen to serve as the model phosphoprotein substrate.^[67]^ In a previous investigation, **pS65‐Ub** was derivatized to the pyrophosphorylated protein **p_2_S65‐Ub** by treatment with P‐imidazolide **1 a**; this modification occurred site‐selectively and only minor off‐target phosphorylation was observed.^[19]^ To synthesize the longer homolog, **pS65‐Ub** was subjected to similar conditions with triphosphorylation reagent **3 b** in the presence of ZnCl_2_ in a 9 : 1 DMF/aqueous buffer solution at 45 °C (Figure 5A). After two hours of incubation, **pS65‐Ub** was consumed, and the major species detected by ESI‐MS was the NPE‐protected tetraphosphorylated protein **NPE‐p_4_S65‐Ub**. Irradiation of the sample with UV light (365 nm) led to the photocleavage of the NPE‐group, yielding the tetraphosphate **p_4_S65‐Ub**, the first reported example of a synthetic oligophosphorylated protein (Figure 5B). The deconvoluted masses of the peaks assigned to these products (labeled •) are in excellent agreement with their calculated values. Some over‐phosphorylated product—ubiquitin featuring two oligoP chains—was also detected. The extent of this off‐target modification is similar to that in reactions with **1 a**.^[19]^

Key features to note in Figure 5B are the multiple peaks adjacent to the product peaks, which were not observed previously with reagent **1 a**. The signals (labeled ◊) with deconvoluted masses larger than the products’ expected values have masses mostly consistent with the product's adducts to metal ions (namely Na^+^, K^+^, and Fe^3+^). As previously discussed, metal‐ion adduction was frequently observed during MS characterization of oligophosphorylated peptides. While these peaks are tentatively assigned to metal ion adducts, we concede the possibility that they are deconvolution artifacts.^[68]^

The peaks to the left of the products’ calculated masses (labeled ▵) correspond to incremental losses of 18 Da, indicating a series of dehydrations. In a control experiment, we found that although wild‐type ubiquitin is not appreciably phosphorylated by **3 b**, both ESI and matrix assisted laser desorption ionization‐time of flight (MALDI‐ToF) MS revealed a distribution of dehydrated products (SI section 11.5). We propose that these peaks arise from the reaction of **3 b** with protein carboxyl groups (Asp, Glu, C‐terminus) to generate short‐lived acyl‐triphosphates. Such activated carboxylates can undergo condensation with proximal amines (Lys, N‐terminus), leading to a net loss of water through isopeptide bond formation. The plausibility of this mechanism is supported by recent literature demonstrating that aminoacyl phosphates can generate peptide oligomers.^[69]^ To rationalize the difference between **3 b** and **1 a**, we point to the results of the hydrolysis kinetics studies (SI section 9) demonstrating that oligoP‐imidazolides are slightly more labile—and thus slightly less chemoselective—than P‐imidazolides.

While further optimization will be required, this experiment demonstrates the potential utility (and limitations) of our new reagents in the conjugation of an oligoP chain to a phosphoprotein—an exciting prospect that might facilitate future studies to probe how oligophosphorylation influences protein structure and function. For such applications, a methodology that is compatible with fully aqueous media would be desirable.

## Conclusion

We have introduced a novel methodology for the chemoselective oligophosphorylation of peptides. This method addresses the challenges associated with studying protein oligo‐ and polyphosphorylation, marking the initial headway for the development of analytical, biochemical, and biophysical methods for investigating these complex PTMs. Our set of oligoP‐imidazolide reagents enabled the site‐selective introduction of multiple phosphate groups in a single synthetic step. We have demonstrated the generalizability of this method through a substrate scope study, wherein a variety of functionally diverse phosphopeptides was selectively transformed into monodisperse peptide oligoPs. Moreover, we have shown that this approach extends to a full‐length phosphoprotein.

Access to such a wide range of modified peptides will facilitate the development of a tailored enrichment workflow for oligophosphorylated peptides from biological samples, as well as oligoP‐specific sensor and antibody development. Additionally, we can use these peptides to investigate if and how various phosphatases and polyphosphatases can hydrolyze the phosphoanhydride bonds. Finally, the modified peptides will serve as essential standards to optimize and establish MS/MS techniques for the detection of this set of modifications in biological samples. Only with such analytical tools can we begin to characterize the cellular relevance and regulation of this complex and uncommon phosphorylation mode.

## Supporting Information

See the accompanying Supporting Information document for further experimental details and characterization data.

## Conflict of Interests

The authors declare no conflict of interest.

1

## Supporting information

As a service to our authors and readers, this journal provides supporting information supplied by the authors. Such materials are peer reviewed and may be re‐organized for online delivery, but are not copy‐edited or typeset. Technical support issues arising from supporting information (other than missing files) should be addressed to the authors.

Supporting Information

## Data Availability

The data that support the findings of this study are available in the supplementary material of this article.
