# Supplementary material for: Monodisperse Chemical Oligophosphorylation of Peptides via Protected Oligophosphorimidazolide Reagents
Source: Angew Chem Int Ed Engl. 2024 Dec 16;64(11):e202419147. doi: 10.1002/anie.202419147 (PMC11891630; doi:10.1002/anie.202419147)
Supplement: Supplementary file 1 — Supporting Information [file ANIE-64-e202419147-s001.pdf]

Angewandte Chemie
Eine Zeitschrift der Gesellschaft Deutscher Chemiker

Supporting Information

Monodisperse Chemical Oligophosphorylation of Peptides via Protected Oligophosphorimidazolide Reagents

K. Qian, B. Hanf, C. C. Cummins, D. Fiedler**

Monodisperse Chemical Oligophosphorylation of Peptides via Protected Oligophosphorimidazolide Reagents

†Kevin Qian[1], †Björn Hanf[2,3], Christopher C. Cummins[1*], and Dorothea Fiedler[2,3*]

[1]Department of Chemistry, Massachusetts Institute of Technology, Cambridge MA, Unites States (ccummins@mit.edu)

[2]Leibniz-Forschungsinstitut für Molekulare Pharmakologie (FMP), Berlin, Germany (fiedler@fmp-berlin.de)

[3]Institut für Chemie, Humboldt-Universität zu Berlin, Germany

†Authors Contributed Equally to this Work

*Corresponding Authors

Table of Contents

1 General Considerations 3

2 Synthesis and Characterization of OligoP-imidazolide Reagents 5

2.1 Synthesis of Diphosphorylation Reagents 2a and 2b 5

2.1.1 Synthesis of Precursor 10 5

2.1.2 Synthesis of 2a 5

2.1.3 Synthesis of bis(1-(2-nitrophenyl)ethyl) hydrogen phosphate 8

2.1.4 Preparation of 2b 12

2.2 Synthesis of Triphosphorylation Reagents 3a and 3b 15

2.2.1 Synthesis of 3a 15

2.2.2 Synthesis of 3b 18

2.3 Synthesis of Tetraphosphorylation Reagents 4a/4b 21

2.3.1 Synthesis of 4a 21

2.3.2 Synthesis of 4b 24

2.4 Synthesis of Pentaphosphorylation Reagents 5a/5b 28

2.4.1 Synthesis of Precursor 9 28

2.4.2 Synthesis of 5a 30

2.4.3 Synthesis of 5b 33

2.5 Synthesis of Oligophosphoryl Diimidazolides 36

2.5.1 Synthesis of 3c 36

2.5.2 Synthesis of 4c 38

2.5.3 Synthesis of 5c 41

3 Solid-Phase Peptide Synthesis (SPPS) of Phosphopeptide Substrates 44
4 Synthesis of Pyrophosphorylated Peptides 45
4.1 Synthesis of NPE-protected Pyrophosphopeptides 45
4.2 Photodeprotection of Pyrophosphopeptides 46
5 Synthesis of Oligophosphorylated Peptides 47
5.1 Additional Optimization of Reaction Conditions for Oligophosphorylation 47
5.2 Procedures for Chemical Synthesis of Oligophosphorylated Peptides 50
5.2.1 Synthesis of NPE-protected Oligophosphopeptides 50
5.2.2 Photodeprotection of Oligophosphopeptides 52
5.2.3 Synthesis of Benzyl-protected Triphosphopeptides 54
5.2.4 Debenzylation of Triphosphopeptides 55
6 Peptide HPLC-UV Traces and Mass Spectrometry Characterization 56
6.1 pThr Model Peptides 56
6.2 pSer Model Peptides 68
6.3 NME1 (Thr94) Peptides 73
6.4 SRRM1 (Ser795) Peptides 84
6.5 DEK (Ser303) Peptides 94
6.6 NPM1 (Ser125) Peptides 106
6.7 NOLC1 (Ser84) Peptides 118
7 Reactions of p-Pep1 with OligoP-Diimidazolides 3c and 4c 129
7.1 Reaction with 3c 129
7.2 Reaction with 4c 132
8 NMR spectra of Pentaphosphopeptide Bn-p$_5$-Pep1 134
9 OligoP-imidazolide Hydrolysis Kinetics Study 137
10 Tandem MS/MS Spectrometry 142
11 Protein Oligophosphorylation 144
11.1 wt-Ub 144
11.2 pS65-Ub 145
11.3 NPE-p$_4$S65-Ub 145
11.4 p$_4$S65-Ub 147
11.5 wt-Ub + Reagent 3b 148
12 Bibliography 150

1 General Considerations

Reactions and operations involving peptides and proteins were performed under typical bench-top conditions unless otherwise specified. Chemical syntheses that are described as being performed under inert atmospheric conditions were conducted using conventional Schlenk techniques or inside a Vacuum Atmospheres model MO-40M glovebox under an atmosphere of purified nitrogen. All solvents used in the glovebox were obtained anhydrous and oxygen-free by bubble degassing with argon and purification through columns of alumina and Q5 by the method of Grubbs.[1] Commercially available chemicals were purchased from Millipore-Sigma, TCI America, Carl Roth, Alfa Aesar, Ambeed, Thermo Fisher Scientific, Strem Chemicals, Iris Biotech, and Anaspec; these chemicals were used as received without further purification unless otherwise stated. Deuterated solvents were purchased from Cambridge Isotope Labs and Deutero GmbH.

^{1}H, ^{13}C, and ^{31}P NMR spectra were recorded at ambient temperature (ca. 22 °C) on a Bruker 400, 500, and 600 AVANCE III spectrometers. Chemical shifts are reported in δ ppm, and multiplicities are reported by peak identity (s = singlet, d = doublet, t = triplet, q = quartet, dd = doublet of doublets, dt = doublet of triplets, m = multiplet, br = broad) and coupling constant (*J*, Hz). NMR spectra were processed and analyzed with TopSpin 3.5 and Mestrenova 14.3 software.

Photochemical reactions were conducted using consumer grade ultra-violet (UV)-LED lamps. Samples were irradiated with either a Navanino Sun X9 Plus UV-LED lamp (λ = 365 nm, 48 W, AC 100–240 V, 50/60 Hz) or an Everbeam UV-LED Black Light (λ = 365 nm, 50 W, AC 240 V). Reaction vessels were placed on ice and irradiated from the top.

To monitor the conversion of peptide intermediates during reaction, **LC-MS measurements** were performed on an Agilent Infinity 1260 LC system with a binary solvent delivery system (module no. G1312B), an autosampler (module no. G1367E), a column oven (module no. G1316A), a diode-array detector (module no. G4212B), coupled to a quadrupole low-resolution mass spectrometer (module no. G6130B), running on water and acetonitrile, both with 0.1% formic acid. The software Agilent OpenLab CDS ChemStation Edition was used. Conversions for peptide phosphorylation reactions were quantified based on the area-under-the-curve (AUC) in the UV chromatogram at λ = 274 nm or in the total-ion-chromatogram (TIC) for peptide substrates that lacked any aromatic moieties.

To purify crude peptides, **preparative high-performance liquid chromatography** (HPLC) was performed on Agilent Infinity 1260 II LC systems running on Agilent OpenLab CDS ChemStation Edition software. **Method I**: Collected on an instrument with a binary solvent delivery system (module no. G7161A), an autosampler (module no. G7157A), a diode-array detector (module no. G7114A), and a fraction collector (module no. G7159B), using a YMC-Actus Triart 5 µm C18 column (150 x 20 mm). Solvents: A = Milli-Q water + 0.1% trifluoroacetic acid (TFA), B = acetonitrile + 0.1% TFA; Gradient: 5% B for 5 min, 5–40% B for 15 min, 40–90% B for 1 min, 90% B for 3 min; Flow rate: 30 mL/min; Detection: 220 nm, 280 nm. **Method II**: Collected on an instrument with a binary solvent delivery system (module no. G7161A), a manual injector (module no. G1328D), a diode-array detector (module no. G7115A), and a fraction collector (module no. G1364E), using a Zorbax SB-C8 5 µm column (250 x 21.2 mm). Solvents: A = Milli-Q water + 0.1% TFA, B = acetonitrile + 0.1% TFA; Gradient: 10% B for 2 min, 10–30% B for 20 min, 30–90% B for 2 min; Flow rate: 21 mL/min; Detection: 214, 254, and 274 nm.

For the characterization of isolated peptides, **HPLC-UV traces** were obtained on an Agilent Infinity 1260 LC systems running on Agilent OpenLab CDS ChemStation Edition software. **Method A**: Collected on an instrument fitted with a quaternary solvent delivery system (module no. G1311B), an autosampler (module no. G1329B), a column oven (module no. G1316C), a diode-array detector (module

no. G4212B), and a fraction collector (module no. G1364C), using a YMC-Triart 5 µm C18 column (150 x 3.0 mm). Solvents: A = Milli-Q water + 0.1% TFA, B = acetonitrile + 0.1% TFA; Gradient: 5% B for 1 min, 5–40% B for 15 min, 40–90% B for 1 min, 90% B for 3 min; Flow rate: 1.0 mL/min; Temperature: 25 °C; Detection: 214 nm. **Method B**: Collected on an instrument fitted with a binary solvent delivery system (module no. G7112B), an autosampler (module no. G7167A), a column oven (module no. 7116A), a diode-array detector (module no. G7117C), and a fraction collector (module no. G1364F), using a Zorbax SB-C8 3.5 µm column (150 x 4.6 mm). Solvents: A = Milli-Q water + 0.1% TFA, B = acetonitrile + 0.1% TFA; Gradient: 5% B for 1 min, 5–40% B for 14 min, 40–90% B for 1 min, 90% B for 3 min; Flow rate: 1.0 mL/min; Temperature: 25 °C; Detection: 214 nm, 254, and 274 nm.

For the characterization of isolated peptides, **high-resolution mass spectrometry** (HRMS) spectra were acquired either on a Xevo G2-XS QToF (Waters) high-resolution mass spectrometer coupled to an Acquity UPLC system using the MassLynx software (V4.1, Waters), or on a Q Exactive Orbitrap (Thermo Fisher Scientific) high-resolution mass spectrometer coupled to a Thermo Fisher Scientific UPLC system using the FreeStyle 1.8 SP2 software (Thermo Fisher Scientific), or an Agilent 6545 Q-ToF high-resolution mass spectrometer coupled to an Agilent Infinity 1260 LC system running on Agilent Chemstation, MassHunter, and BioConfirm software, or an Ultivo triple-quadrupole QQQ (Agilent) mass spectrometer coupled to an Agilent Infinity 1260 LC system running on Agilent Chemstation and MassHunter software. All LC systems operate with water and acetonitrile mobile phases, both with 0.1% formic acid. All reported masses were found within a maximal deviation of 10 ppm from the calculated m/z.

2 Synthesis and Characterization of OligoP-imidazolide Reagents

Unless otherwise stated, all chemical syntheses were performed in a Vacuum Atmospheres model MO-40M glovebox under an inert atmosphere of purified nitrogen. All solvents used in the glovebox were obtained anhydrous and oxygen-free by bubble degassing with argon and purification through columns of alumina and Q5 by the method of Grubbs.[1] Pure anhydrous phosphorus pentoxide (≥99.99% trace metals basis) was obtained from Millipore-Sigma (Catalog#: 431419).

2.1 Synthesis of Diphosphorylation Reagents 2a and 2b

2.1.1 Synthesis of Precursor 10

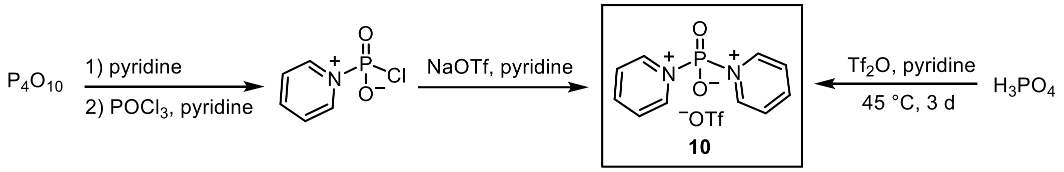

The bis-pyridine adduct **10** can be prepared by two alternative routes. The group of Weigand recently published a gram-scale synthesis of **10** by heating phosphoric acid with trifluoromethanesulfonic anhydride in neat pyridine over the course of several days.[2] We found it convenient to synthesize this reagent starting from phosphorus pentoxide, adapting a route previously reported by Meisel.[3] The treatment of phosphorus pentoxide with pyridine, and subsequent reaction with $POCl_3$ yields the pyridine adduct of phosphenic chloride (pyPO$_2$Cl). Inside the glovebox, a scintillation vial was charged with PyPO$_2$Cl (0.447 g, 2.5 mmol, 1 equiv.), pyridine (ca. 2 mL), and acetonitrile (ca. 5 mL). A solution of sodium trifluoromethanesulfonate (606 mg, 3.5 mmol, 1.4 equiv.) in 5 mL of acetonitrile was added dropwise to the stirring reaction mixture, leading to the precipitation of NaCl. After stirring at ambient temperature for 30 min, the reaction mixture was passed through a 0.2 micron PTFE syringe filter and concentrated under vacuum. The residue was triturated with 3x5 mL of THF, and afforded a colorless crystalline solid after drying under vacuum (732 mg, 80% yield). The spectral data matches with the previously reported characterization data.

2.1.2 Synthesis of 2a

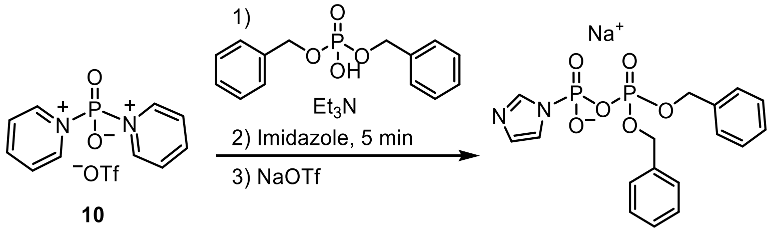

In the glovebox, dibenzylphosphoric acid (150 mg, 0.54 mmol, 1 equiv.) and triethylamine (2 equiv.) were combined with ca. 4 mL of acetonitrile. In a separate vial with a magnetic stir bar, a solution of **10** (200 mg, 0.54 mmol, 1 equiv.) in 4 mL of acetonitrile was prepared. To the stirring solution of **10**, the dibenzylphosphate solution was added dropwise over the course of a minute. This initial reaction is instantaneous and was immediately followed by the addition of imidazole (37 mg, 0.54 mmol, 1 equiv.). After stirring for 5 min, a 2 mL acetonitrile solution containing sodium trifluoromethanesulfonate (190 mg, 1.08 mmol, 2 equiv.) was added to the reaction mixture. The reaction mixture was transferred outside of

the glovebox and into a 50 mL Falcon conical centrifuge tube and then centrifuged at 2,000 RPM for 10 min. The supernatant was decanted, an additional 20 mL of acetonitrile was added to the pellet, which was followed by brief agitation by vortex mixing. The sample was again centrifuged at 2,000 RPM for 10 min, and this washing procedure was repeated for a total of 3 cycles. The product was dried on the vacuum line, yielding pure **2a** as a fine colorless powder (154 mg, 67% yield)

$^{31}P\{^{1}H\}$ NMR (162 MHz, D_2O) δ -13.31 (d, *J* = 21.5 Hz, 1P), -21.05 (d, *J* = 21.7 Hz, 1P).

^{31}P NMR (162 MHz, D_2O) δ -13.31 (dq, *J* = 21.3, 9.8 Hz, 1P), -21.07 (d, *J* = 21.4 Hz, 1P).

^{1}H NMR (400 MHz, D_2O) δ 7.76 (t, *J* = 1.2 Hz, 1H), 7.39–7.19 (m, 10H), 7.16 (d, *J* = 1.7 Hz, 1H), 6.99 (p, *J* = 1.1 Hz, 1H), 4.94 (d, *J* = 10.0 Hz, 4H).

$^{13}C\{^{1}H\}$ NMR (101 MHz, D_2O) δ 139.80 (br), 134.64 (d, *J* = 5.8 Hz), 129.15, 128.80, 128.43, 120.36 (br), 70.91 (d, *J* = 6.1 Hz).

HRMS(−) (m/z) of $C_{17}H_{17}N_2O_6P_2^-$ $[M]^-$: 407.0577 (calc’d 407.0567)

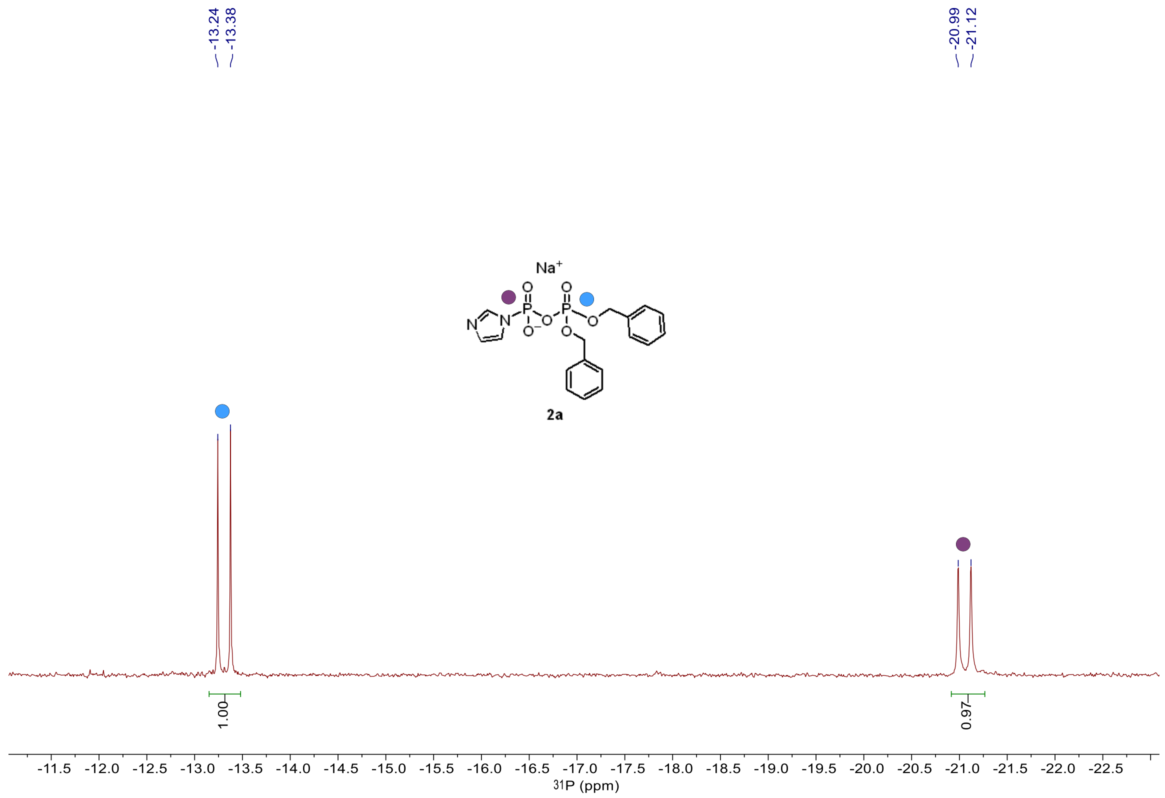

Figure S1: $^{31}P\{^{1}H\}$ NMR spectrum of **2a.**

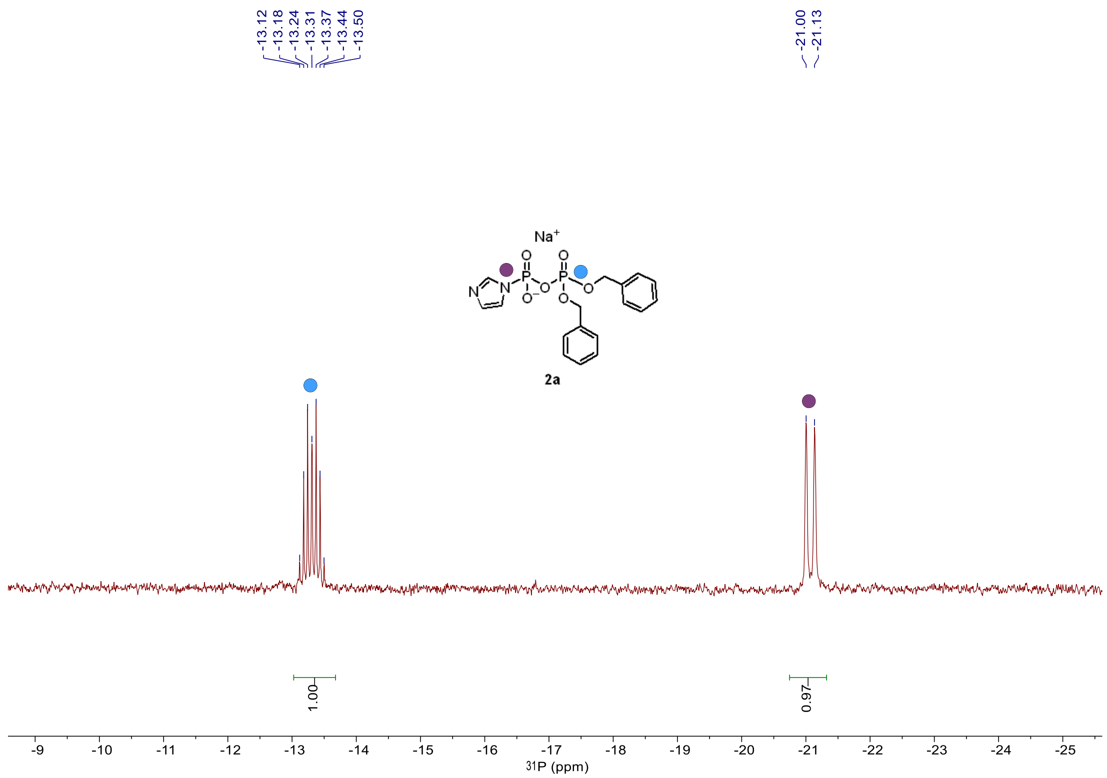

Figure S2: ^{31}P NMR spectrum of **2a**.

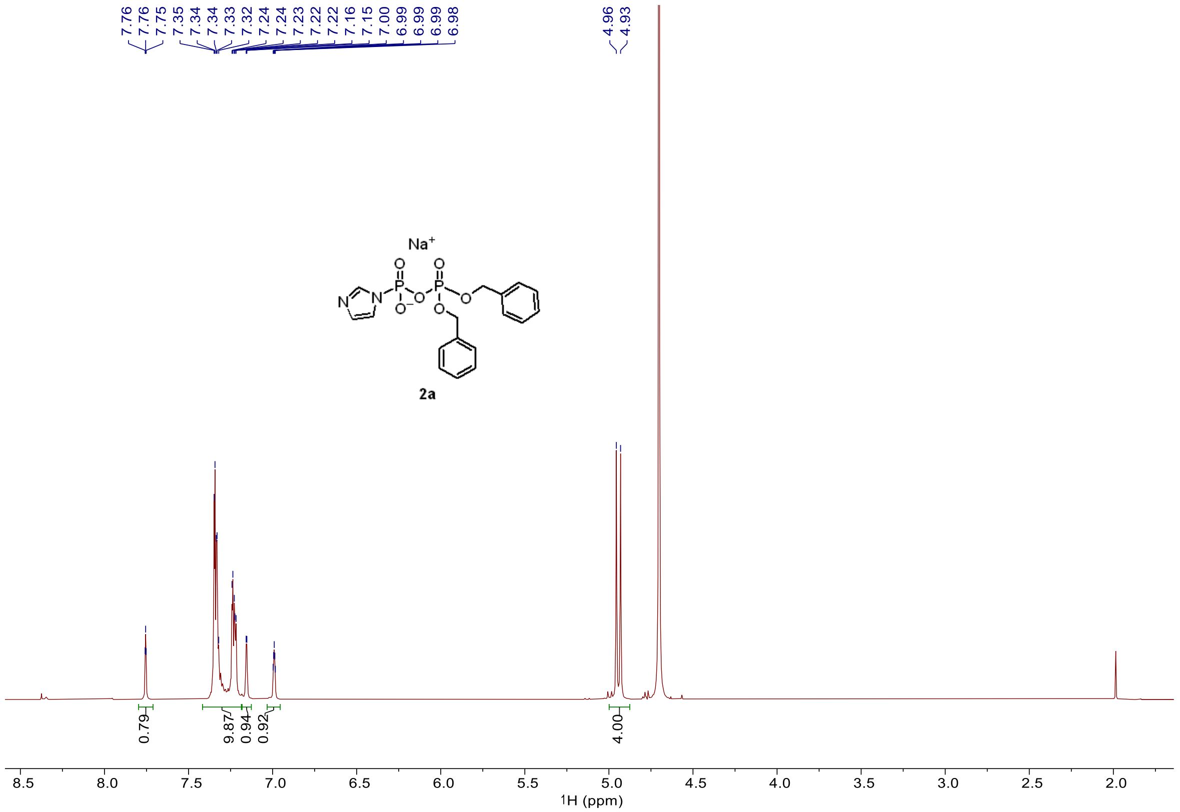

Figure S3: ^{1}H NMR spectrum of **2a**.

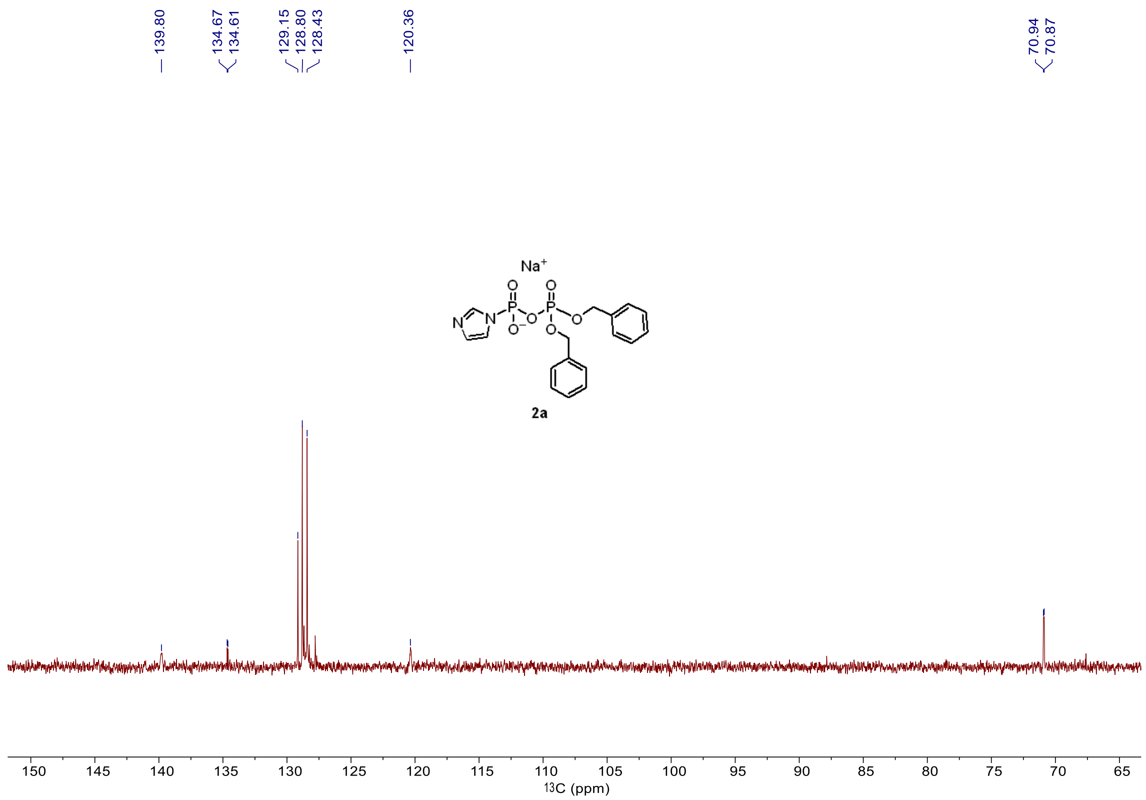

Figure S4: $^{13}C\{^{1}H\}$ NMR spectrum of **2a**.

2.1.3 Synthesis of bis(1-(2-nitrophenyl)ethyl) hydrogen phosphate

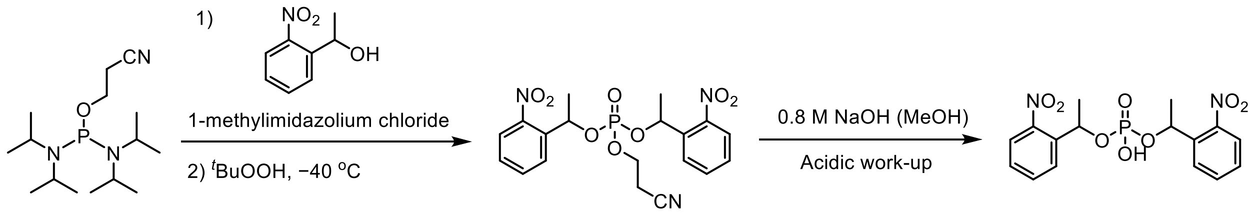

This procedure was adapted from one we previously published for the mono-NPE phosphate ester.[4] Under an inert atmosphere, 2-cyanoethyl N,N,N′,N′-tetraisopropylphosphorodiamidite (1.00 g, 3.32 mmol, 1 equiv.) and 1-(2-nitrophenyl)ethanol (1.16 g, 6.97 mmol, 2.1 equiv.) were combined in 15 mL of dry acetonitrile. To this stirring mixture was added 1-methylimidazolium chloride (1.18 g, 9.95 mmol, 3 equiv.), leading to the immediate formation of a colorless precipitate. After stirring at room temperature for 1 h, the reaction vessel was transferred to an acetonitrile/dry-ice bath (−40 °C). *Tert*butylhydroperoxide as 5.5 M solution in decane (1 mL, 5.64 mmol, 1.7 equiv.) was added to the chilled solution. After 2 h, the reaction was quenched with 25 mL of $Na_2S_2O_3$ (5% w/v) and stirred for 20 min. The reaction mixture was diluted with 80 mL of ethyl acetate in a separatory funnel, and the organic layer was collected. The aqueous layer was further extracted with 2×80 mL of ethyl acetate. The combined organic layers were washed with 2x60 mL deionized water, dried with magnesium sulfate, and concentrated under vacuum. The crude product was then purified by column chromatography with a solvent gradient of 0-10% methanol in diethyl ether; the desired product had an $R_F \approx 0.7$ on a TLC plate in 1:9 MeOH/Et_2O, and eluted out as the second band. Pure 2-cyanoethyl bis(1-(2-nitrophenyl)ethyl) phosphate was obtained as a yellow oil (0.95 g, 65% yield).

*Due to the chirality of the nitrophenylethyl groups (and because 1-(2-nitrophenyl)ethanol is purchased as a racemic mixture), the ^{31}P NMR spectra display multiple peaks corresponding to a mixture of diastereomers.

^{31}P{^{1}H} NMR (162 MHz, $CDCl_3$) δ [-3.89, -4.36, -4.40] (sum 1P).

^{31}P NMR (162 MHz, $CDCl_3$) δ [-3.89 (p, *J* = 7.8 Hz), -4.36 (p, *J* = 7.6 Hz), -4.40 (p, *J* = 7.5 Hz)] (sum 1P).

^{1}H NMR (400 MHz, $CDCl_3$) δ 8.00–7.30 (8H), 6.05 (2H), 4.15 (2H), 2.65 (2H), 1.76–1.55 (6H).

2-cyanoethyl bis(1-(2-nitrophenyl)ethyl) phosphate (0.72 g, 1.6 mmol) was dissolved in 20 mL of a 0.8 M NaOH solution in methanol and heated to 50 °C for 1 h, resulting in a pale-yellow solution. Methanol was evaporated under vacuum, and to the residue was added 100 mL of an aqueous 0.5 M HCl solution. The aqueous layer was extracted with ethyl acetate (4×80 mL), and the combined organic layers were dried with magnesium sulfate, filtered, and concentrated under reduced pressure. The product was afforded as a dark yellow oil, which solidified after placing in a -20 °C freezer overnight (0.51 g, 81% yield).

*Due to the chirality of the nitrophenylethyl groups (and because 1-(2-nitrophenyl)ethanol is purchased as a racemic mixture), the ^{31}P NMR spectra display multiple peaks corresponding to a mixture of diastereomers.

^{31}P{^{1}H} NMR (162 MHz, $CDCl_3$) δ [-1.50, -1.61] (sum 1P).

^{31}P NMR (162 MHz, $CDCl_3$) δ [-1.50 (t, *J* = 7.4 Hz) , -1.61 (t, *J* = 7.4 Hz)] (sum 1P).

^{1}H NMR (400 MHz, $CDCl_3$) δ 8.05–7.35 (8H), 6.1–5.90 (2H), 5.85 (br, 1H), 1.60–1.53 (6H).

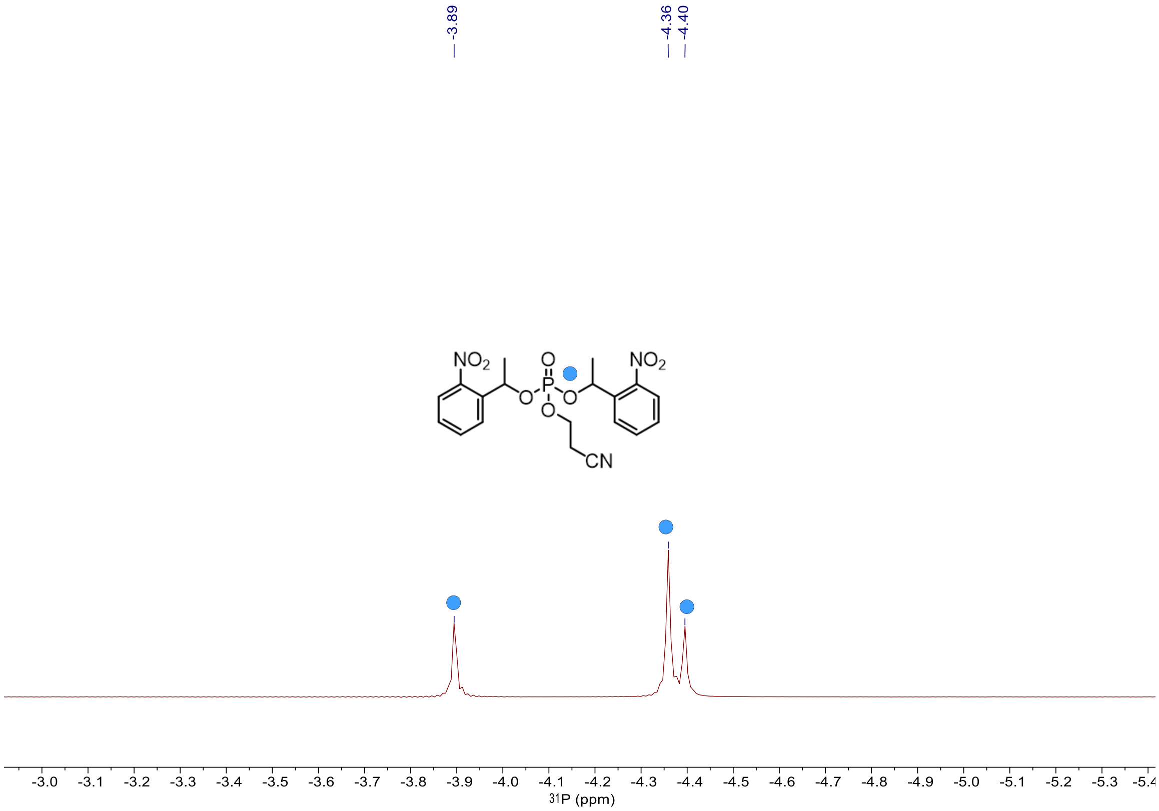

Figure S5: ^{31}P{^{1}H} NMR spectrum of 2-cyanoethyl bis(1-(2-nitrophenyl)ethyl) phosphate.

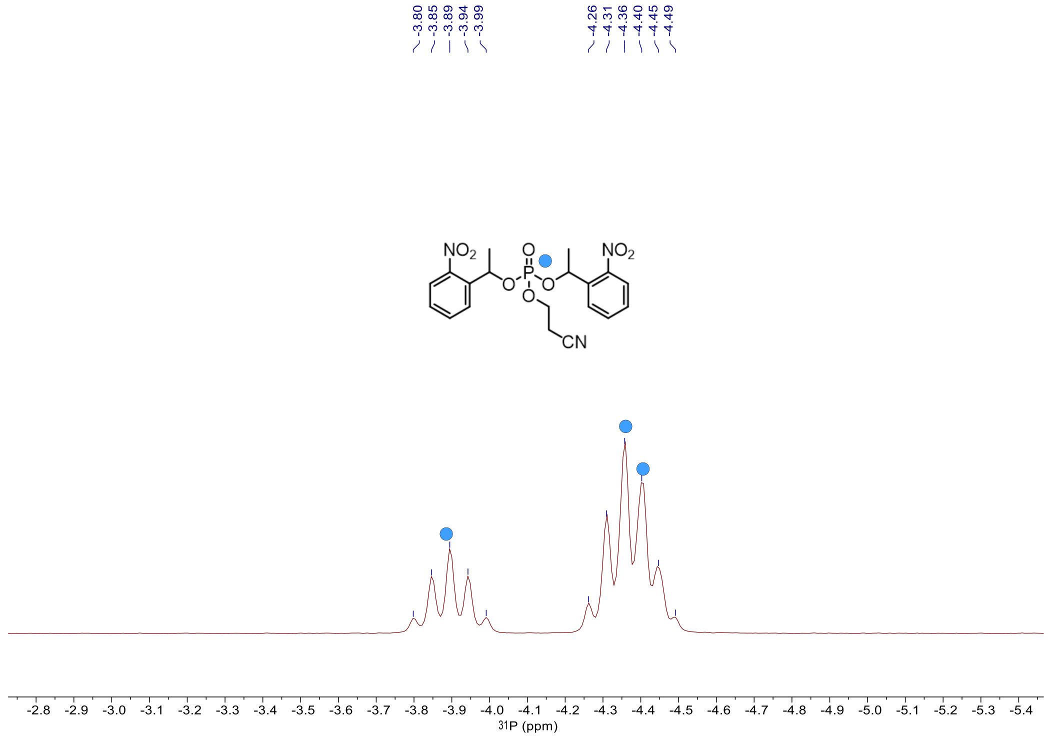

Figure S6: ^{31}P NMR spectrum of 2-cyanoethyl bis(1-(2-nitrophenyl)ethyl) phosphate.

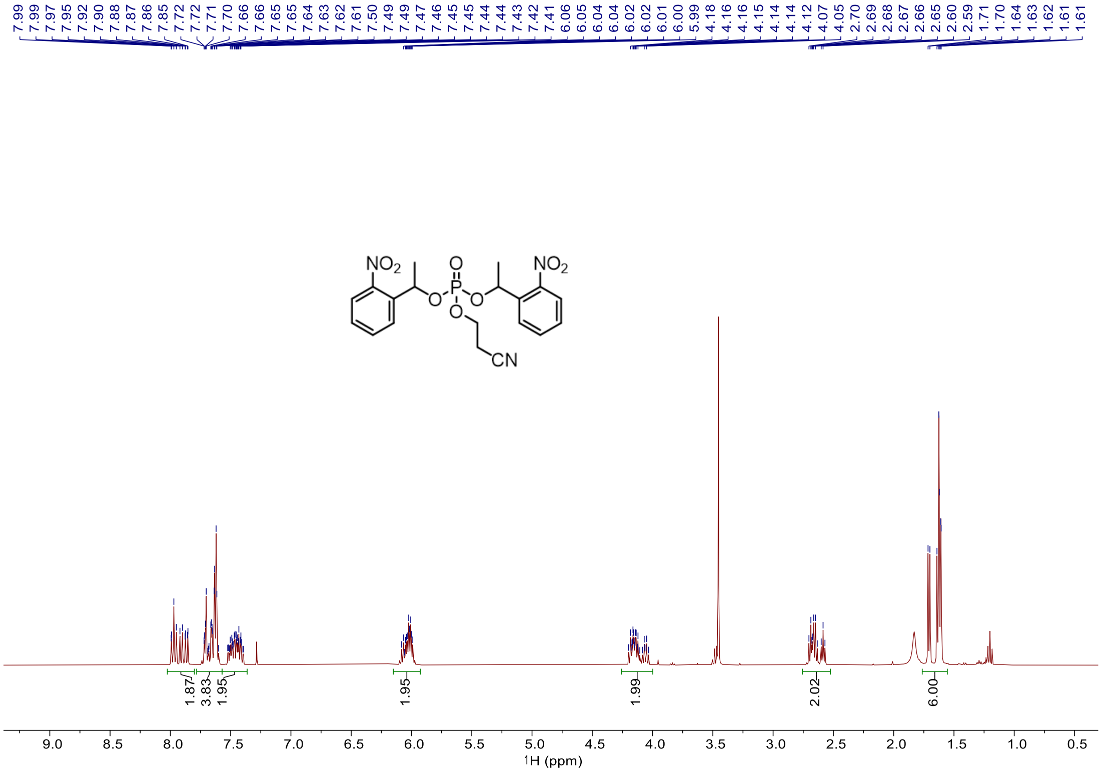

Figure S7: ^{1}H NMR spectrum of 2-cyanoethyl bis(1-(2-nitrophenyl)ethyl) phosphate.

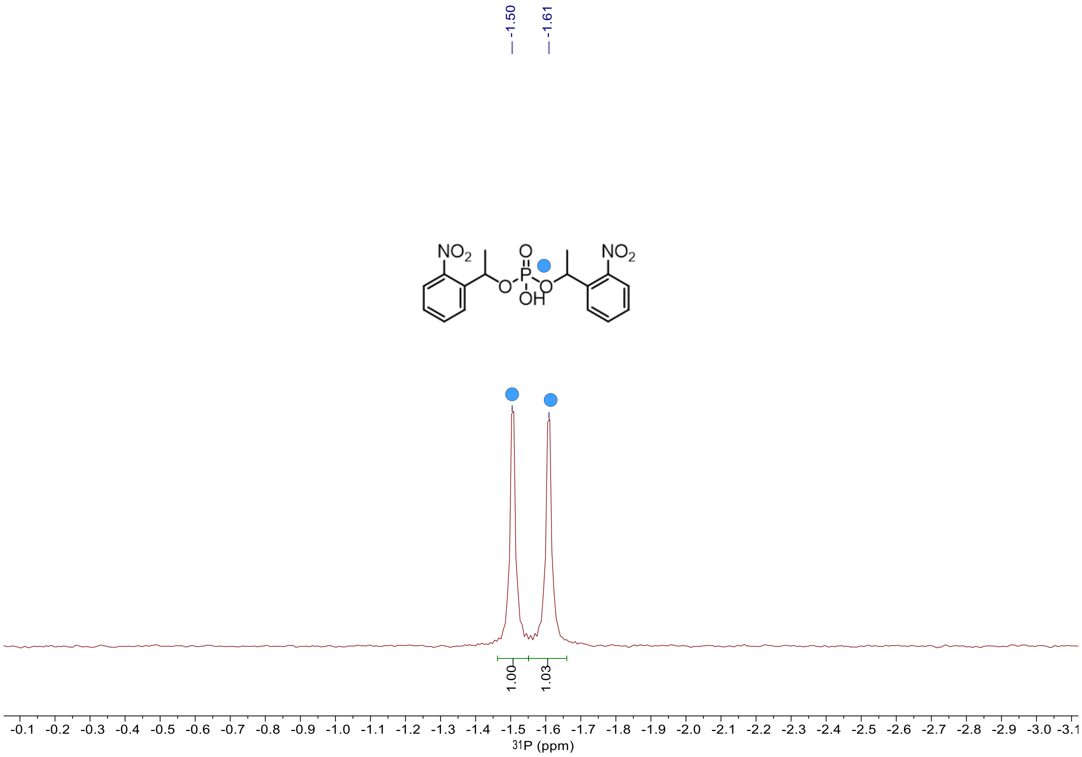

Figure S8: $^{31}P\{^{1}H\}$ NMR spectrum of bis(1-(2-nitrophenyl)ethyl) hydrogen phosphate.

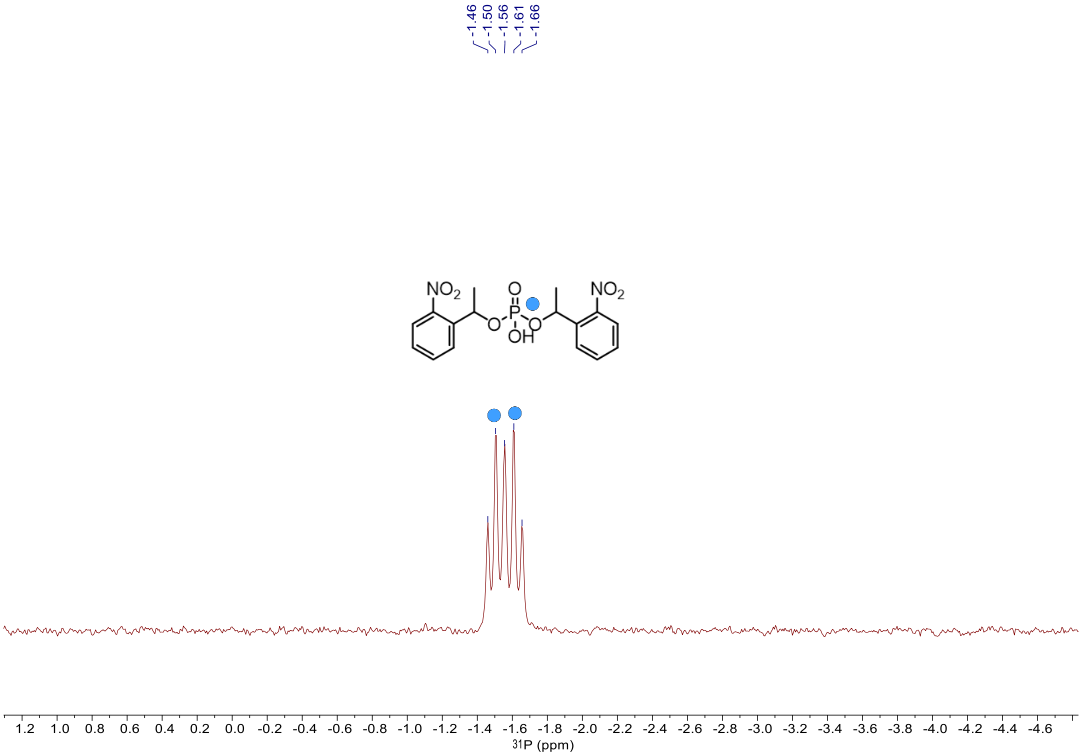

Figure S9: ^{31}P NMR spectrum of bis(1-(2-nitrophenyl)ethyl) hydrogen phosphate.

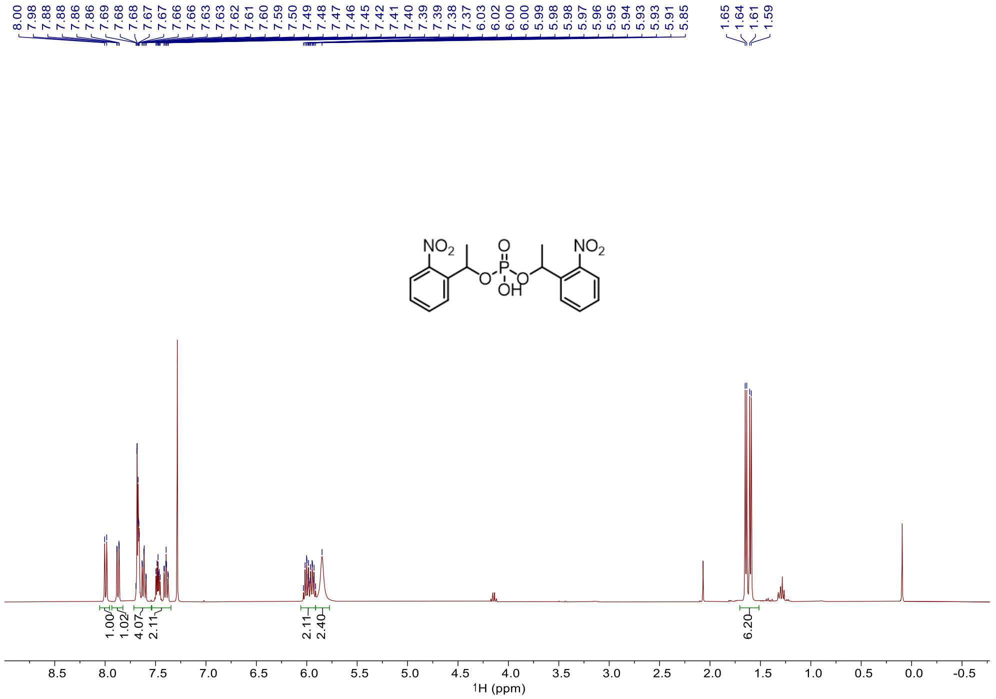

Figure S10: ^{1}H NMR spectrum of bis(1-(2-nitrophenyl)ethyl) hydrogen phosphate.

2.1.4 Preparation of 2b

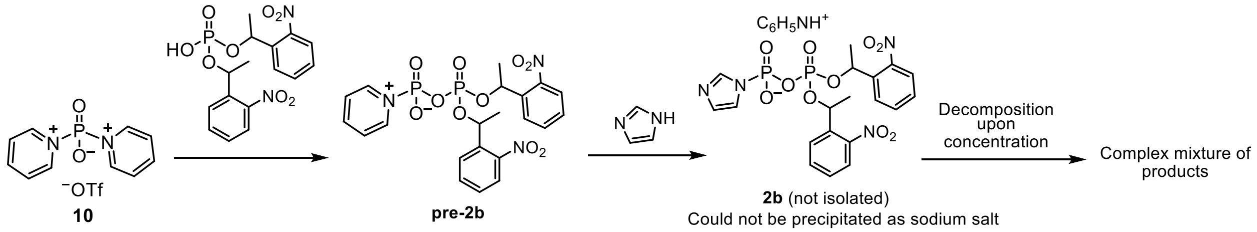

Inside the glovebox within a scintillation vial, **10** (90 mg, .243 mmol, 1 equiv.) was dissolved in ca. 5 mL of dry acetonitrile. A separate solution of bis(1-(2-nitrophenyl)ethyl) hydrogen phosphate (96 mg, .243 mmol, 1 equiv.) in 5 mL of acetonitrile was prepared, and was added dropwise at room temperature to the stirring solution of **10**. The immediate formation of the zwitterionic intermediate **pre-2b** was observed by ^{31}P NMR spectroscopy. Then, 1 equiv. of imidazole (NOTE: avoid addition of excess imidazole) was added to the mixture, leading to the clean formation of compound **2b**.

Unfortunately, in contrast to compound **2a**, the pyrophosphoryl-imidazolide **2b** could not be easily obtained by precipitation via counterion exchange with sodium trifluoromethanesulfonate, as the nitrophenylethyl groups impart enhanced solubility to the pyrophosphate. Furthermore, concentration under reduced pressure leads to the decomposition of **2b**, presumably due to condensation reactions with itself that become apparent over the course of several hours at room temperature. Given the poor stability of **2b**, its reactivity towards phosphopeptides was not explored in this present study.

We hypothesize that the NPE groups decrease the stability of **2b** in two ways: (1) the electron-poor arenes enhance the phosphate diester's propensity to act as a leaving group or a P-centered electrophile, and (2) the increased lipophilicity of the NPE groups prevents the isolation of **2b** as a crystalline sodium salt, leaving it in an oil phase after concentration. The other NPE oligoPs (**3b**, **4b**, **5b**) are more stable because they contain terminal phosphate monoesters, which are less labile than phosphate diesters. Similarly, analog **2a** is stable since the benzyl groups are much less electron-withdrawing than NPE.

*Due to the chirality of the nitrophenylethyl groups (and because 1-(2-nitrophenyl)ethanol is purchased as a racemic mixture), the ^{31}P NMR spectra display multiple peaks corresponding to a mixture of diastereomers.

Intermediate Pre-2b: ^{31}P{^{1}H} NMR (202 MHz, CH_3CN) δ [-15.41 (d, *J* = 20.1 Hz), -15.88 (d, *J* = 19.5 Hz), -16.13 (d, *J* = 18.7 Hz)] (sum: 1P), -21.23 (m, 1P).

Compound 2b: ^{31}P{^{1}H} NMR (202 MHz, CH_3CN) δ [-15.30 (d, *J* = 18.7 Hz), -15.82 (d, *J* = 18.2 Hz), -16.07 (d, *J* = 17.4 Hz)] (sum: 1P), -26.03 (q, *J* = 17.8 Hz, 1P).

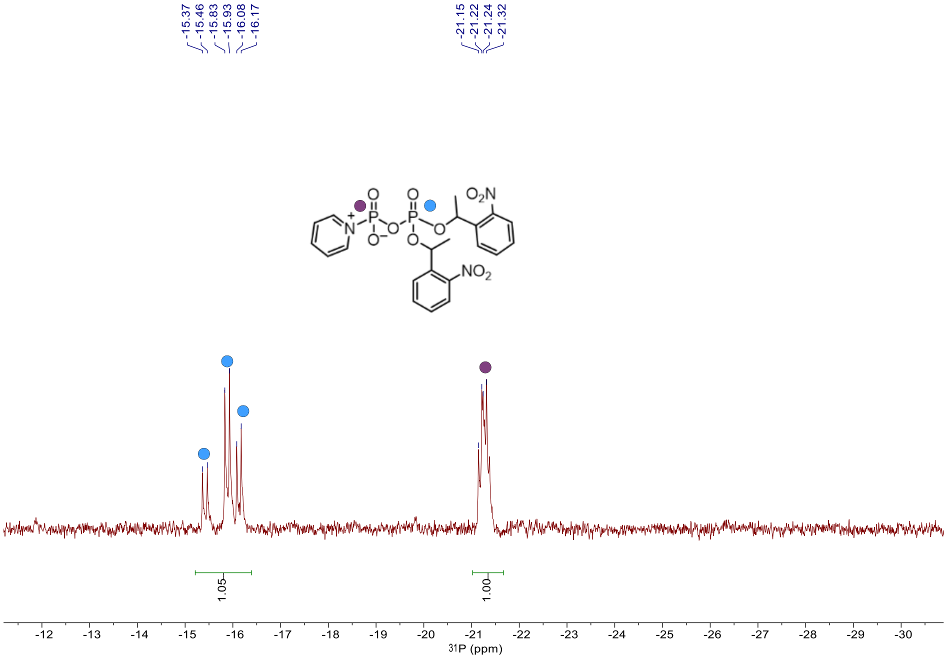

Figure S11: ^{31}P{^{1}H} NMR spectrum of intermediate **pre-2b.**

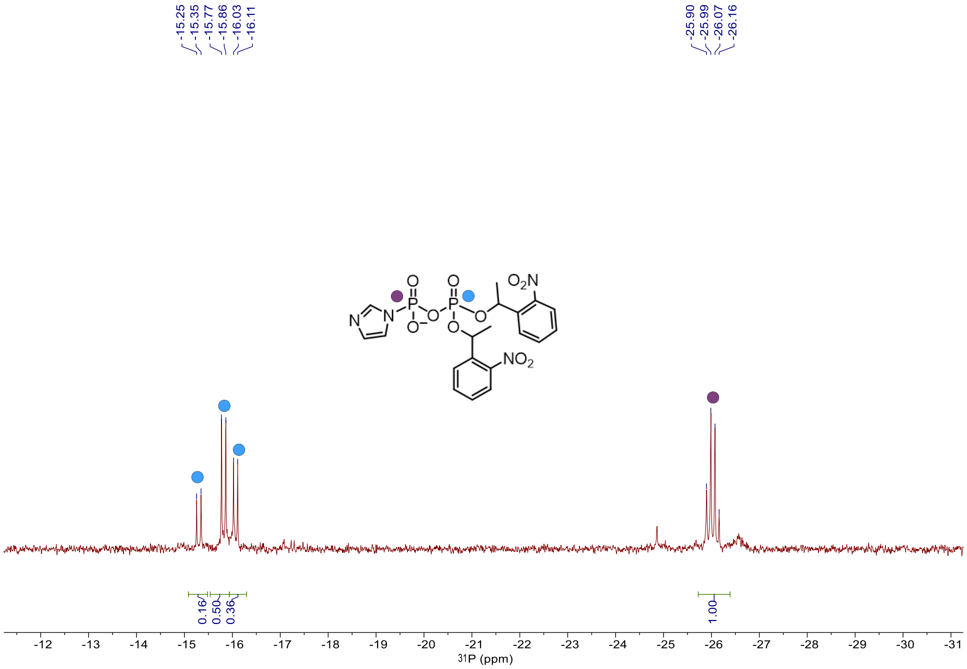

Figure S12: ^{31}P{^{1}H} NMR spectrum of **2b.**

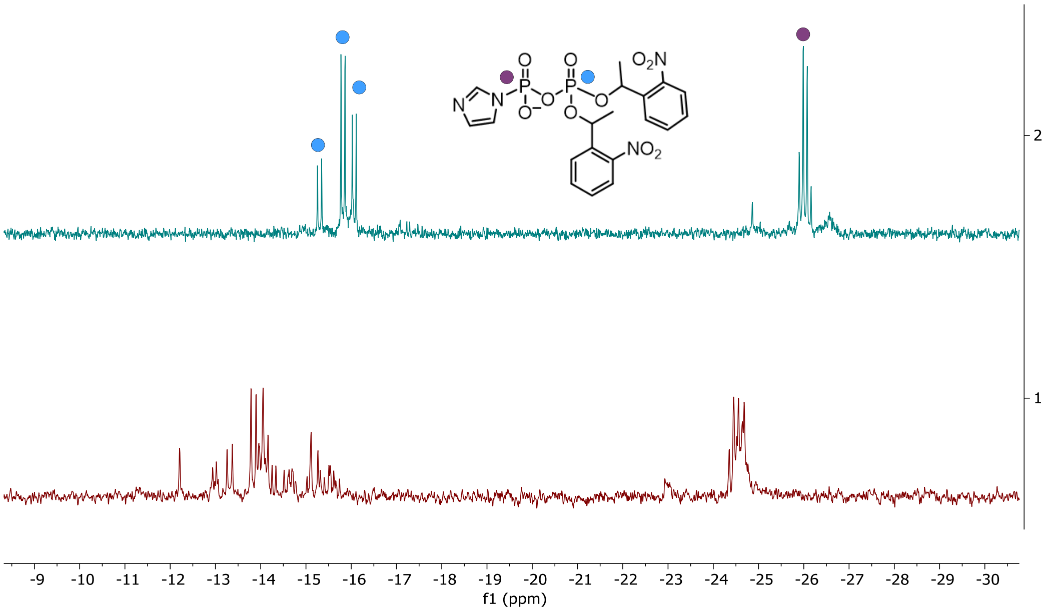

Figure S13: $^{31}P\{^{1}H\}$ NMR spectra of (top) freshly prepared compound **2b** and (bottom) the decomposition products of **2b** after concentration.

2.2 Synthesis of Triphosphorylation Reagents 3a and 3b

2.2.1 Synthesis of 3a

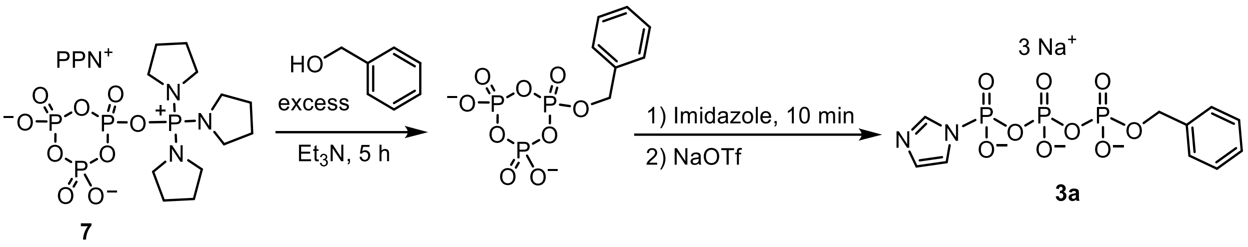

Precursor **7** was prepared as previously reported.[5] Under an inert atmosphere, a solution of **7** (105 mg, 0.10 mmol, 1 equiv.) in ca. 1 mL of acetonitrile was treated with excess benzyl alcohol (2.16 g, 19.95 mmol, 200 equiv.) and triethylamine (50 µL, 0.36 mmol, 3.6 equiv.). The yellow mixture was stirred at room temperature for 5 h, forming the intermediate benzyl trimetaphosphate. This compound was ring-opened by the addition of imidazole (135 mg, 2.0 mmol, 20 equiv.) followed by 10 min of stirring. The addition of a solution of sodium trifluoromethanesulfonate (308 mg, 1.8 mmol, 18 equiv.) in 5 mL of acetonitrile led to the precipitation of the product. The reaction mixture was transferred outside of the glovebox and into a 50 mL Falcon conical centrifuge tube, and centrifuged at 2,000 RPM for 10 min. The supernatant was decanted, and an additional 20 mL of acetonitrile was added to the pellet, which was followed by brief agitation by vortex mixing. The sample was again centrifuged at 2,000 RPM for 5 min, and this washing procedure was repeated for a total of 5 cycles. The product was dried on the vacuum line, yielding pure **3a** as a colorless solid (41 mg, 89% yield).

$^{31}P\{^1H\}$ NMR (162 MHz, D_2O) δ -11.93 (d, *J* = 19.6 Hz, 1P), -20.45 (d, *J* = 19.4 Hz, 1P), -24.19 (t, *J* = 19.8 Hz, 1P).

^{31}P NMR (162 MHz, D_2O) δ -11.93 (dt, *J* = 19.6, 6.8 Hz, 1P), -20.45 (d, *J* = 19.2 Hz, 1P), -24.19 (t, *J* = 19.6 Hz, 1P).

^1H NMR (400 MHz, D_2O) δ 7.87 (s, 1H), 7.42–7.23 (m, 6H), 6.95 (p, *J* = 1.1 Hz, 1H), 4.78 (d, *J* = 6.8 Hz, 2H).

$^{13}C\{^1H\}$ NMR (101 MHz, D_2O) δ 139.99 (d, *J* = 6.7 Hz), 137.26 (d, *J* = 8.3 Hz), 128.67, 128.61 (d, *J* = 12.5 Hz), 128.14, 127.67, 120.44 (d, *J* = 6.5 Hz), 67.88 (d, *J* = 5.7 Hz).

HRMS(−) (m/z) of $C_{10}H_{12}N_2O_9P_3^-$ $[M]^-$: 396.9756 (calc'd 396.9761)

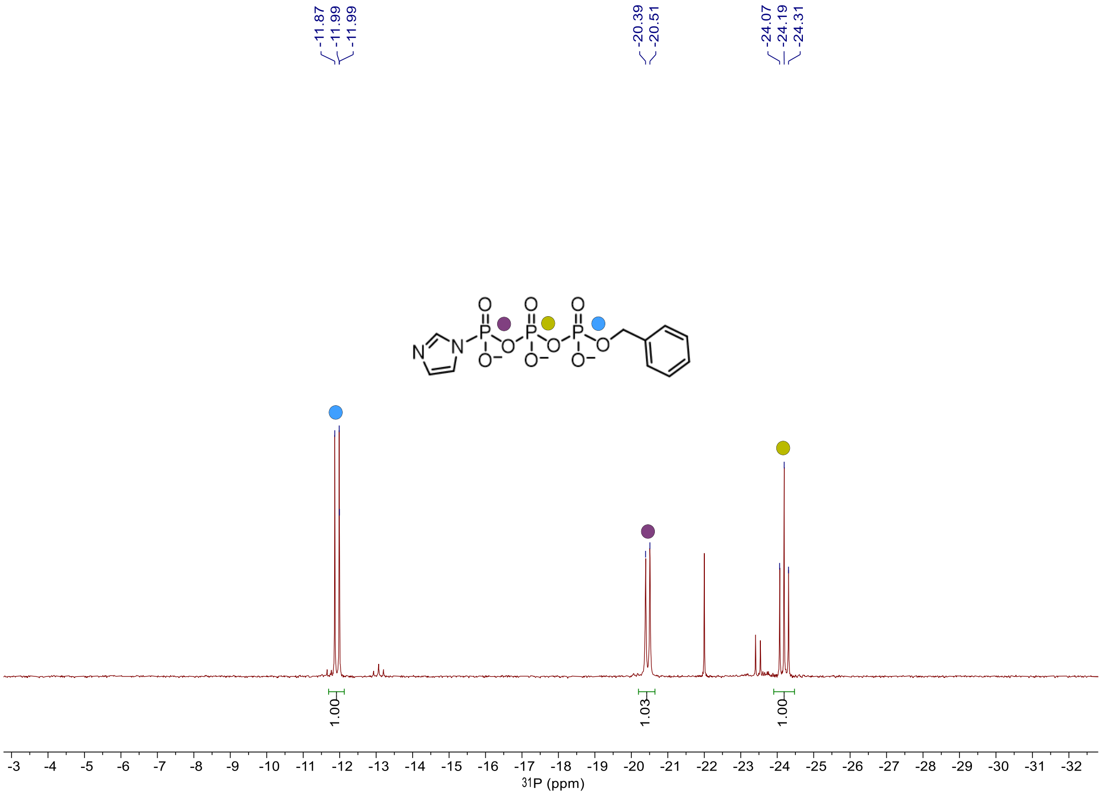

Figure S14: $^{31}P\{^{1}H\}$ NMR spectrum of **3a.**

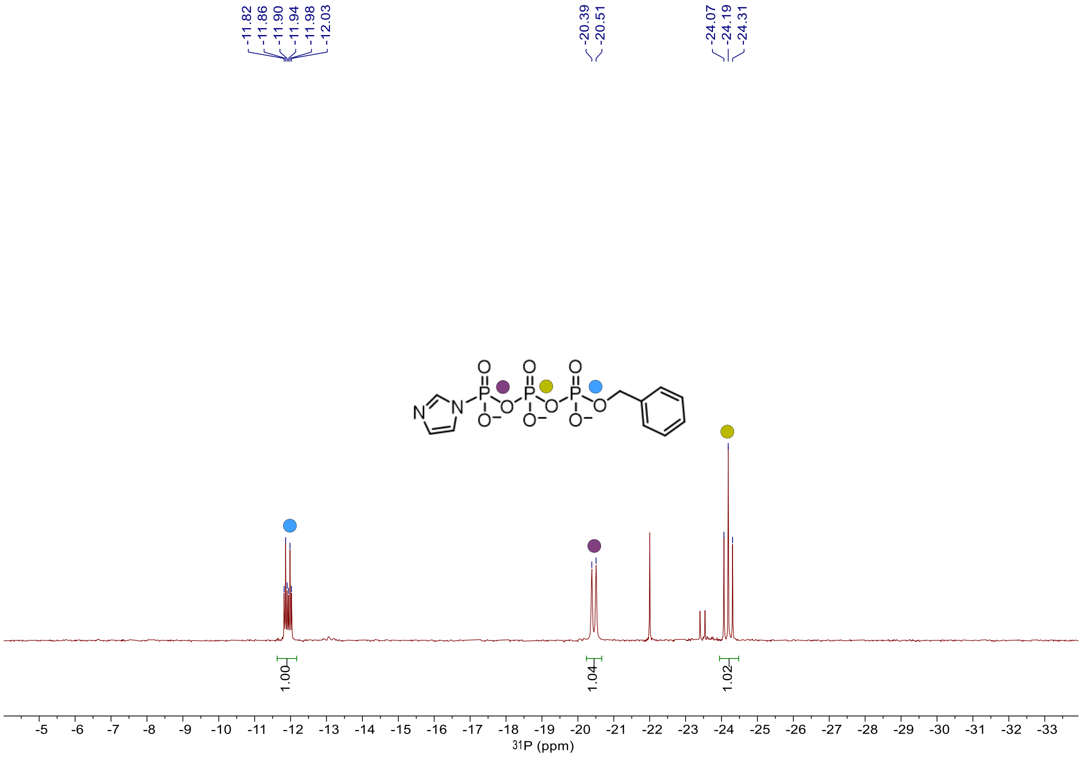

Figure S15: ^{31}P NMR spectrum of **3a.**

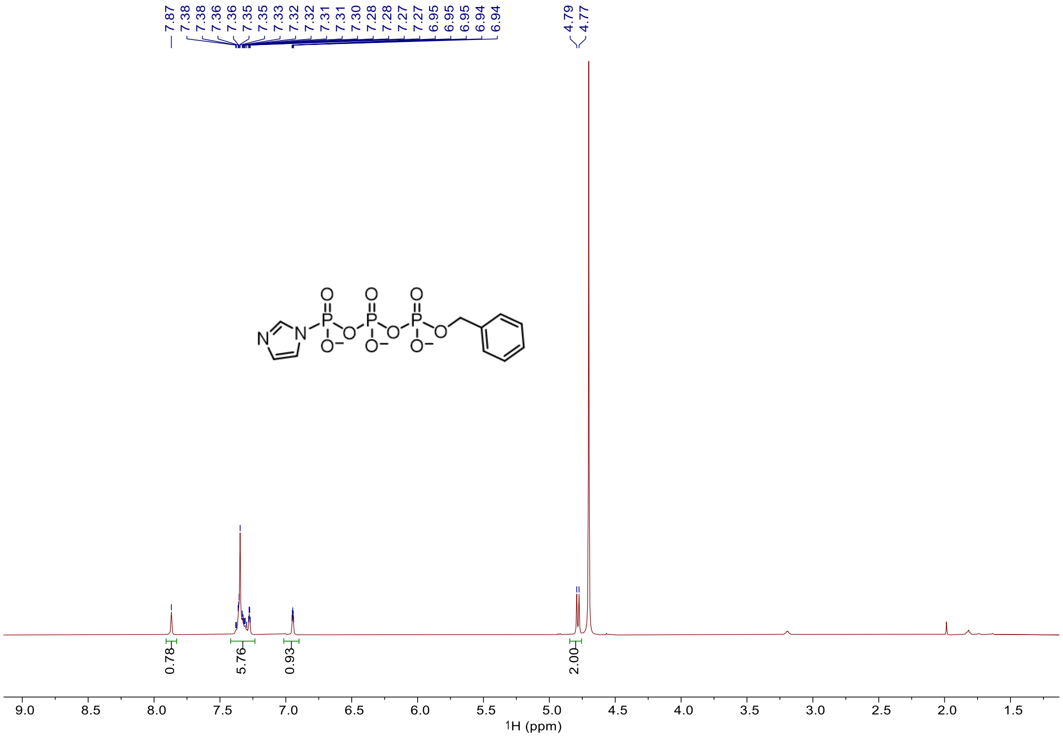

Figure S16: ^{1}H NMR spectrum of **3a.**

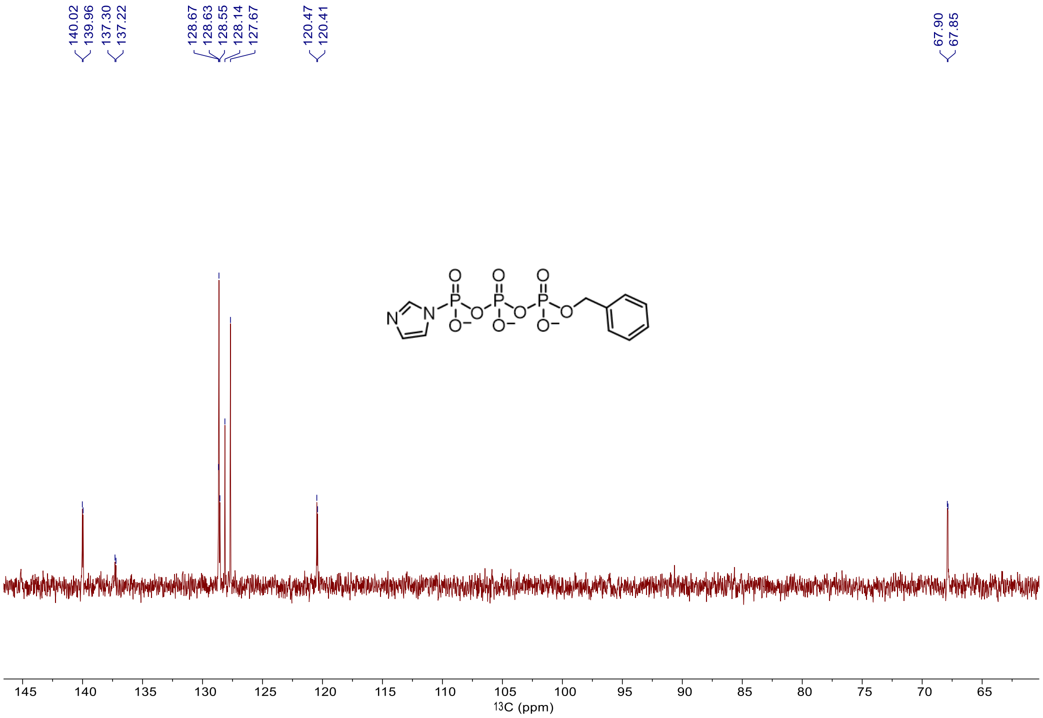

Figure S17: ^{13}C{^{1}H} NMR spectrum of **3a.**

2.2.2 Synthesis of 3b

Reagent **3b** can be prepared *via* two alternative routes.

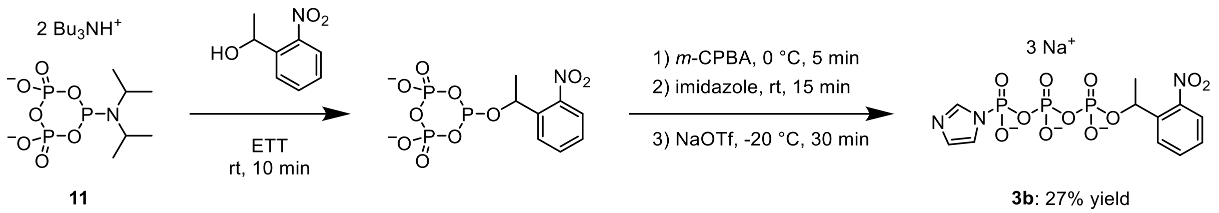

Route 1: Cyclic-pyrophosphoryl-P-amidite (*c*-PyPA, **11**) was prepared according to previously published protocols.[6,7] Spectral data for **11** matches previously reported values. 1-(2-Nitrophenyl)ethanol (128 mg, 750 µmol, 1.00 equiv.) and 5-(ethylthio)-1*H*-tetrazole (ETT, 398 mg, 3.00 mmol, 4.00 equiv.) were coevaporated with anhydrous acetonitrile (2 x 3 mL). Under inert conditions, a solution containing *c*-PyPA (**11**, 75 mM in MeCN, 10.0 mL, 750 µmol, 1.00 equiv.) was added to the dried solids in a Schlenk flask and the resulting reaction mixture was stirred at room temperature for 10 min. Subsequently, *meta*-chloroperoxybenzoic acid (*m*-CPBA, 70%, 277 mg, 1.13 mmol, 1.50 equiv.) was added as a solid at 0 °C and the reaction mixture was stirred at 0 °C for 5 min. A solution of 1*H*-imidazole (2.01 g, 29.3 mmol, 39.0 equiv.) in anhydrous *N*,*N*-dimethylformamide (DMF, 7 mL) was added and the resulting reaction mixture was stirred at room temperature for 15 min. A colorless precipitate was forming upon addition. Afterward, the supernatant was poured into a solution of sodium trifluoromethanesulfonate (1.16 g, 6.75 mmol, 9.00 equiv.) in acetonitrile (30 mL, -20 °C) and after 30 min at -20 °C the resulting solids were collected by centrifugation (4,000 RPM, 5 min, 4 °C). The precipitate was washed with acetonitrile (3 x 35 mL) by centrifugation and dried *in vacuo* at room temperature to yield pure **3b** as a colorless solid (107 mg, 27% yield).

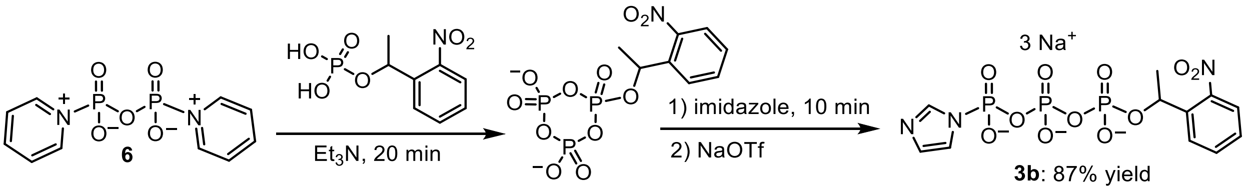

Route 2: 1-(2-nitrophenyl)ethyl phosphate and reagent **6** were prepared according to previously published protocols.[4,8] Spectral data matches previously reported values. Inside a nitrogen-filled glovebox, 1-(2-nitrophenyl)ethyl phosphate (50 mg, 0.202 mmol, 1 equiv.) was combined with tributylamine (105 µL, 0.445 mmol, 2.2 equiv.) in ca. 4 mL of acetonitrile. Separately, reagent **6** (67 mg, 0.222 mmol, 1.1 equiv.) was suspended in an equal volume of acetonitrile and stirred vigorously. The addition of the 1-(2-nitrophenyl)ethyl phosphate solution to this stirring suspension led to the gradual homogenization of the reaction mixture, with the disappearance of most insoluble material after about 5 min. The reaction mixture was filtered through a plug of Celite, and the compound was ring-opened by the addition of imidazole (275 mg, 4.04 mmol, 20 equiv.) and stirred for 10 min. A solution of sodium trifluoromethanesulfonate (280 mg, 1.62 mmol, 8 equiv.) in 4 mL of acetonitrile was added dropwise to the reaction mixture, which was subsequently transferred outside of the glovebox and into a 50 mL Falcon conical centrifuge tube. The sample was centrifuged at 2,000 RPM for 10 min. The supernatant was decanted, and an additional 15 mL of acetonitrile was added to the pellet, which was followed by brief agitation by vortex mixing. The sample was again centrifuged at 2,000 RPM for 5 min, and this washing procedure was repeated for a total of 4 cycles. The product was dried on the vacuum line, yielding pure **3b** as a colorless solid (92 mg, 87% yield).

$^{31}P\{^{1}H\}$ NMR (202 MHz, D_2O) δ -13.01 (d, *J* = 19.9 Hz, 1P), -20.50 (d, *J* = 18.8 Hz, 1P), -24.29 (d, *J* = 19.3 Hz, 1P).

^{31}P NMR (202 MHz, D_2O) δ -13.01 (dd, *J* = 19.9, 8.6 Hz, 1P), -20.50 (d, *J* = 18.8 Hz, 1P), -24.33 (t, *J* = 19.0 Hz, 1P).

^{1}H NMR (500 MHz, D_2O) δ 7.93 (dd, *J* = 8.3, 1.3 Hz, 1H), 7.86 (s, 1H), 7.77 (dd, *J* = 8.0, 1.5 Hz, 1H), 7.66 (td, *J* = 7.7, 1.3 Hz, 1H), 7.41 (ddd, *J* = 8.5, 7.4, 1.5 Hz, 1H), 7.25 (m, 1H), 6.98 (br, 1H), 5.82 (dq, *J* = 8.4, 6.4 Hz, 1H), 1.46 (d, *J* = 6.4 Hz, 3H).

$^{13}C\{^{1}H\}$ NMR (101 MHz, D_2O) δ 139.89 (d, *J* = 6.7 Hz), 134.39, 128.27, 128.20 (d, *J* = 11.9 Hz) 127.79, 124.42, 120.49 (d, *J* =6.4 Hz), 70.89 (d, *J* = 4.9 Hz), 23.59 (d, *J* = 6.2 Hz).

HRMS(−) (m/z) of $C_{11}H_{13}N_3O_{11}P_3^-$ $[M]^-$: 455.9768 (calc'd 455.9768)

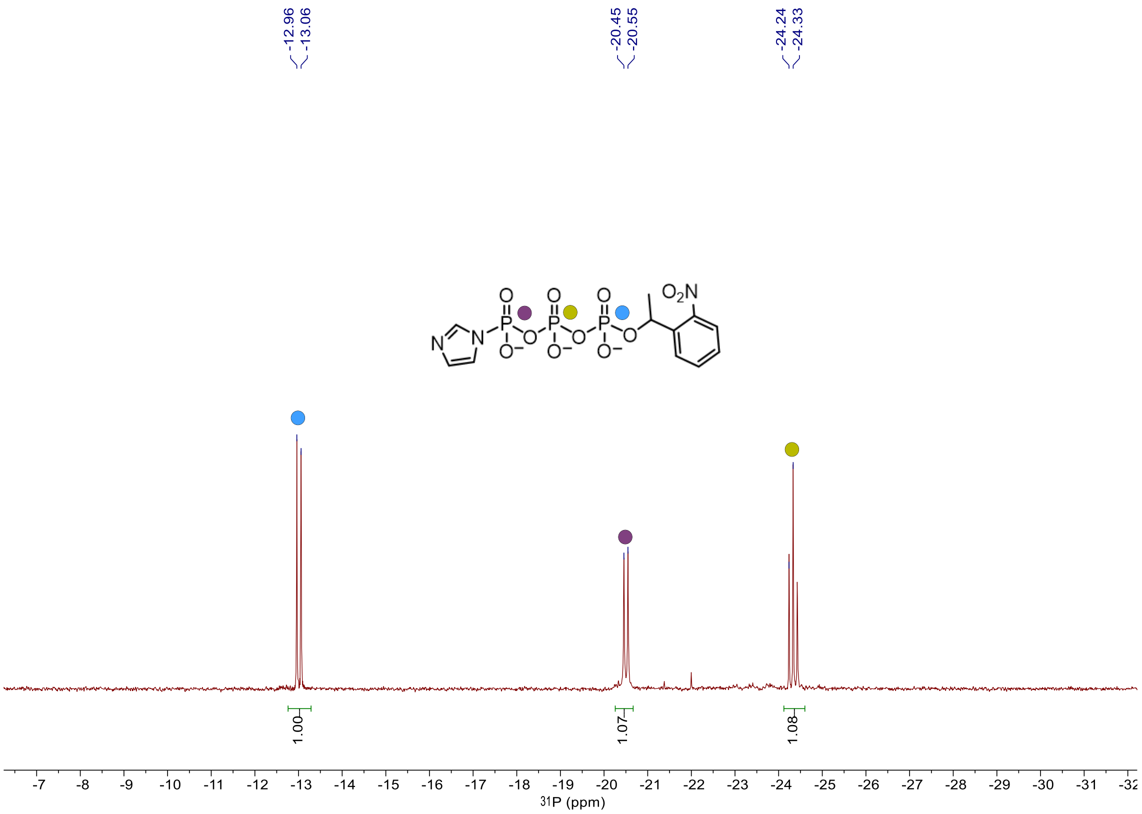

Figure S18: $^{31}P\{^{1}H\}$ NMR spectrum of **3b.**

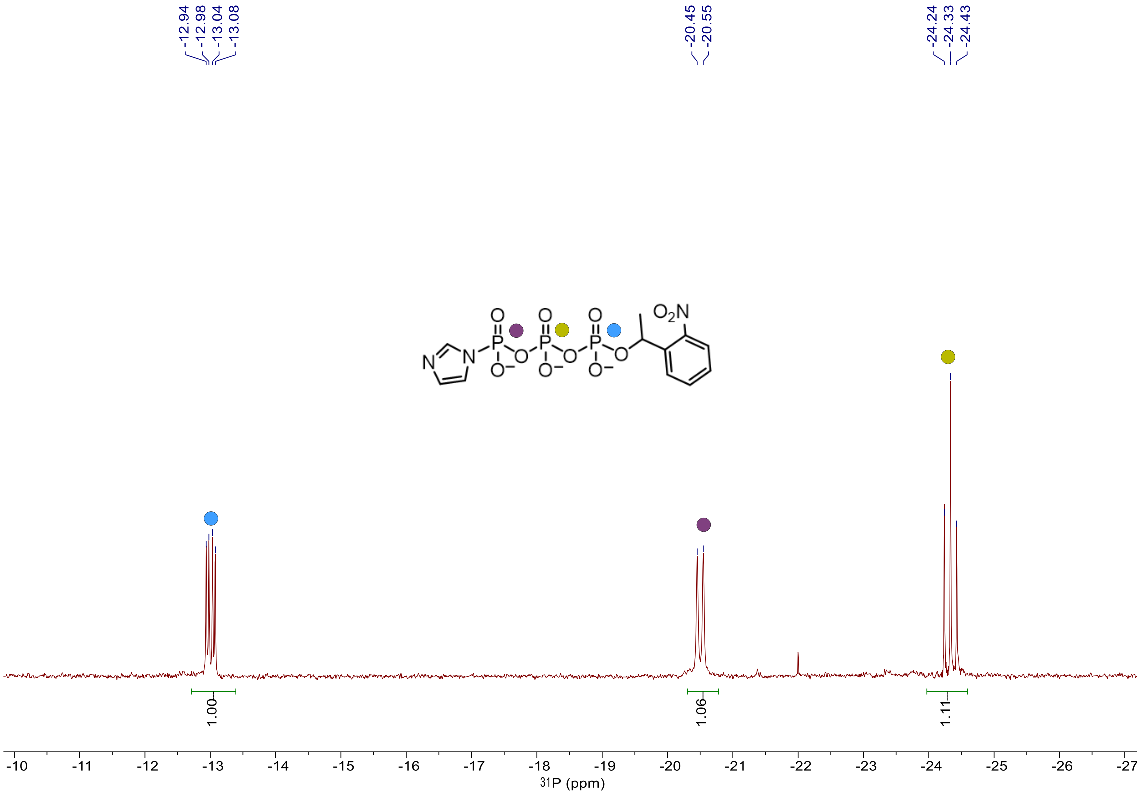

Figure S19: ^{31}P NMR spectrum of **3b.**

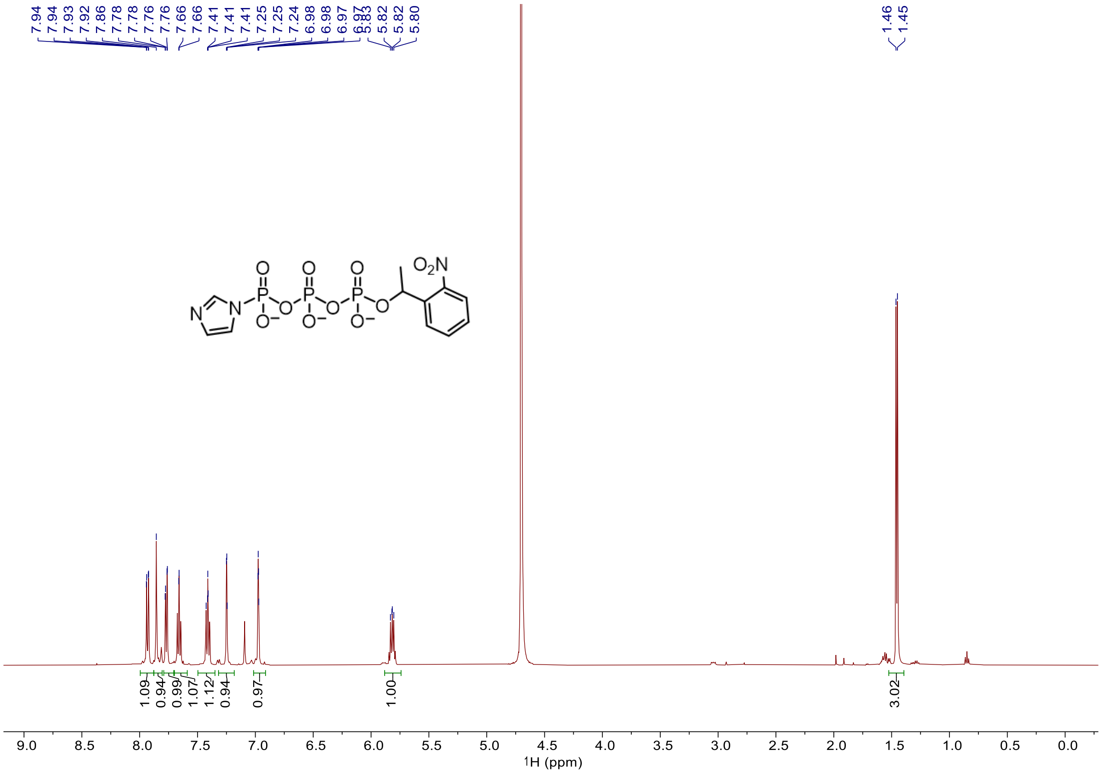

Figure S20: ^{1}H NMR spectrum of **3b.**

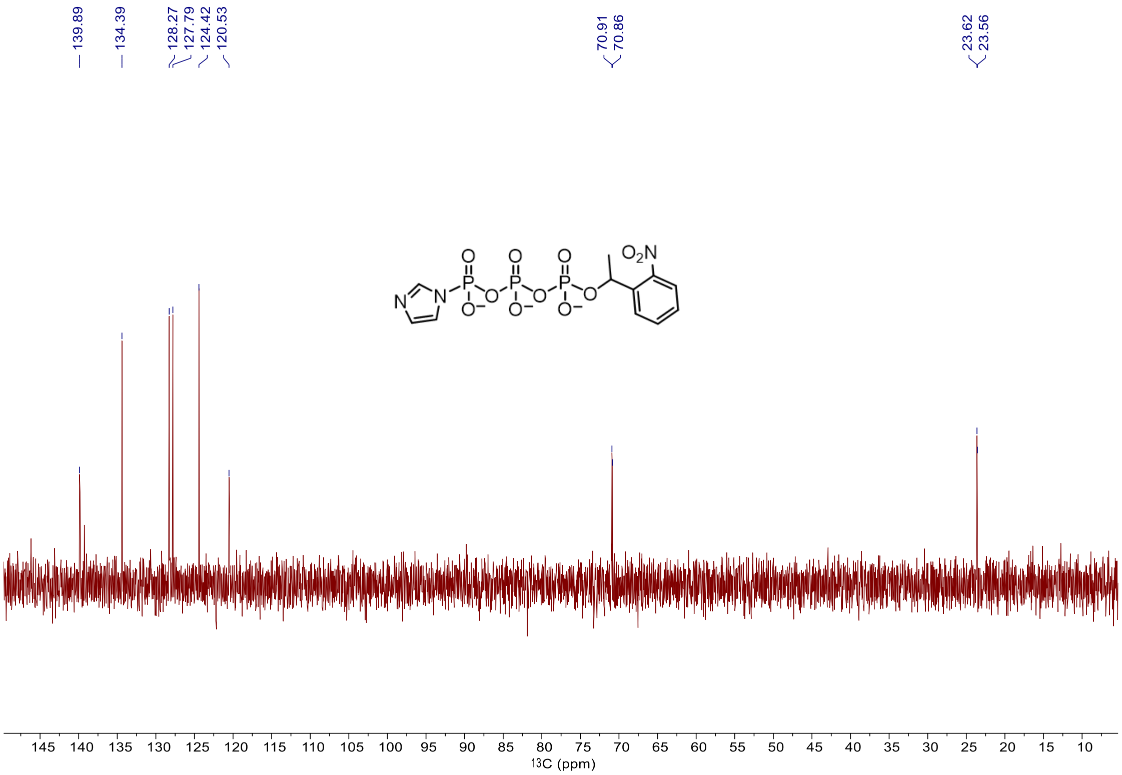

Figure S21: ^{13}C NMR spectrum of **3b.**

2.3 Synthesis of Tetraphosphorylation Reagents 4a/4b

2.3.1 Synthesis of 4a

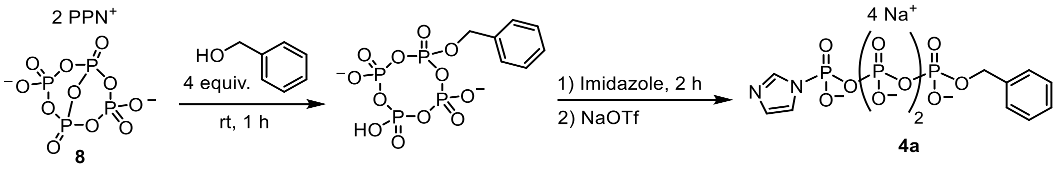

Tetraphosphorylation reagent **8** was synthesized according to literature procedure.[9] Under an inert atmosphere, reagent **8** (1.40 g, 1.02 mmol, 1 equiv.) and benzyl alcohol (440 mg, 4.06 mmol, 4 equiv.) were combined in a scintillation vial with 5 mL of acetonitrile. The mixture was allowed to stir at room temperature for 1 h, cleanly forming benzyl tetrametaphosphate. Imidazole (3.46 g, 50.82 mmol, 50 equiv.) was added to the reaction mixture, and this ring-opening reaction was complete after 2 h. A solution of sodium trifluoromethanesulfonate (1.40 g, 8.13 mmol, 8 equiv.) in 15 mL of acetonitrile was combined with the reaction mixture, leading to the precipitation of the desired product. The reaction mixture was transferred outside of the glovebox and into a 50 mL Falcon conical centrifuge tube. The sample was centrifuged at 2,000 RPM for 10 min. The supernatant was decanted, and an additional 25 mL of acetonitrile was added to the pellet, which was followed by brief agitation by vortex mixing. The sample was again centrifuged at 2,000 RPM for 5 min, and this washing procedure was repeated for a total of 5 cycles. The product was dried on the vacuum line, yielding pure **4a** as a fine colorless powder (509 mg, 90% yield).

$^{31}P\{^1H\}$ NMR (162 MHz, D_2O) δ -11.71 (d, *J* = 19.3 Hz, 1P), -20.67 (d, *J* = 18.4 Hz, 1P), -23.67 (t, *J* = 18.4 Hz, 1P), -24.15 (t, *J* = 17.7 Hz, 1P).

^{31}P NMR (162 MHz, D_2O) δ -11.73 (dt, *J* = 19.1, 6.9 Hz, 1P), -20.69 (d, *J* = 18.9 Hz, 1P), -23.82 (t, *J* = 18.2 Hz, 1P), -24.26 (t, *J* = 17.7 Hz, 1P).

^1H NMR (400 MHz, D_2O) δ 8.00 (br, 1H), 7.47–7.24 (m, 5H), 7.16 (br, 1H), 6.99 (br, 1H), 4.90 (d, *J* = 6.8 Hz, 2H).

$^{13}C\{^1H\}$ NMR (101 MHz, D_2O) δ 139.51 (d, *J* = 7.2 Hz), 137.36 (d, *J* = 8.5 Hz), 128.63, 128.12, 127.86, 127.09 (d, *J* = 12.3 Hz), 120.73 (d, *J* = 6.1 Hz), 68.04 (d, *J* = 5.7 Hz).

HRMS(−) (m/z) of $C_{10}H_{13}N_2O_{12}P_4^-$ $[M]^-$: 476.9425 (calc'd 476.9424)

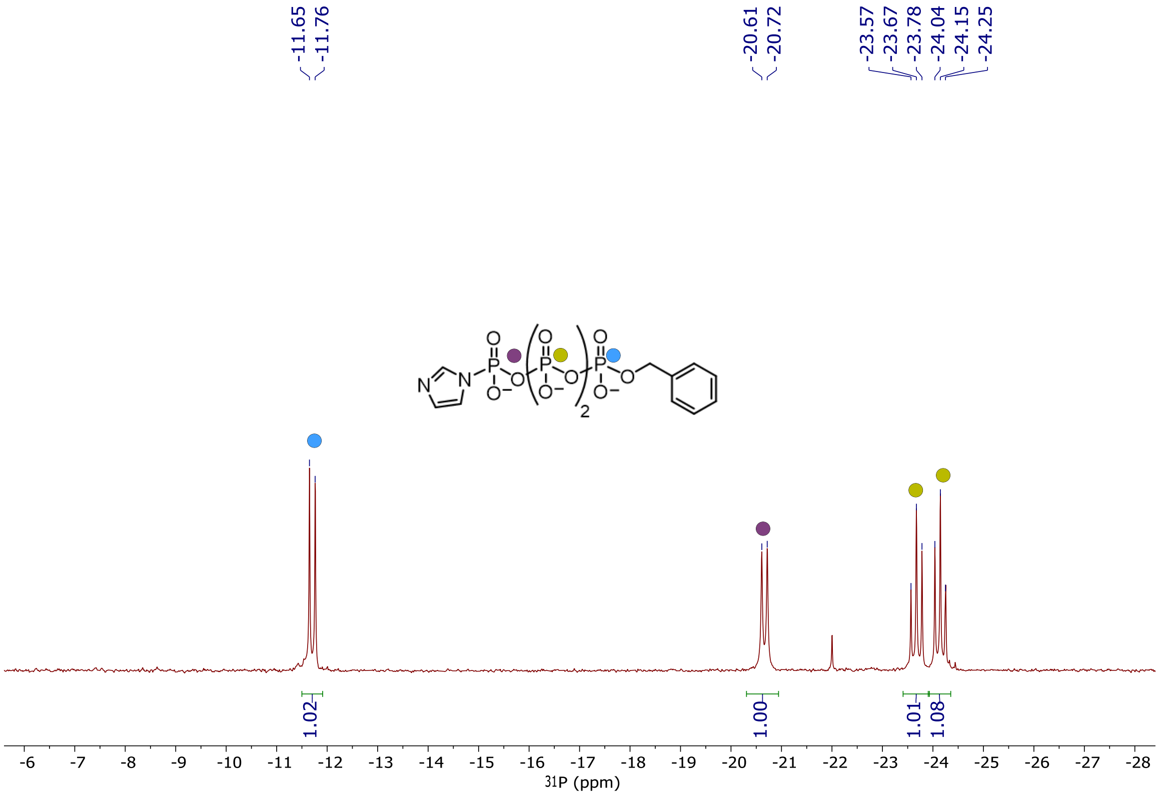

Figure S22: $^{31}P\{^1H\}$ NMR spectrum of **4a.**

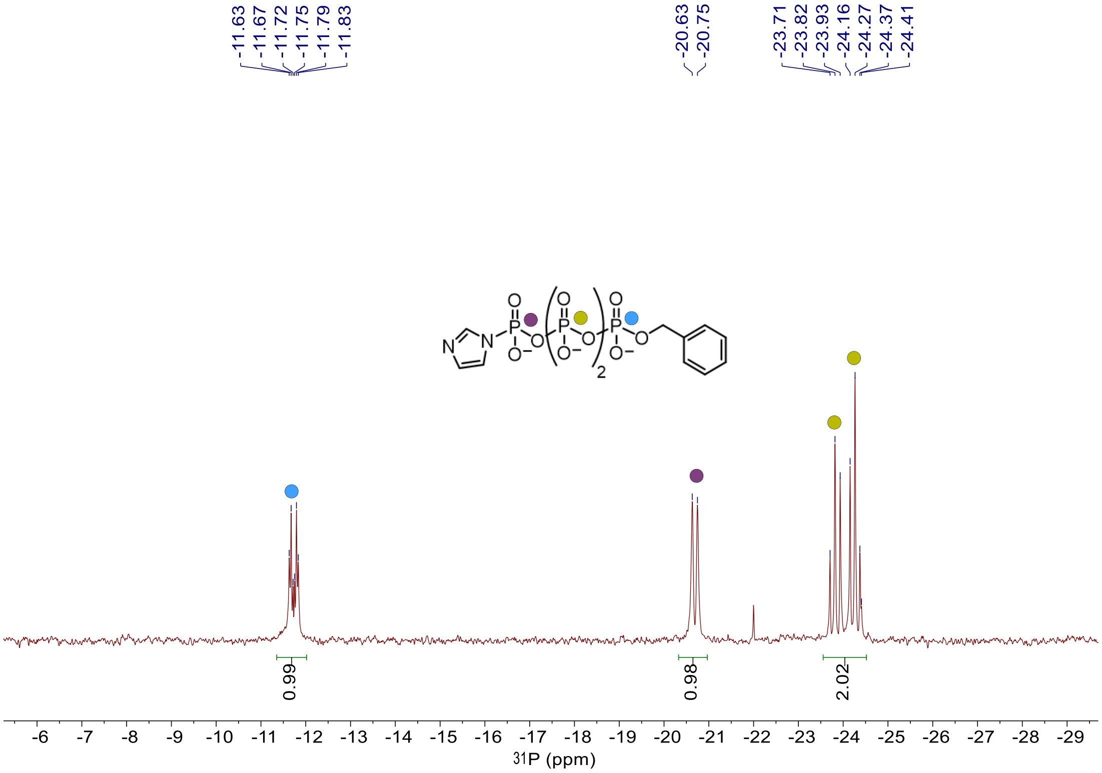

Figure S23: ^{31}P NMR spectrum of **4a.**

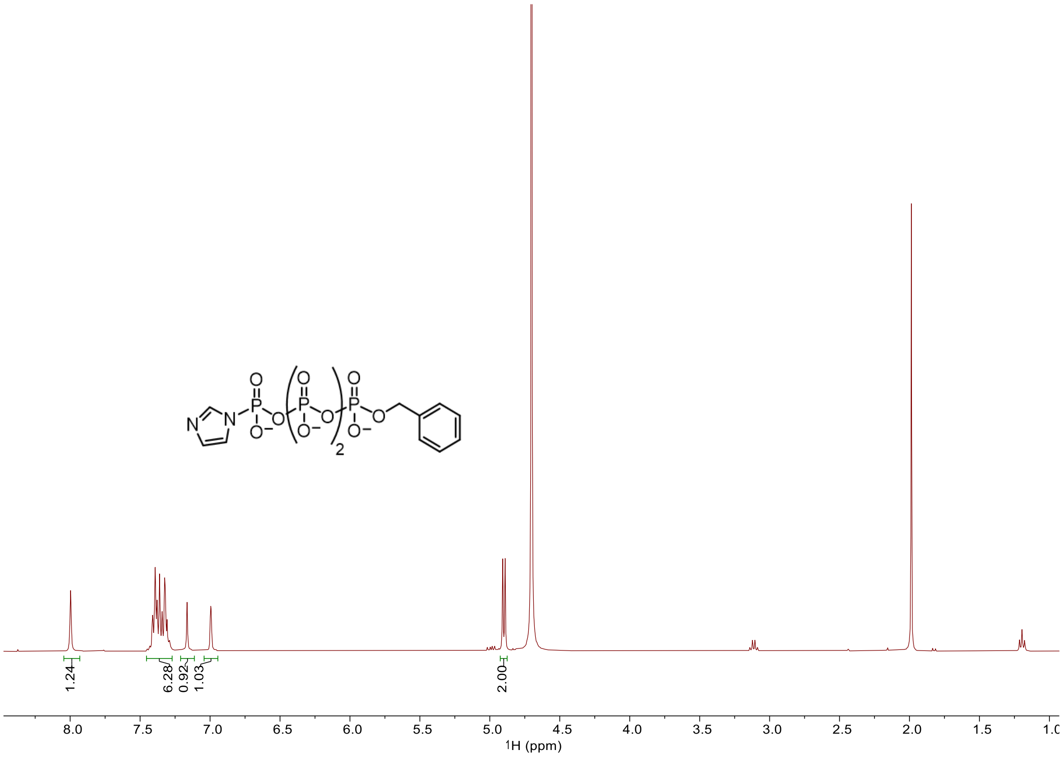

Figure S24: ^{1}H NMR spectrum of **4a.**

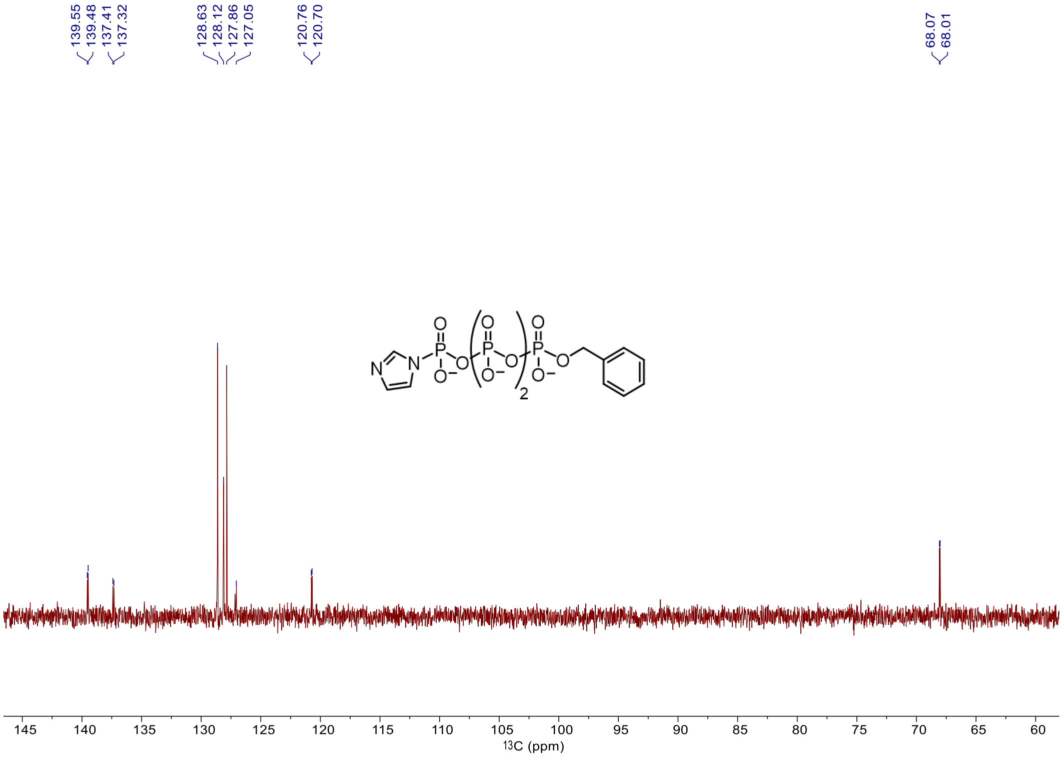

Figure S25: $^{13}C\{^{1}H\}$ NMR spectrum of **4a.**

2.3.2 Synthesis of 4b

The tetraphosphorylation of 1-(2-nitrophenyl)ethanol with **8** does not proceed with sufficient conversion due to the steric bulk of the substrate. In order to synthesize **4b**, it is necessary to convert 1-(2-nitrophenyl)ethanol to more nucleophilic alkoxy salts.

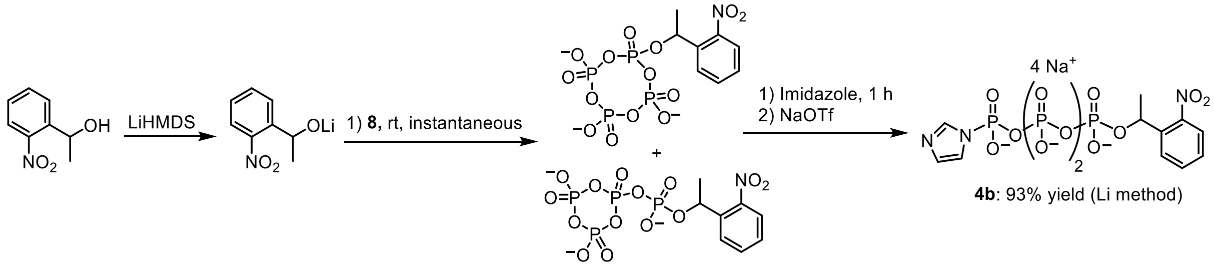

Synthesis of 4b *via* lithium 1-(2-nitrophenyl)ethanoxide: Under an inert atmosphere, 1-(2-nitrophenyl)ethanol (167 mg, 1 mmol, 1 equiv.) was dissolved in ca. 5 mL of THF. Lithium bis(trimethylsilyl)amide (334 mg, 2 mmol, 2 equiv.) was added to the solution, causing the color of the reaction mixture to change from a pale yellow to a deep yellow-orange. The solution was stirred for 20 min, and solvent was removed under reduced pressure. The residue was triturated with 3×6 mL of hexanes, and dried again *in vacuo*. The lithium salt of 1-(2-nitrophenyl)ethanoxide was obtained as a “beach sand” colored pale-yellow powder. ^{1}H NMR spectroscopy was used to quantify the amount of residual THF molecules.

In a nitrogen-filled glovebox, reagent **8** (689 mg, 0.50 mmol, 1 equiv.) was dissolved in ca. 5 mL of acetonitrile and stirred vigorously. Lithium 1-(2-nitrophenyl)ethanoxide (1 equiv.) was added to the reaction mixture, resulting in a pink-colored solution. Consumption of starting material was monitored by collecting ^{31}P NMR spectroscopy of reaction aliquots. In the instances where reactant **8** was still present, additional lithium 1-(2-nitrophenyl)ethanoxide was introduced (by increments of 0.1 equivalents) until all of **8** was consumed.* Afterwards, imidazole (1.7 g, 25 mmol, 50 equiv.) was added to the reaction mixture, causing the pink color to disappear. The ring-opening reaction was complete after 1 h of stirring at room temperature. A solution of sodium trifluoromethanesulfonate (0.688 g, 4.0 mmol, 8 equiv.) in 5 mL of acetonitrile was combined with the reaction mixture, leading to the precipitation of the desired product. The reaction mixture was transferred outside of the glovebox and into a 50 mL Falcon conical centrifuge tube. The sample was centrifuged at 2,000 RPM for 10 min. The supernatant was decanted, and an additional 15 mL of acetonitrile was added to the pellet, which was followed by brief agitation by vortex mixing. The sample was again centrifuged at 2,000 RPM for 5 min, and this washing procedure was repeated for a total of 4 cycles. The product was dried on the vacuum line, yielding pure **4b** as an off-white powder (291 mg, 93% yield).

NOTE: the stoichiometry must be carefully controlled as an excess of lithium 1-(2-nitrophenyl)ethanoxide will result in the undesired di-substitution of* ***8.*

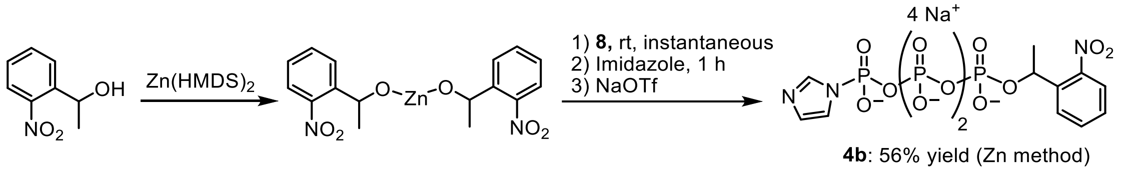

Synthesis of 4b *via* zinc bis[1-(2-nitrophenyl)ethanoxide]: Reagent **4b** can also be prepared following the identical procedure but instead from the zinc bis[1-(2-nitrophenyl)ethanoxide], the preparation of which is described in SI 2.4.3. The reaction between **8** (138 mg, 0.1 mmol, 2 equiv.), and zinc bis[1-(2-nitrophenyl)ethanoxide] (20 mg, 0.05 mmol, 1 equiv.), and imidazole (680 mg, 10.0 mmol, 200 equiv.) followed by the standard counterion exchange procedure with sodium trifluoromethanesulfonate (172 mg, 1.0 mmol, 20 equiv.) yielded the product as a colorless solid (35 mg, 56% yield). Although the yield is lower with this procedure, using the zinc salt of 1-(2-nitrophenyl)ethanoxide is more convenient compared to the lithium salt as it is less reactive and does not so readily ring-open the substituted tetrametaphosphate intermediate, such that the reaction stoichiometry does not have to be as carefully controlled.

^{31}P{^{1}H} NMR (162 MHz, D_2O) δ -12.77 (d, *J* = 18.3 Hz, 1P), -20.47 (d, *J* = 17.4 Hz, 1P), -23.87 (t, *J* = 18.1 Hz, 1P), -24.23 (t, *J* = 17.4 Hz, 1P).

^{31}P NMR (162 MHz, D_2O) δ -12.77 (dd, *J* = 18.2, 8.2 Hz, 1P), -20.47 (d, *J* = 17.3 Hz, 1P), -23.87 (t, *J* = 18.0 Hz, 1P), -24.23 (t, *J* = 17.4 Hz, 1P).

^{1}H NMR (400 MHz, D_2O) δ 8.01–7.88 (m, 2H), 7.84 (dd, *J* = 8.0, 1.5 Hz, 1H), 7.69 (td, *J* = 7.7, 1.3 Hz, 1H), 7.43 (ddd, *J* = 8.6, 7.4, 1.5 Hz, 1H), 7.33 (q, *J* = 1.6 Hz, 1H), 7.02 (br, 1H), 5.88 (dq, *J* = 8.2, 6.4 Hz, 1H), 1.53 (d, *J* = 6.4 Hz, 3H).

^{13}C{^{1}H} NMR (101 MHz, D_2O) δ 139.80, 134.44, 128.26, 127.92, 124.39, 120.62, 70.94 (d, *J* = 4.6 Hz), 23.57 (d, *J* = 6.2 Hz).

HRMS(−) (m/z) of $C_{11}H_{14}N_3O_{14}P_4^-$ $[M]^-$: 535.9441 (calc'd 535.9432)

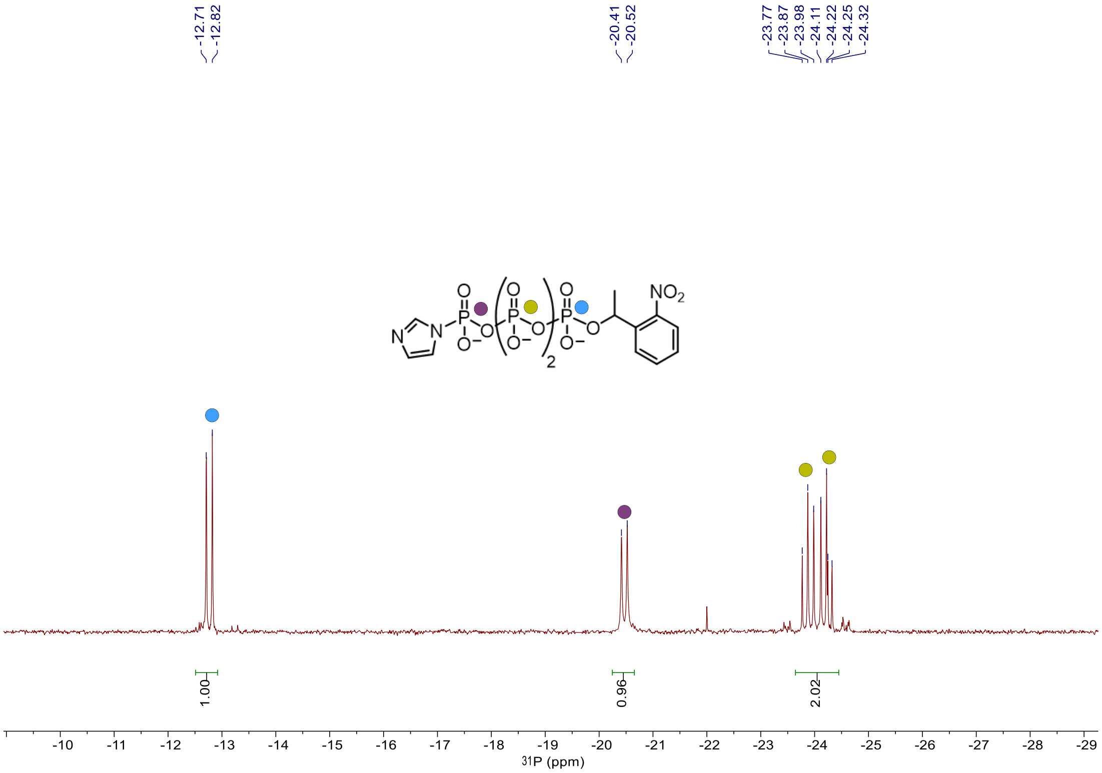

Figure S26: $^{31}P\{^1H\}$ NMR spectrum of **4b.**

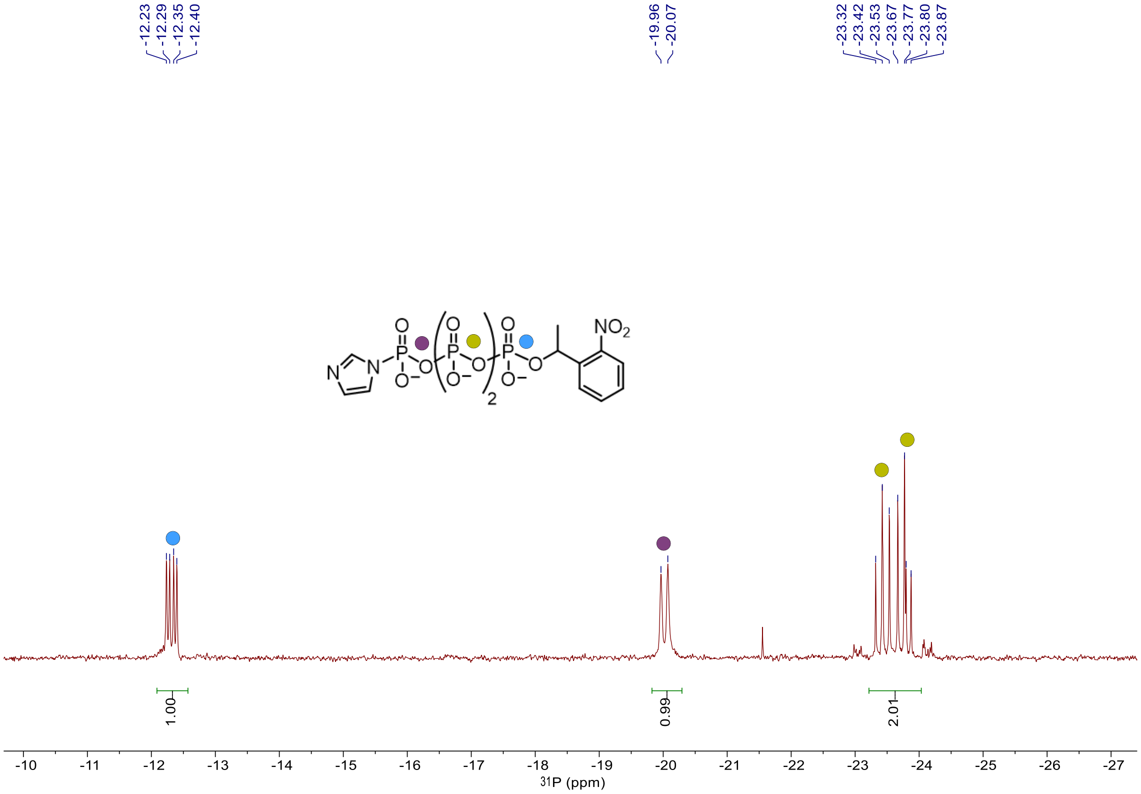

Figure S27: ^{31}P NMR spectrum of **4b.**

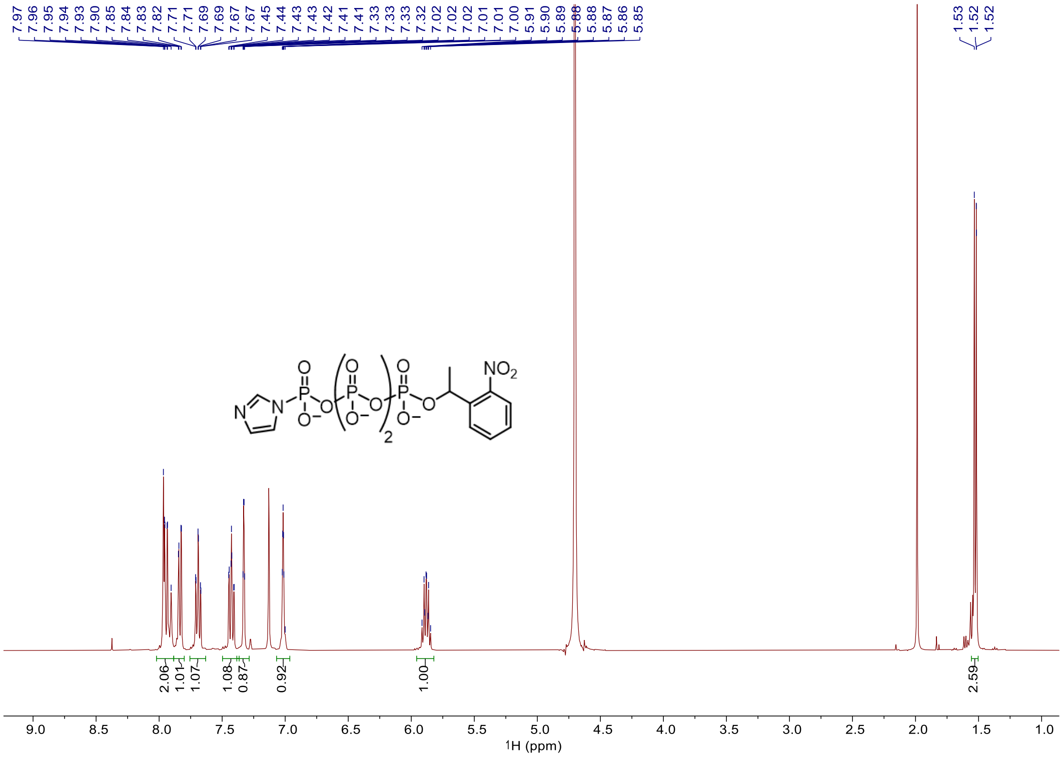

Figure S28: ^{1}H NMR spectrum of **4b.**

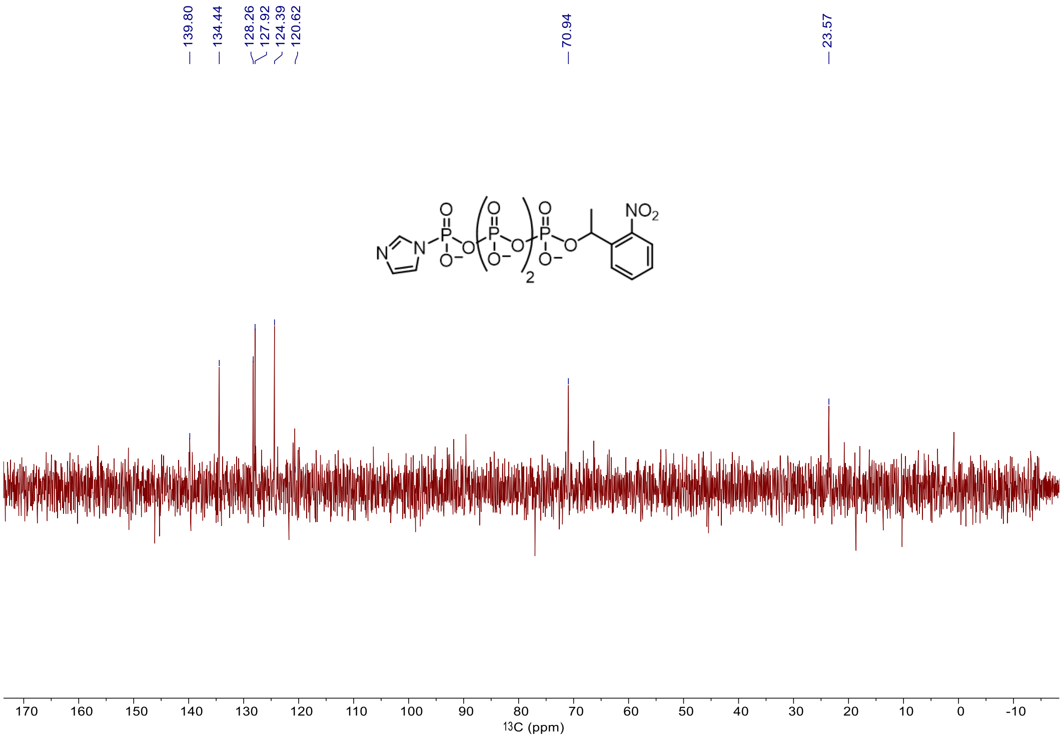

Figure S29: ^{13}C{^{1}H} NMR spectrum of **4b.**

2.4 Synthesis of Pentaphosphorylation Reagents 5a/5b

2.4.1 Synthesis of Precursor 9

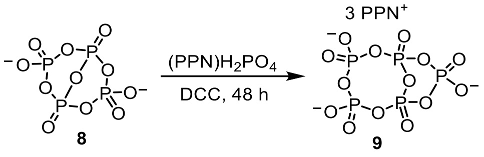

To synthesize compound **5b**, it is necessary that precursor **9** be prepared as the tris PPN^+ salt. Our group has previously published the synthesis of the mixed $(PPN)_2$(TBA) salt of **9**,[10] but the TBA^+ counterion is incompatible with zinc bis[1-(2-nitrophenyl)ethanoxide].

Synthesis of $(PPN)H_2PO_4$: this salt was reported in Patent US5045632A.[11] However, despite numerous attempts, the results could not be reproduced by our hands following their procedure as written. Modifications were made to this protocol to obtain $(PPN)H_2PO_4$. In the fumehood, bis(triphenylphosphine)iminium chloride (5.74 g, 10 mmol, 4 equiv.) and potassium hydroxide (0.56 g, 10 mmol, 4 equiv.) were dissolved separately in a minimal amount of methanol (6 mL and 2 mL, respectively). Both solutions were combined, and the mixture was allowed to stir for 5 min. The potassium chloride precipitate was removed by filtration, and a solution of phosphoric acid (0.25 g, 2.5 mmol, 1 equiv.) in 1 mL of methanol was added to the filtrate. This mixture was allowed to stir for 15 min, and precipitates were again removed by filtration. Volatiles were removed under reduced pressure in a rotary evaporator at 70 °C (all residual methanol must be removed or the product will dissolve in acetone in the next step). The dried solids were suspended in ca. 40 mL of acetone and centrifuged at 1,000 RPM for 5 min. The supernatant was decanted, and the pellet was similarly washed again with 3x40 mL acetone. The product was dried under vacuum overnight, yielding a colorless powder (1.1 g, 69% yield).

^{31}P NMR (162 MHz, CD_3CN) δ 20.78 (br, 2P), 2.29 (s, 1P)

Synthesis of 9, $(PPN)_3P_5O_{14}$: In a nitrogen-filled glovebox, compound **8** (1.38 g, 1 mmol, 1 equiv.), $(PPN)H_2PO_4$ (0.67 g, 1.05 mmol, 1.05 equiv.), and N,N'-dicyclohexylcarbodiimide (DCC, 0.42 g, 2.00 mmol, 2 equiv.) were dissolved in 10 mL of dry acetonitrile. The white suspension was allowed to stir vigorously at room temperature for 48 h. Then, the mixture was passed through a Celite plug to remove insoluble dicyclohexylurea (DCU), and concentrated under reduced pressure to ~1 mL. Diethyl ether (ca. 15 mL) was added to precipitate the product out of solution as a waxy goo. The supernatant was decanted, and the residue was dried under vacuum. The solids were triturated with 3x5 mL tetrahydrofuran and 3x5 mL diethyl ether, and after drying under vacuum, the product was obtained as a free-flowing colorless solid (1.85 g, 94% yield).

$^{31}P\{^1H\}$ NMR (162 MHz, None) δ 21.74 (d, J = 5.3 Hz, 6P), -24.10 (td, J = 24.5, 5.2 Hz, 1P), -24.99 (m, 2P), -35.29 (m, 2P).

^1H NMR (400 MHz, CD_3CN) δ 7.69 (td, J = 7.4, 1.6 Hz, 18H), 7.66 – 7.56 (m, 36H), 7.51 (td, J = 7.8, 3.1 Hz, 36H).

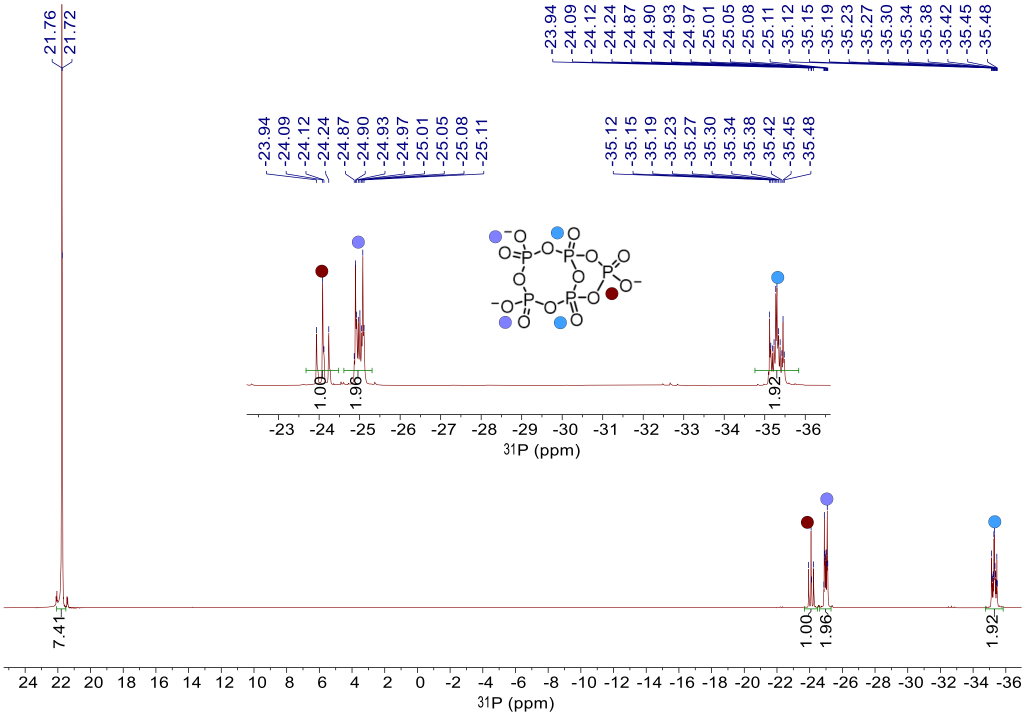

Figure S30: $^{31}P\{^{1}H\}$ NMR spectrum of **9**.

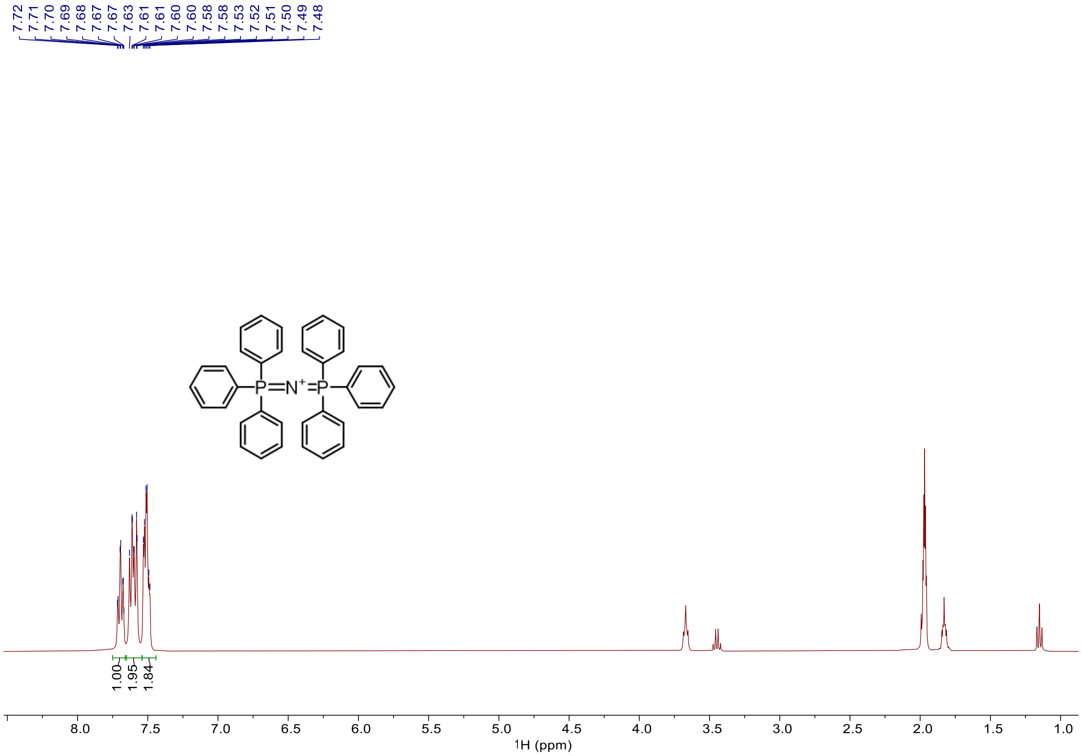

Figure S31: ^{1}H NMR spectrum of **9**.

2.4.2 Synthesis of 5a

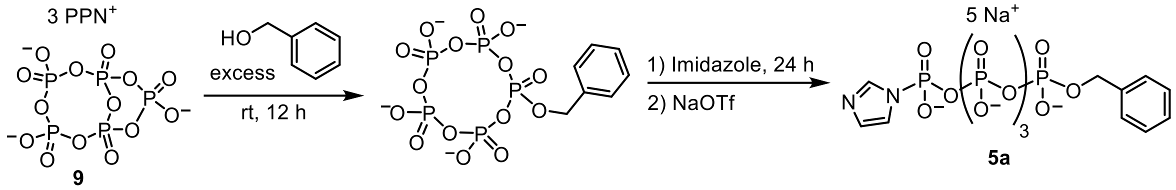

Under an inert atmosphere, reagent **9** (0.500 g, 0.251 mmol, 1 equiv.) was combined with excess benzyl alcohol (1.35 g, 12.51 mmol, 50 equiv.) in ca. 5 mL of acetonitrile and stirred at room temperature overnight. Imidazole (0.853 g, 12.53 mmol, 50 equiv.) was added, and the reaction mixture was stirred for another 24 h. A solution of sodium trifluoromethanesulfonate (0.432 g, 2.51 mmol, 10 equiv.) in 10 mL of acetonitrile was combined with the reaction mixture, leading to the precipitation of the desired product. The reaction mixture was transferred to a 50 mL Falcon conical centrifuge tube. The sample was centrifuged at 2,000 RPM for 10 min. The supernatant was decanted, and an additional 20 mL of acetonitrile was added to the pellet, which was followed by brief agitation by vortex mixing. The sample was again centrifuged at 2,000 RPM for 5 min, and this washing procedure was repeated for a total of 5 cycles. The product was dried on the vacuum line, yielding pure **5a** as a fine colorless powder (157 mg, 93% yield).

$^{31}P\{^{1}H\}$ NMR (162 MHz, D_2O) δ -11.53 (d, *J* = 17.5 Hz, 1P), -20.53 (d, *J* = 17.8 Hz, 1P), -23.24 (t, *J* = 15.8 Hz, 1P), -23.46 (t, *J* = 15.6 Hz, 1P), -23.95 (dd, *J* = 18.0, 14.7 Hz, 1P).

^{31}P NMR (162 MHz, D_2O) δ -11.53 (dt, *J* = 17.5, 6.9 Hz, 1P), -20.53 (d, *J* = 17.8 Hz, 1P), -23.24 (t, *J* = 15.8 Hz, 1P), -23.46 (t, *J* = 15.7 Hz, 1P), -23.95 (dd, *J* = 17.8, 14.7 Hz, 1P).

^{1}H NMR (400 MHz, D_2O) δ 8.03–7.91 (m, 1H), 7.50–7.23 (m, 5H), 7.15 (br, 1H), 7.02 (br, 1H), 4.95 (d, *J* = 7.0 Hz, 2H).

$^{13}C\{^{1}H\}$ NMR (101 MHz, D_2O) δ 139.68 (d, *J* = 7.0 Hz), 137.40 (d, *J* = 8.6 Hz), 128.64, 128.12, 127.88, 127.54 (m) ,120.72 (m), 68.10 (d, *J* = 5.5 Hz).

HRMS(−) (m/z) of $C_{10}H_{14}N_2O_{15}P_5^{-}$ $[M]^{-}$: 556.9098 (calc'd 556.9088)

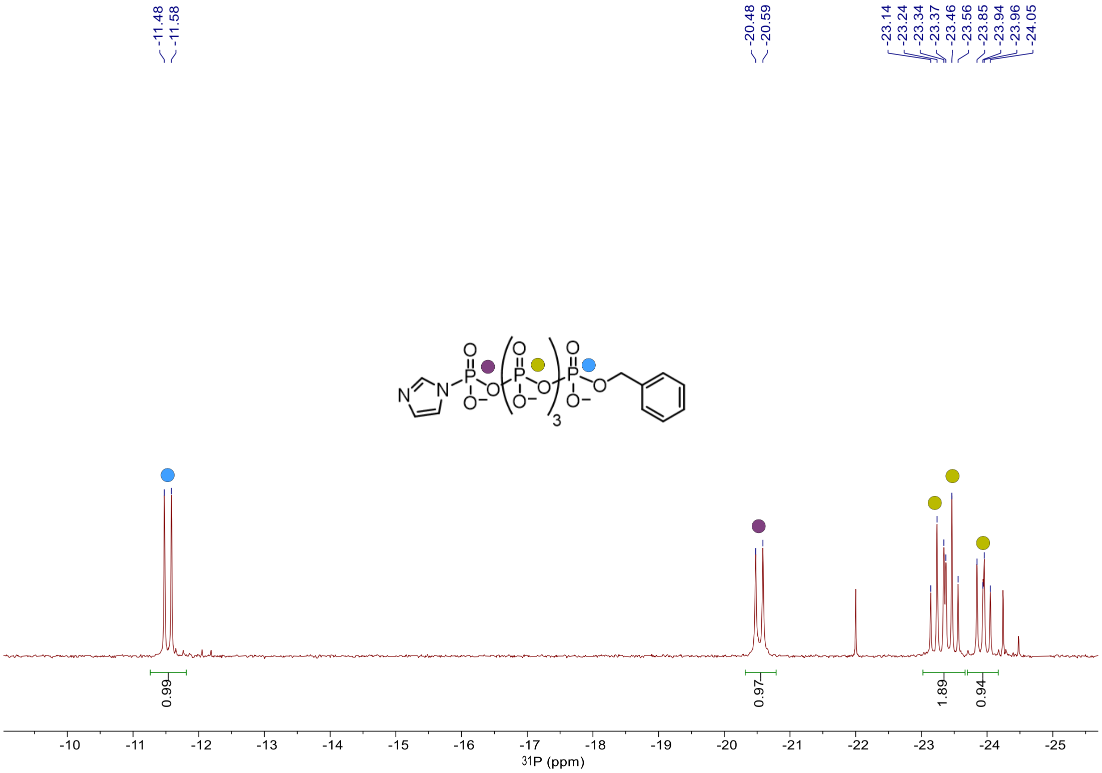

Figure S32: $^{31}P\{^{1}H\}$ NMR spectrum of **5a**.

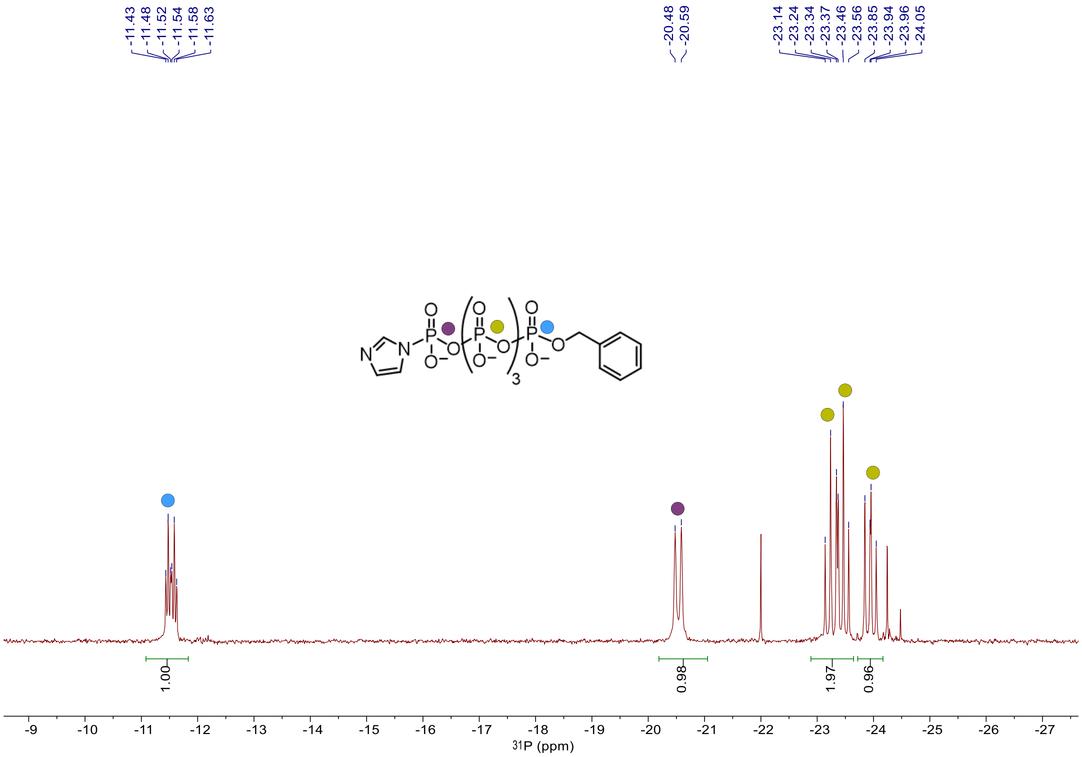

Figure S33: ^{31}P NMR spectrum of **5a**.

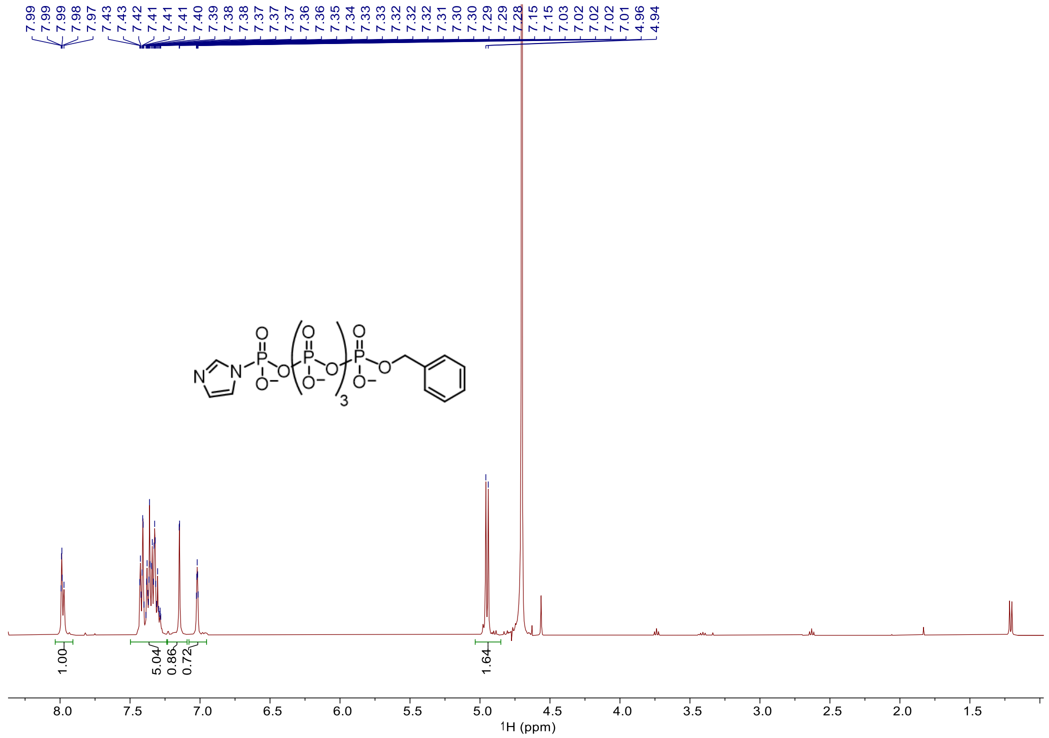

Figure S34: ^{1}H NMR spectrum of **5a**.

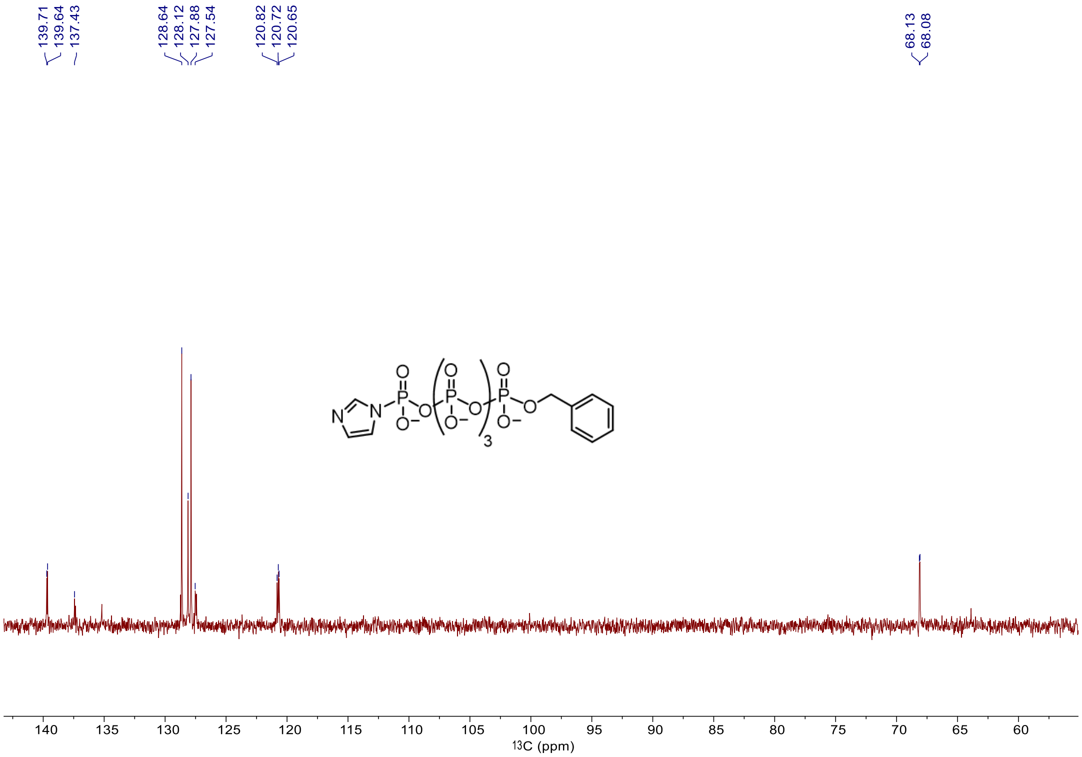

Figure S35: ^{13}C{^{1}H} NMR spectrum of **5a**.

2.4.3 Synthesis of 5b

The pentaphosphorylation of 1-(2-nitrophenyl)ethanol with **9** does not proceed with sufficient conversion (even with excess base and heating) due to the steric bulk of the substrate. To synthesize **5b**, it is necessary to convert 1-(2-nitrophenyl)ethanol to the more nucleophilic zinc salt. Unlike with reagent **8**, reagent **9** does not react cleanly with lithium 1-(2-nitrophenyl)ethanoxide, and so zinc bis[1-(2-nitrophenyl)ethanoxide] must be used.

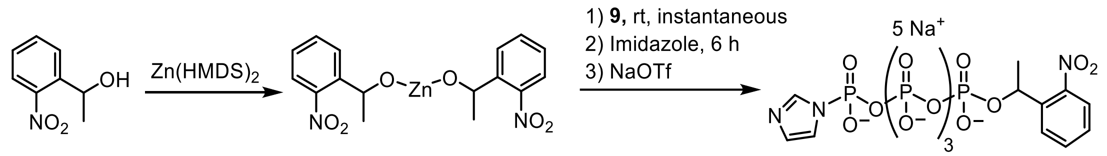

5b

Synthesis of 5b *via* zinc bis[1-(2-nitrophenyl)ethanoxide]:
Zinc bis[bis(trimethylsilyl)amide] was prepared from $ZnCl_2$ and lithium bis(trimethylsilyl)amide according to literature procedure.[12] Inside a nitrogen-filled glovebox, zinc bis[bis(trimethylsilyl)amide] (386 mg, 1 mmol, 1 equiv.) was dissolved in 8 mL of toluene. 1-(2-nitrophenyl)ethanol (334 mg, 2 mmol, 2 equiv.) was added, immediately forming the product as a white precipitate. The suspension was vigorously stirred for 1 h. The solvent was decanted, and the residue was triturated with 3×10 mL of toluene. Pentane (ca. 2 mL) was added to the sample and all solvent was evaporated under reduced pressure. This was repeated once more, and zinc bis[1-(2-nitrophenyl)ethanoxide] was obtained as a pale-yellow solid (341 mg, 86% yield).

In a nitrogen-filled glovebox, **9** (1.00 g, 0.50 mmol, 1 equiv.) was dissolved in ca. 10 mL of acetonitrile. With stirring, zinc bis[1-(2-nitrophenyl)ethanoxide] (0.200 g, 0.50 mmol, 1 equiv.) was introduced.* Afterwards, imidazole (3.4 g, 50 mmol, 100 equiv.) was added to the reaction mixture, which was allowed to stir for an additional 6 h at room temperature. A solution of sodium trifluoromethanesulfonate (1.03 g, 6.0 mmol, 12 equiv.) in 10 mL of acetonitrile was combined with the reaction mixture, leading to the precipitation of the desired product. The reaction mixture was transferred outside of the glovebox and into a 50 mL Falcon conical centrifuge tube. The sample was centrifuged at 2,000 RPM for 10 min. The supernatant was decanted, and an additional 30 mL of acetonitrile was added to the pellet, which was followed by brief agitation by vortex mixing. The sample was again centrifuged at 2,000 RPM for 5 min, and this washing procedure was repeated for a total of 4 cycles. The product was dried on the vacuum line, yielding **5b** as an off-white powder (235 mg, 65% yield).

NOTE: while 1 molar equivalent of zinc bis[1-(2-nitrophenyl)ethanoxide] is indeed two molar equivalents of 1-(2-nitrophenyl)ethanoxide, reactant* **9 *is not fully consumed if one only uses half an equivalent of zinc bis[1-(2-nitrophenyl)ethanoxide.*

$^{31}P\{^1H\}$ NMR (162 MHz, D_2O) δ -11.23 (d, *J* = 13.8 Hz, 1P), -19.48 (d, *J* = 14.8 Hz, 1P), -20.48 (t, *J* = 10.7 Hz, 1P), -20.67 (t, *J* = 10.9 Hz, 1P), -21.15 (dd, *J* = 14.8, 10.8 Hz, 1P).

^{31}P NMR (162 MHz, D_2O) δ -11.23 (dd, *J* = 14.0, 8.7 Hz, 1P), -19.48 (d, *J* = 14.8 Hz, 1P), -20.48 (t, *J* = 10.5 Hz, 1P), -20.67 (t, *J* = 10.9 Hz, 1P), -21.15 (dd, *J* = 14.6, 10.6 Hz, 1P).

^1H NMR (400 MHz, D_2O) δ 8.03 (m, 1H), 7.94 (m, 1H), 7.84 (dd, *J* = 8.0, 1.5 Hz, 1H), 7.70 (td, *J* = 7.7, 1.4 Hz, 1H), 7.43 (ddt, *J* = 10.1, 7.8, 2.1 Hz, 1H), 7.34 (br, 1H), 7.05 (br, 1H), 5.90 (dt, *J* = 7.9, 6.2 Hz, 1H), 1.56 (d, *J* = 6.3 Hz, 3H).

$^{13}C\{^1H\}$ NMR (101 MHz, D_2O) δ 134.62, 128.44, 128.09, 124.51, 71.13 (d, *J* = 4.8 Hz) , 23.77 (d, *J* = 6.3 Hz).

HRMS(−) (m/z) of $C_{11}H_{15}N_3O_{17}P_5^-$ $[M]^-$: 615.9101 (calc'd 615.9095)

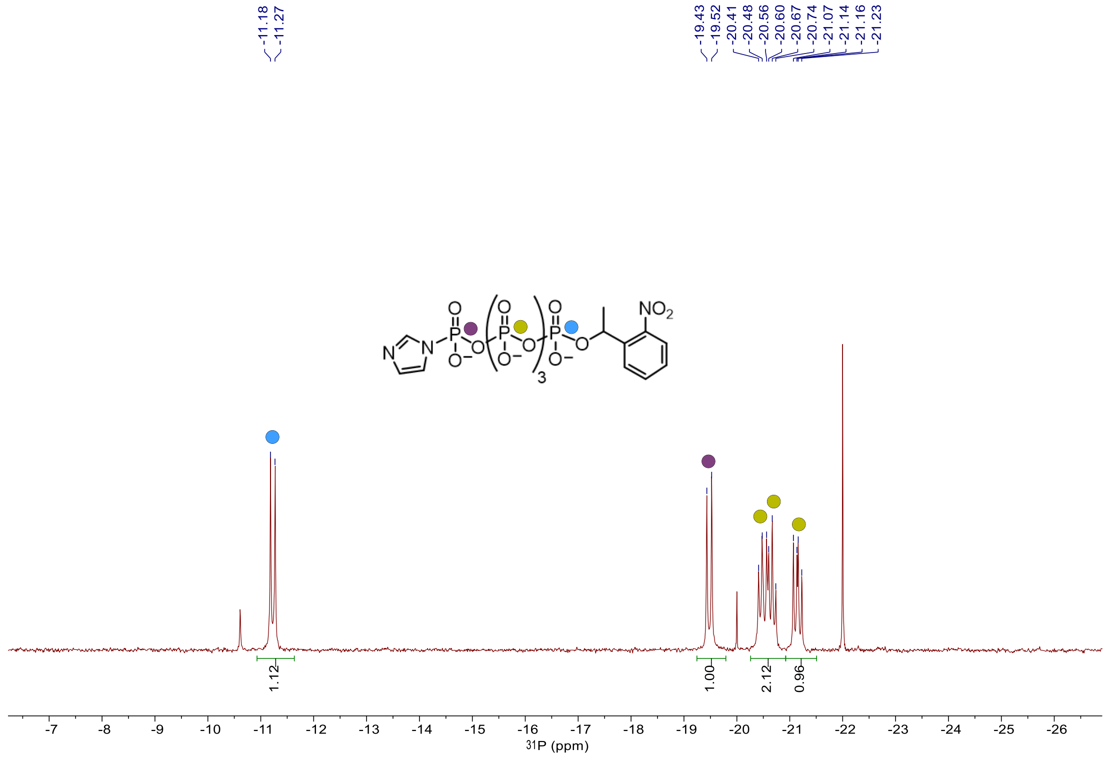

Figure S36: $^{31}P\{^{1}H\}$ NMR spectrum of **5b**.

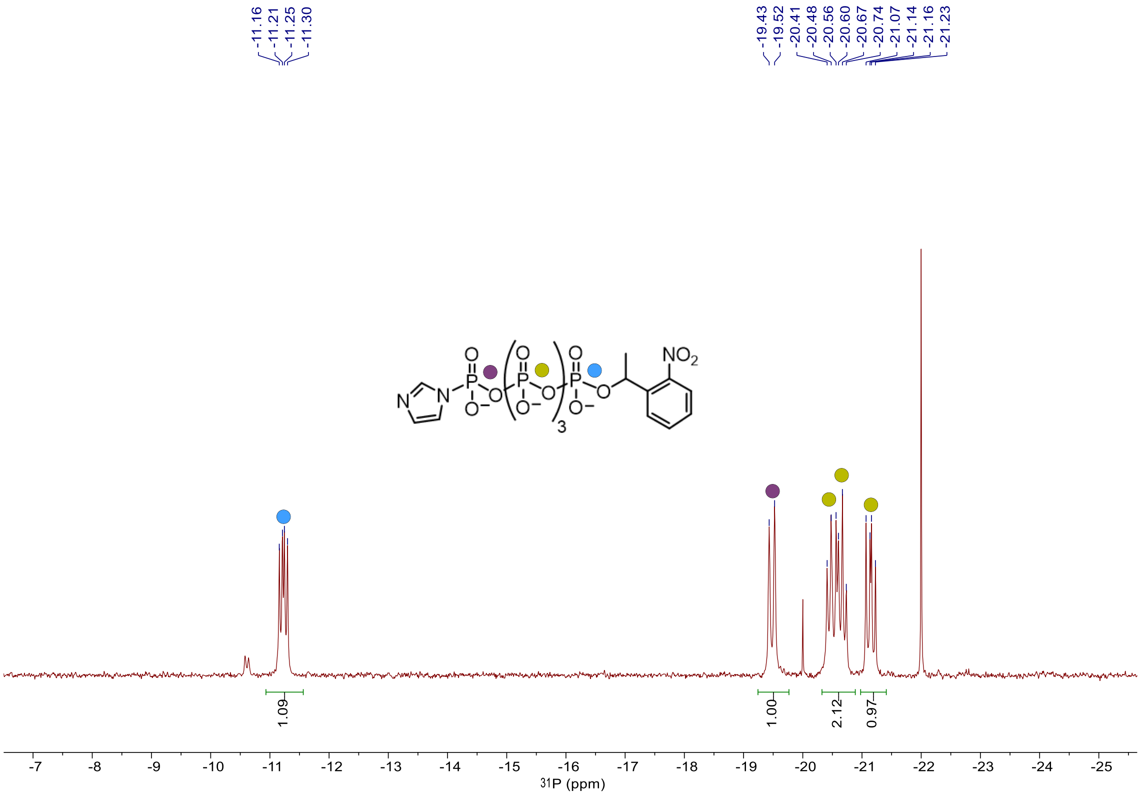

Figure S37: ^{31}P NMR spectrum of **5b**.

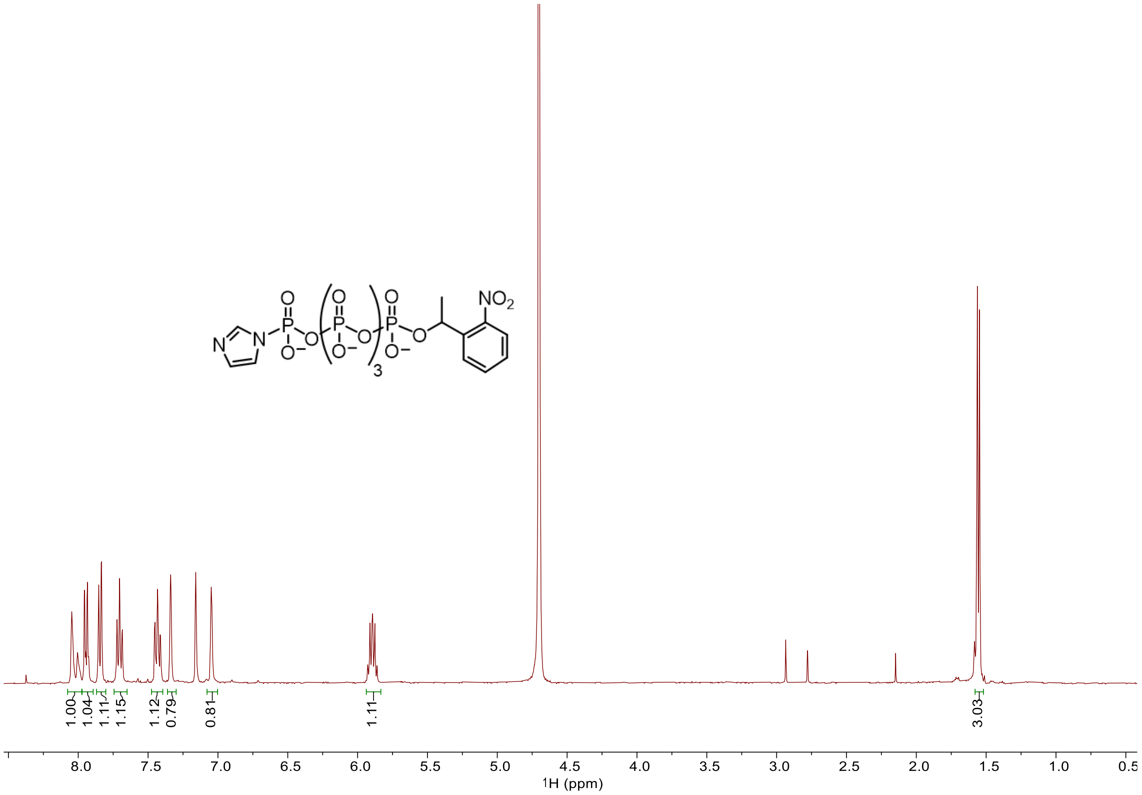

Figure S38: ^{1}H NMR spectrum of **5b**.

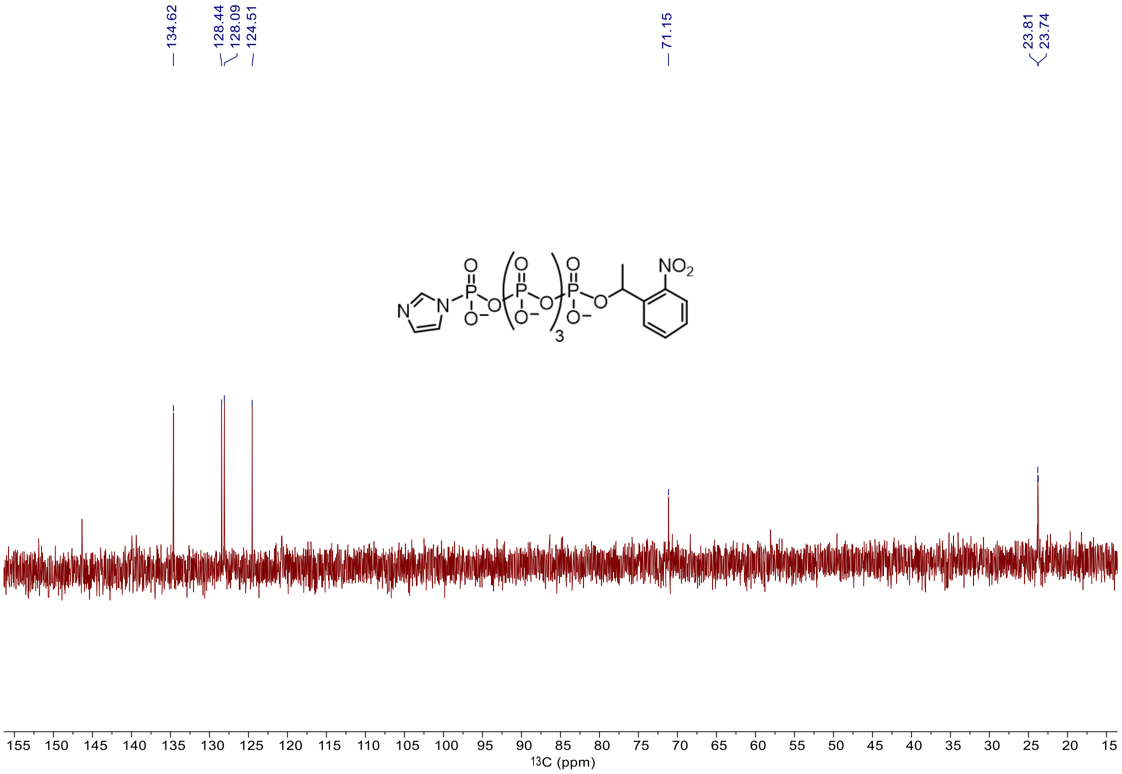

Figure S39: ^{13}C{^{1}H} NMR spectrum of **5b**.

2.5 Synthesis of Oligophosphoryl Diimidazolides

2.5.1 Synthesis of 3c

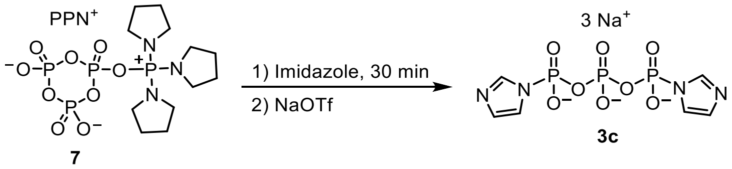

Inside the glovebox, a solution of precursor **7** (1.05 g, 1.0 mmol, 1 equiv.) in ca. 10 mL of acetonitrile was treated with excess imidazole (1.36 g, 20.0 mmol, 20 equiv.) followed by 30 min of stirring. The solution was separated from a brown residue that deposited on the insides of the vial and filtered through Celite. To this pale-yellow filtrate was added a solution of sodium trifluoromethanesulfonate (1.03 g, 6.0 mmol, 6 equiv.) in 20 mL of acetonitrile, which led to the precipitation of the product. The reaction mixture was transferred outside of the glovebox and into a 50 mL Falcon conical centrifuge tube, and centrifuged at 2,000 RPM for 10 min. The supernatant was decanted, and an additional 30 mL of acetonitrile was added to the pellet, which was followed by brief agitation by vortex mixing. The sample was again centrifuged at 2,000 RPM for 5 min, and this washing procedure was repeated for a total of 4 cycles. The product was dried on the vacuum line, yielding pure **3c** as a colorless solid (319 mg, 75% yield).

^{31}P{^{1}H} NMR (162 MHz, D_2O) δ -20.35 (d, *J* = 20.2 Hz, 2P), -24.59 (t, *J* = 20.1 Hz, 1P).

^{31}P NMR (162 MHz, D_2O) δ -20.35 (d, *J* = 20.2 Hz, 2P), -24.59 (t, *J* = 20.1 Hz, 1P).

^{1}H NMR (400 MHz, D_2O) δ 7.77 (br, 2H), 7.15 (q, *J* = 1.6 Hz, 2H), 6.95 (br, 2H).

^{13}C{^{1}H} NMR (101 MHz, D_2O) δ 139.84 (d, *J* = 6.7 Hz), 128.39 (d, *J* = 11.7 Hz), 120.30 (d, *J* = 6.8 Hz).

HRMS(−) (m/z) of $C_6H_8N_4O_8P_3^-$ [M]$^-$: 356.9577 (calc'd 356.9561)

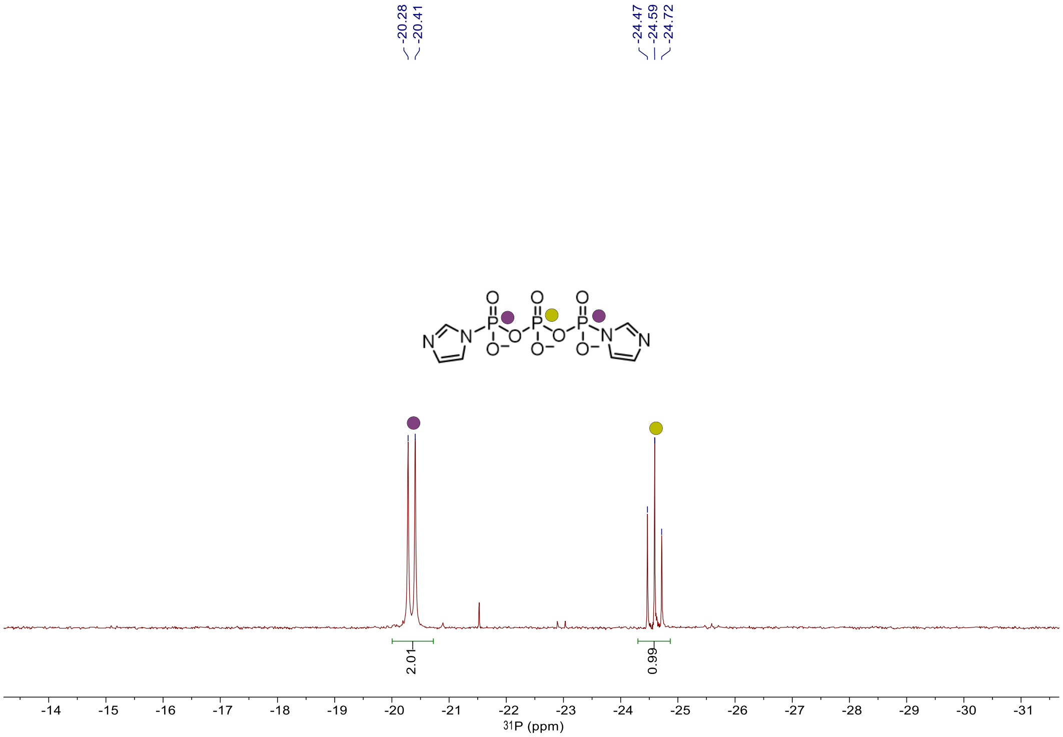

Figure S40: ^{31}P{^{1}H} NMR spectrum of **3c**.

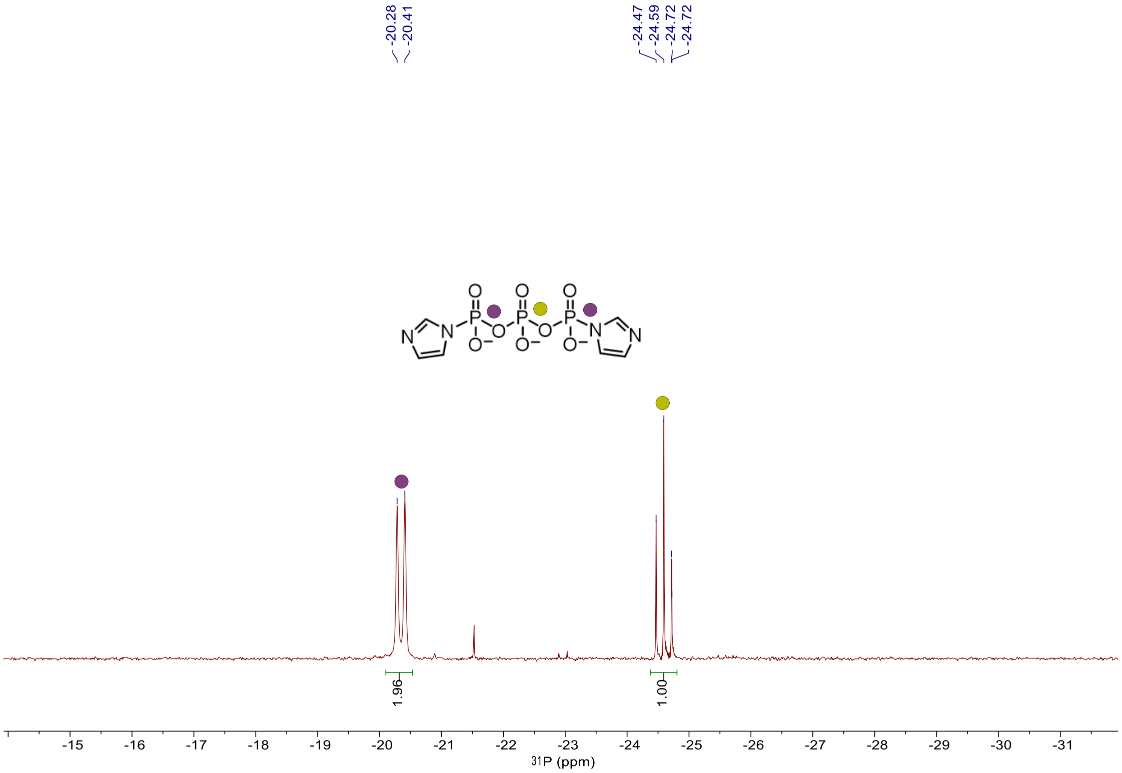

Figure S41: ^{31}P NMR spectrum of **3c**.

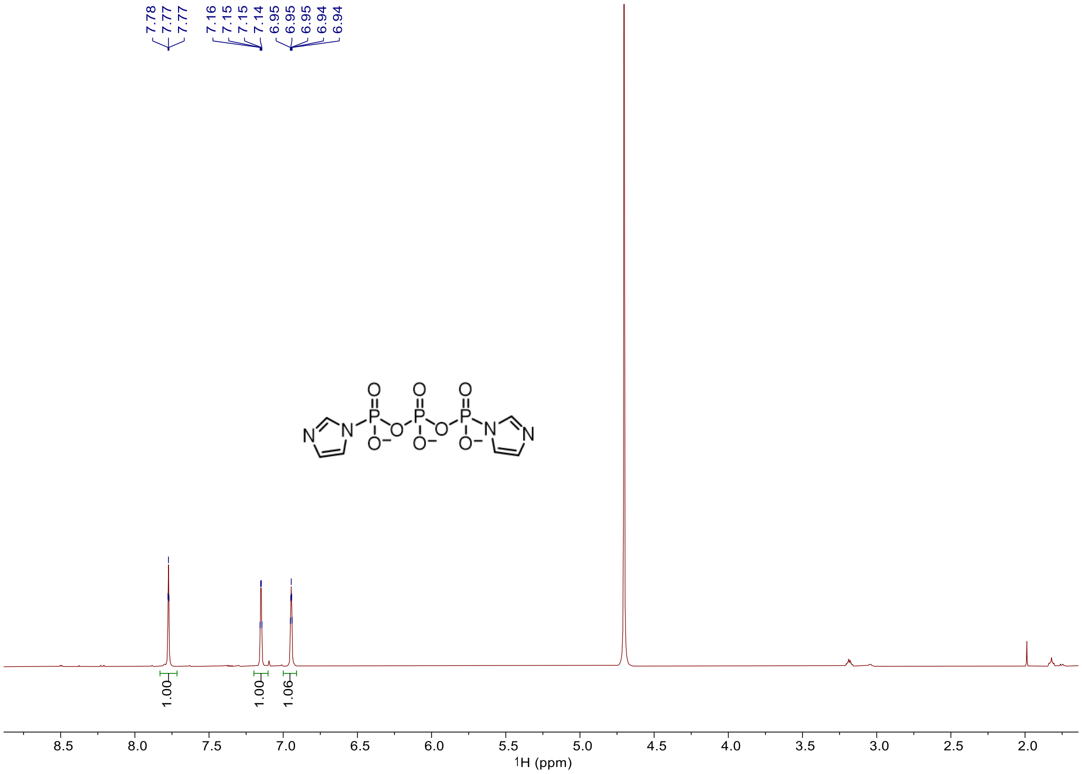

Figure S42: ^{1}H NMR spectrum of **3c**.

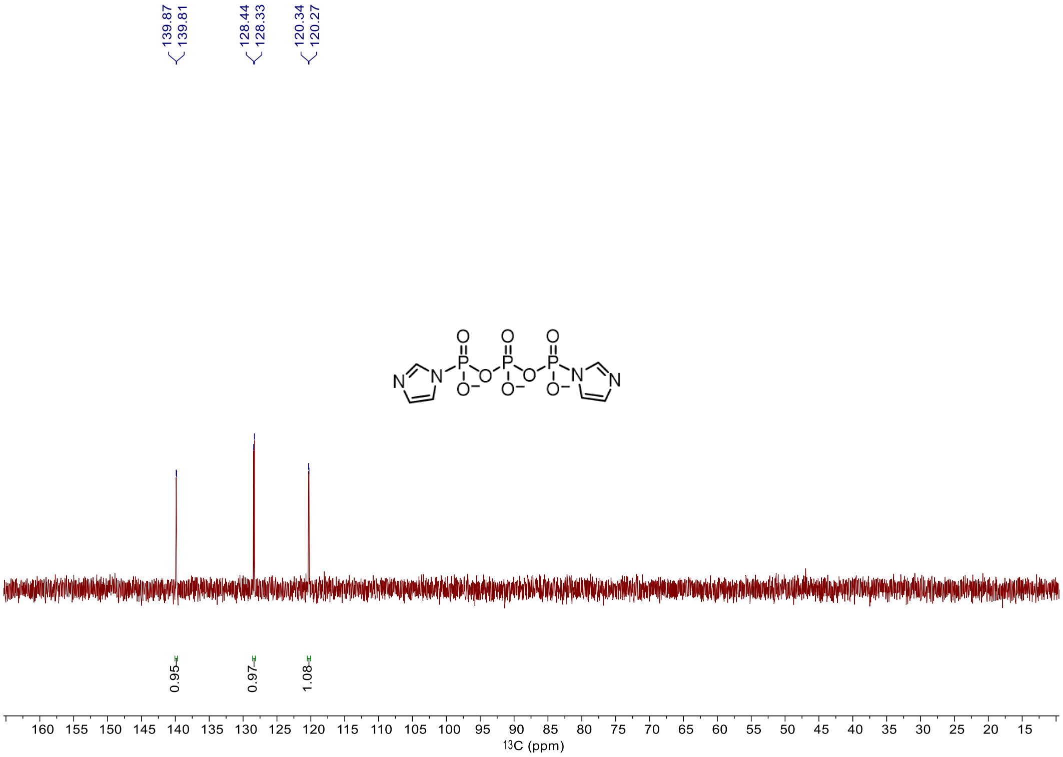

Figure S43: $^{13}C\{^1H\}$ NMR spectrum of **3c**.

2.5.2 Synthesis of 4c

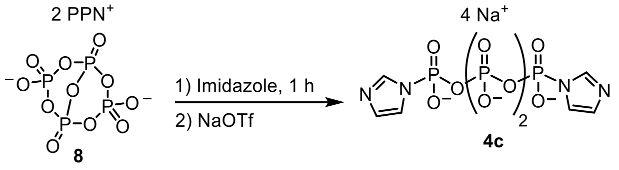

Inside the glovebox, a solution of precursor **8** (0.50 g, 0.36 mmol, 1 equiv.) in ca. 4 mL of acetonitrile was treated with excess imidazole (0.50 g, 7.30 mmol, 20 equiv.) followed by 1 h of stirring. The reaction mixture was transferred outside of the glovebox, and addition of a solution of sodium trifluoromethanesulfonate (0.50 g, 2.9 mmol, 6 equiv.) in 10 mL of acetonitrile led to the precipitation of the product. The reaction mixture was transferred into a 50 mL Falcon conical centrifuge tube, and centrifuged at 2,000 RPM for 10 min. The supernatant was decanted, and an additional 20 mL of acetonitrile was added to the pellet, which was followed by brief agitation by vortex mixing. The sample was again centrifuged at 2,000 RPM for 5 min, and this washing procedure was repeated for a total of 4 cycles. The product was dried on the vacuum line, yielding **4c** as a colorless solid (181 mg, 88% yield*).

*calculated % yield adjusting for metaphosphate impurities present in ^{31}P NMR spectrum.

$^{31}P\{^1H\}$ NMR (162 MHz, D_2O) δ -20.61 (m, 2P), -24.09 (m, 2P).

^{31}P NMR (162 MHz, D_2O) δ -20.61 (m, 2P), -24.09 (m, 2P).

^1H NMR (400 MHz, D_2O) δ 8.04 (br, 2H), 7.24 (br, 2H), 7.05 (br, 2H).

$^{13}C\{^1H\}$ NMR (101 MHz, D_2O) δ 139.41 (d, J = 7.1 Hz), 126.73 (d, J = 10.6 Hz), 120.79 (d, J = 6.1 Hz).

HRMS(−) (m/z) of $C_6H_9N_4O_{11}P_4^-$ $[M]^-$: 436.9220 (calc'd 436.9224)

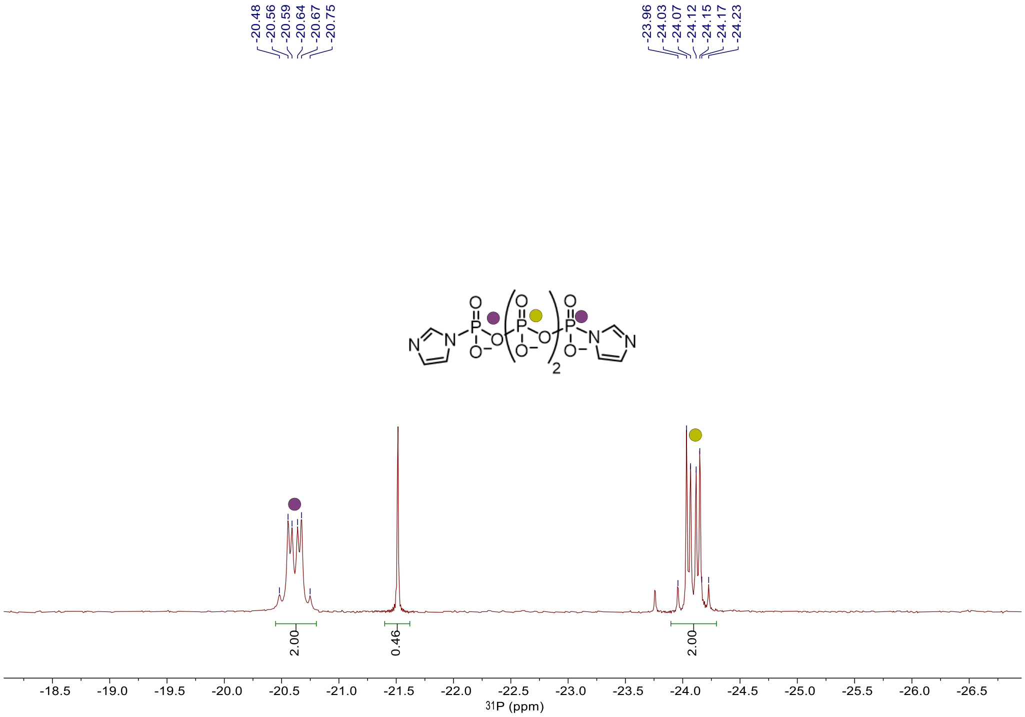

Figure S44: $^{31}P\{^{1}H\}$ NMR spectrum of **4c**.

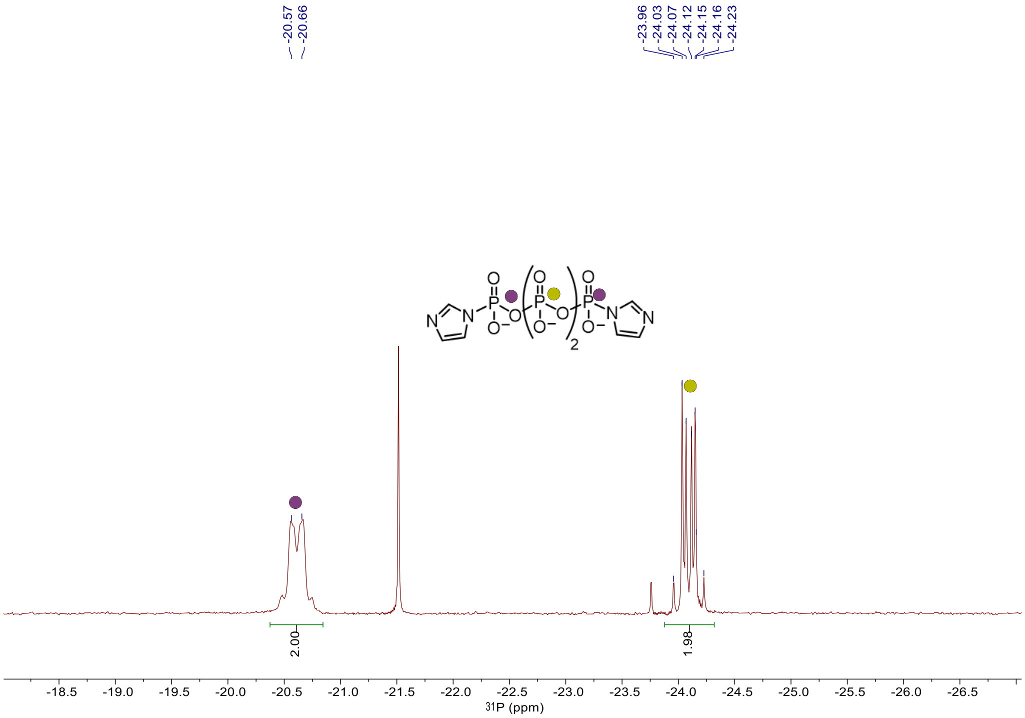

Figure S45: ^{31}P NMR spectrum of **4c**.

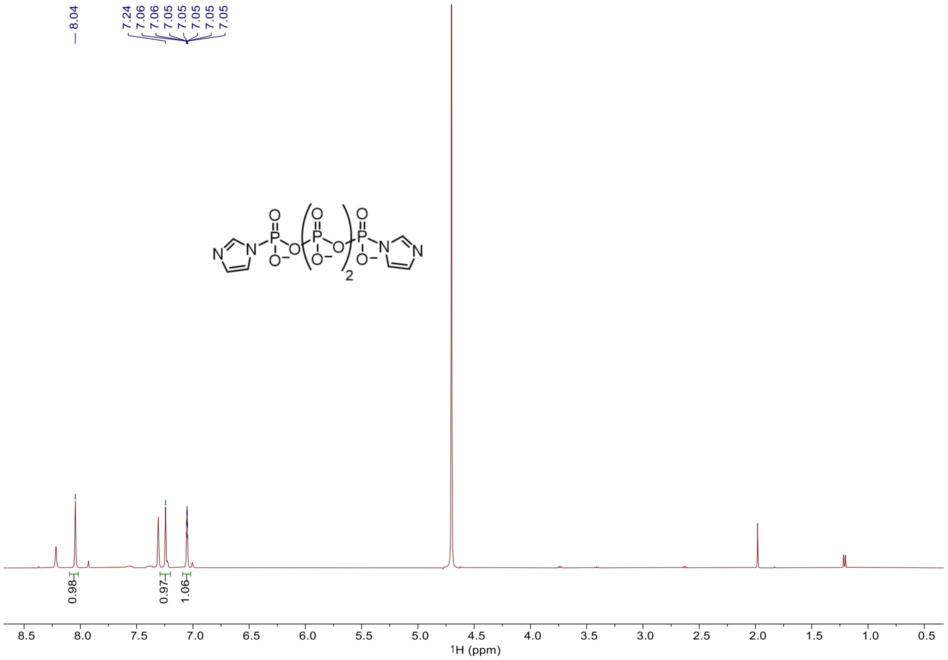

Figure S46: ^{1}H NMR spectrum of **4c**.

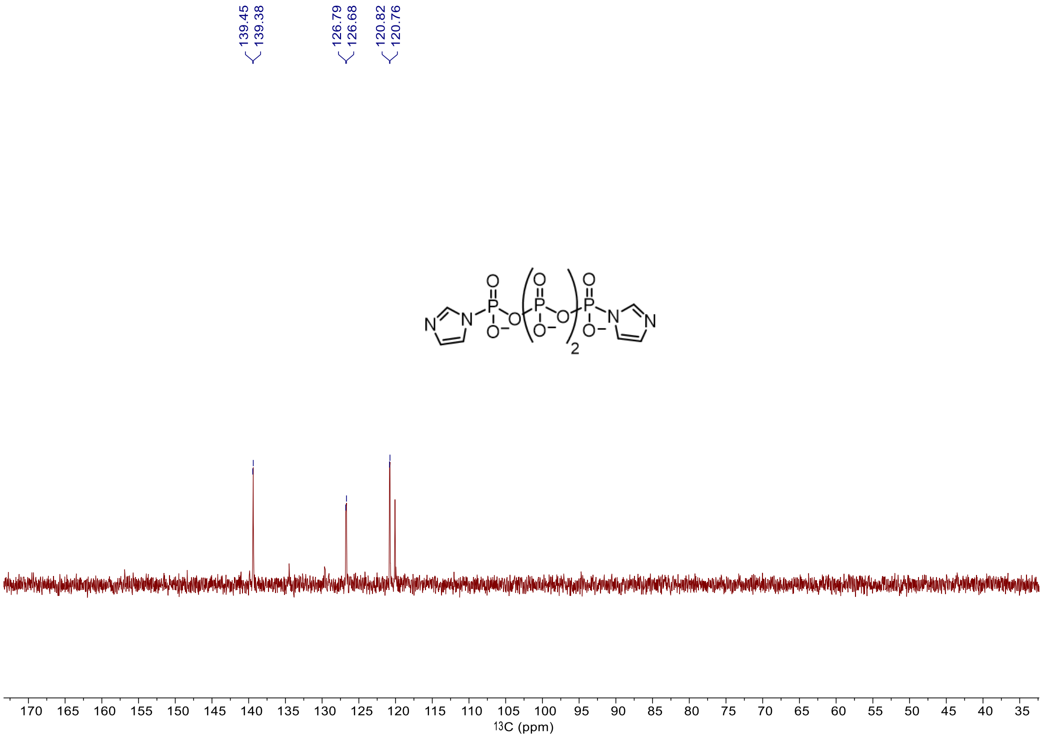

Figure S47: $^{13}C\{^{1}H\}$ NMR spectrum of **4c**.

2.5.3 Synthesis of 5c

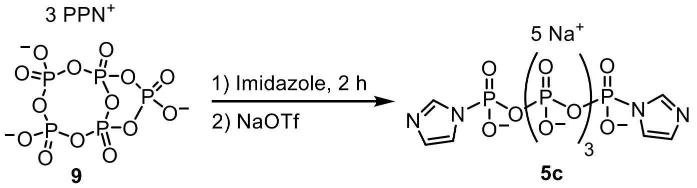

Inside the glovebox, a solution of precursor **9** (100 mg, 0.050 mmol, 1 equiv.) in ca. 2 mL of acetonitrile was treated with excess imidazole (170 mg, 2.5 mmol, 50 equiv.) followed by 2 h of stirring. The reaction mixture was transferred outside of the glovebox, and a solution of sodium trifluoromethanesulfonate (86 mg, 0.50 mmol, 10 equiv.) in 2 mL of acetonitrile led to the precipitation of the product. The reaction mixture was transferred into a 50 mL Falcon conical centrifuge tube, and centrifuged at 2,000 RPM for 10 min. The supernatant was decanted, and an additional 10 mL of acetonitrile was added to the pellet, which was followed by brief agitation by vortex mixing. The sample was again centrifuged at 2,000 RPM for 5 min, and this washing procedure was repeated for a total of 3 cycles. The product was dried on the vacuum line, yielding **5c** as a colorless solid (30 mg, 78% yield*).

*calculated % yield adjusting for metaphosphate impurities present in ^{31}P NMR spectrum.

^{31}P{^{1}H} NMR (162 MHz, D_2O) δ -19.71 (d, *J* = 17.3 Hz, 2P), -23.07 – -23.74 (m, 3P).

^{31}P NMR (162 MHz, D_2O) δ -19.71 (d, *J* = 17.3 Hz, 2P), -23.07 – -23.74 (m, 3P).

^{1}H NMR (400 MHz, D_2O) δ 7.90 (br, 2H), 7.31 (br, 2H), 6.99 (br, 2H).

^{13}C NMR (101 MHz, D_2O) δ 139.99 41 (d, *J* = 6.8 Hz), 128.47 (d, *J* = 10.9 Hz), 120.48 (d, *J* = 6.4 Hz).

HRMS(−): product signals not detected due to poor ionization, which we sometimes observe for longer oligophosphates.

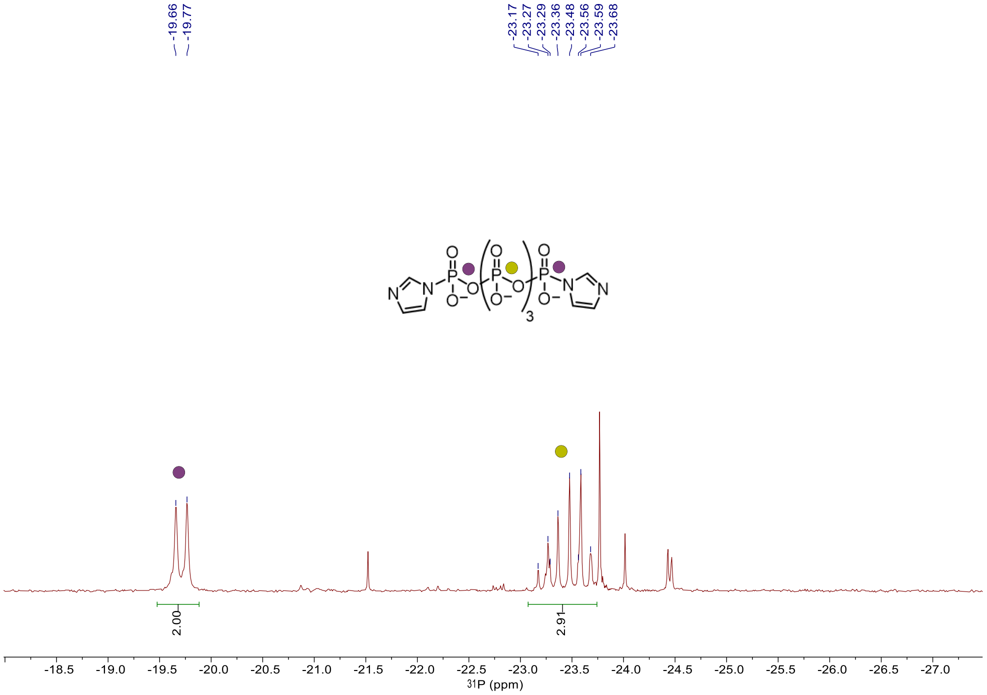

Figure S48: ^{31}P{^{1}H} NMR spectrum of **5c**.

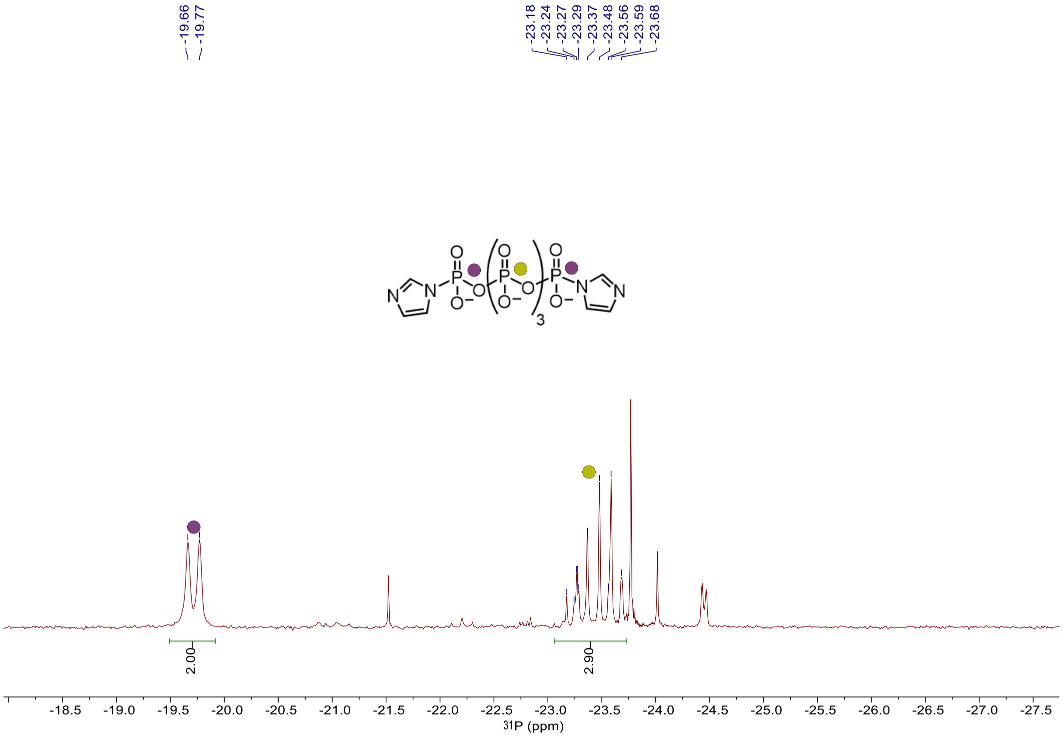

Figure S49: ^{31}P NMR spectrum of **5c**.

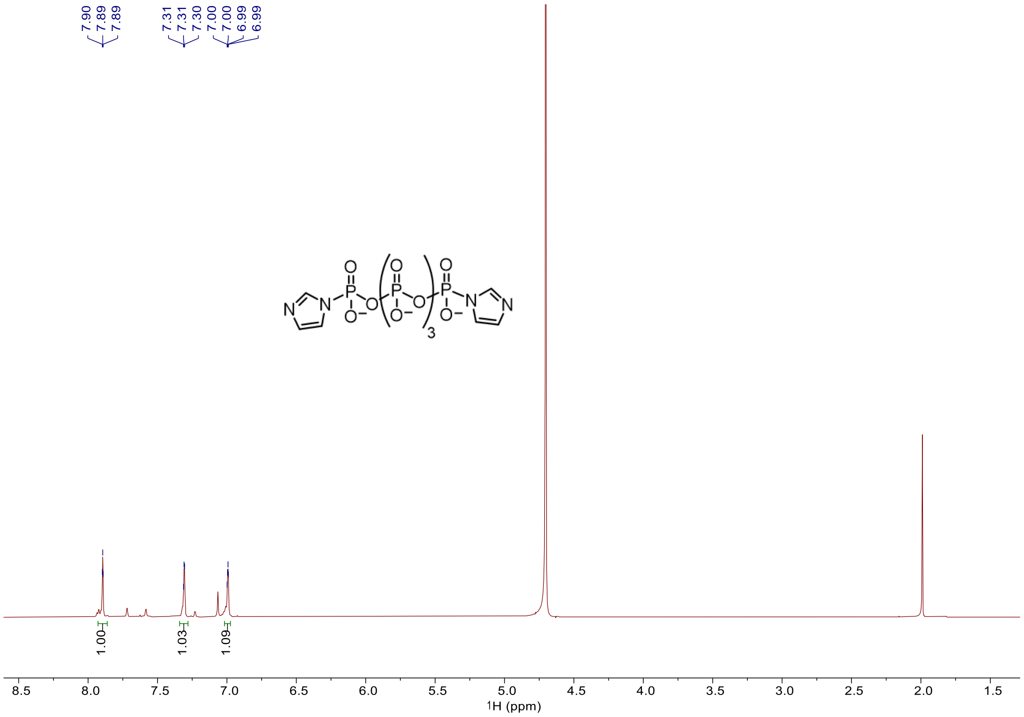

Figure S50: ^{1}H NMR spectrum of **5c**.

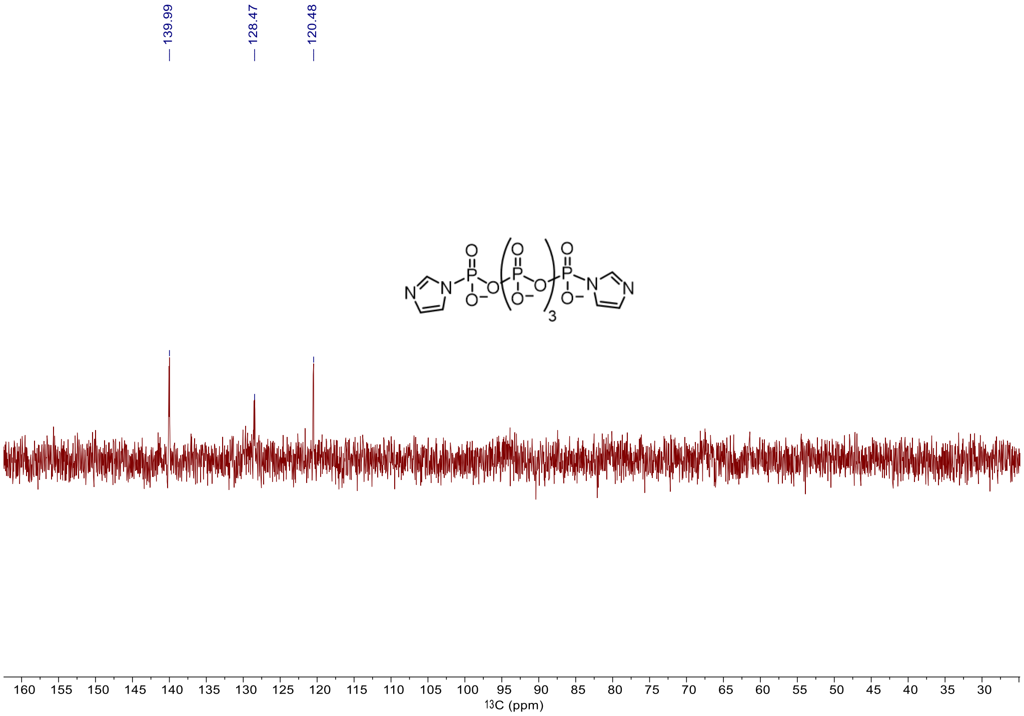

Figure S51: $^{13}C\{^{1}H\}$ NMR spectrum of **5c**.

3 Solid-Phase Peptide Synthesis (SPPS) of Phosphopeptide Substrates

Phosphopeptide substrates for this project were kindly provided by the peptide synthesis core facility at FMP Berlin. The SPPS reactions were conducted in Bio-Rad polypropylene columns at room temperature by agitating with nitrogen using a PEEK switching valve. Fmoc-L-amino acids (AA) were used as starting materials, and the primary coupling and activating agents used were 2-(1*H*-benzotriazole-1-yl)-1,1,3,3-tetramethyluronium hexafluoro-phosphate (HBTU) and hydroxyl-benzotriazole (HOBt), respectively, in DMF. Fmoc-Gly-Wang Resin, Fmoc-Arg(Pbf)-Wang Resin, Fmoc-Lys(Boc)-Wang Resin, Fmoc-Glu(OtBu)-Wang Resin, Fmoc-Rink-Amide Resin, Ninhydrin Test Kit, coupling reagents, activating agents, and Fmoc-L-AA starting materials were purchased from Iris Biotech and Sigma Aldrich.

Phosphopeptides **p-Pep1** to **p-Pep7** were prepared by a standard fluorenylmethyloxy-carbonyl (Fmoc) mode solid phase peptide synthesis (SPPS) protocol.[13] Briefly, 0.05–0.2 mmol of resin was swelled for 30 min in DMF. The resin was treated with 20% piperidine in DMF for 20 min to deprotect the Fmoc group. The resin was incubated with the activated AA (5.00 equiv.) in 0.5 M HBTU/HOBt in DMF for 30 min with N_2 agitation. After allowing the suspension to react for 30 min, the resin was rinsed with DMF and was dried briefly. A small aliquot of resin was treated with the Ninhydrin Test Kit reagents at 100 °C for 6 min to monitor for coupling efficiency. If the test result was negative, the cycle was repeated until the final AA was coupled; if the test result was positive, the resin was incubated with an additional portion of activated AA (5.00 equiv.) in 0.5 M HBTU/HOBt solution. Upon completion of the AA sequence and a final Fmoc-deprotection step, the full-length peptide was cleaved and globally deprotected with a TFA cocktail [95% TFA, 2.5% triisopropylsilane (TIS), 2.5% H_2O, by volume] for 3 h to yield the crude peptides. The crude peptides were purified by preparative HPLC (**Method I**), and the isolated yields reported are based on initial resin loading (for all peptides: 200 µmol; Supporting Table 1). Characterization data for all isolated peptides by analytical HPLC and HRMS as described in SI section 1 is summarized in SI section 6.

p-Pep3 was synthesized on an Fmoc-Arg(Pbf)-Wang Resin, **p-Pep4**, **p-Pep5**, and **p-Pep6** were synthesized on an Fmoc-Lys(Boc)-Wang Resin and **p-Pep7** was synthesized on an Fmoc-Glu(OtBu)-Wang Resin using the standard Fmoc-mode coupling protocol as described above. **p-Pep1** and **p-Pep2** were synthesized on an Fmoc-Rink-Amide Resin using the standard Fmoc-mode coupling protocol as described above. After the final Fmoc-deprotection step, the resin was treated with acetic anhydride (Ac_2O, 20.0 equiv.) and DIPEA (40.0 equiv.) in DMF (3 x 10 min) to cap the *N*-terminus. The TFA cleavage time was increased to 4 h to ensure complete deprotection of the benzyl group on the phosphothreonine/-serine residue.

Supporting Table 1: SPPS yields for phosphopeptides (initial resin loading: 200 µmol).

Entry	Compound	Sequence	Yield
1	**p-Pep1**	AcHN-WNA[pT]ANG-CONH$_2$	81 mg, 95 µmol (**48%**)
2	**p-Pep2**	AcHN-WNA[pS]ANG-CONH$_2$	29 mg, 35 µmol (**18%**)
3	**p-Pep3**	H$_2$N-VMLGE[pT]NPADSKPGTIR-COOH	79 mg, 42 µmol (**21%**)
4	**p-Pep4**	H$_2$N-SPTP[pS]PSPPRNSDQEGGGK-COOH	100 mg, 51 µmol (**26%**)
5	**p-Pep5**	H$_2$N-KESE[pS]EDSSDDEPLIK-COOH	96 mg, 51 µmol (**26%**)
6	**p-Pep6**	H$_2$N-VEEDAE[pS]EDEEEEDVK-COOH	70 mg, 36 µmol (**18%**)
7	**p-Pep7**	H$_2$N-KKAS[pS]SDSEDSSEEE-COOH	66 mg, 39 µmol (**20%**)

4 Synthesis of Pyrophosphorylated Peptides

4.1 Synthesis of NPE-protected Pyrophosphopeptides

The *o*-nitrophenylethyl (NPE) protected pyrophosphopeptides **NPE-p$_2$-Pep1** to **NPE-p$_2$-Pep7** were synthesized using a modified procedure from Marmelstein *et al*.[14] Lithium 1-(2-nitrophenyl)ethyl-1*H*-imidazol-1-ylphosphonate (**1b**) was prepared according to previously published protocols.[4] Spectral data for **1b** matches previously reported values.

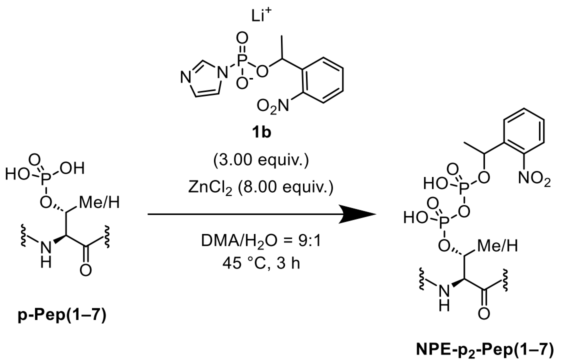

Figure S52: General reaction scheme for the phosphorylation of phosphopeptides **p-Pep1** to **p-Pep7** with monoP-imidazolide reagent **1b**.

__**General procedure 1:**__
Phosphopeptide (4.00 mg., 1.00 equiv.) was dissolved in DMA (240 µL) and Milli-Q water (60 µL) with the assistance of sonication. In parallel, NPE-P-imidazolide (**1b**, 3.00 equiv.) and zinc chloride (8.00 equiv.) were suspended in DMA (300 µL) and dissolved with the assistance of sonication. Both solutions were combined and the resulting clear colorless reaction mixture was shaken at 45 °C for 2–3 h. Afterward, the reaction mixture was diluted with a Milli-Q water/ acetonitrile mixture (9:1 + 0.1% formic acid, 4 mL), filtered through a syringe filter (RC 0.45 µm), and submitted to preparative HPLC (**Method I**). After lyophilization of the product containing fractions, the NPE-protected pyrophosphopeptide was obtained as a white solid.

Yields determined by weight for **NPE-p$_2$-Pep1** to **NPE-p$_2$-Pep7** are summarized in Supporting Table 2. Characterization data for all isolated peptides by analytical HPLC and HRMS as described in SI section 1 is summarized in SI section 6.

Supporting Table 2: Yields for NPE-protected pyrophosphopeptides.

Entry	Compound	Sequence	% Yield
1	**NPE-p$_2$-Pep1**	AcHN-WNA**[NPE-ppT]**ANG-CONH$_2$	51 (98[a])
2	**NPE-p$_2$-Pep2**	AcHN-WNA**[NPE-ppS]**ANG-CONH$_2$	40 (97[a])
3	**NPE-p$_2$-Pep3**	H$_2$N-VMLGE**[NPE-ppT]**NPADSKPGTIR-COOH	37 (95[a])
4	**NPE-p$_2$-Pep4**	H$_2$N-SPTP**[NPE-ppS]**PSPPRNSDQEGGGK-COOH	31 (92[a])
5	**NPE-p$_2$-Pep5**	H$_2$N-KESE**[NPE-ppS]**EDSSDDEPLIK-COOH	45 (82[a])
6	**NPE-p$_2$-Pep6**	H$_2$N-VEEDAE**[NPE-ppS]**EDEEEEDVK-COOH	15 (50[a])
7	**NPE-p$_2$-Pep7**	H$_2$N-KKAS**[NPE-ppS]**SDSEDSSEEE-COOH	27 (80[a])

[a]Conversion after 3 h determined by analytical HPLC.

4.2 Photodeprotection of Pyrophosphopeptides

The unprotected pyrophosphopeptides **p_2-Pep1** to **p_2-Pep7** were synthesized using a modified procedure from Marmelstein *et al.*[4]

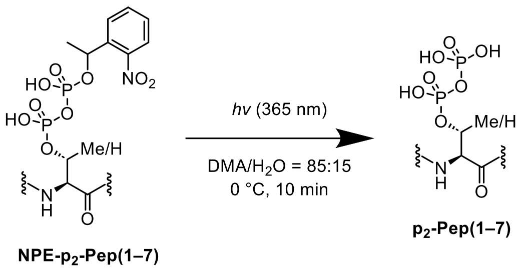

Figure S53: General reaction scheme for the photodeprotection of NPE-protected pyrophosphopeptides **NPE-p_2-Pep1** to **NPE-p_2-Pep7** by irradiation with UV light.

<u>**General procedure 2:**</u>
NPE-protected pyrophosphopeptide was dissolved in DMA (425 µL) and Milli-Q water (75 µL). The clear colorless reaction mixture was irradiated with UV light (365 nm) at 0 °C for 10 min. The solution turned from colorless to slightly yellow-brownish. Afterward, the reaction mixture was diluted with a Milli-Q water/ acetonitrile mixture (9:1 + 0.1% formic acid, 3750 µL), filtered through a syringe filter (RC 0.45 µm), and submitted to preparative HPLC (**Method I**). After lyophilization of the product containing fractions, the pyrophosphopeptide was obtained as a white solid.

Yields determined by weight for **p_2-Pep1** to **p_2-Pep7** are summarized in Supporting Table 3. Characterization data for all isolated peptides by analytical HPLC and HRMS as described in SI section 1 is summarized in SI section 6.

Supporting Table 3: Yields for free pyrophosphopeptides.

Entry	Compound	Sequence	% Yield
1	**p_2-Pep1**	AcHN-WNA**[ppT]**ANG-$CONH_2$	28 (92[a])
2	**p_2-Pep2**	AcHN-WNA**[ppS]**ANG-$CONH_2$	26 (90[a])
3	**p_2-Pep3**	H_2N-VMLGE**[ppT]**NPADSKPGTIR-COOH	23 (70[a])
4	**p_2-Pep4**	H_2N-SPTP**[ppS]**PSPPRNSDQEGGGK-COOH	16 (90[a])
5	**p_2-Pep5**	H_2N-KESE**[ppS]**EDSSDDEPLIK-COOH	18 (65[a])
6	**p_2-Pep6**	H_2N-VEEDAE**[ppS]**EDEEEEDVK-COOH	14 (91[a])
7	**p_2-Pep7**	H_2N-KKAS**[ppS]**SDSEDSSEEE-COOH	11 (89[a])

[a]Conversion after 10 min determined by analytical HPLC.

5 Synthesis of Oligophosphorylated Peptides

5.1 Additional Optimization of Reaction Conditions for Oligophosphorylation

Besides the optimization of the reaction of **p-Pep1** and tetraphosphorylation reagent **4a** (Table 1), conditions were screened for the triphosphorylation of model phosphoserine substrate **p-Pep2** with reagent **3b** (Supporting Table 4). Compared to the previously described pyrophosphorylation reactions (SI section 4), increased amounts of both reagent and $ZnCl_2$ were needed to achieve optimal conversion to **NPE-p$_4$-Pep2**. Additionally, it was crucial in this reaction to remove any amount of water hinting toward water-induced deceleration of the reaction kinetics probably by creating a solvation shell around the negatively charged phosphoryl groups and/or metal promoter and therefore impeding reactivity.

Supporting Table 4: Optimization of conditions for oligophosphorylation of pSer model peptide **p-Pep2** with triphosphorylation reagent **3b**.

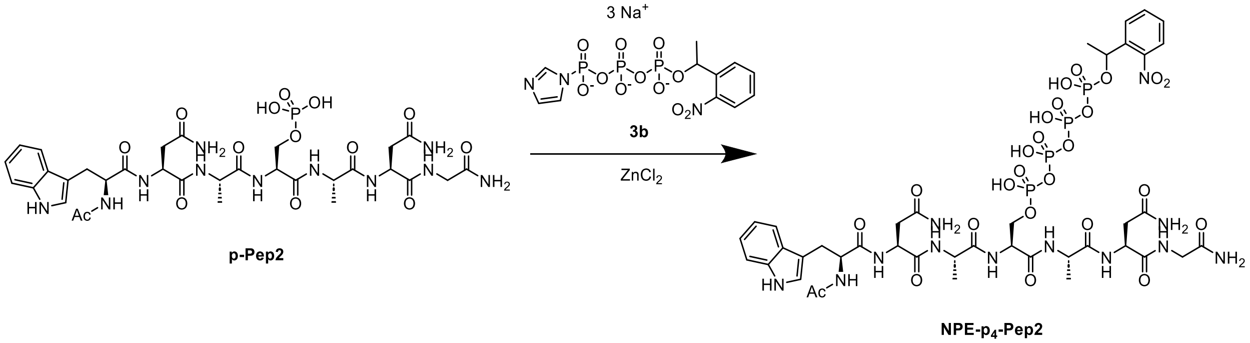

Entry	Reagent **3b** (equiv.)	Zn^{2+} (equiv.)	Solvent	Temp. (°C)	Time (h)	% Conversion[a]
1	3.00	8.00	DMA/H_2O (9:1)	45	18	5
2	5.00	13.0	DMA/H_2O (9:1)	45	18	8
3	5.00	50.0	DMA/H_2O (9:1)	45	18	11
4	5.00	13.0	DMA/H_2O (3:1)	45	18	4
5	5.00	13.0	DMSO	45	18	0[b]
6	5.00	100	DMA/H_2O (9:1)	45	18	10
7	9.00	25.0	DMA/H_2O (9:1)	45	18	19
8	10.0	25.0	DMA	45	18	93

[a]Conversion determined by analytical HPLC. [b]product not observed.

^{31}P Nuclear magnetic resonance (NMR) spectroscopy of the HPLC-purified compound confirmed the attachment of a linear oligoP chain with **3b** to phosphopeptide **p-Pep2**.

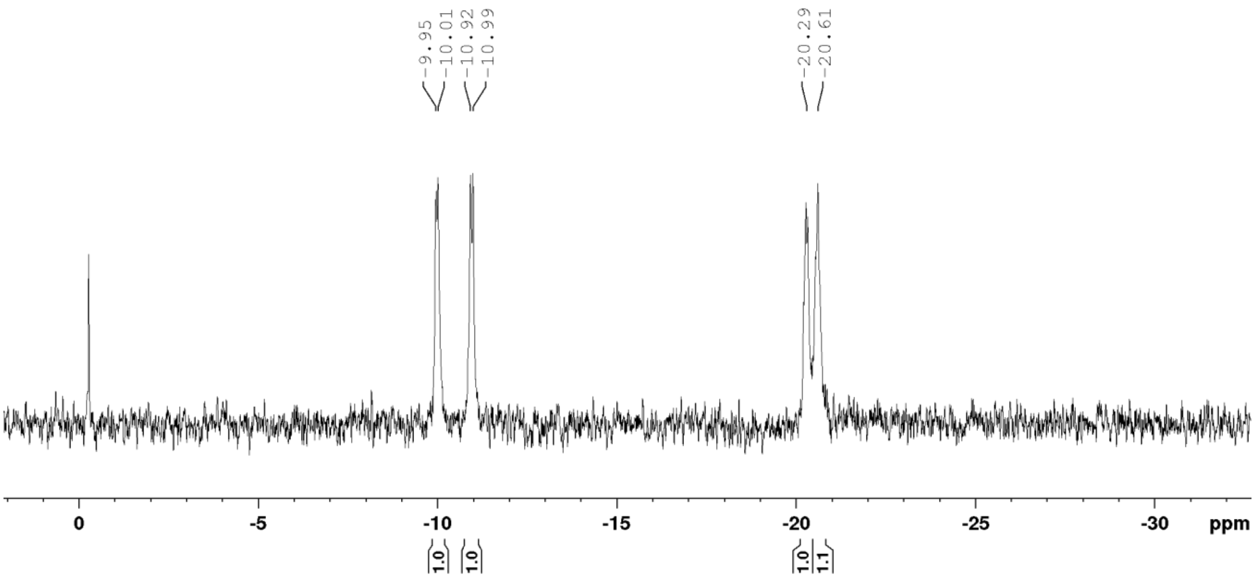

Figure S54: ^{31}P{^{1}H} NMR spectrum of **NPE-p$_4$-Pep2**.

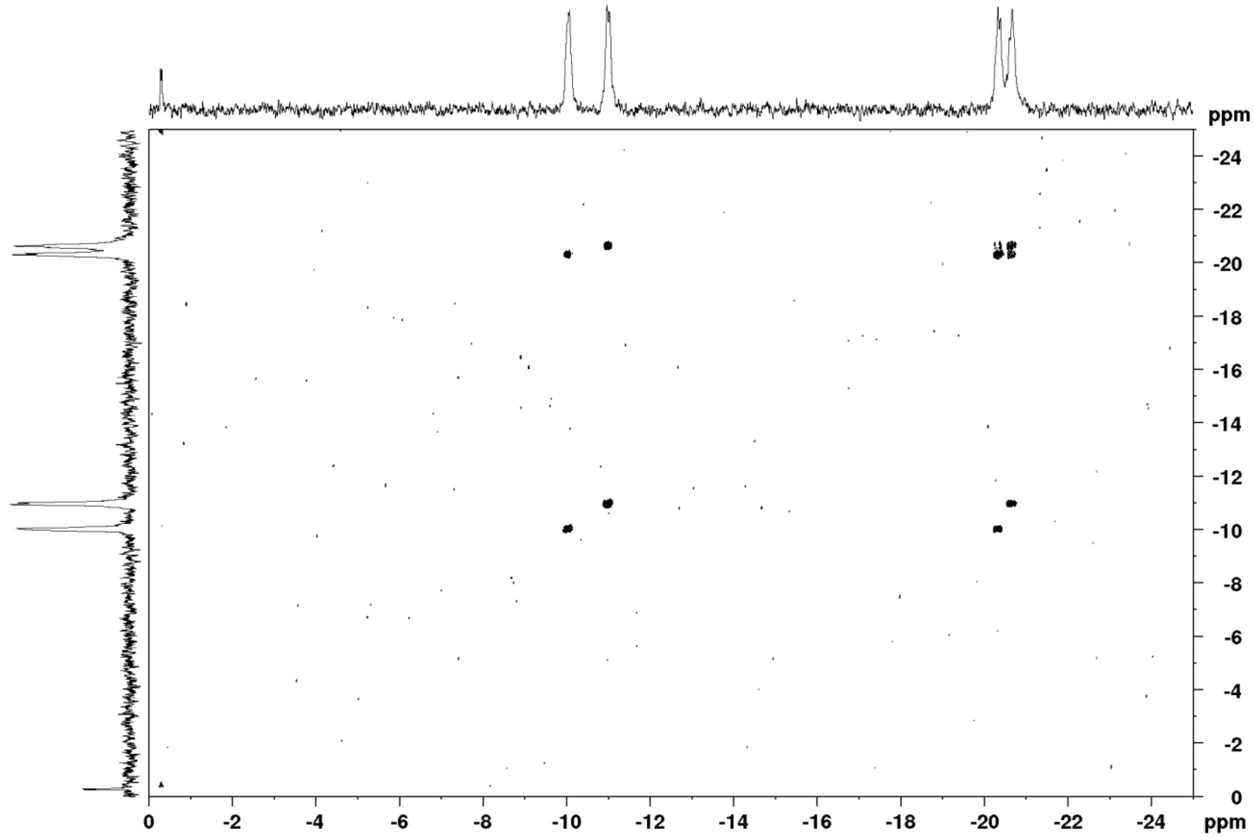

Figure S55: ^{31}P-^{31}P COSY NMR spectrum of **NPE-p$_4$-Pep2**.

To screen the conversion of a more complex phosphopeptide substrate without any aromatic amino acid side chains, the reaction of NME1 peptide **p-Pep3** with triphosphorylation reagent **3b** was investigated in more detail (Supporting Table 5). Following the observed trends for the two model peptides **p-Pep1** (Table 1) and **p-Pep2** (Supporting Table 4), the reaction worked best in pure DMA. Interestingly, an exact ratio of reagent to Zn^{2+} of 1:8 improved the solubility of **3b** in pure DMA immensely. Moreover, the reaction proceeded faster at slightly elevated temperatures with up to 94% conversion reached after 3 h.

Supporting Table 5: Optimization of conditions for oligophosphorylation of NME1 peptide **p-Pep3** with triphosphorylation reagent **3b**.

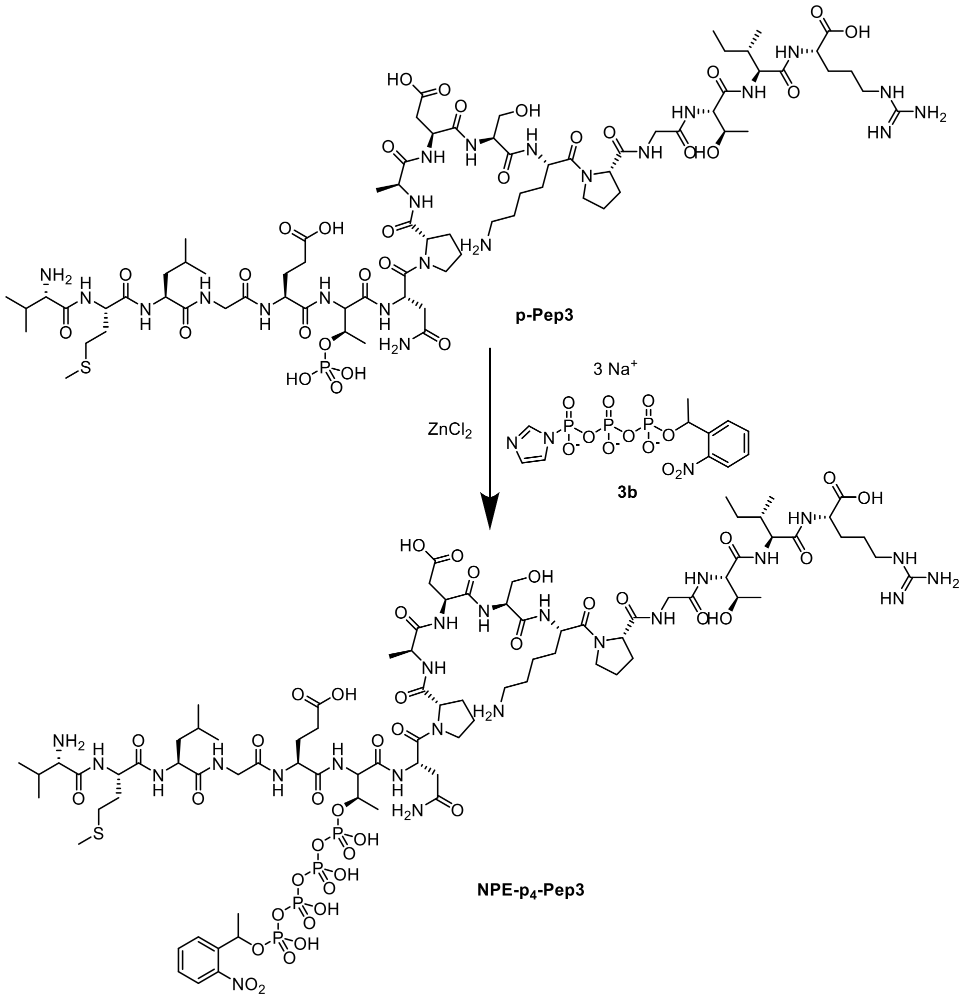

Entry	Reagent **3b** (equiv.)	Zn^{2+} (equiv.)	Solvent	Temp. (°C)	Time (h)	% Conversion[a]
1	10.0	40.0	DMA/H_2O (95:5)	45	18	40
2	10.0	40.0	DMA	45	6	50
3	15.0	40.0	DMA	45	6	50
4	10.0	60.0	DMA	55	6	85
5	10.0	60.0	DMA	45	10	70

6	3.00	18.0	DMA	55	6	80
7	5.00	40.0	DMA	55	3	94
8	3.00	24.0	DMA	55	3	86
9	3.00	30.0	DMA	55	3	60

[a]Conversion determined by analytical HPLC.

5.2 Procedures for Chemical Synthesis of Oligophosphorylated Peptides

Following the optimization of the oligophosphorylation of NME1 peptide **p-Pep3**, general protocols for the reaction of a phosphopeptide substrate with an oligoP-imidazolide reagent were prepared and experiments were conducted as described below.

5.2.1 Synthesis of NPE-protected Oligophosphopeptides

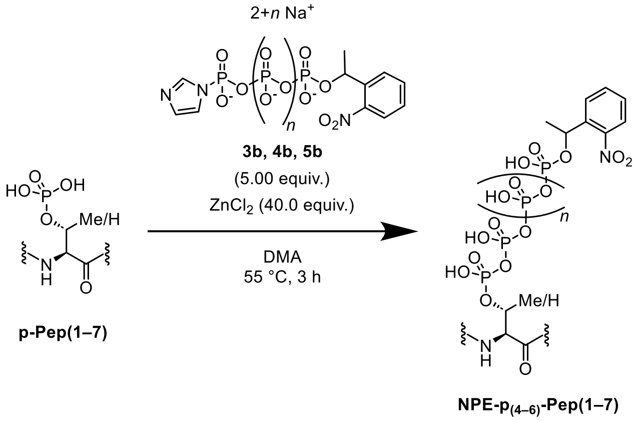

Figure S56: General reaction scheme for the oligophosphorylation of phosphopeptides **p-Pep1** to **p-Pep7** with NPE-derivatized triphosphorylation reagent **3b** (*n* = 1), tetraphosphorylation reagent **4b** (*n* = 2), and pentaphosphorylation reagent **5b** (*n* = 3).

<u>General procedure 3:</u>

Phosphopeptide (4.00 mg, 1.00 equiv.) was dissolved in DMA (1.50 mL) with the assistance of sonication.[a] In parallel, NPE-derivatized oligophosphorylation reagent **3b**, **4b**, or **5b** (5.00 equiv. respectively) and zinc chloride (40.0 equiv.) were suspended in DMA (1.50 mL) and dispersed with the assistance of sonication. Both solutions were combined and the resulting cloudy colorless reaction mixture was shaken at 55 °C for 3 h. Afterward, the reaction mixture was diluted with a Milli-Q water/ acetonitrile mixture (9:1 + 0.1% formic acid, 12 mL), filtered through a syringe filter (RC 0.45 µm), and submitted to preparative HPLC (**Method I**). After lyophilization of the product containing fractions, the respective NPE-protected oligophosphopeptide was obtained as a white solid.

Yields determined by weight for **NPE-p$_4$-Pep1** to **NPE-p$_6$-Pep1**, **NPE-p$_4$-Pep2** to **NPE-p$_6$-Pep2**, and **NPE-p$_4$-Pep3** to **NPE-p$_6$-Pep3**, as well as reaction conversions determined by HPLC-UV for **NPE-p$_4$-Pep4** to **NPE-p$_6$-Pep4**, **NPE-p$_4$-Pep5** to **NPE-p$_6$-Pep5**, **NPE-p$_4$-Pep6** to **NPE-p$_6$-Pep6**, and **NPE-p$_4$-Pep7** to **NPE-p$_6$-Pep7** are summarized in Supporting Table 6. Characterization data for all isolated peptides by analytical HPLC and HRMS as described in SI section 1 is summarized in SI section 6.

[a]For **p-Pep7** only, Milli-Q water (150 µL) was added due to solubility issues to reach a final DMA/H_2O ratio of approx. 95:5.

Supporting Table 6: Yields and reaction conversions for NPE-protected peptide tetra-, penta-, and hexaphosphates synthesized with reagents **3b** (p$_4$), **4b** (p$_5$), and **5b** (p$_6$), respectively.

Entry	Compound	Sequence	% Yield
1	**NPE-p$_4$-Pep1**	AcHN-WNA**[NPE-ppppT]**ANG-CONH$_2$	41 (92[a])
2	**NPE-p$_5$-Pep1**	AcHN-WNA**[NPE-pppppT]**ANG-CONH$_2$	39 (94[a])
3	**NPE-p$_6$-Pep1**	AcHN-WNA**[NPE-ppppppT]**ANG-CONH$_2$	37 (96[a])
4	**NPE-p$_4$-Pep2**	AcHN-WNA**[NPE-ppppS]**ANG-CONH$_2$	40 (93[a])
5	**NPE-p$_5$-Pep2**	AcHN-WNA**[NPE-pppppS]**ANG-CONH$_2$	n.d.[c]
6	**NPE-p$_6$-Pep2**	AcHN-WNA**[NPE-ppppppS]**ANG-CONH$_2$	n.d.[c]
7	**NPE-p$_4$-Pep3**	H$_2$N-VMLGE**[NPE-ppppT]**NPADSKPGTIR-COOH	28 (94[a])
8	**NPE-p$_5$-Pep3**	H$_2$N-VMLGE**[NPE-pppppT]**NPADSKPGTIR-COOH	23 (95[a])
9	**NPE-p$_6$-Pep3**	H$_2$N-VMLGE**[NPE-ppppppT]**NPADSKPGTIR-COOH	26 (90[a])
10	**NPE-p$_4$-Pep4**	H$_2$N-SPTP**[NPE-ppppS]**PSPPRNSDQEGGGK-COOH	70[a]
11	**NPE-p$_5$-Pep4**	H$_2$N-SPTP**[NPE-pppppS]**PSPPRNSDQEGGGK-COOH	76[a]
12	**NPE-p$_6$-Pep4**	H$_2$N-SPTP**[NPE-ppppppS]**PSPPRNSDQEGGGK-COOH	40[a]
13	**NPE-p$_4$-Pep5**	H$_2$N-KESE**[NPE-ppppS]**EDSSDDEPLIK-COOH	80[a]
14	**NPE-p$_5$-Pep5**	H$_2$N-KESE**[NPE-pppppS]**EDSSDDEPLIK-COOH	70[a]
15	**NPE-p$_6$-Pep5**	H$_2$N-KESE**[NPE-ppppppS]**EDSSDDEPLIK-COOH	50[a]
16	**NPE-p$_4$-Pep6**	H$_2$N-VEEDAE**[NPE-ppppS]**EDEEEEDVK-COOH	92[a]
17	**NPE-p$_5$-Pep6**	H$_2$N-VEEDAE**[NPE-pppppS]**EDEEEEDVK-COOH	40[a]
18	**NPE-p$_6$-Pep6**	H$_2$N-VEEDAE**[NPE-ppppppS]**EDEEEEDVK-COOH	49[a]
19	**NPE-p$_4$-Pep7**	H$_2$N-KKAS**[NPE-ppppS]**SDSEDSSEEE-COOH	50[a]
20	**NPE-p$_5$-Pep7**	H$_2$N-KKAS**[NPE-pppppS]**SDSEDSSEEE-COOH	65[a]
21	**NPE-p$_6$-Pep7**	H$_2$N-KKAS**[NPE-ppppppS]**SDSEDSSEEE-COOH	40[a]

[a]Conversion after 3 h determined by analytical HPLC. [b]product not observed. [c]not determined.

5.2.2 Photodeprotection of Oligophosphopeptides

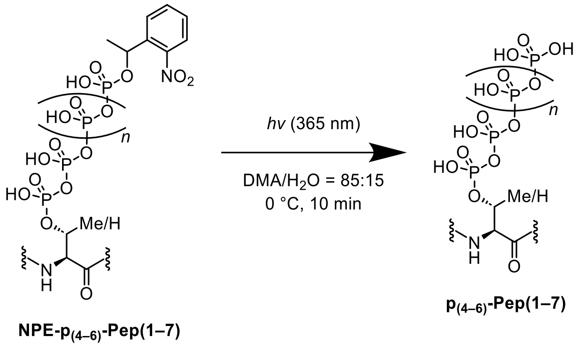

Figure S57: General reaction scheme for the photodeprotection of NPE-protected oligophosphopeptides **NPE-p$_4$-Pep1** to **NPE-p$_4$-Pep7** (*n* = 1), **NPE-p$_5$-Pep1** to **NPE-p$_5$-Pep7** (*n* = 2), and **NPE-p$_6$-Pep1** to **NPE-p$_6$-Pep7** (*n* = 3) by irradiation with UV light.

General procedure 4:

NPE-protected oligophosphopeptide was dissolved in DMA (425 µL) and Milli-Q water (75 µL). The clear colorless reaction mixture was irradiated with UV light (365 nm) at 0 °C for 10 min. The solution turned from colorless to slightly yellow-brownish. Afterward, the reaction mixture was diluted with a Milli-Q water/acetonitrile mixture (9:1 + 0.1% formic acid, 3750 µL), filtered through a syringe filter (RC 0.45 µm), and submitted to preparative HPLC (**Method I**). After lyophilization of the product containing fractions, the respective free oligophosphopeptide was obtained as a white solid.

Yields determined by weight for **p$_4$-Pep1** to **p$_6$-Pep1**, **p$_4$-Pep2** to **p$_6$-Pep2**, and **p$_4$-Pep3** to **p$_6$-Pep3**, as well as reaction conversions determined by HPLC-UV for **p$_4$-Pep4** to **p$_6$-Pep4**, **p$_4$-Pep5** to **p$_6$-Pep5**, **p$_4$-Pep6** to **p$_6$-Pep6**, and **p$_4$-Pep7** to **p$_6$-Pep7** are summarized in Supporting Table 7. Characterization data for all isolated peptides by analytical HPLC and HRMS as described in SI section 1 is summarized in SI section 6.

Supporting Table 7: Yields and reaction conversions for deprotected peptide tetra-, penta-, and hexaphosphates.

Entry	Compound	Sequence	% Yield
1	**p$_4$-Pep1**	AcHN-WNA**[ppppT]**ANG-CONH$_2$	13 (95[a])
2	**p$_5$-Pep1**	AcHN-WNA**[pppppT]**ANG-CONH$_2$	11 (76[a])
3	**p$_6$-Pep1**	AcHN-WNA**[ppppppT]**ANG-CONH$_2$	8 (40[a])
4	**p$_4$-Pep2**	AcHN-WNA**[ppppS]**ANG-CONH$_2$	14 (98[a])
5	**p$_5$-Pep2**	AcHN-WNA**[pppppS]**ANG-CONH$_2$	n.d.[c]
6	**p$_6$-Pep2**	AcHN-WNA**[ppppppS]**ANG-CONH$_2$	n.d.[c]
7	**p$_4$-Pep3**	H$_2$N-VMLGE**[ppppT]**NPADSKPGTIR-COOH	17 (96[a])
8	**p$_5$-Pep3**	H$_2$N-VMLGE**[pppppT]**NPADSKPGTIR-COOH	14 (97[a])
9	**p$_6$-Pep3**	H$_2$N-VMLGE**[ppppppT]**NPADSKPGTIR-COOH	16 (90[a])
10	**p$_4$-Pep4**	H$_2$N-SPTP**[ppppS]**PSPPRNSDQEGGGK-COOH	85[a]

11	**p_5-Pep4**	H_2N-SPTP**[pppppS]**PSPPRNSDQEGGGK-COOH	80[a]
12	**p_6-Pep4**	H_2N-SPTP**[ppppppS]**PSPPRNSDQEGGGK-COOH	81[a]
13	**p_4-Pep5**	H_2N-KESE**[ppppS]**EDSSDDEPLIK-COOH	95[a]
14	**p_5-Pep5**	H_2N-KESE**[pppppS]**EDSSDDEPLIK-COOH	93[a]
15	**p_6-Pep5**	H_2N-KESE**[ppppppS]**EDSSDDEPLIK-COOH	91[a]
16	**p_4-Pep6**	H_2N-VEEDAE**[ppppS]**EDEEEEDVK-COOH	41[a]
17	**p_5-Pep6**	H_2N-VEEDAE**[pppppS]**EDEEEEDVK-COOH	39[a]
18	**p_6-Pep6**	H_2N-VEEDAE**[ppppppS]**EDEEEEDVK-COOH	63[a]
19	**p_4-Pep7**	H_2N-KKAS**[ppppS]**SDSEDSSEEE-COOH	67[a]
20	**p_5-Pep7**	H_2N-KKAS**[pppppS]**SDSEDSSEEE-COOH	81[a]
21	**p_6-Pep7**	H_2N-KKAS**[ppppppS]**SDSEDSSEEE-COOH	92[a]

[a]Conversion after 10 min determined by analytical HPLC. [b]product not observed. [c]not determined.

5.2.3 Synthesis of Benzyl-protected Triphosphopeptides

To generate triphosphopeptides, benzyl-derivatized diphosphorylation reagent **2a** was used followed by palladium-catalyzed deprotection of the benzyl protecting group in a one-pot approach.

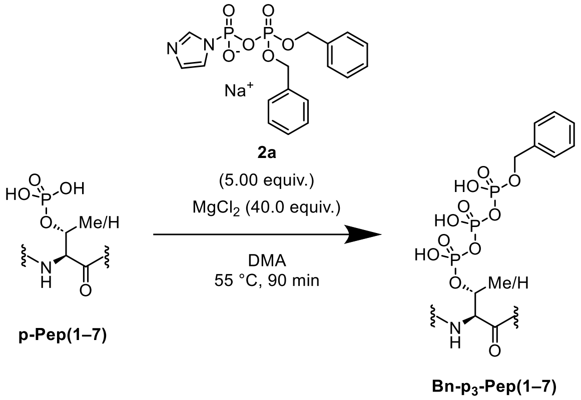

Figure S58: General reaction scheme for the diphosphorylation of phosphopeptides **p-Pep1** to **p-Pep7** with benzyl-derivatized diphosphorylation reagent **2a**.

General procedure 5:

Phosphopeptide (4.00 mg, 1.00 equiv.) was dissolved in DMA (1.50 mL) with the assistance of sonication.[a] In parallel, reagent **2a** (5.00 equiv.) and magnesium chloride (40.0 equiv.) were suspended in DMA (1.50 mL) and dispersed with the assistance of sonication. Both solutions were combined and the resulting cloudy colorless reaction mixture was shaken at 55 °C for 90 min. The reaction mixture was used in the next step without further manipulation.

Reaction conversions determined by HPLC-UV for **Bn-p$_3$-Pep1** to **Bn-p$_3$-Pep7** are summarized in Supporting Table 8. Characterization data for all peptides by HRMS as described in SI section 1 is summarized in SI section 6.

[a]For **p-Pep7** only, Milli-Q water (150 µL) was added due to solubility issues to reach a final DMA/H_2O ratio of approx. 95:5.

Supporting Table 8: Reaction conversions for benzyl-protected peptide triphosphates synthesized with reagent **2a**.

Entry	Compound	Sequence	% Conversion[a]
1	**Bn-p$_3$-Pep1**	AcHN-WNA**[Bn-pppT]**ANG-CONH$_2$	98
2	**Bn-p$_3$-Pep2**	AcHN-WNA**[Bn-pppS]**ANG-CONH$_2$	n.d.[c]
3	**Bn-p$_3$-Pep3**	H$_2$N-VMLGE**[Bn-pppT]**NPADSKPGTIR-COOH	97
4	**Bn-p$_3$-Pep4**	H$_2$N-SPTP**[Bn-pppS]**PSPPRNSDQEGGGK-COOH	79
5	**Bn-p$_3$-Pep5**	H$_2$N-KESE**[Bn-pppS]**EDSSDDEPLIK-COOH	97
6	**Bn-p$_3$-Pep6**	H$_2$N-VEEDAE**[Bn-pppS]**EDEEEEDVK-COOH	93
7	**Bn-p$_3$-Pep7**	H$_2$N-KKAS**[Bn-pppS]**SDSEDSSEEE-COOH	96

[a]Conversion after 90 min determined by analytical HPLC. [c]not determined.

5.2.4 Debenzylation of Triphosphopeptides

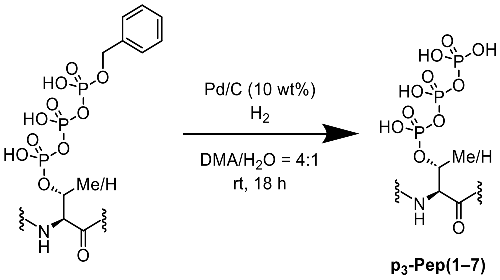

Figure S59: General reaction scheme for the hydrogenation of benzyl-protected triphosphopeptides **Bn-p$_3$-Pep1** to **Bn-p$_3$-Pep7**.

__General procedure 6:__
The reaction mixture containing benzyl-protected triphosphopeptide was diluted with Milli-Q water (750 µL) and transferred to a 25-mL Schlenk flask equipped with a rubber septum and stir bar. After the addition of palladium (10 wt% on carbon, dry, 10 mg), the reaction mixture was carefully evacuated under vacuum and backfilled with nitrogen three times, leaving the flask under vacuum. Subsequently, a balloon filled with hydrogen gas was connected through the septum to the flask by using a needle and the resulting black reaction mixture was stirred under hydrogen at room temperature for 18 h. Afterward, the reaction mixture was filtered through a short Celite plug. Therefore, a 3-mL glass pipette was equipped with a small cotton plug and filled with Celite® 503 to reach an approx. height of 5 cm dry powder inside of the pipette. The black reaction mixture was added from the top of the pipette and the filtrate was collected in a 15-mL Falcon tube. A pipette hat was used to accelerate the filtration process. The collected filtrate was diluted with a Milli-Q water/ acetonitrile mixture (9:1 + 0.1% formic acid, 10.0 mL), filtered through a syringe filter (RC 0.45 µm), and submitted to preparative HPLC (**Method I**). After lyophilization of the product containing fractions, the respective free triphosphopeptide was obtained as a white solid.

Yields determined by weight for **p$_3$-Pep1** to **p$_3$-Pep3** as well as reaction conversions determined by HPLC-UV for **p$_3$-Pep4** to **p$_3$-Pep7** are summarized in Supporting Table 9. Characterization data for all isolated peptides by analytical HPLC and HRMS as described in SI section 1 is summarized in SI section 6.

Supporting Table 9: Yields and reaction conversions for deprotected peptide triphosphates.

Entry	Compound	Sequence	% Yield
1	**p$_3$-Pep1**	AcHN-WNA**[pppT]**ANG-CONH$_2$	17 (94[a])
2	**p$_3$-Pep2**	AcHN-WNA**[pppS]**ANG-CONH$_2$	n.d.[c]
3	**p$_3$-Pep3**	H$_2$N-VMLGE**[pppT]**NPADSKPGTIR-COOH	11 (79[a])
4	**p$_3$-Pep4**	H$_2$N-SPTP**[pppS]**PSPPRNSDQEGGGK-COOH	82
5	**p$_3$-Pep5**	H$_2$N-KESE**[pppS]**EDSSDDEPLIK-COOH	80
6	**p$_3$-Pep6**	H$_2$N-VEEDAE**[pppS]**EDEEEEDVK-COOH	86
7	**p$_3$-Pep7**	H$_2$N-KKAS**[pppS]**SDSEDSSEEE-COOH	91

[a]Conversion after 18 h determined by analytical HPLC. [b]product not observed. [c]not determined.

6 Peptide HPLC-UV Traces and Mass Spectrometry Characterization

6.1 pThr Model Peptides

p-Pep1
AcHN-Trp-Asn-Ala-[pThr]-Ala-Asn-Gly-CONH$_2$

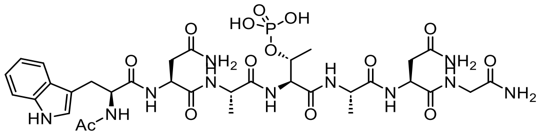

HRMS (ESI): m/z calc'd. for $C_{33}H_{47}N_{11}O_{14}P^-$ $[M\text{-}H]^-$ 852.3047, found 852.3012.
T_R (Method **A**) = 7.330 min.

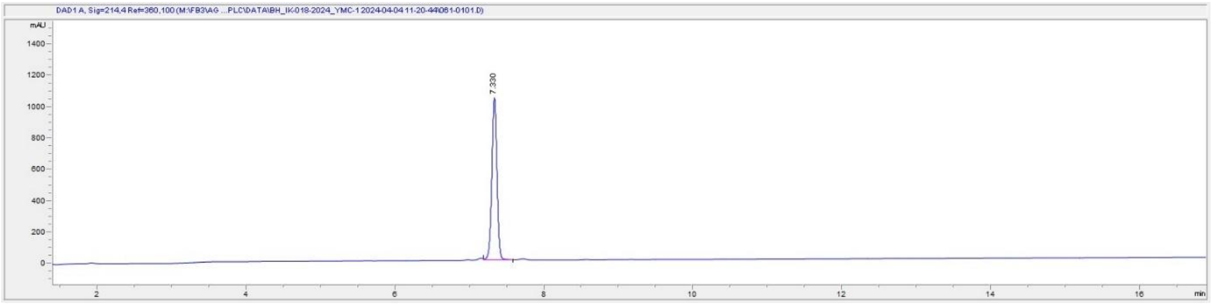

Figure S60: HPLC-UV trace (214 nm) of purified **p-Pep1**.

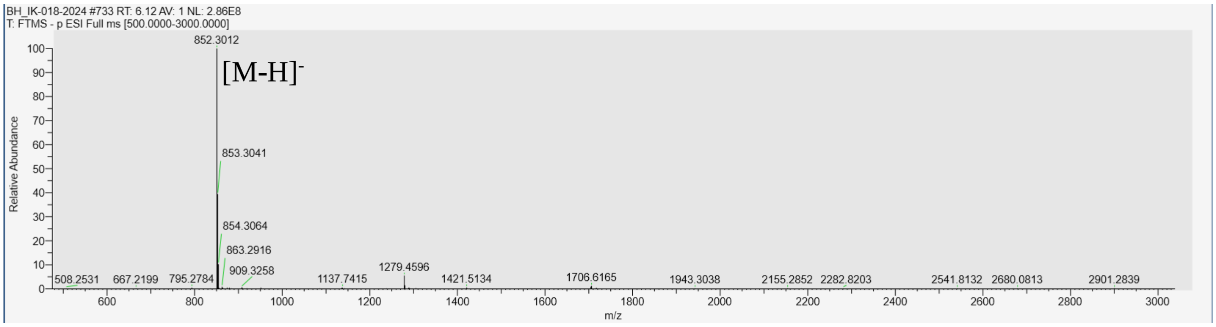

Figure S61: HRMS spectrum of **p-Pep1**.

NPE-p$_2$-Pep1
AcHN-Trp-Asn-Ala-[NPE-ppThr]-Ala-Asn-Gly-CONH$_2$

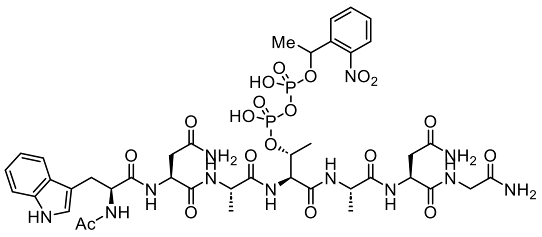

HRMS (ESI): m/z calc'd. for $C_{41}H_{55}N_{12}O_{19}P_2^-$ [M-H]$^-$ 1081.3187, found 1081.3296.
T_R (Method **A**) = 11.808 min.

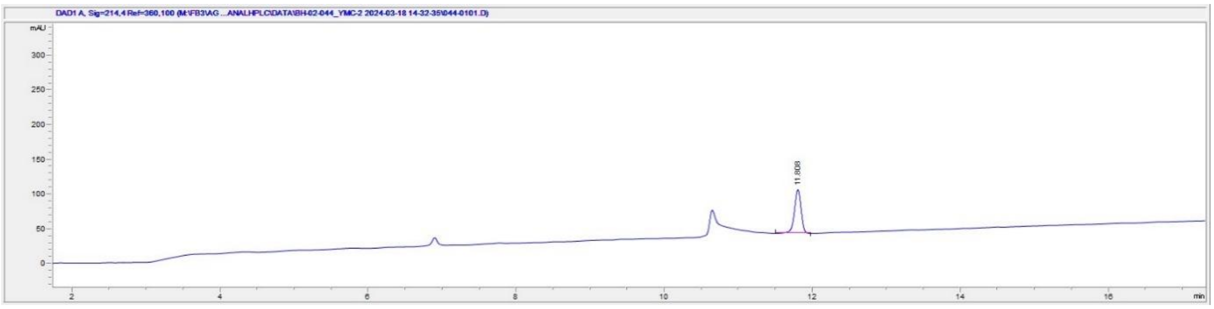

Figure S62: HPLC-UV trace (214 nm) of purified **NPE-p$_2$-Pep1**.

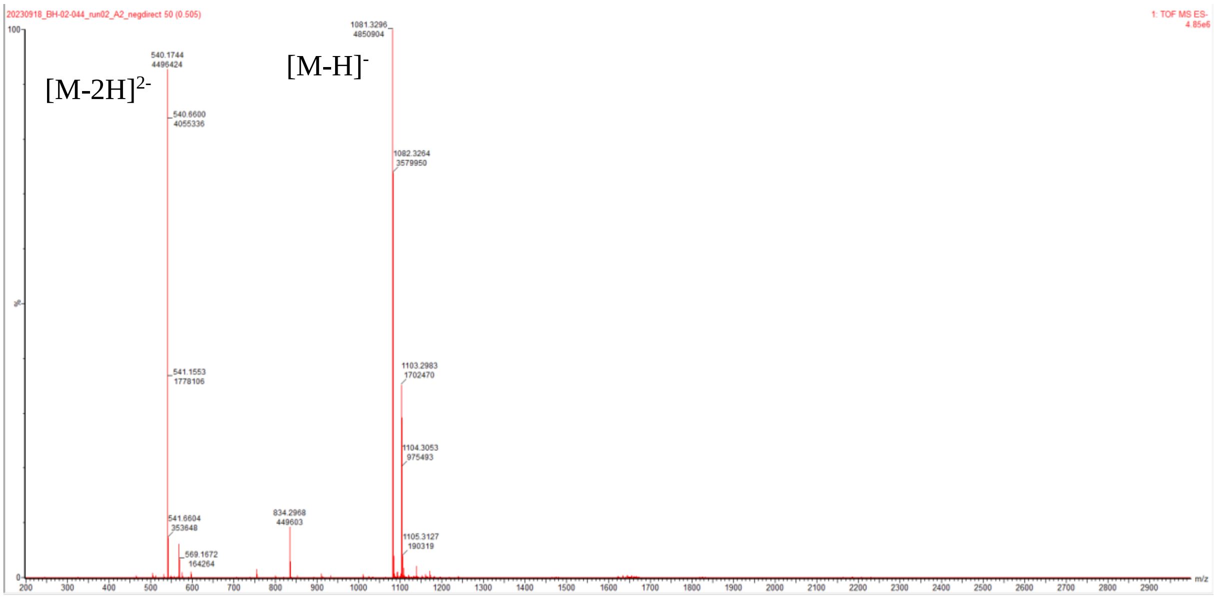

Figure S63: HRMS spectrum of **NPE-p$_2$-Pep1**.

p_2-Pep1
AcHN-Trp-Asn-Ala-[ppThr]-Ala-Asn-Gly-$CONH_2$

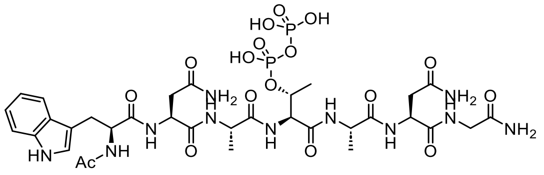

HRMS (ESI): m/z calc'd. for $C_{33}H_{48}N_{11}O_{17}P_2^-$ $[M-H]^-$ 932.2710, found 932.2700.
T_R (Method **A**) = 7.770 min.

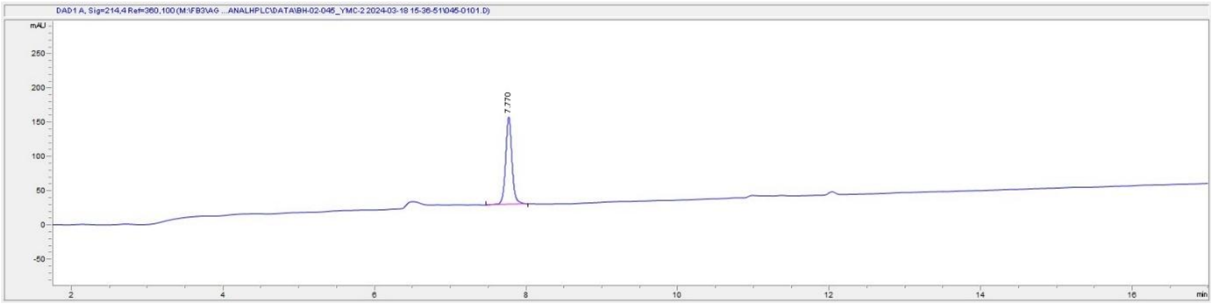

Figure S64: HPLC-UV trace (214 nm) of purified **p_2-Pep1**.

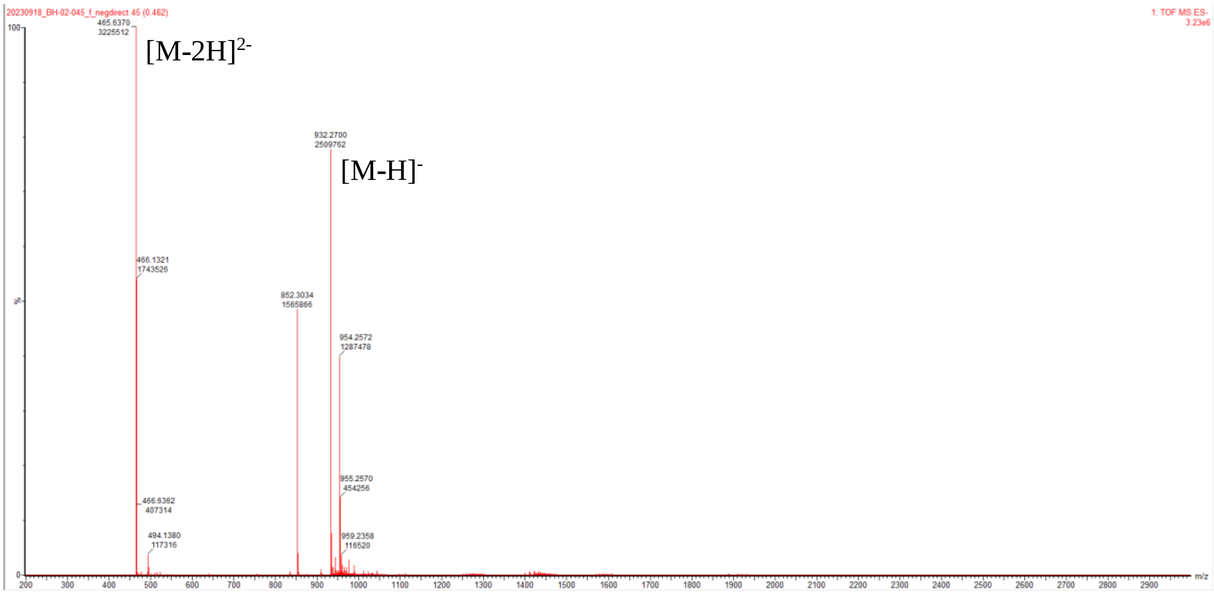

Figure S65: HRMS spectrum of **p_2-Pep1**.

Bn_2-p_3-Pep1

AcHN-Trp-Asn-Ala-[Bn-pppThr]-Ala-Asn-Gly-$CONH_2$

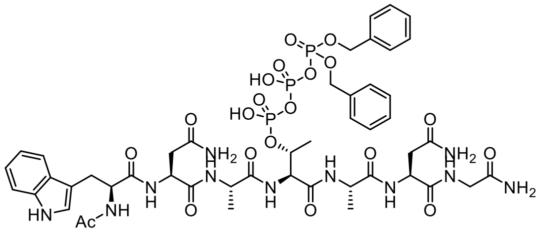

HRMS (ESI): m/z calc'd. for $C_{47}H_{61}N_{11}O_{20}P_3^-$ [M-H]$^-$ 1192.3312, found 1192.3494

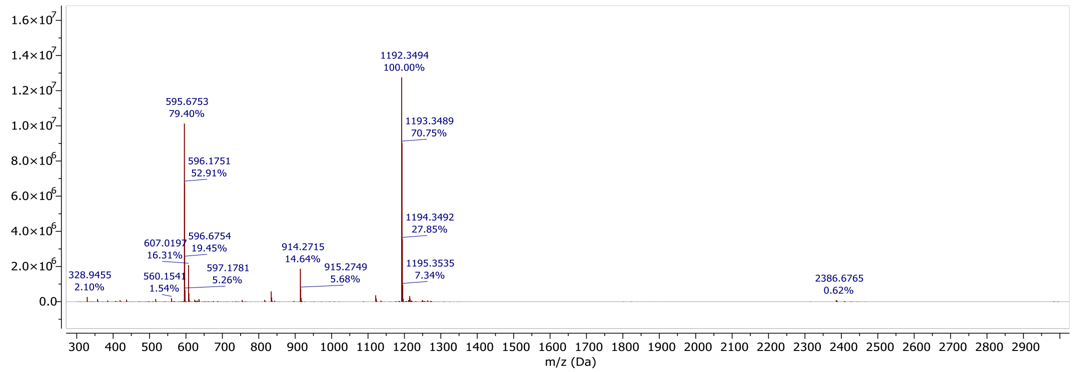

Figure S66A: HRMS spectrum of **Bn_2-p_3-Pep1**.

Bn-p_3-Pep1

AcHN-Trp-Asn-Ala-[Bn-pppThr]-Ala-Asn-Gly-$CONH_2$

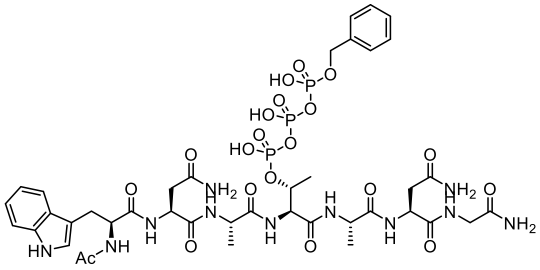

HRMS (ESI): m/z calc'd. for $C_{40}H_{55}N_{11}O_{20}P_3^-$ [M-H]$^-$ 1102.2843, found 1102.3009

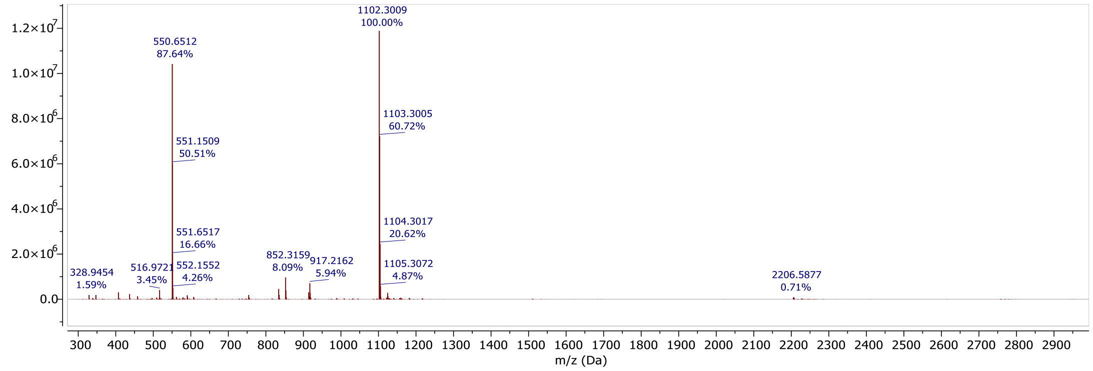

Figure S66B: HRMS spectrum of **Bn-p_3-Pep1**.

p_3-Pep1

AcHN-Trp-Asn-Ala-[pppThr]-Ala-Asn-Gly-$CONH_2$

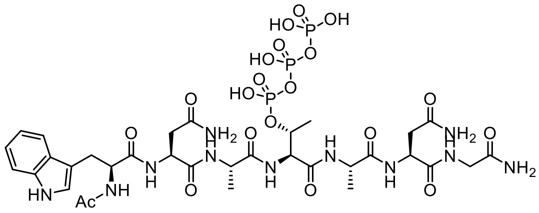

HRMS (ESI): m/z calc'd. for $C_{33}H_{49}N_{11}O_{20}P_3^-$ $[M-H]^-$ 1012.2373, found 1012.2296

T_R (Method **A**) = 7.259 min.

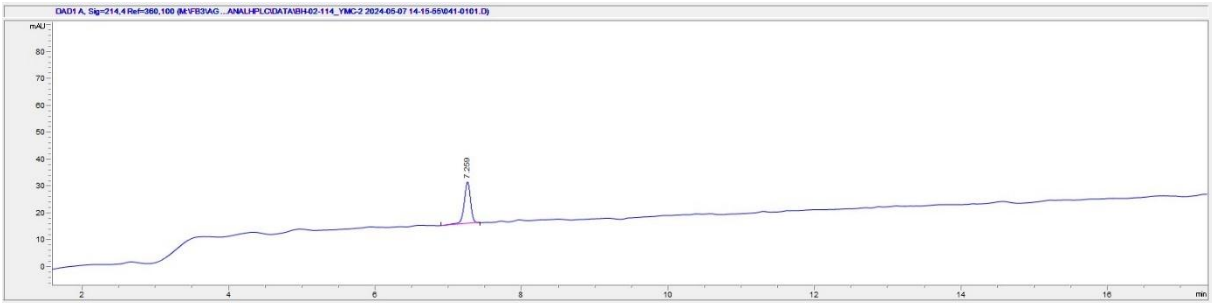

Figure S67: HPLC-UV trace (214 nm) of purified **p_3-Pep1**.

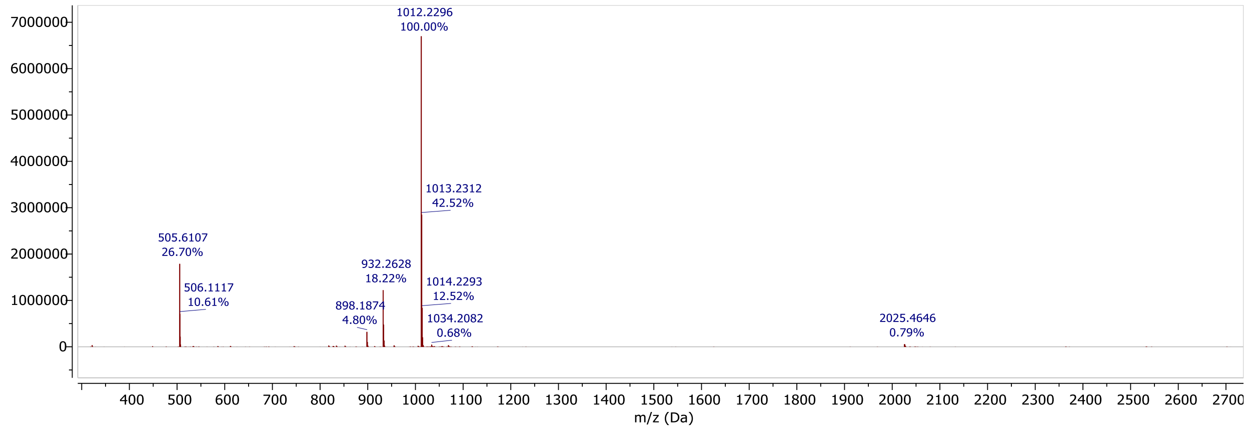

Figure S68: HRMS spectrum of **p_3-Pep1**.

NPE-p$_4$-Pep1
AcHN-Trp-Asn-Ala-[NPE-ppppThr]-Ala-Asn-Gly-CONH$_2$

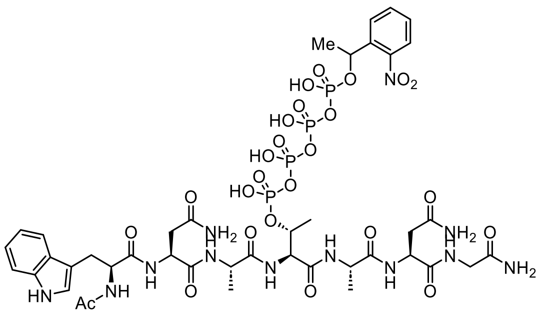

HRMS (ESI): m/z calc'd. for $C_{41}H_{54}AlN_{12}O_{25}P_4^-$ $[M-4H+Al]^-$ 1265.2094, found 1265.2087.
T_R (Method **A**) = 10.622 min.

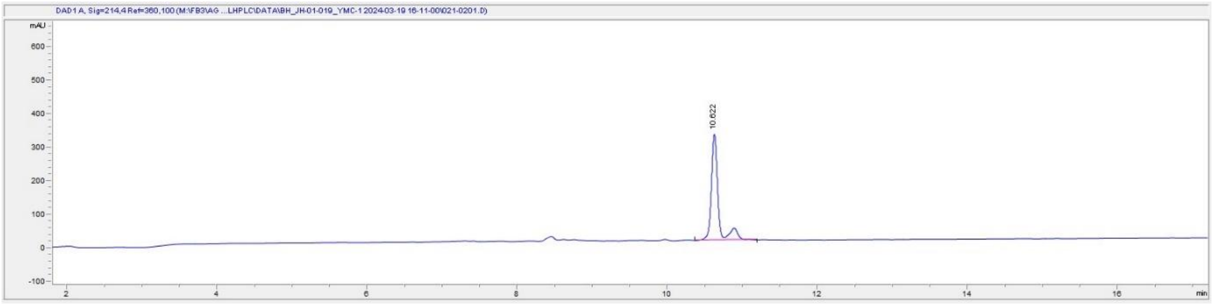

Figure S69: HPLC-UV trace (214 nm) of purified **NPE-p$_4$-Pep1**.

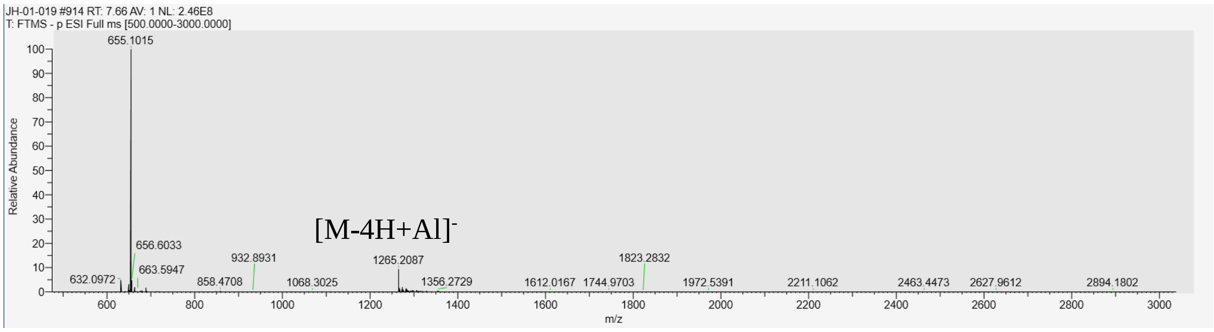

Figure S70: HRMS spectrum of **NPE-p$_4$-Pep1**.

p$_4$-Pep1
AcHN-Trp-Asn-Ala-[ppppThr]-Ala-Asn-Gly-CONH$_2$

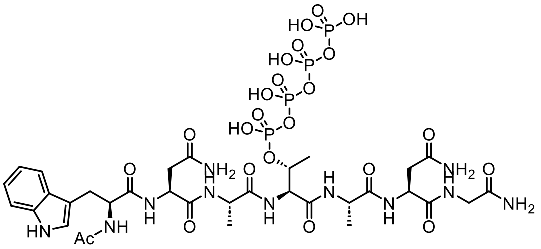

HRMS (ESI): m/z calc'd. for $C_{33}H_{47}AlN_{11}O_{23}P_4^-$ [M-4H+Al]$^-$ 1116.1617, found 1116.1643.
T_R (Method **A**) = 7.280 min.

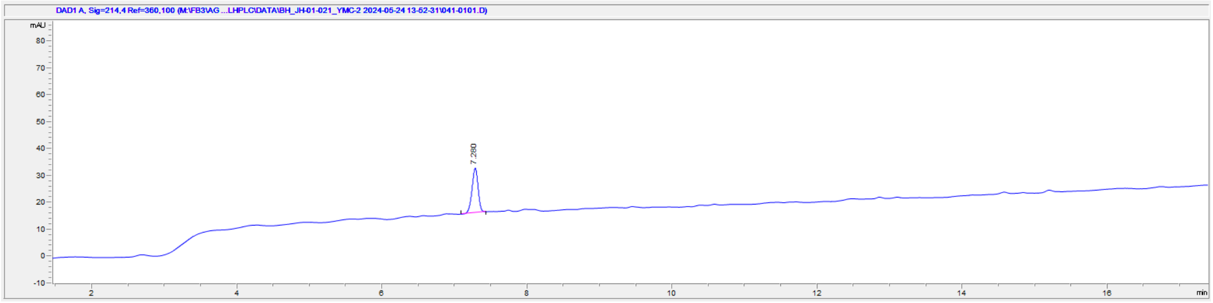

Figure S71: HPLC-UV trace (214 nm) of purified **p$_4$-Pep1**.

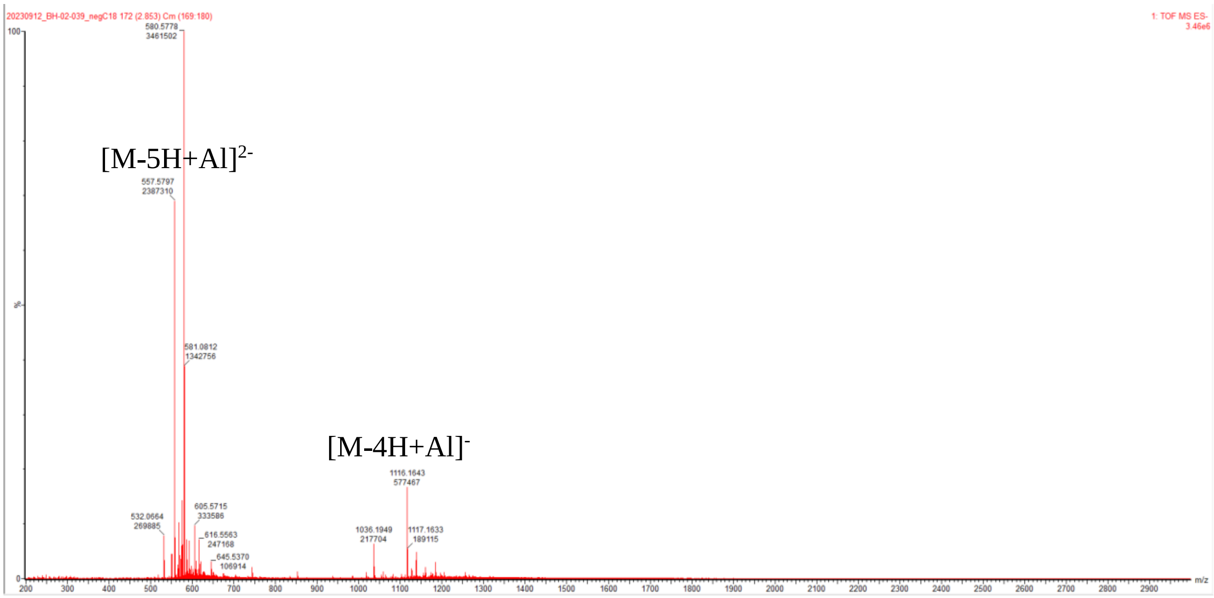

Figure S72: HRMS spectrum of **p$_4$-Pep1**.

Bn-p5-Pep1

AcHN-Trp-Asn-Ala-[Bn-pppppThr]-Ala-Asn-Gly-$CONH_2$

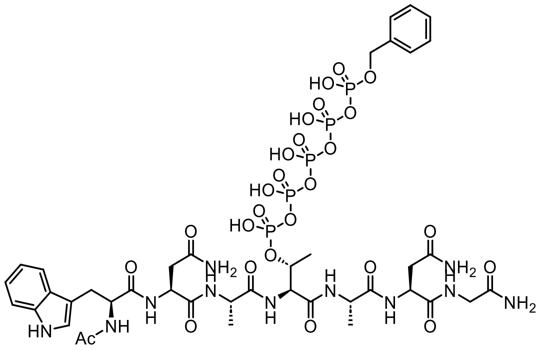

HRMS (ESI): m/z calc'd. for $C_{40}H_{53}AlN_{11}O_{26}P_5^{2-}$ $[M-5H+Al]^{2-}$ 642.5839, found 642.5780.

T_R (Method **B**) = 7.818 min; T_R (Method **X,** see Section 7.1) = 6.727 min.

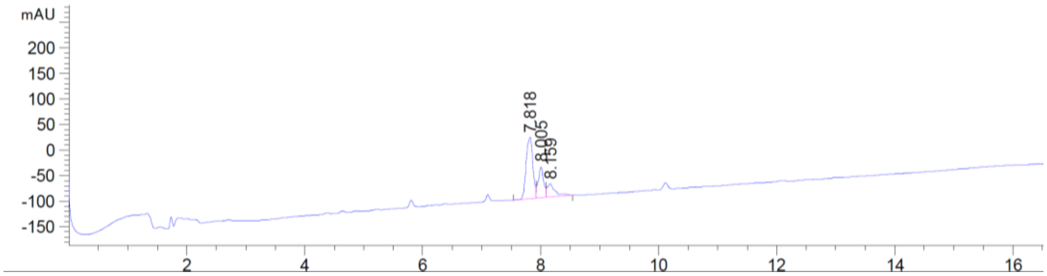

Figure S73: HPLC-UV trace (214 nm) of purified **Bn-p5-Pep1**.

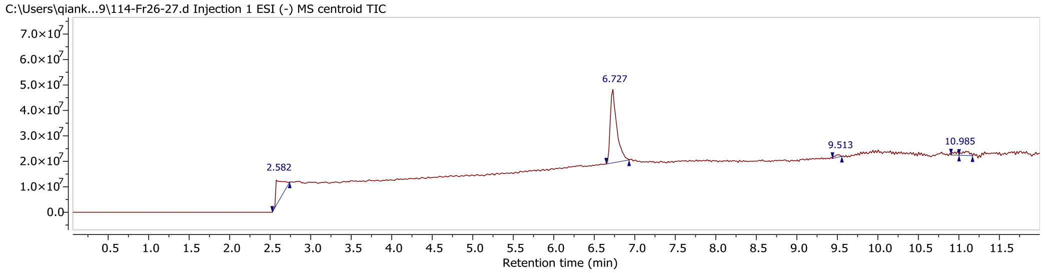

Figure S74: Total-ion-chromatogram (TIC) of purified **Bn-p5-Pep1**.

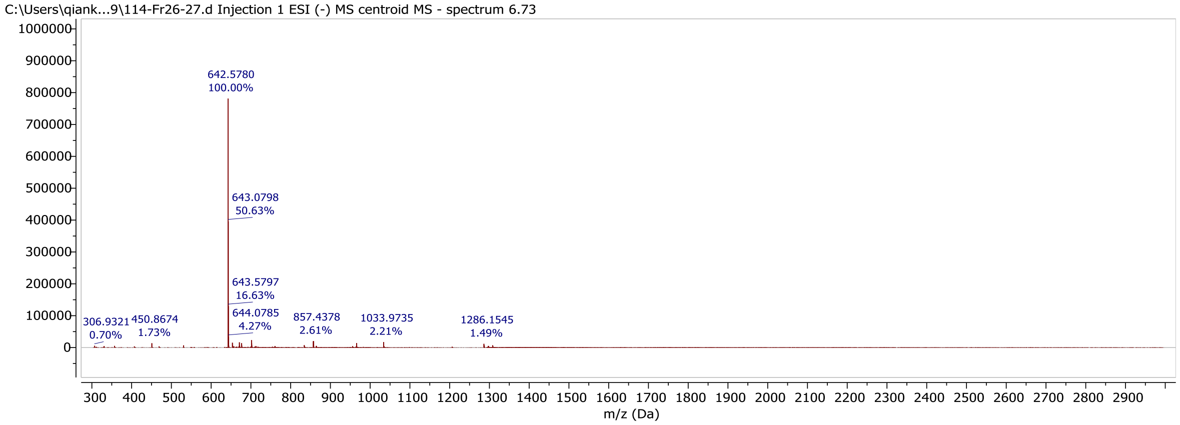

Figure S75: HRMS spectrum of **Bn-p5-Pep1**.

NPE-p5-Pep1

AcHN-Trp-Asn-Ala-[NPE-pppppThr]-Ala-Asn-Gly-$CONH_2$

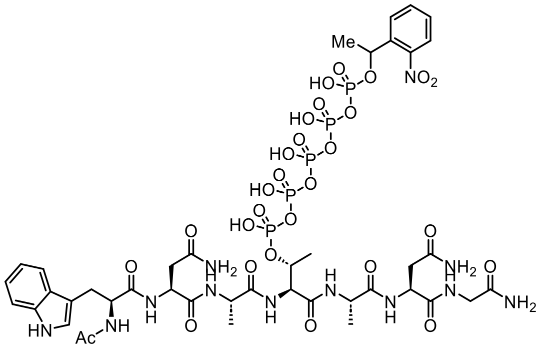

HRMS (ESI): m/z calc'd. for $C_{41}H_{54}AlN_{12}O_{28}P_5^{2-}$ $[M-5H+Al]^{2-}$ 672.0842, found 672.0793.

T_R (Method **A**) = 9.400 min (Al), 9.545 min (Fe).

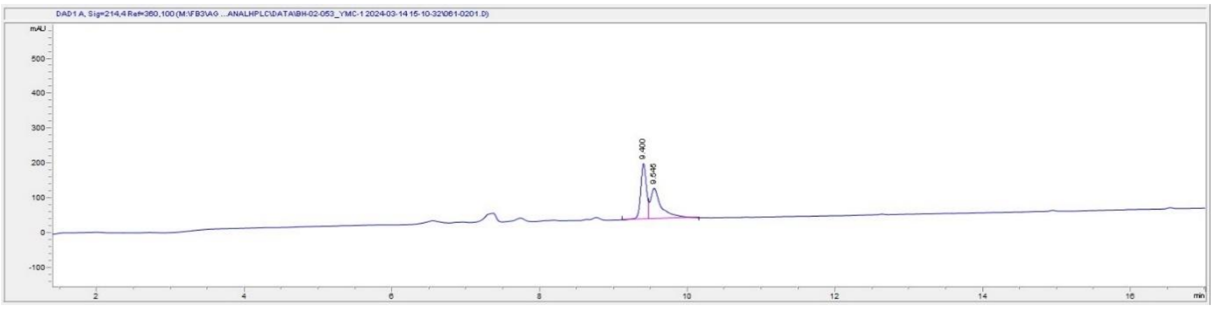

Figure S76: HPLC-UV trace (214 nm) of purified **NPE-p5-Pep1**.

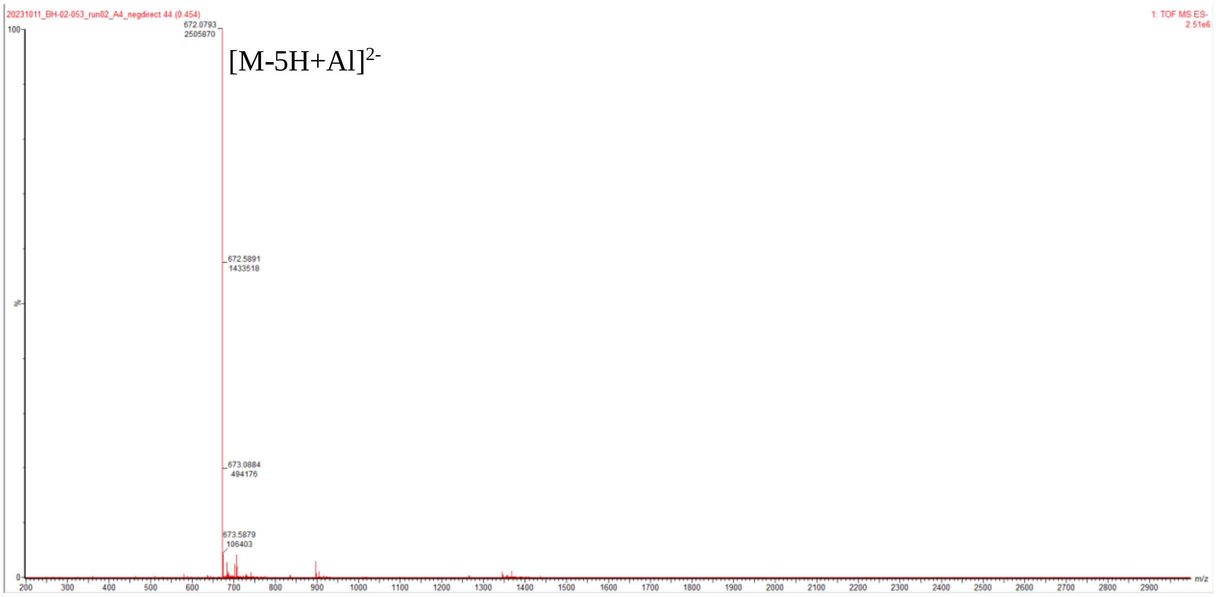

Figure S77: HRMS spectrum of **NPE-p5-Pep1**.

p_5-Pep1

AcHN-Trp-Asn-Ala-[pppppThr]-Ala-Asn-Gly-$CONH_2$

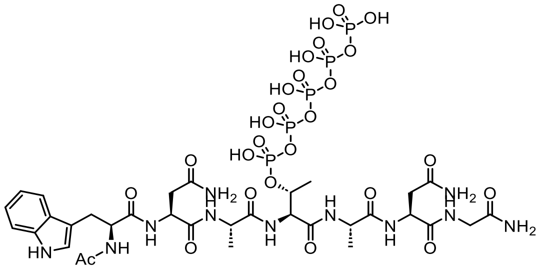

HRMS (ESI): m/z calc'd. for $C_{33}H_{47}AlN_{11}O_{26}P_5^{2-}$ $[M-5H+Al]^{2-}$ 597.5604, found 597.5614.

T_R (Method **A**) = 4.811 min.

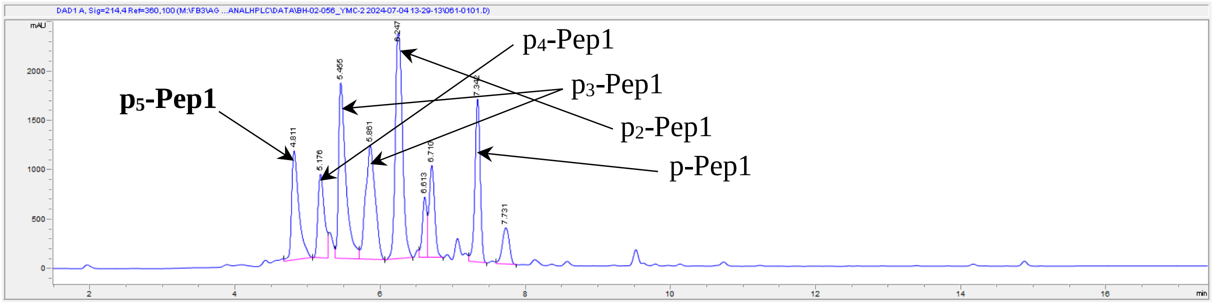

Figure S78: HPLC-UV trace (214 nm) of **p_5-Pep1** obtained after removal of DMA by preparative HPLC. Indicated degradation products identified by LC-MS. Similar stability issues observed for **p_6-Pep1**.

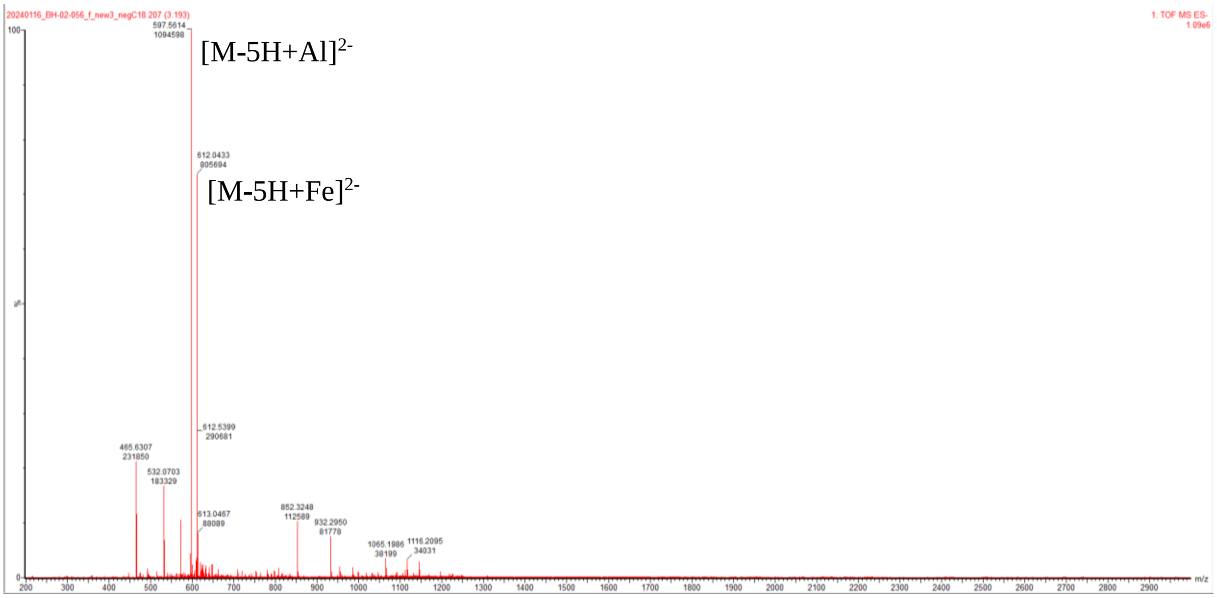

Figure S79: HRMS spectrum of **p_5-Pep1**.

NPE-p$_6$-Pep1

AcHN-Trp-Asn-Ala-[NPE-ppppppThr]-Ala-Asn-Gly-$CONH_2$

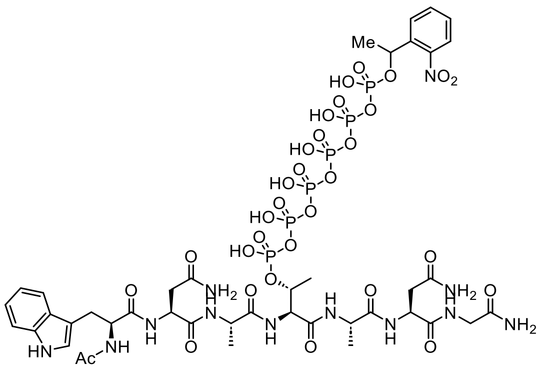

HRMS (ESI): m/z calc'd. for $C_{41}H_{55}AlN_{12}O_{31}P_6^{2-}$ $[M-5H+Al]^{2-}$ 712.0674, found 712.0651.

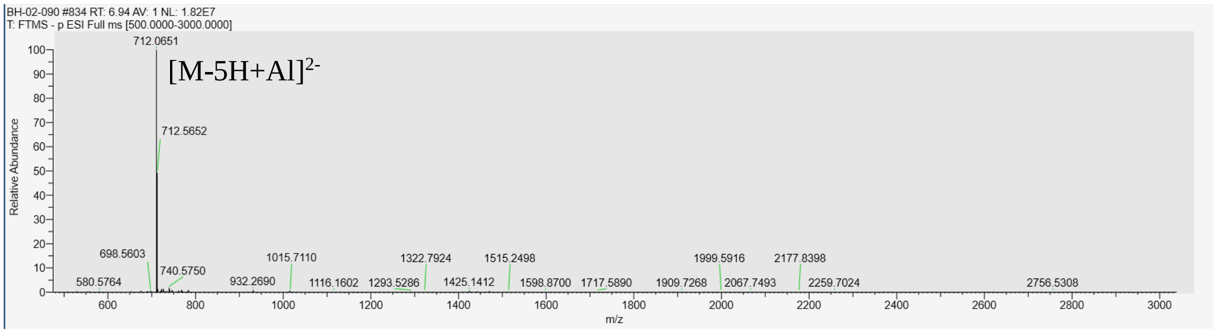

Figure S80: HRMS spectrum of **NPE-p$_6$-Pep1**.

p$_6$-Pep1

AcHN-Trp-Asn-Ala-[ppppppThr]-Ala-Asn-Gly-CONH$_2$

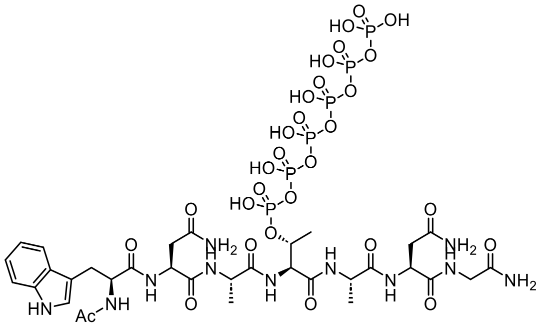

HRMS (ESI): m/z calc'd. for $C_{33}H_{48}AlN_{11}O_{29}P_6^{2-}$ [M-5H+Al]$^{2-}$ 637.5436, found 637.5416.

Similar stability issues compared to **p$_5$-Pep1** (Fig. S77) observed for **p$_6$-Pep1**, leading to the partial degradation of the oligophosphoryl chain.

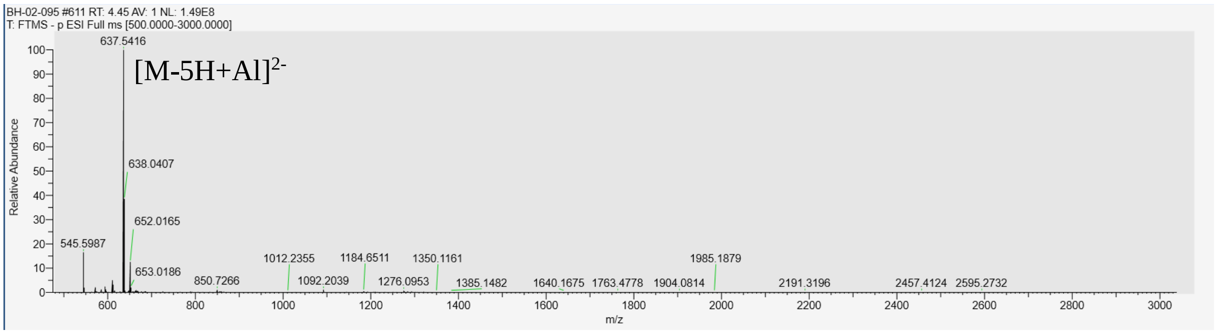

Figure S81: HRMS spectrum of **p$_6$-Pep1**.

6.2 pSer Model Peptides

p-Pep2

AcHN-Trp-Asn-Ala-[pSer]-Ala-Asn-Gly-CONH$_2$

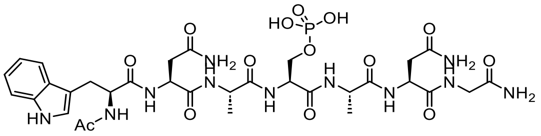

HRMS (ESI): m/z calc'd. for $C_{32}H_{45}N_{11}O_{14}P^-$ $[M-H]^-$ 838.2890, found 838.2865.
T_R (Method **A**) = 7.083 min.

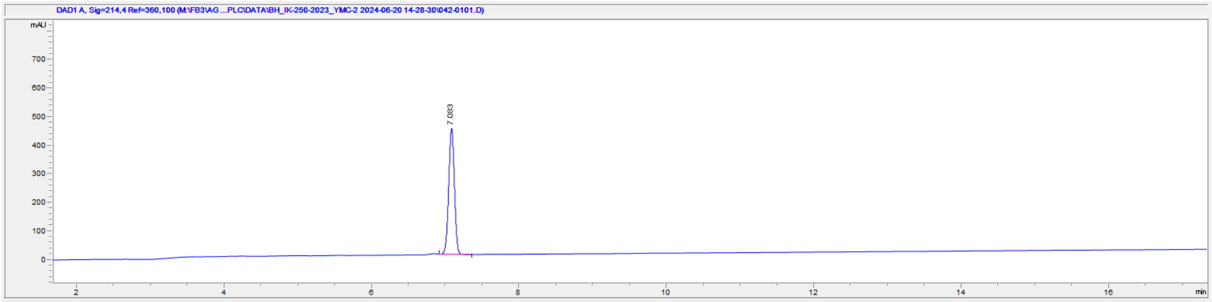

Figure S82: HPLC-UV trace (214 nm) of purified **p-Pep2**.

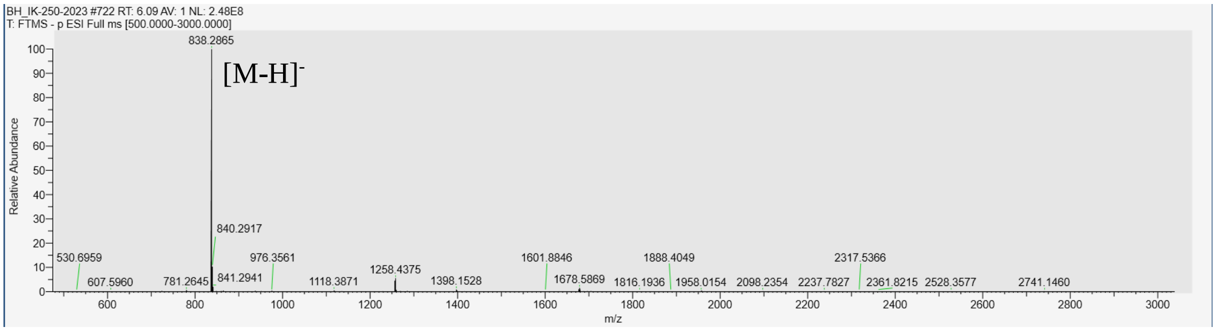

Figure S83: HRMS spectrum of **p-Pep2**.

NPE-p2-Pep2

AcHN-Trp-Asn-Ala-[NPE-ppSer]-Ala-Asn-Gly-$CONH_2$

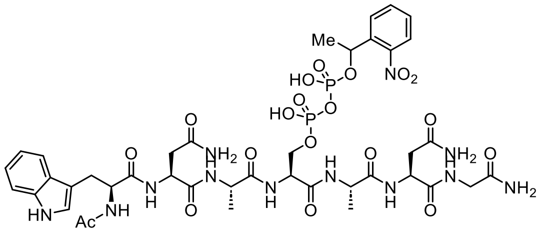

HRMS (ESI): m/z calc'd. for $C_{40}H_{53}N_{12}O_{19}P_2^-$ $[M-H]^-$ 1067.3030, found 1067.3027.
T_R (Method **A**) = 10.060 min.

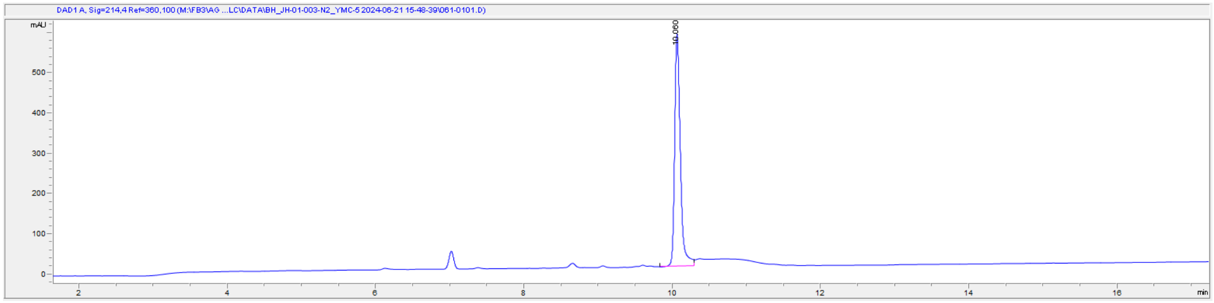

Figure S84: HPLC-UV trace (214 nm) of purified **NPE-p2-Pep2**.

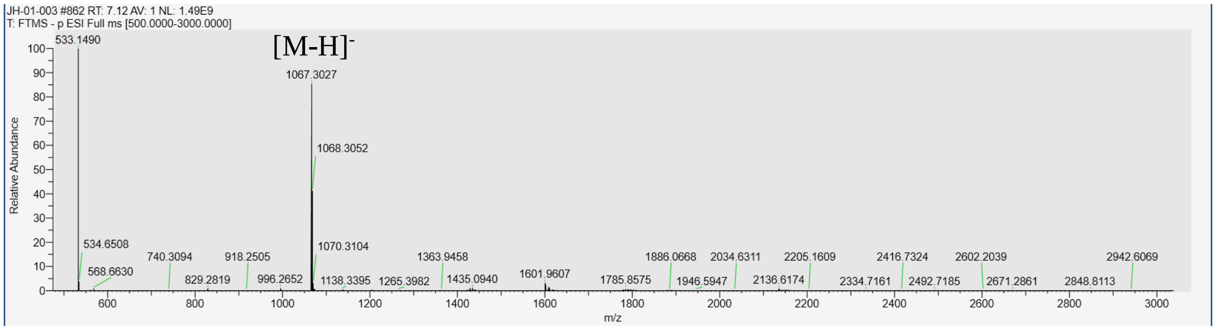

Figure S85: HRMS spectrum of **NPE-p2-Pep2**.

p_2-Pep2

AcHN-Trp-Asn-Ala-[ppSer]-Ala-Asn-Gly-$CONH_2$

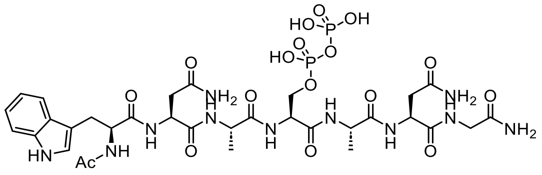

HRMS (ESI): m/z calc'd. for $C_{32}H_{46}N_{11}O_{17}P_2^-$ $[M-H]^-$ 918.2553, found 918.2538.

T_R (Method **A**) = 7.620 min.

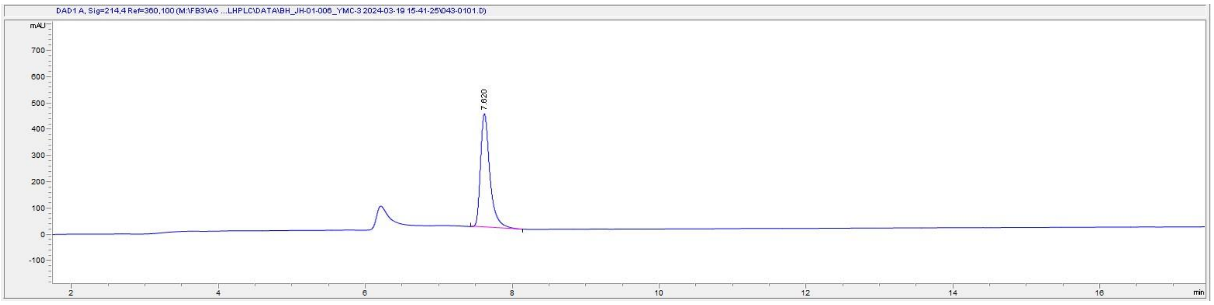

Figure S86: HPLC-UV trace (214 nm) of purified **p_2-Pep2**.

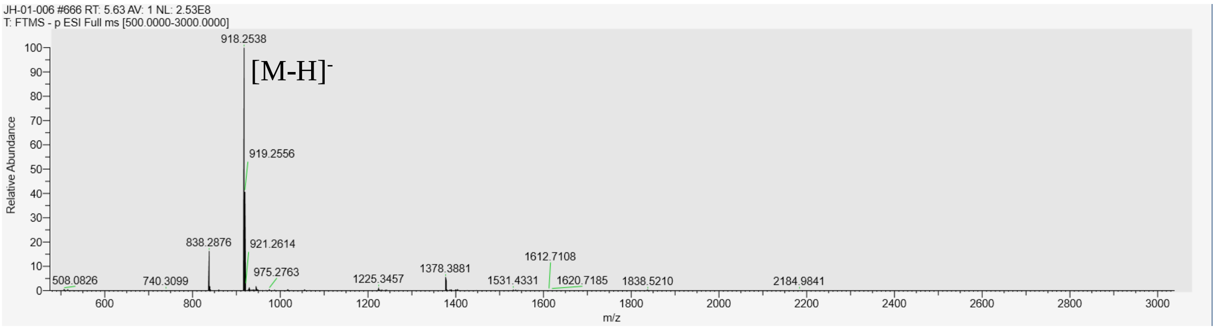

Figure S87: HRMS spectrum of **p_2-Pep2**.

NPE-p$_4$-Pep2
AcHN-Trp-Asn-Ala-[NPE-ppppSer]-Ala-Asn-Gly-CONH$_2$

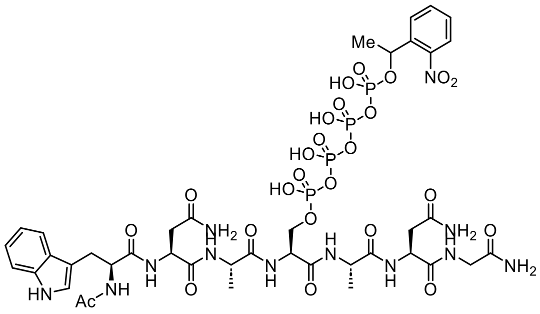

HRMS (ESI): m/z calc'd. for $C_{40}H_{52}AlN_{12}O_{25}P_4^-$ [M-4H+Al]$^-$ 1251.1937, found 1251.1860.
T_R (Method **A**) = 10.506 min.

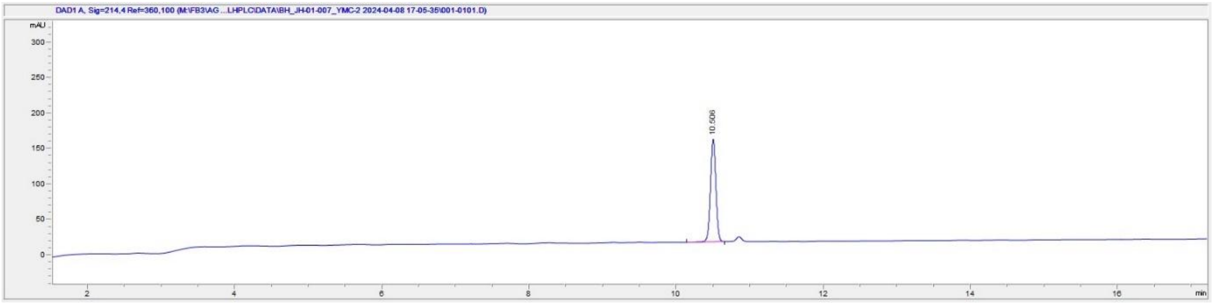

Figure S88: HPLC-UV trace (214 nm) of purified **NPE-p$_4$-Pep2**.

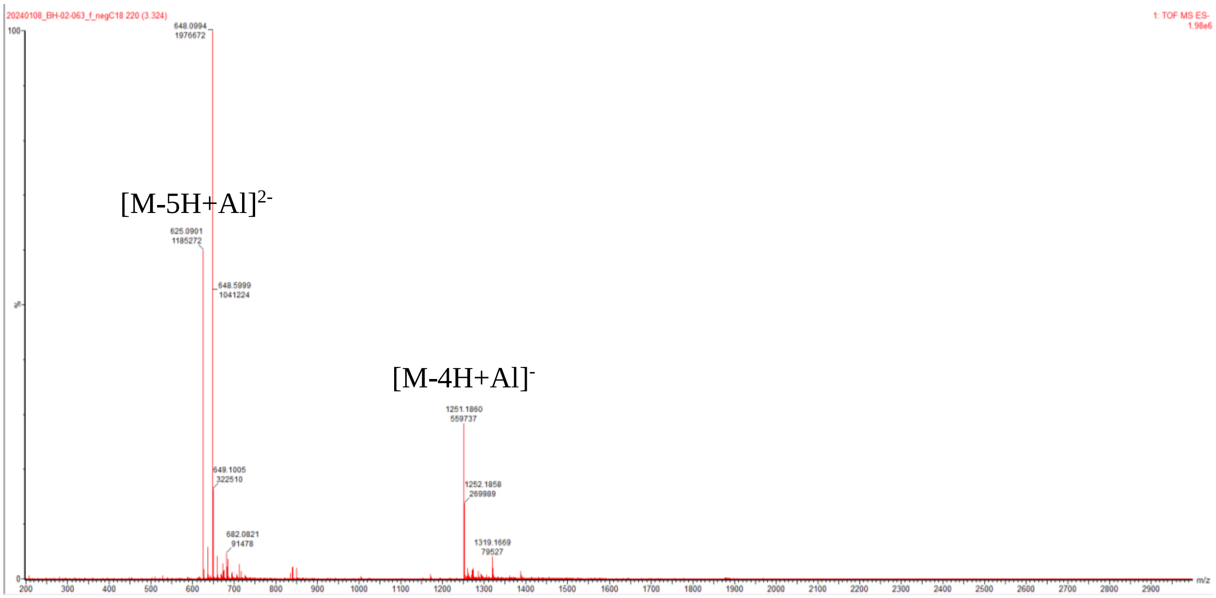

Figure S89: HRMS spectrum of **NPE-p$_4$-Pep2**.

p_4-Pep2
AcHN-Trp-Asn-Ala-[ppppSer]-Ala-Asn-Gly-$CONH_2$

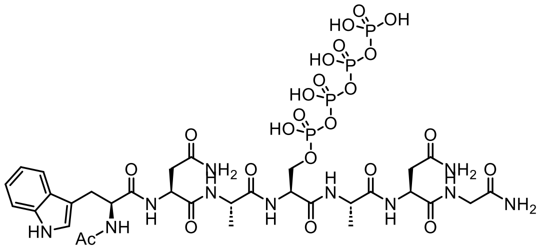

HRMS (ESI): m/z calc'd. for $C_{32}H_{45}AlN_{11}O_{23}P_4^-$ $[M-4H+Al]^-$ 1102.1461, found 1102.1346.
T_R (Method **A**) = 7.088 min.

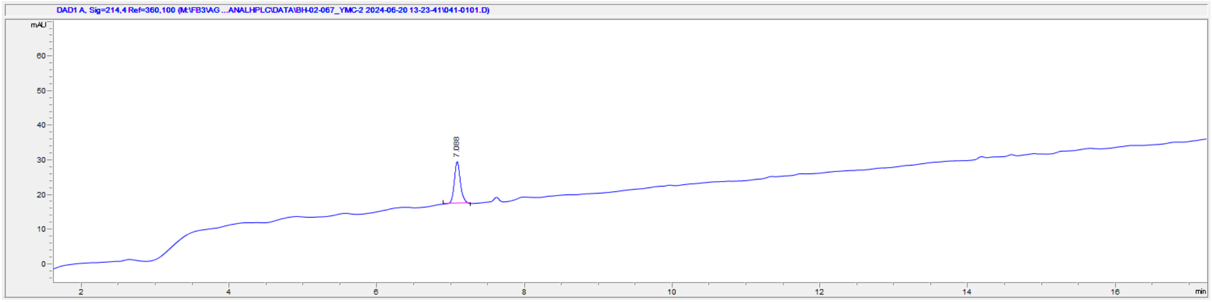

Figure S90: HPLC-UV trace (214 nm) of purified **p_4-Pep2**.

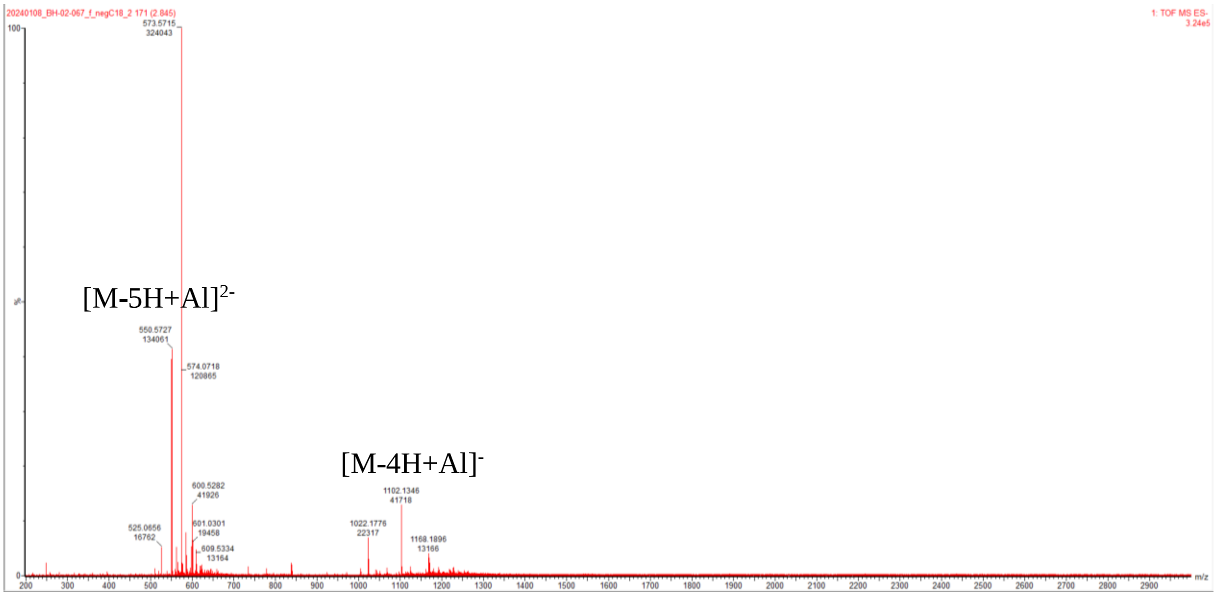

Figure S91: HRMS spectrum of **p_4-Pep2**.

6.3 NME1 (Thr94) Peptides

p-Pep3

H_2N-Val-Met-Leu-Gly-Glu-[pThr]-Asn-Pro-Ala-Asp-Ser-Lys-Pro-Gly-Thr-Ile-Arg-COOH

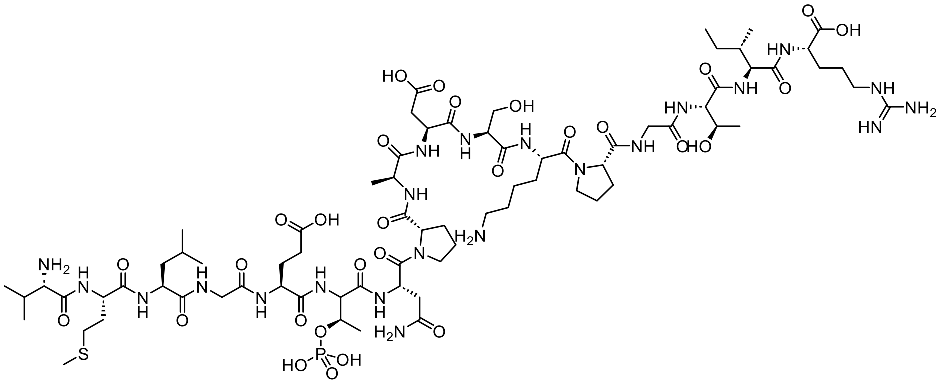

HRMS (ESI): m/z calc'd. for $C_{75}H_{127}N_{22}O_{29}PS^{2-}$ $[M-2H]^{2-}$ 931.4304, found 931.4323.
T_R (Method **A**) = 9.780 min.

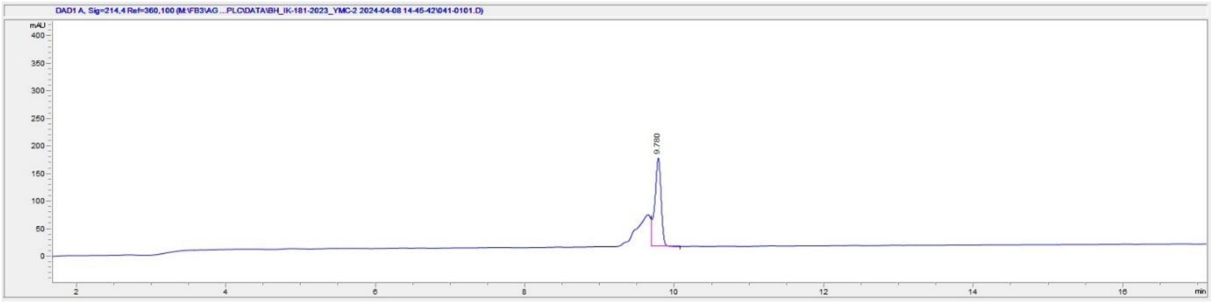

Figure S92: HPLC-UV trace (214 nm) of purified **p-Pep3**.

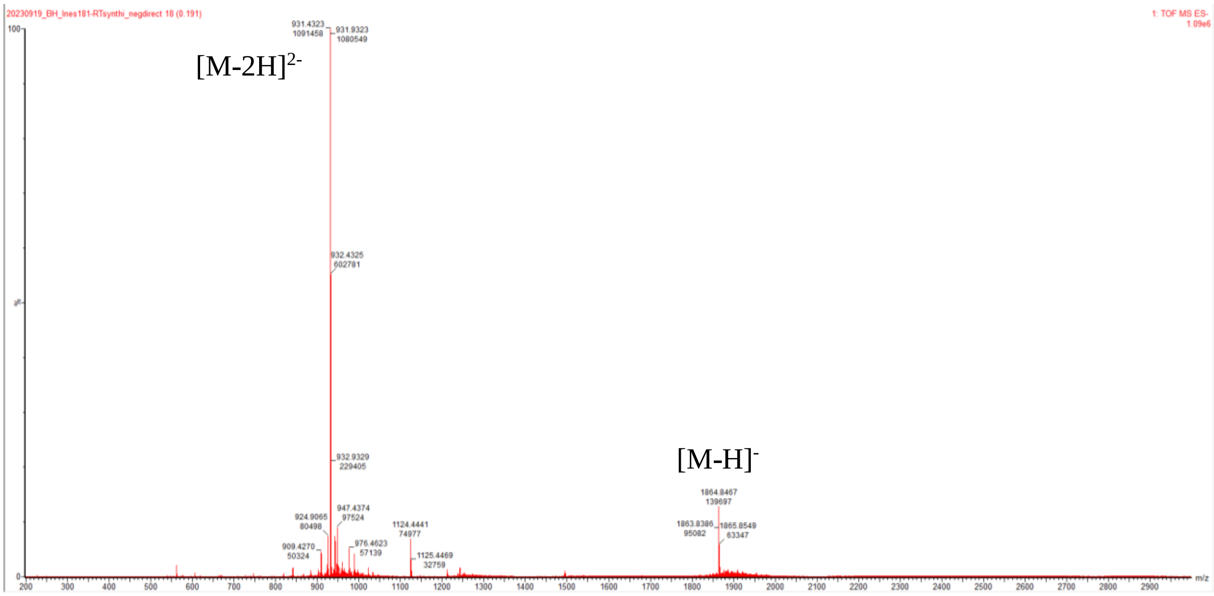

Figure S93: HRMS spectrum of **p-Pep3**.

NPE-p$_2$-Pep3

H_2N-Val-Met-Leu-Gly-Glu-[NPE-ppThr]-Asn-Pro-Ala-Asp-Ser-Lys-Pro-Gly-Thr-Ile-Arg-COOH

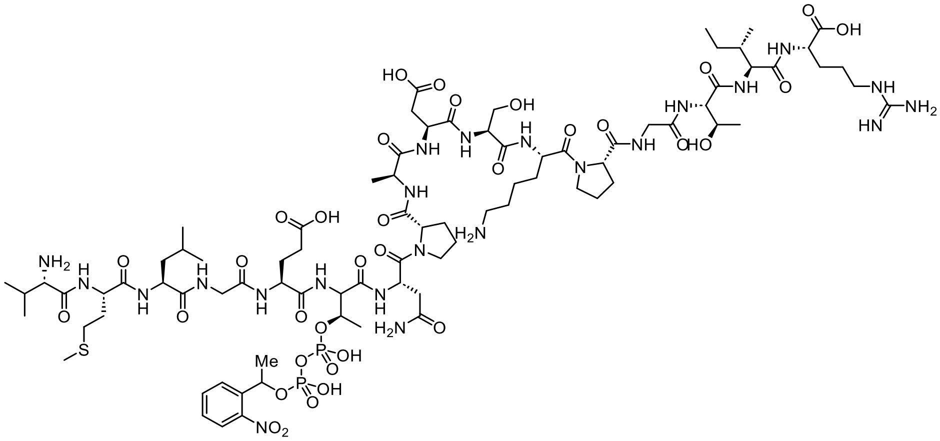

HRMS (ESI): m/z calc'd. for $C_{83}H_{135}N_{23}O_{34}P_2S^{2-}$ $[M-2H]^{2-}$ 1045.9374, found 1045.9369.
T_R (Method **A**) = 12.121 min.

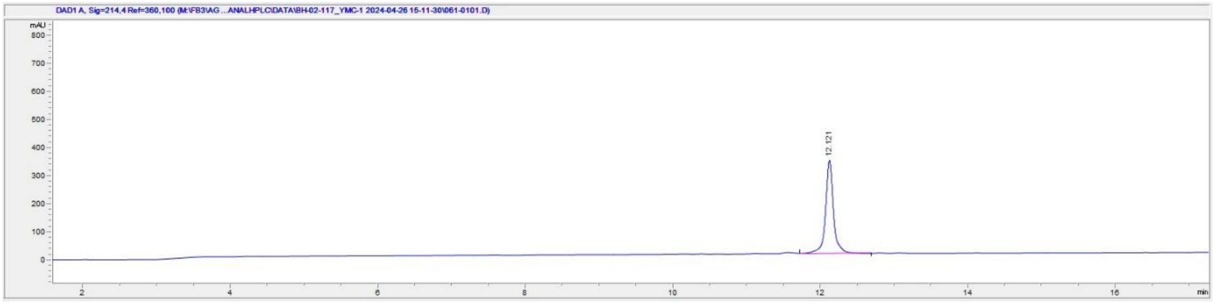

Figure S94: HPLC-UV trace (214 nm) of purified **NPE-p$_2$-Pep3**.

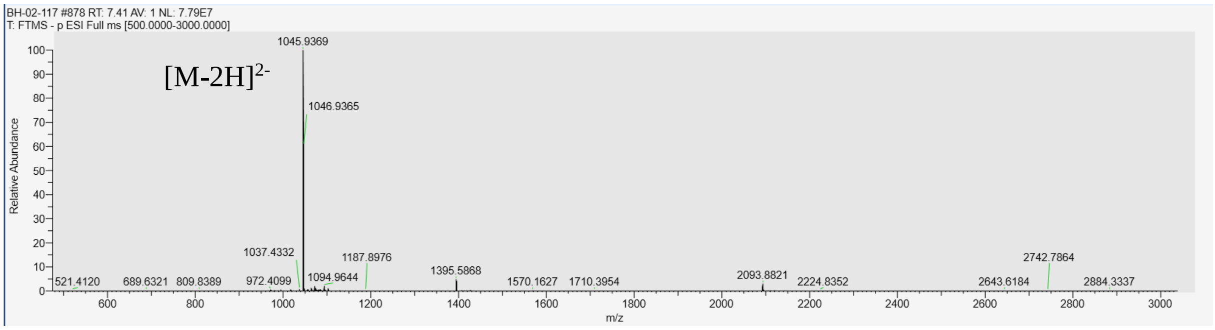

Figure S95: HRMS spectrum of **NPE-p$_2$-Pep3**.

p$_2$-Pep3
H_2N-Val-Met-Leu-Gly-Glu-[ppThr]-Asn-Pro-Ala-Asp-Ser-Lys-Pro-Gly-Thr-Ile-Arg-COOH

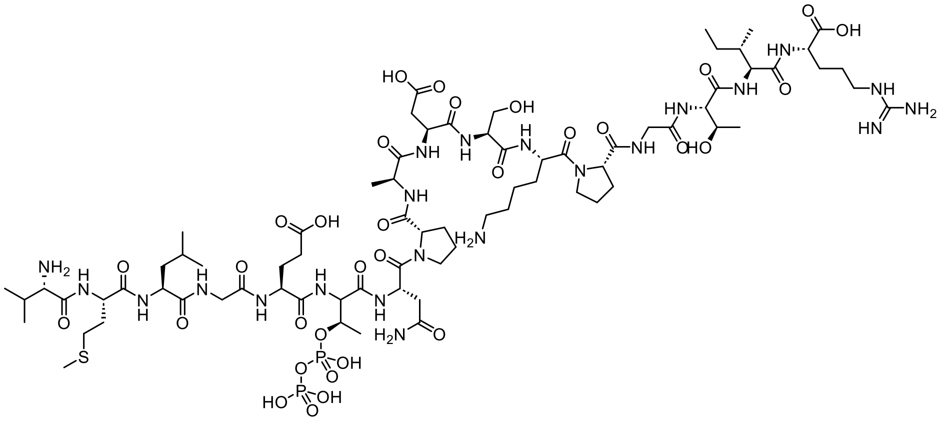

HRMS (ESI): m/z calc'd. for $C_{75}H_{128}N_{22}O_{32}P_2S^{2-}$ $[M-2H]^{2-}$ 971.4136, found 971.4125.
T_R (Method **A**) = 9.625 min.

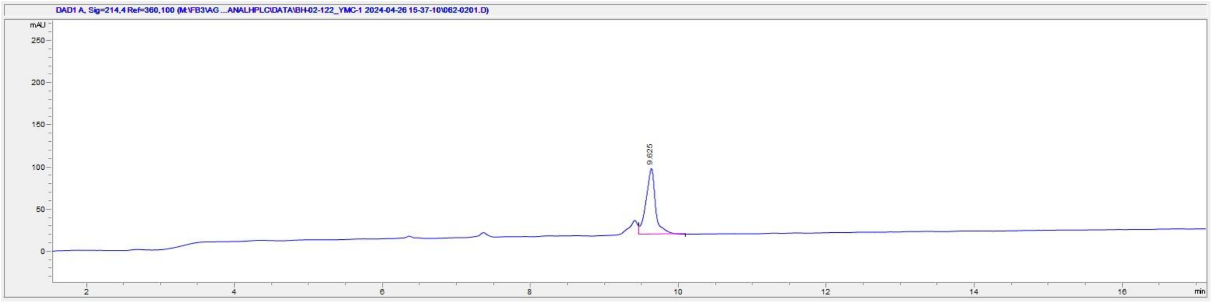

Figure S96: HPLC-UV trace (214 nm) of purified **p$_2$-Pep3**.

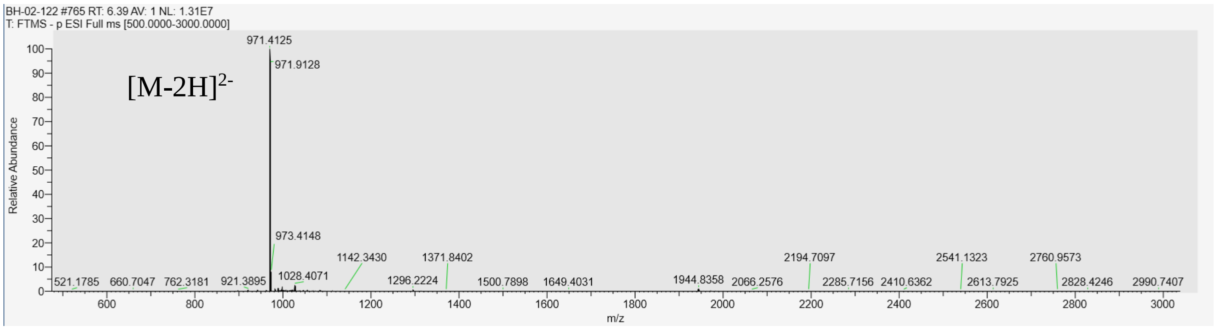

Figure S97: HRMS spectrum of **p$_2$-Pep3**.

Bn-p$_3$-Pep3

H$_2$N-Val-Met-Leu-Gly-Glu-[Bn-pppThr]-Asn-Pro-Ala-Asp-Ser-Lys-Pro-Gly-Thr-Ile-Arg-COOH

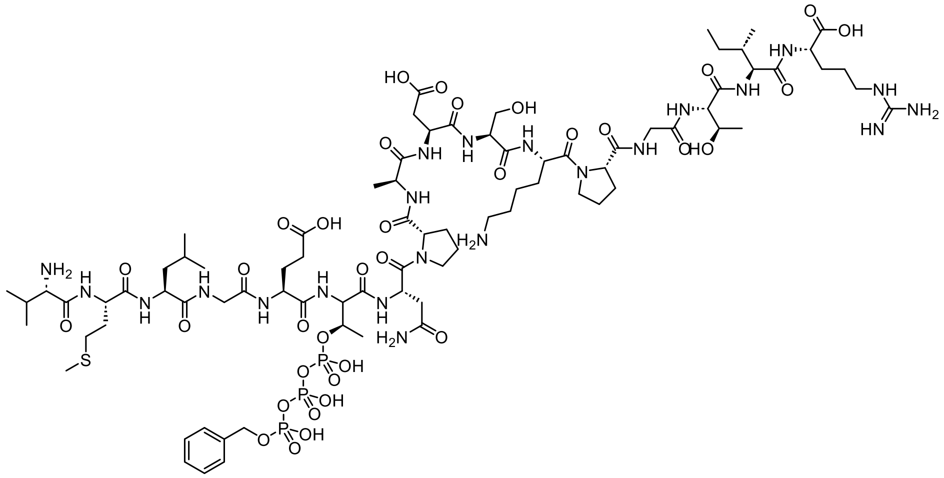

HRMS (ESI): m/z calc'd. for $C_{82}H_{135}N_{22}O_{35}P_3S^{2-}$ $[M-2H]^{2-}$ 1056.4202, found 1056.4177.

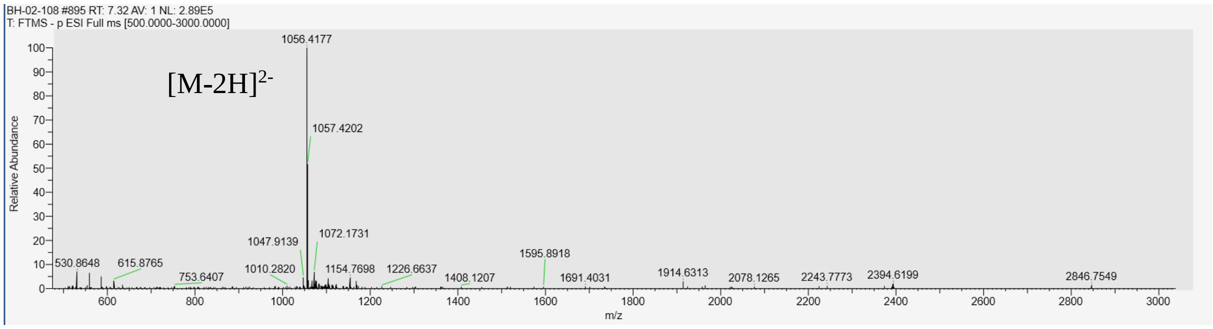

Figure S98: HRMS spectrum of **Bn-p$_3$-Pep3**.

p_3-Pep3

H_2N-Val-Met-Leu-Gly-Glu-[pppThr]-Asn-Pro-Ala-Asp-Ser-Lys-Pro-Gly-Thr-Ile-Arg-COOH

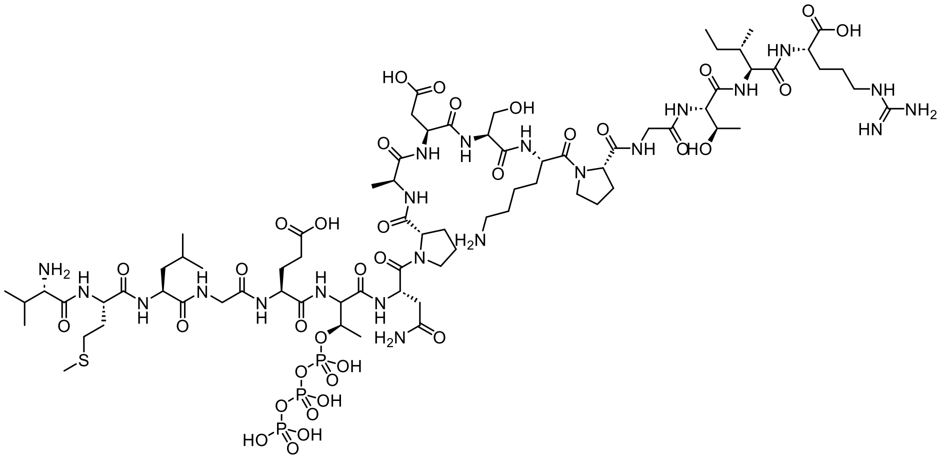

HRMS (ESI): m/z calc'd. for $C_{75}H_{126}AlN_{22}O_{35}P_3S^{2-}$ $[M-5H+Al]^{2-}$ 1023.3758, found 1023.3763.
T_R (Method **A**) = 9.435 min.

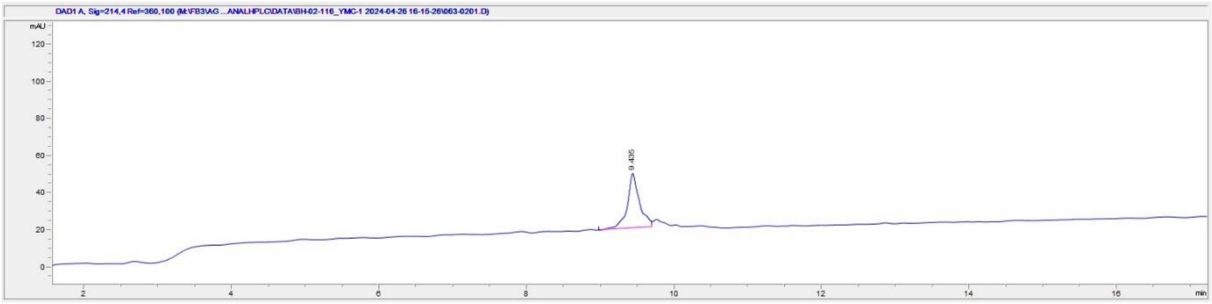

Figure S99: HPLC-UV trace (214 nm) of purified **p_3-Pep3**.

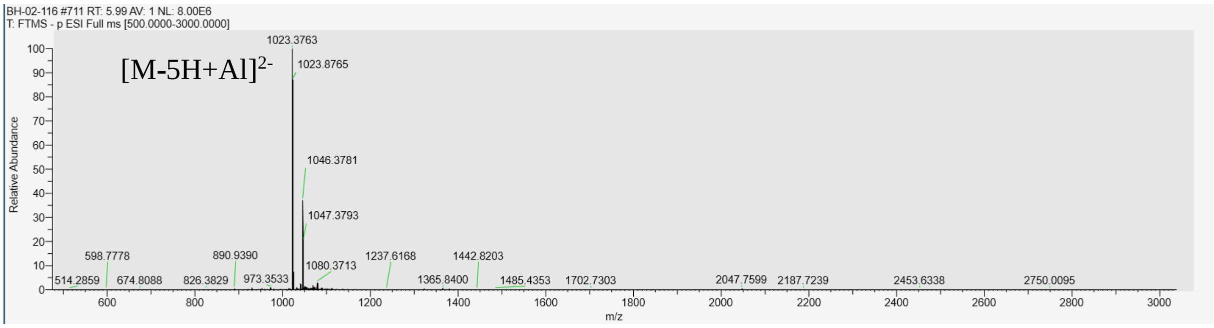

Figure S100: HRMS spectrum of **p_3-Pep3**.

NPE-p4-Pep3

H_2N-Val-Met-Leu-Gly-Glu-[NPE-ppppThr]-Asn-Pro-Ala-Asp-Ser-Lys-Pro-Gly-Thr-Ile-Arg-COOH

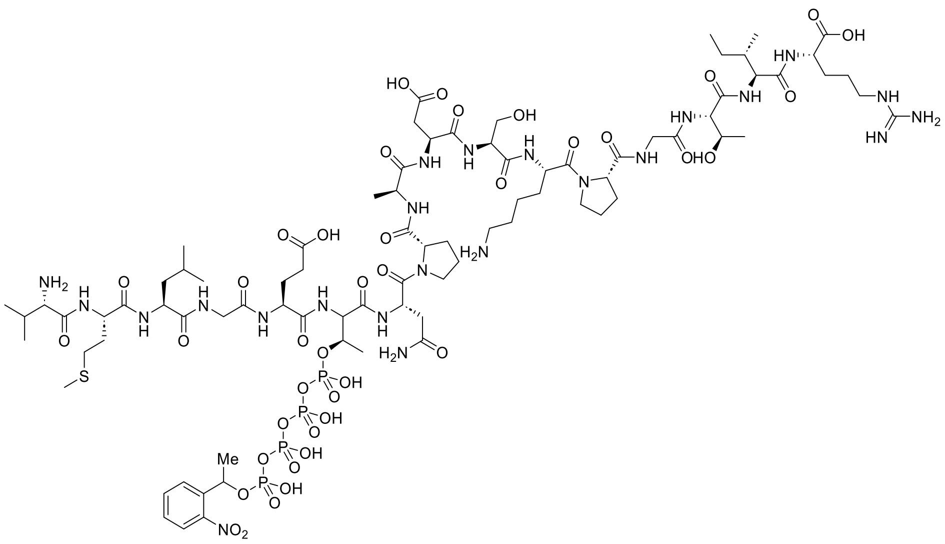

HRMS (ESI): m/z calc'd. for $C_{83}H_{134}AlN_{23}O_{40}P_4S^{2-}$ $[M\text{-}5H\text{+}Al]^{2-}$ 1137.8828, found 1137.8761.

T_R (Method **A**) = 11.722 min (Al), 11.927 min (Fe).

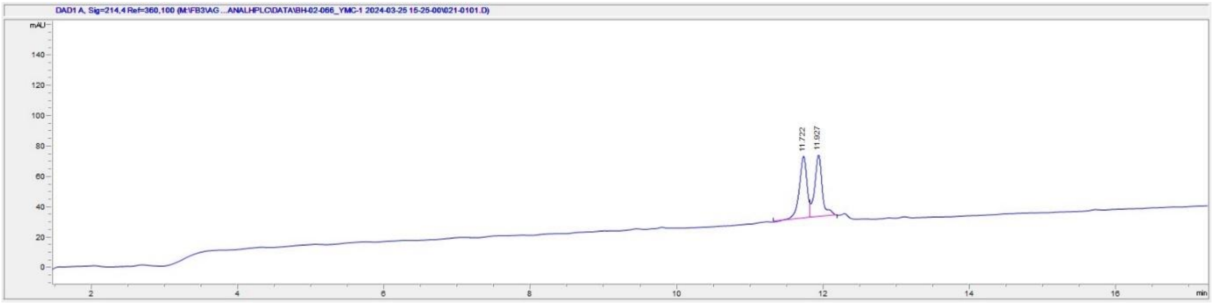

Figure S101: HPLC-UV trace (214 nm) of purified **NPE-p4-Pep3**.

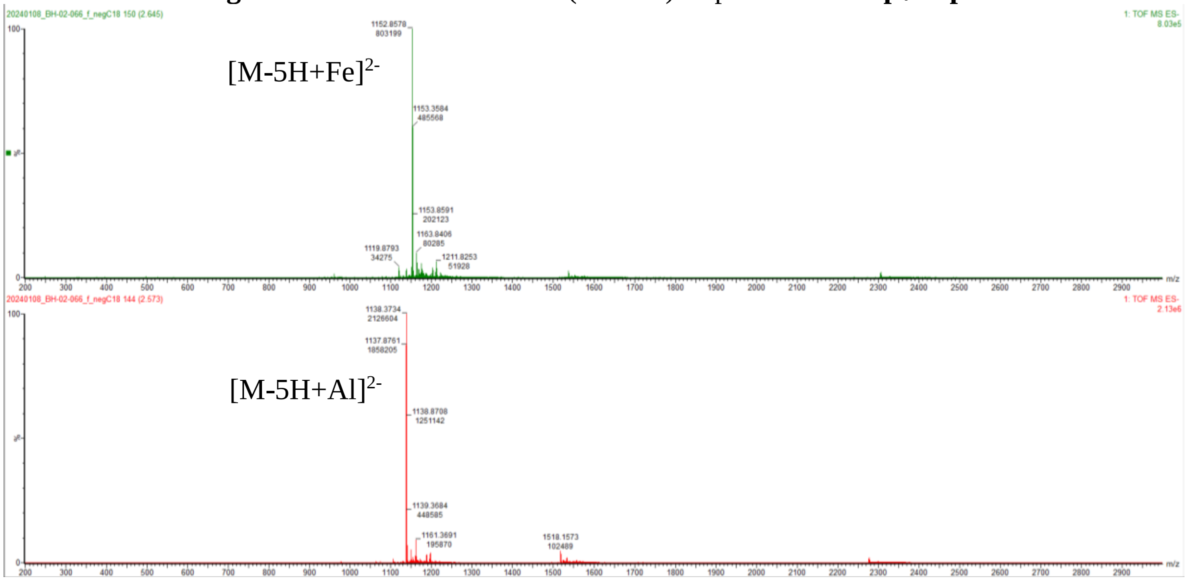

Figure S102: HRMS spectrum of **NPE-p4-Pep3**.

p_4-Pep3
H_2N-Val-Met-Leu-Gly-Glu-[ppppThr]-Asn-Pro-Ala-Asp-Ser-Lys-Pro-Gly-Thr-Ile-Arg-COOH

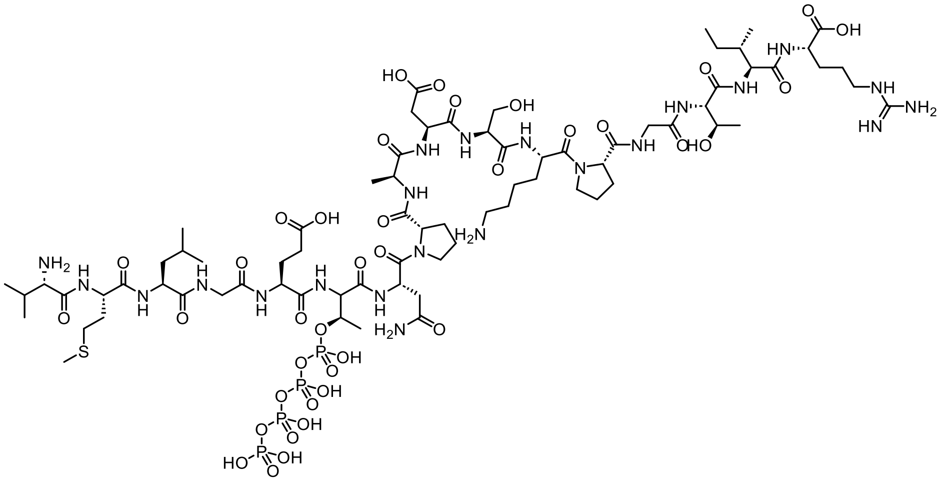

HRMS (ESI): m/z calc'd. for $C_{75}H_{131}AlN_{22}O_{38}P_4S^{2+}$ $[M-H+Al]^{2+}$ 1065.3735, found 1065.3966.
T_R (Method **A**) = 9.395 min.

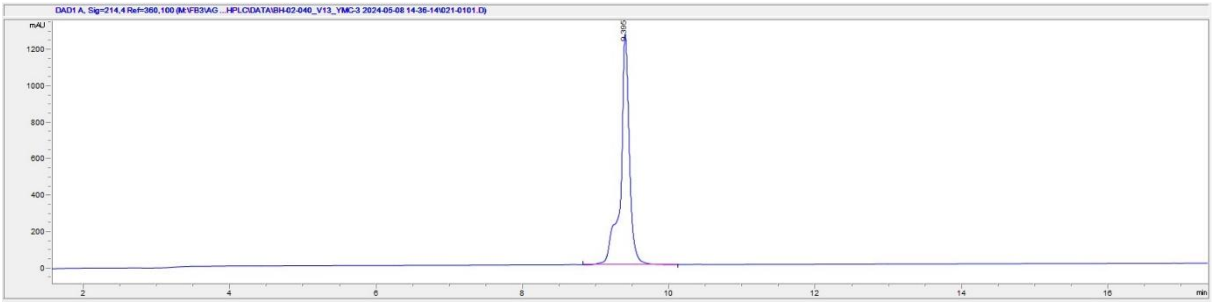

Figure S103: HPLC-UV trace (214 nm) of purified **p_4-Pep3**.

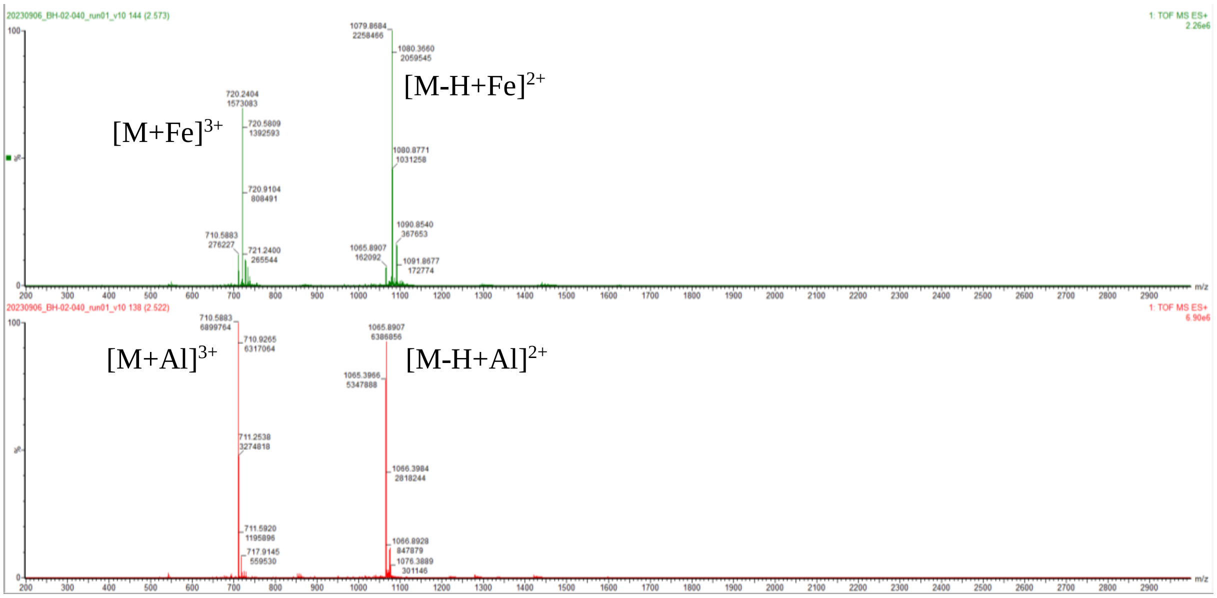

Figure S104: HRMS spectrum of **p_4-Pep3**.

NPE-p$_5$-Pep3

H_2N-Val-Met-Leu-Gly-Glu-[NPE-pppppThr]-Asn-Pro-Ala-Asp-Ser-Lys-Pro-Gly-Thr-Ile-Arg-COOH

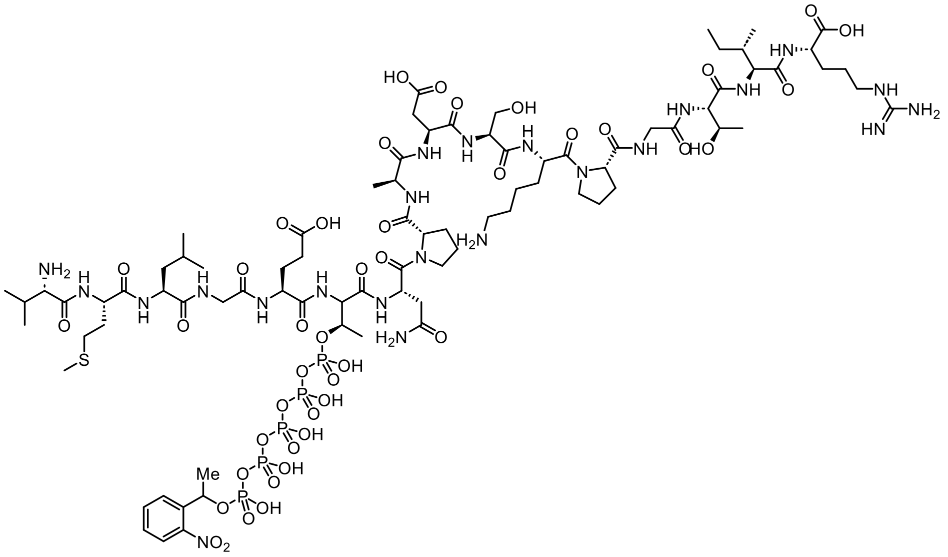

HRMS (ESI): m/z calc'd. for $C_{83}H_{135}AlN_{23}O_{43}P_5S^{2-}$ $[M-5H+Al]^{2-}$ 1177.8659, found 1177.8531.

T_R (Method **A**) = 11.556 min.

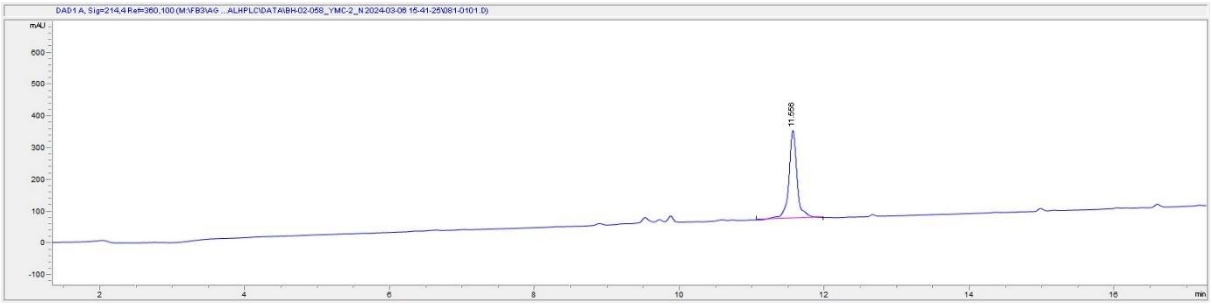

Figure S105: HPLC-UV trace (214 nm) of purified **NPE-p$_5$-Pep3**.

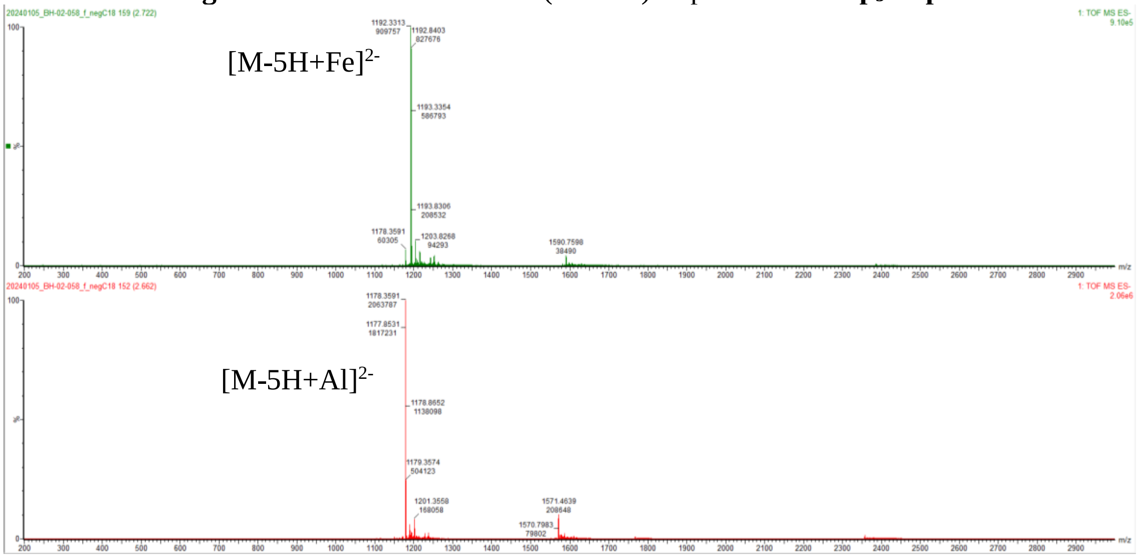

Figure S106: HRMS spectrum of **NPE-p$_5$-Pep3**.

p_5-Pep3

H_2N-Val-Met-Leu-Gly-Glu-[ppppppThr]-Asn-Pro-Ala-Asp-Ser-Lys-Pro-Gly-Thr-Ile-Arg-COOH

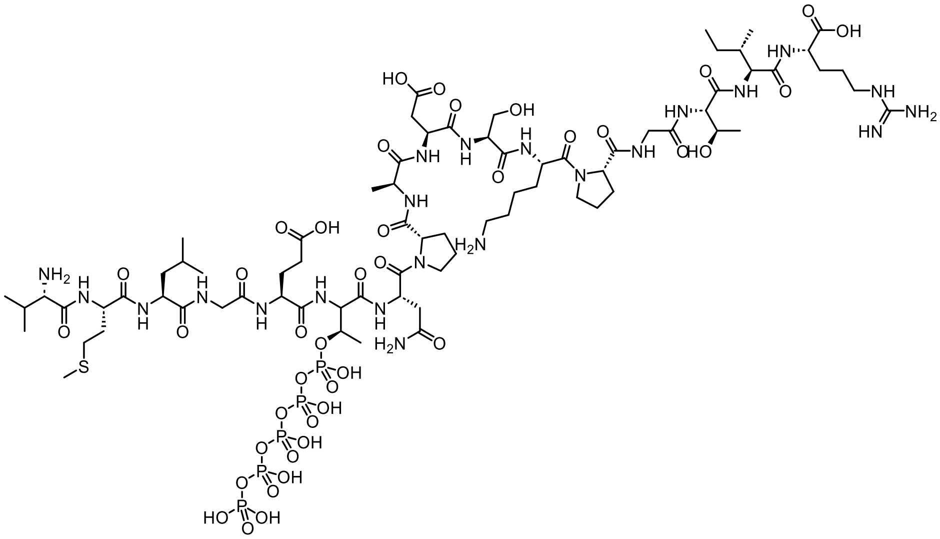

HRMS (ESI): m/z calc'd. for $C_{75}H_{128}FeN_{22}O_{41}P_5S^{2-}$ $[M\text{-}5H\text{+}Fe]^{2-}$ 1117.8185, found 1117.8108.

T_R (Method **A**) = 9.521 min.

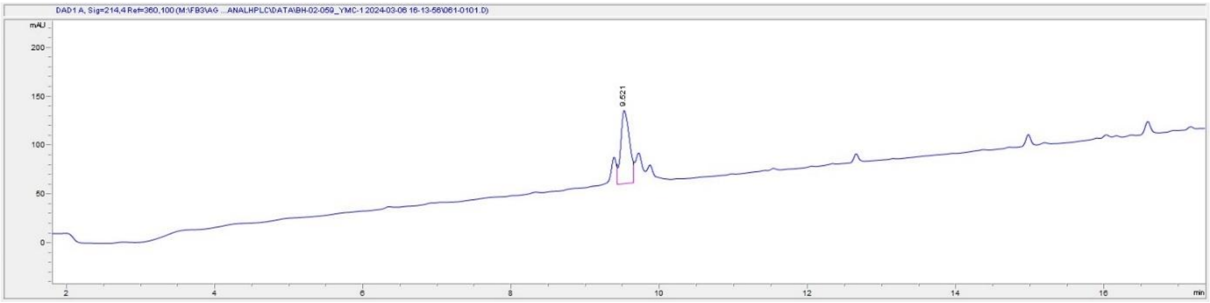

Figure S107: HPLC-UV trace (214 nm) of purified **p_5-Pep3**.

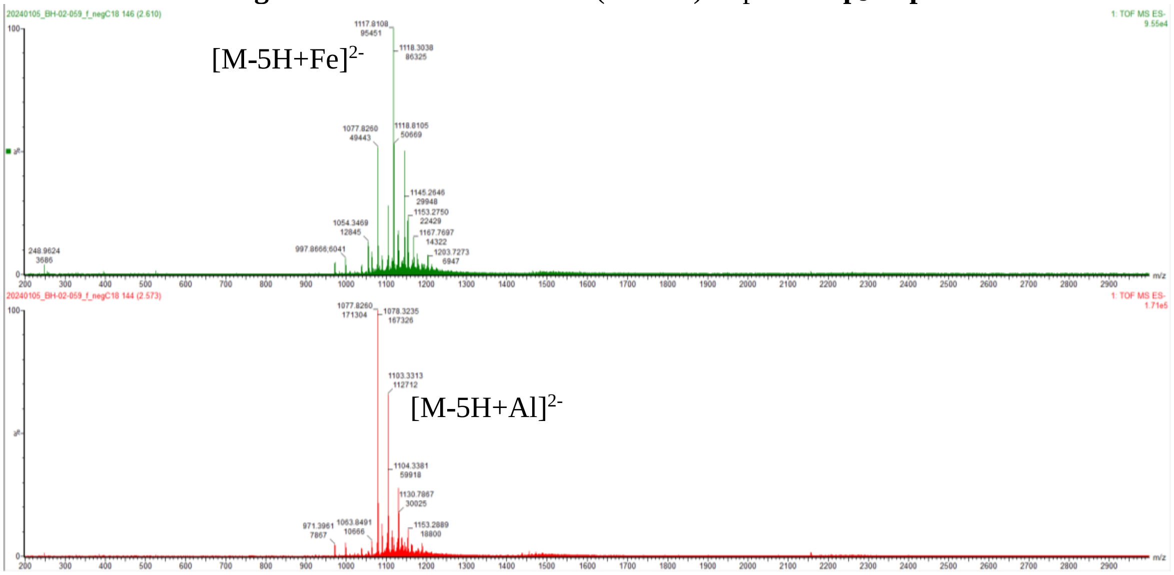

Figure S108: HRMS spectrum of **p_5-Pep3**.

NPE-p$_6$-Pep3

H_2N-Val-Met-Leu-Gly-Glu-[NPE-ppppppThr]-Asn-Pro-Ala-Asp-Ser-Lys-Pro-Gly-Thr-Ile-Arg-COOH

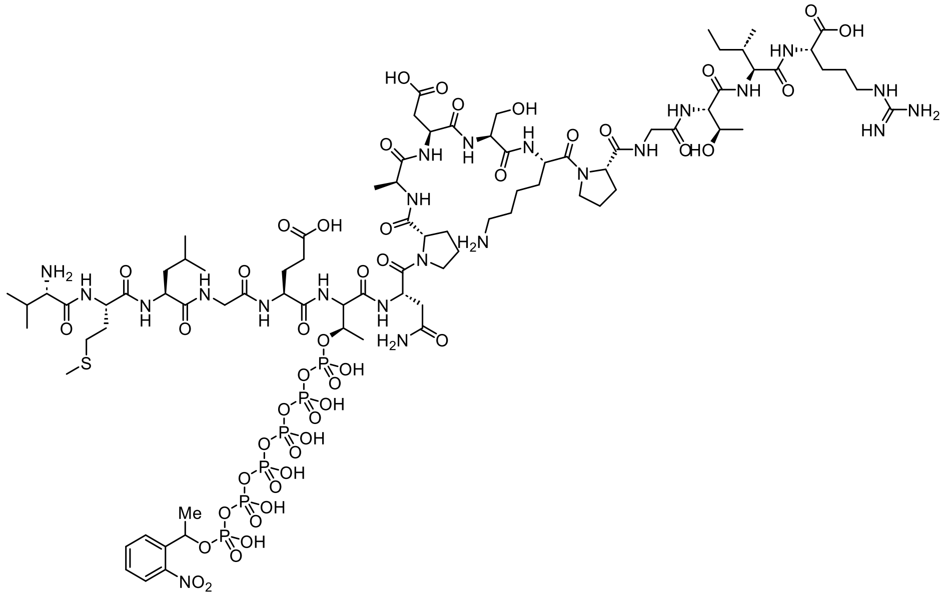

HRMS (ESI): m/z calc'd. for $C_{83}H_{136}AlN_{23}O_{46}P_6S^{2-}$ $[M-5H+Al]^{2-}$ 1217.8491, found 1217.8484.

T_R (Method **A**) = 11.345 min.

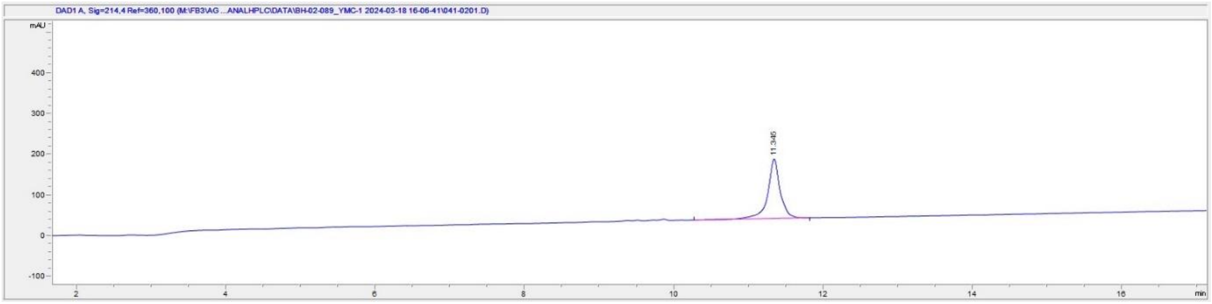

Figure S109: HPLC-UV trace (214 nm) of purified **NPE-p$_6$-Pep3**.

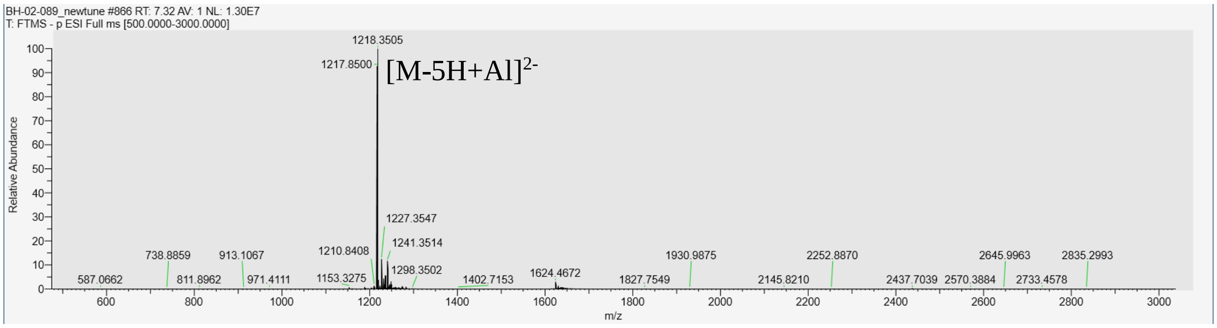

Figure S110: HRMS spectrum of **NPE-p$_6$-Pep3**.

p_6-Pep3

H_2N-Val-Met-Leu-Gly-Glu-[ppppppThr]-Asn-Pro-Ala-Asp-Ser-Lys-Pro-Gly-Thr-Ile-Arg-COOH

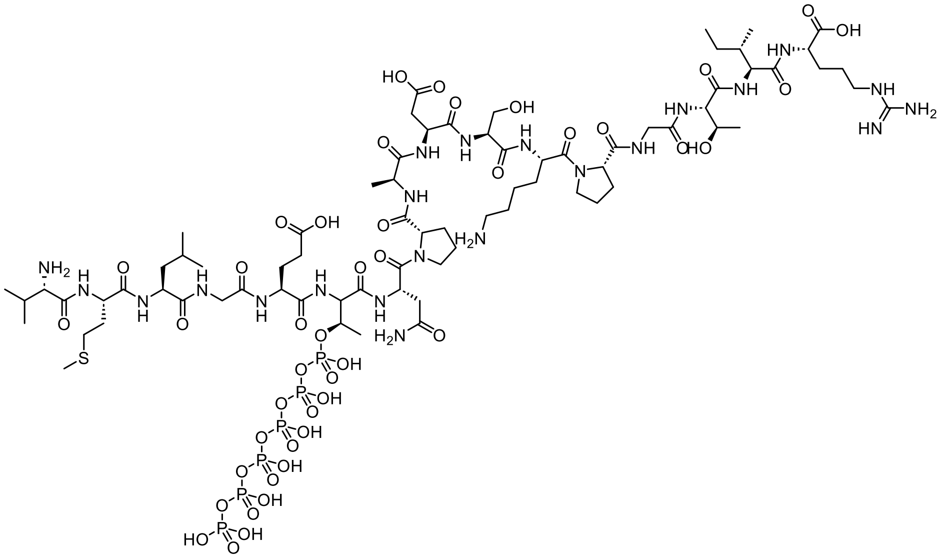

HRMS (ESI): m/z calc'd. for $C_{75}H_{129}AlN_{22}O_{44}P_6S^{2-}$ $[M\text{-}5H\text{+}Al]^{2-}$ 1143.3253, found 1143.3264.

T_R (Method **A**) = 9.158 min (Al), 9.285 (Fe).

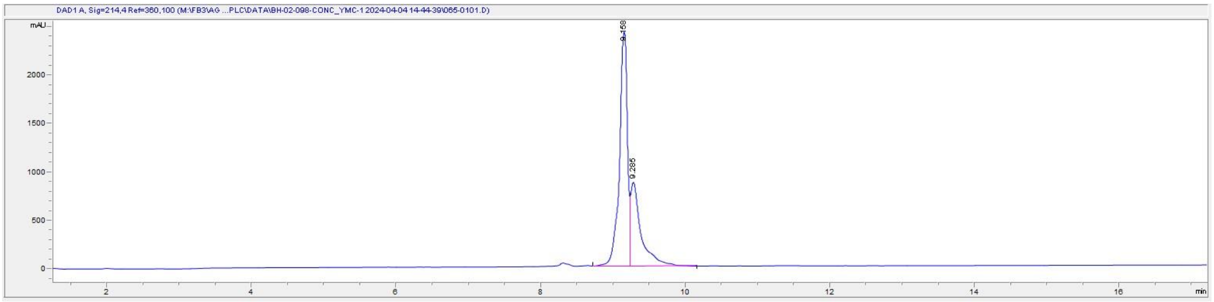

Figure S111: HPLC-UV trace (214 nm) of purified **p_6-Pep3**.

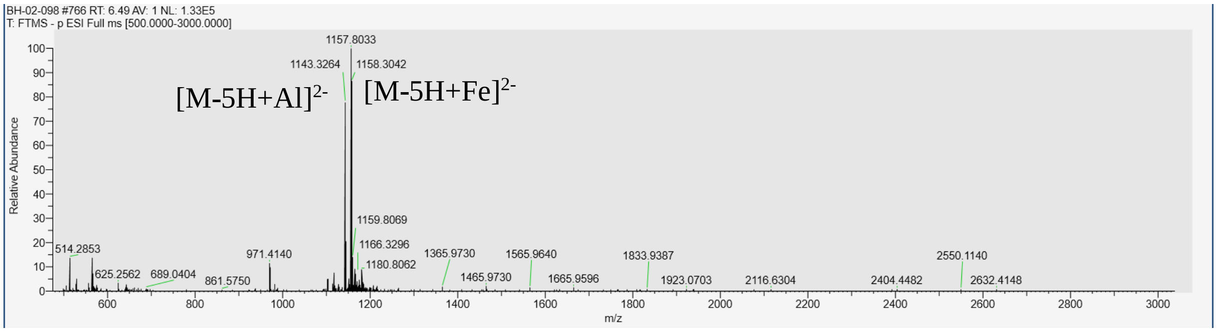

Figure S112: HRMS spectrum of **p_6-Pep3**.

6.4 SRRM1 (Ser795) Peptides

p-Pep4

H_2N-Ser-Pro-Thr-Pro-[pSer]-Pro-Ser-Pro-Pro-Arg-Asn-Ser-Asp-Gln-Glu-Gly-Gly-Gly-Lys-COOH

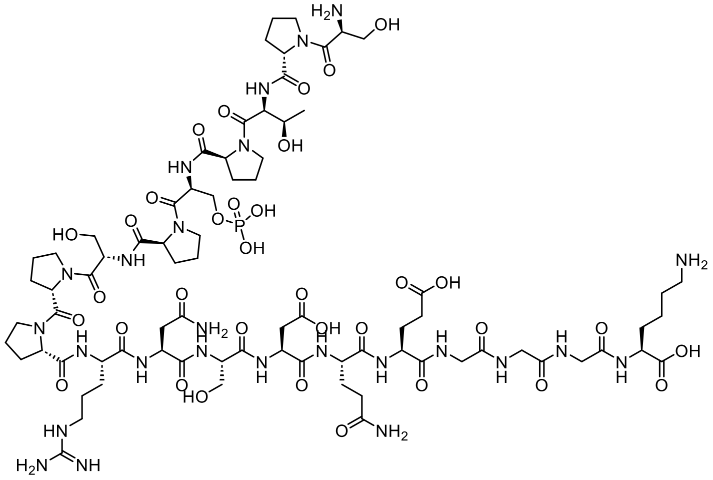

HRMS (ESI): m/z calc'd. for $C_{77}H_{122}N_{25}O_{34}P^{2-}$ $[M-2H]^{2-}$ 985.9167, found 985.9174.
T_R (Method **A**) = 6.053 min.

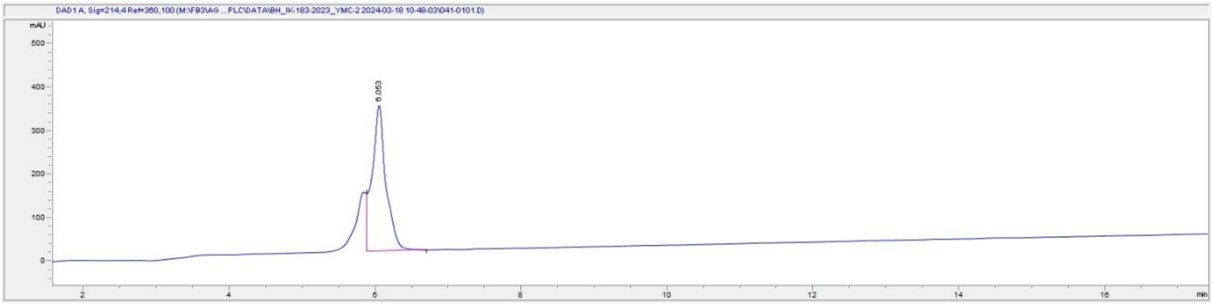

Figure S113: HPLC-UV trace (214 nm) of purified **p-Pep4**.

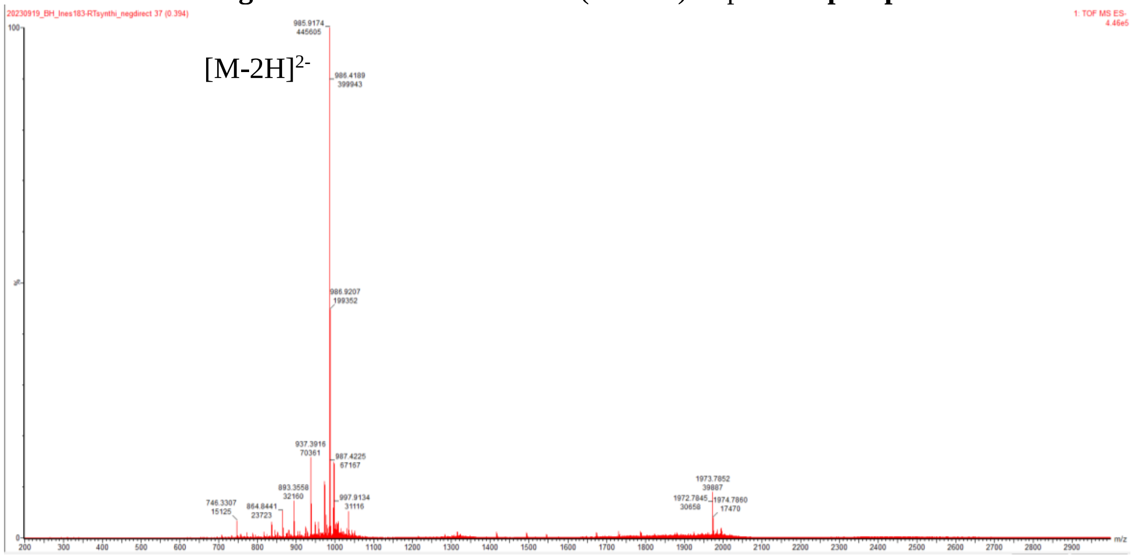

Figure S114: HRMS spectrum of **p-Pep4**.

NPE-p₂-Pep4

H$_2$N-Ser-Pro-Thr-Pro-[NPE-ppSer]-Pro-Ser-Pro-Pro-Arg-Asn-Ser-Asp-Gln-Glu-Gly-Gly-Gly-Lys-COOH

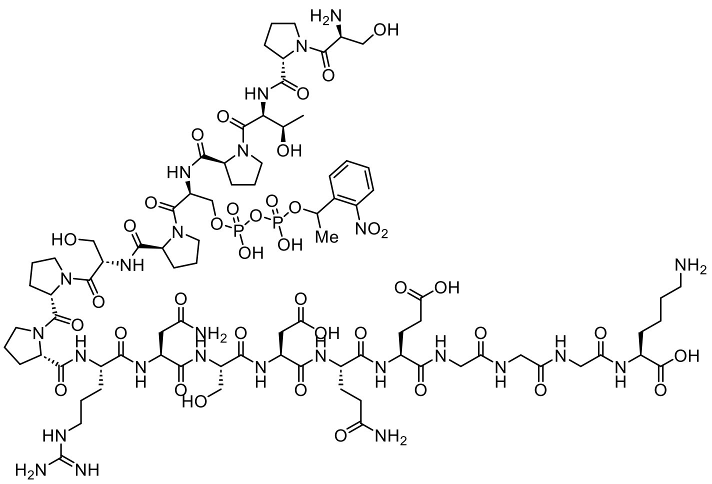

HRMS (ESI): m/z calc'd. for $C_{85}H_{130}N_{26}O_{39}P_2^{2-}$ $[M\text{-}2H]^{2-}$ 1100.4237, found 1100.4232.

T_R (Method **A**) = 8.296 min.

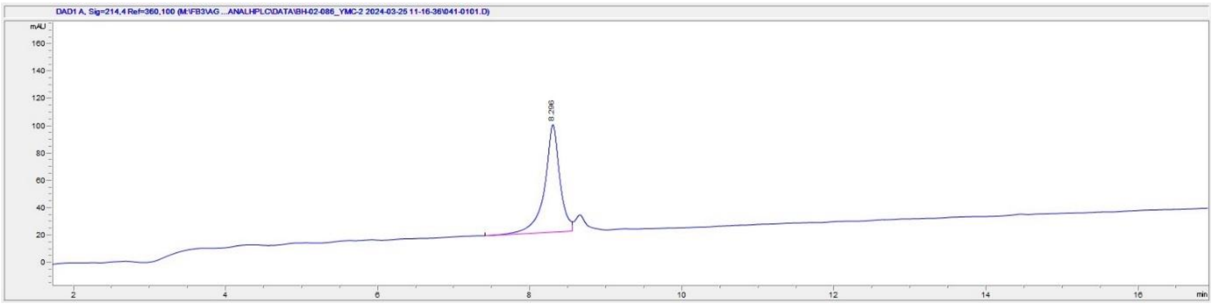

Figure S115: HPLC-UV trace (214 nm) of purified **NPE-p$_2$-Pep4**.

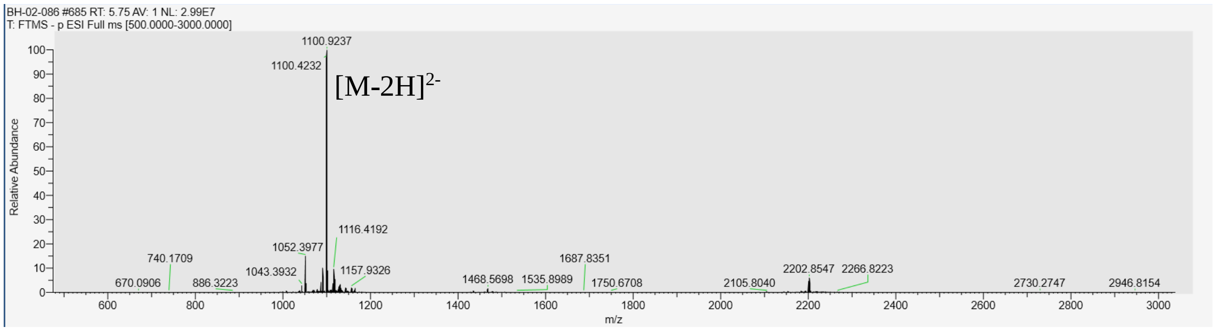

Figure S116: HRMS spectrum of **NPE-p$_2$-Pep4**.

p_2-Pep4
H_2N-Ser-Pro-Thr-Pro-[ppSer]-Pro-Ser-Pro-Pro-Arg-Asn-Ser-Asp-Gln-Glu-Gly-Gly-Gly-Lys-COOH

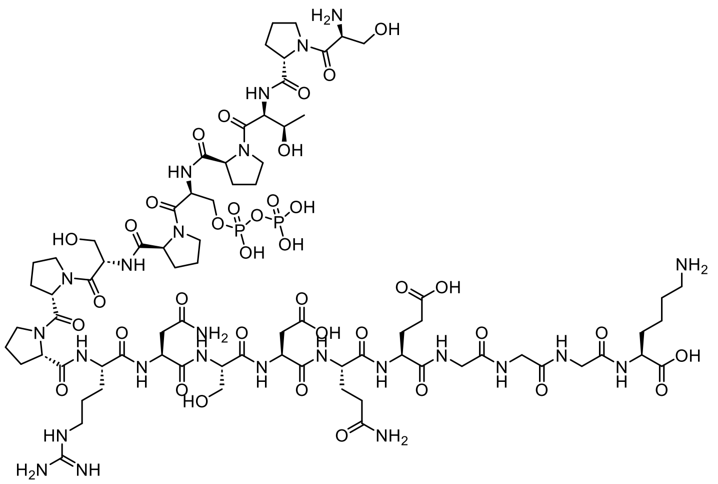

HRMS (ESI): m/z calc'd. for $C_{77}H_{123}N_{25}O_{37}P_2^{2-}$ $[M-2H]^{2-}$ 1025.8999, found 1025.9006.
T_R (Method **A**) = 5.785 min.

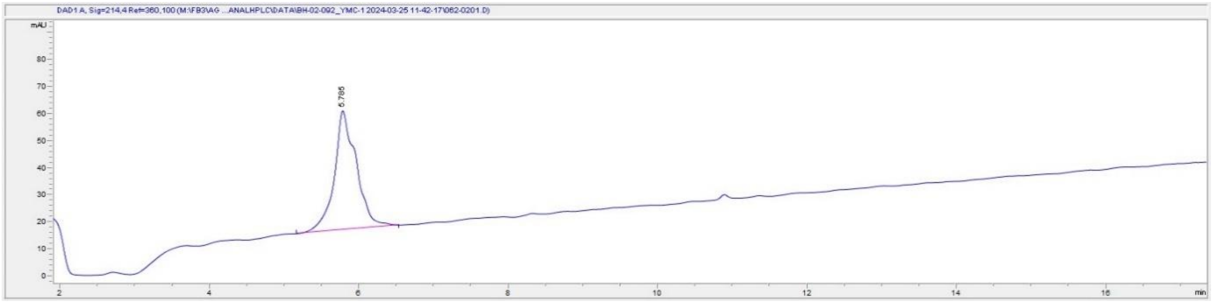

Figure S117: HPLC-UV trace (214 nm) of purified **p_2-Pep4**.

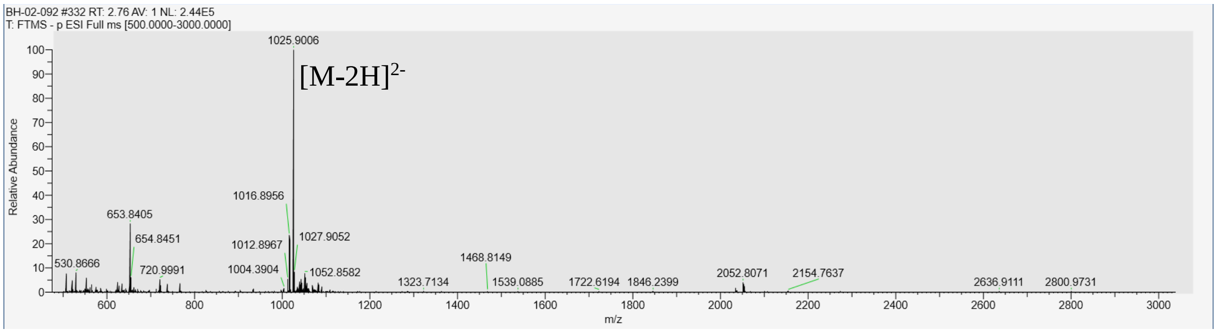

Figure S118: HRMS spectrum of **p_2-Pep4**.

Bn-p$_3$-Pep4
H_2N-Ser-Pro-Thr-Pro-[Bn-pppSer]-Pro-Ser-Pro-Pro-Arg-Asn-Ser-Asp-Gln-Glu-Gly-Gly-Gly-Lys-COOH

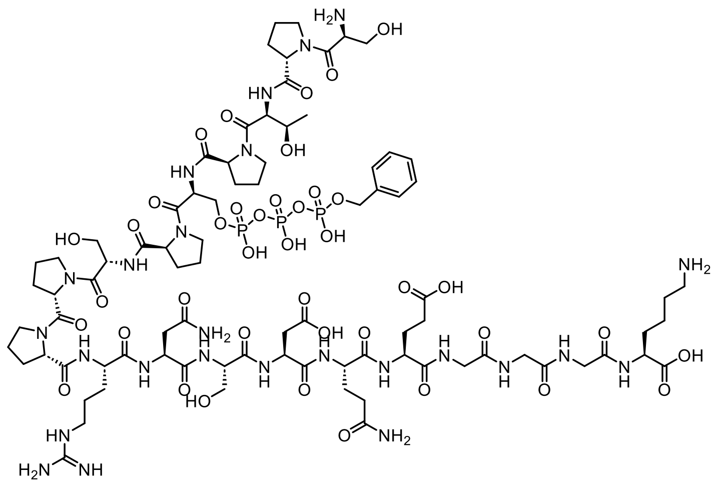

HRMS (ESI): m/z calc'd. for $C_{84}H_{127}AlN_{25}O_{40}P_3^{2-}$ $[M\text{-}5H+Al]^{2-}$ 1122.8855, found 122.8865.

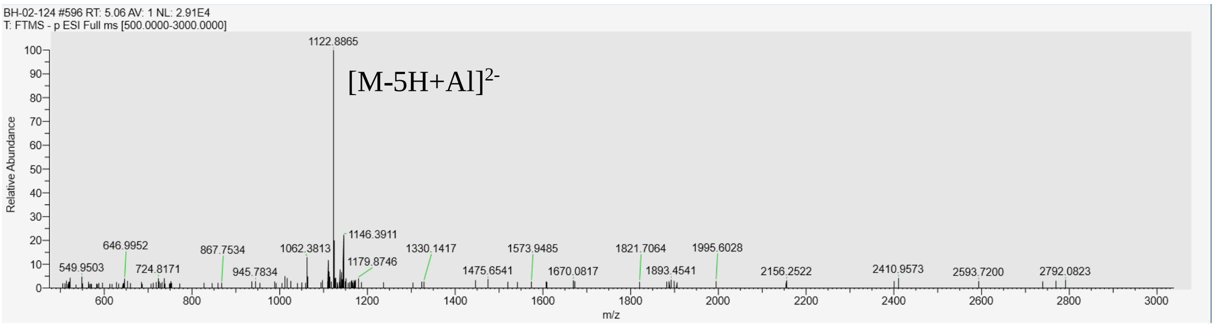

Figure S119: HRMS spectrum of **Bn-p$_3$-Pep4**.

p$_3$-Pep4
H_2N-Ser-Pro-Thr-Pro-[pppSer]-Pro-Ser-Pro-Pro-Arg-Asn-Ser-Asp-Gln-Glu-Gly-Gly-Gly-Lys-COOH

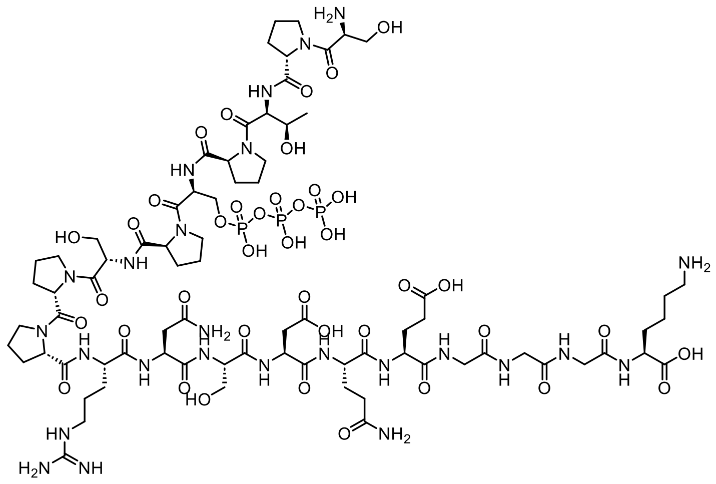

HRMS (ESI): m/z calc'd. for $C_{77}H_{121}AlN_{25}O_{40}P_3^{2-}$ $[M\text{-}5H+Al]^{2-}$ 1077.8621, found 1077.6.

NPE-p$_4$-Pep4

H$_2$N-Ser-Pro-Thr-Pro-[NPE-ppppSer]-Pro-Ser-Pro-Pro-Arg-Asn-Ser-Asp-Gln-Glu-Gly-Gly-Gly-Lys-COOH

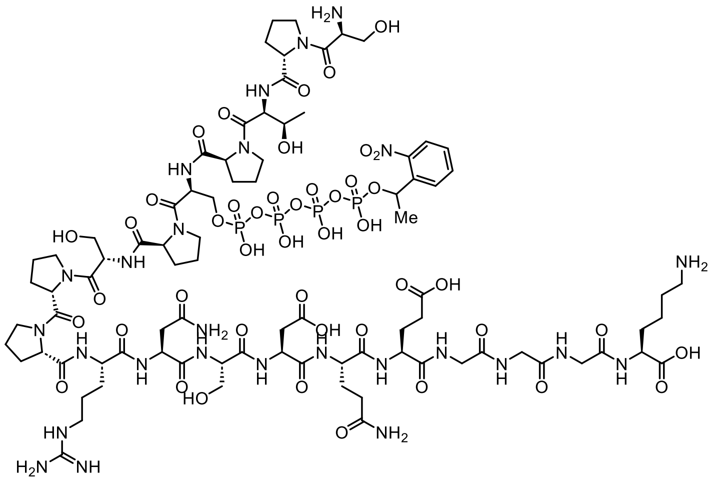

HRMS (ESI): m/z calc'd. for $C_{85}H_{129}AlN_{26}O_{45}P_4^{2-}$ [M-5H+Al]$^{2-}$ 1192.3691, found 1192.3595.

T$_R$ (Method **A**) = 8.287 min (Al), 8.538 min (Al+Fe), 8.765 min (Fe).

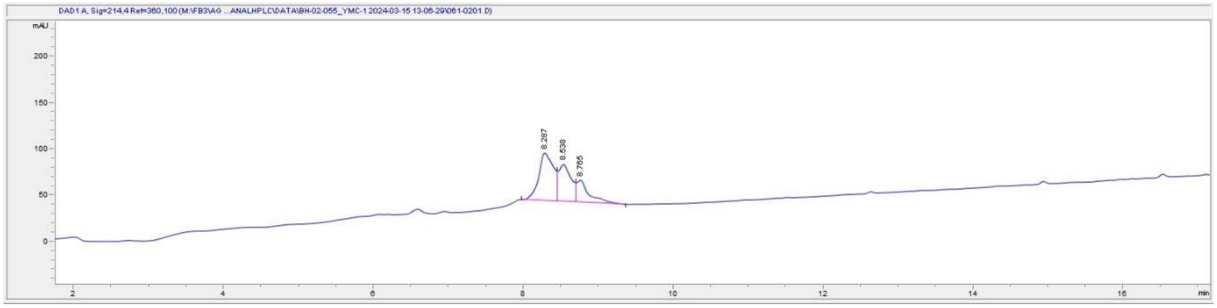

Figure S120: HPLC-UV trace (214 nm) of purified **NPE-p$_4$-Pep4**.

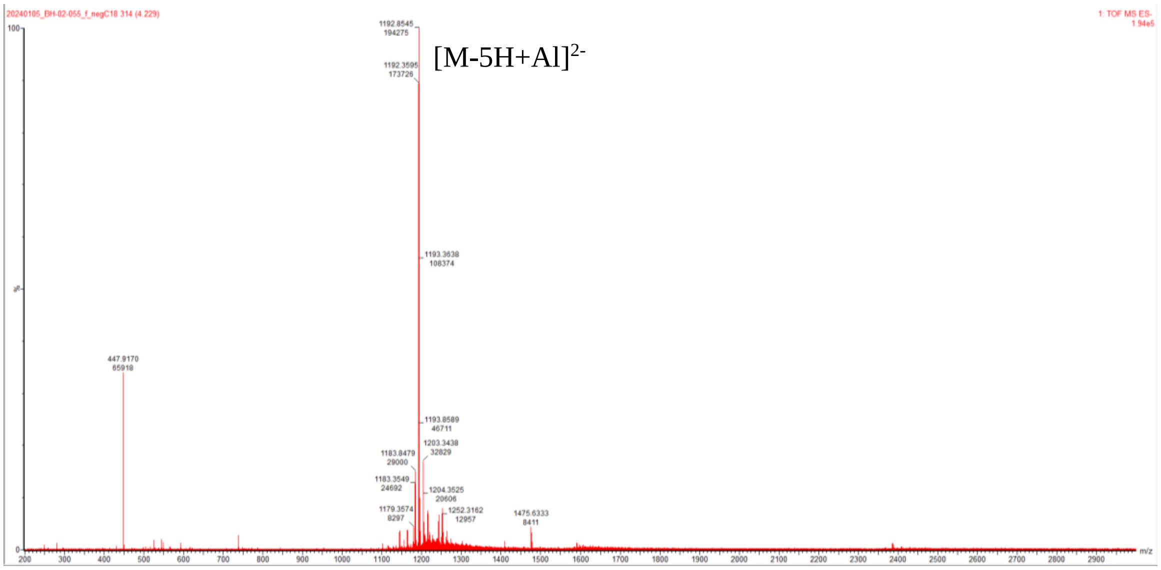

Figure S121: HRMS spectrum of **NPE-p$_4$-Pep4**.

p_4-Pep4

H_2N-Ser-Pro-Thr-Pro-[ppppSer]-Pro-Ser-Pro-Pro-Arg-Asn-Ser-Asp-Gln-Glu-Gly-Gly-Gly-Lys-COOH

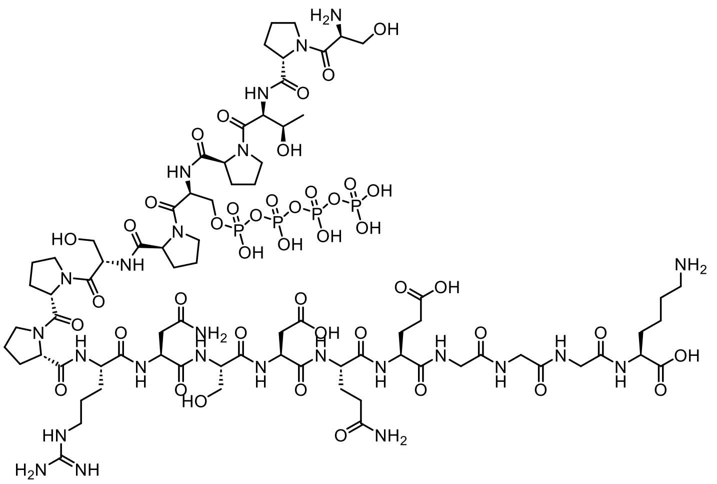

HRMS (ESI): m/z calc'd. for $C_{77}H_{122}AlN_{25}O_{43}P_4^{2-}$ [M-5H+Al]$^{2-}$ 1117.8452, found 1117.8383.
T_R (Method **A**) = 5.842 min.

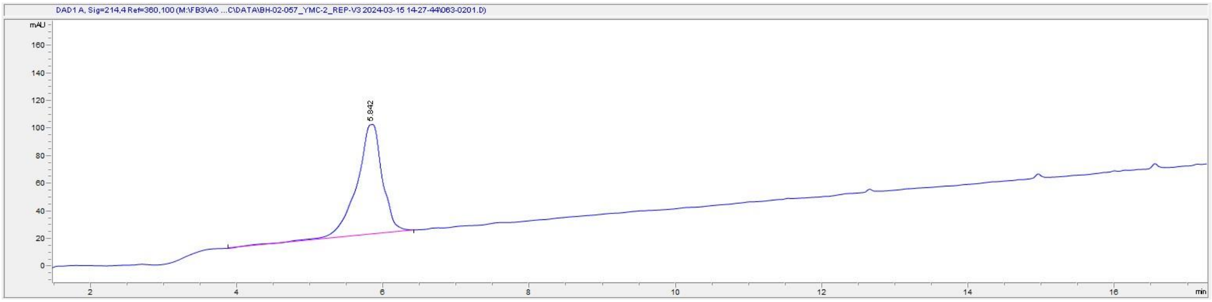

Figure S122: HPLC-UV trace (214 nm) of purified **p_4-Pep4**.

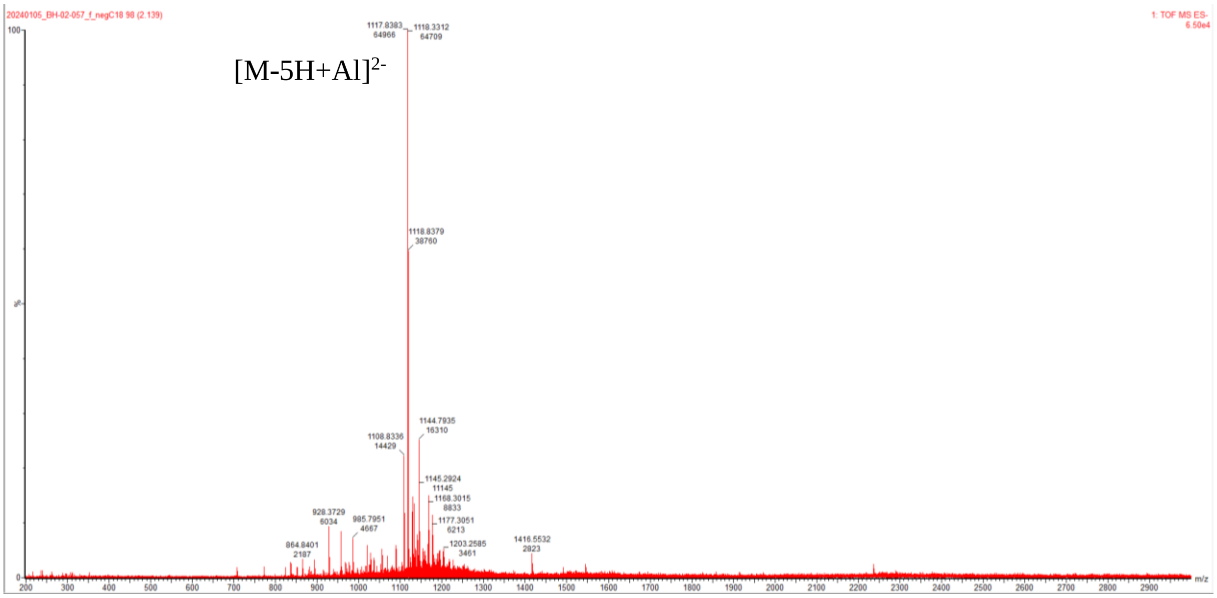

Figure S123: HRMS spectrum of **p_4-Pep4**.

NPE-p$_5$-Pep4
H$_2$N-Ser-Pro-Thr-Pro-[NPE-pppppSer]-Pro-Ser-Pro-Pro-Arg-Asn-Ser-Asp-Gln-Glu-Gly-Gly-Gly-Lys-COOH

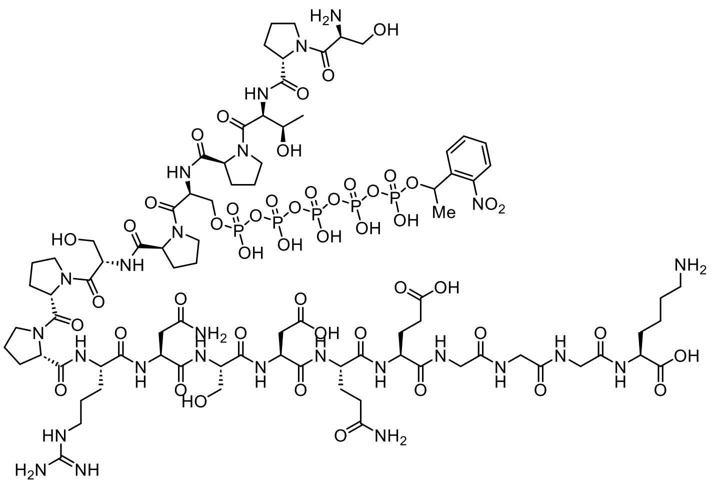

HRMS (ESI): m/z calc'd. for $C_{85}H_{130}AlN_{26}O_{48}P_5^{2-}$ $[M-5H+Al]^{2-}$ 1232.3522, found 1232.3534.
T_R (Method **A**) = 7.938 min.

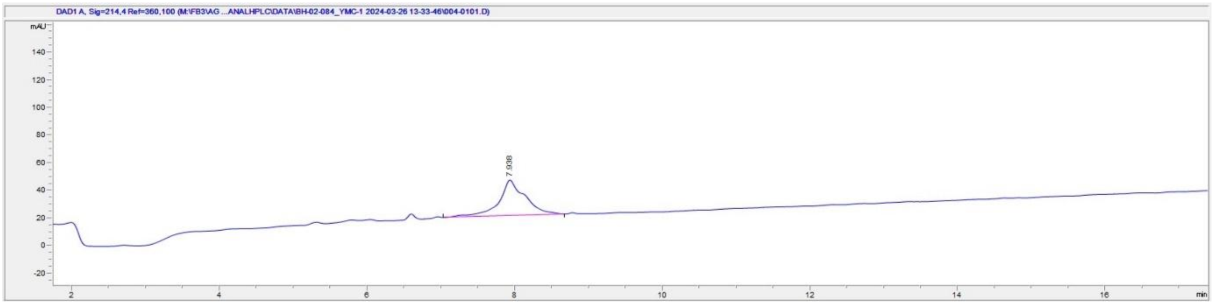

Figure S124: HPLC-UV trace (214 nm) of purified **NPE-p$_5$-Pep4**.

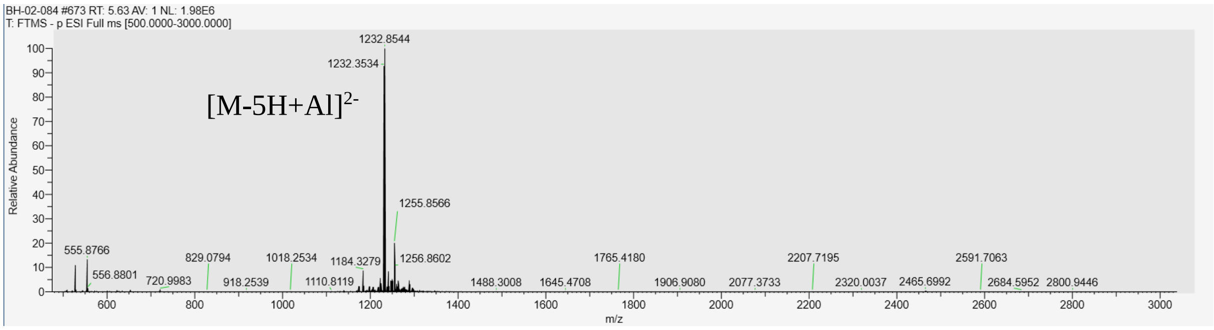

Figure S125: HRMS spectrum of **NPE-p$_5$-Pep4**.

p_5-Pep4
H_2N-Ser-Pro-Thr-Pro-[pppppSer]-Pro-Ser-Pro-Pro-Arg-Asn-Ser-Asp-Gln-Glu-Gly-Gly-Gly-Lys-COOH

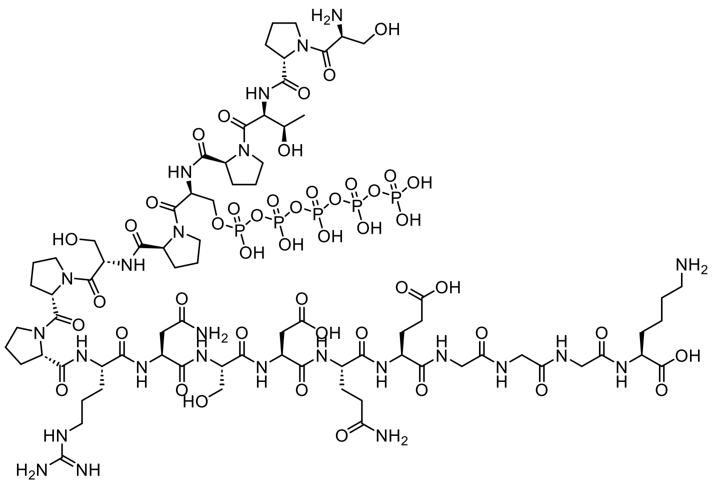

HRMS (ESI): m/z calc'd. for $C_{77}H_{123}AlN_{25}O_{46}P_5^{2-}$ $[M-5H+Al]^{2-}$ 1157.8284, found 1157.8263.
T_R (Method **A**) = 5.665 min.

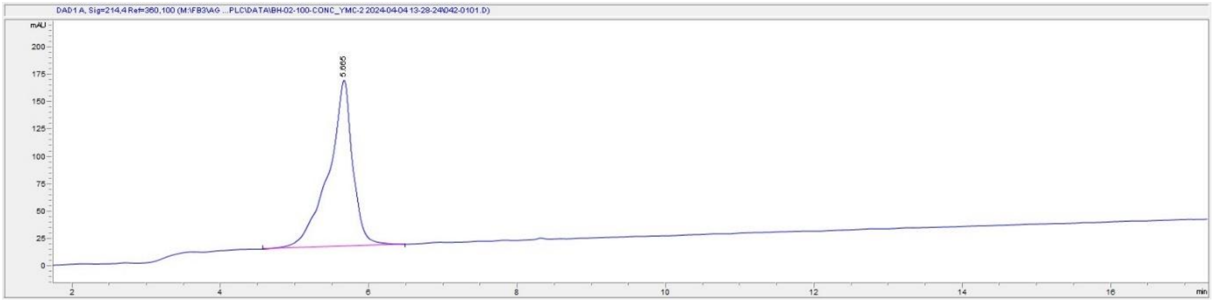

Figure S126: HPLC-UV trace (214 nm) of purified **p_5-Pep4**.

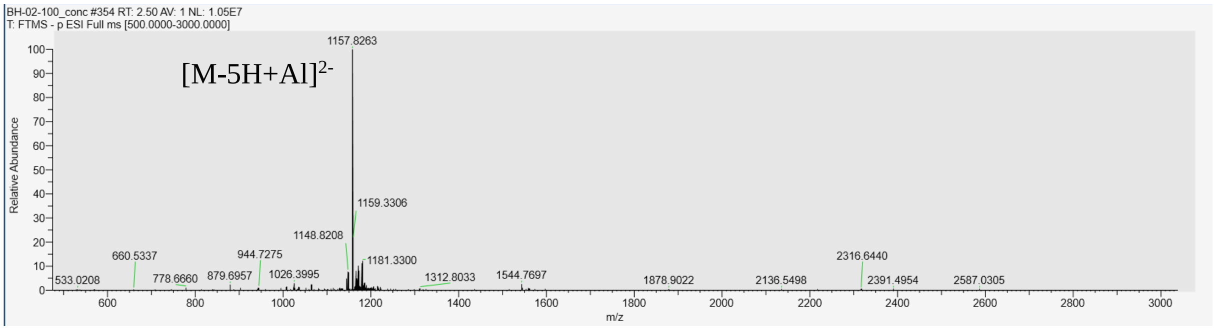

Figure S127: HRMS spectrum of **p_5-Pep4**.

NPE-p6-Pep4

H_2N-Ser-Pro-Thr-Pro-[NPE-ppppppSer]-Pro-Ser-Pro-Pro-Arg-Asn-Ser-Asp-Gln-Glu-Gly-Gly-Gly-Lys-COOH

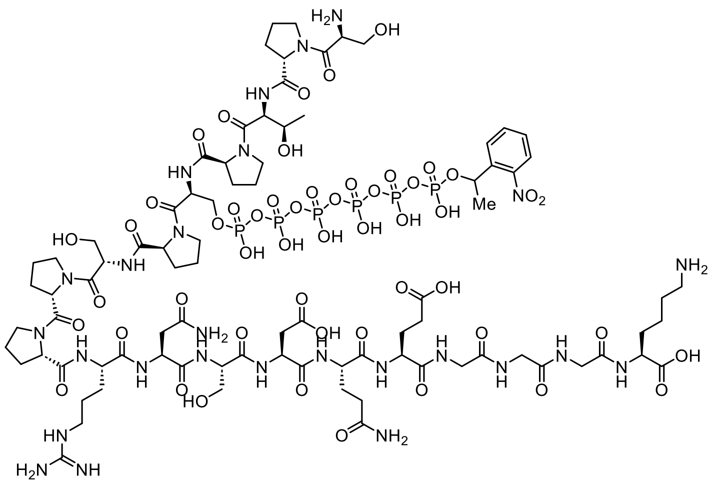

HRMS (ESI): m/z calc'd. for $C_{85}H_{131}AlN_{26}O_{51}P_6^{2-}$ $[M\text{-}5H+Al]^{2-}$ 1272.3354, found 1272.3374.

T_R (Method **A**) = 7.692 min (Al), 7.892 (Fe).

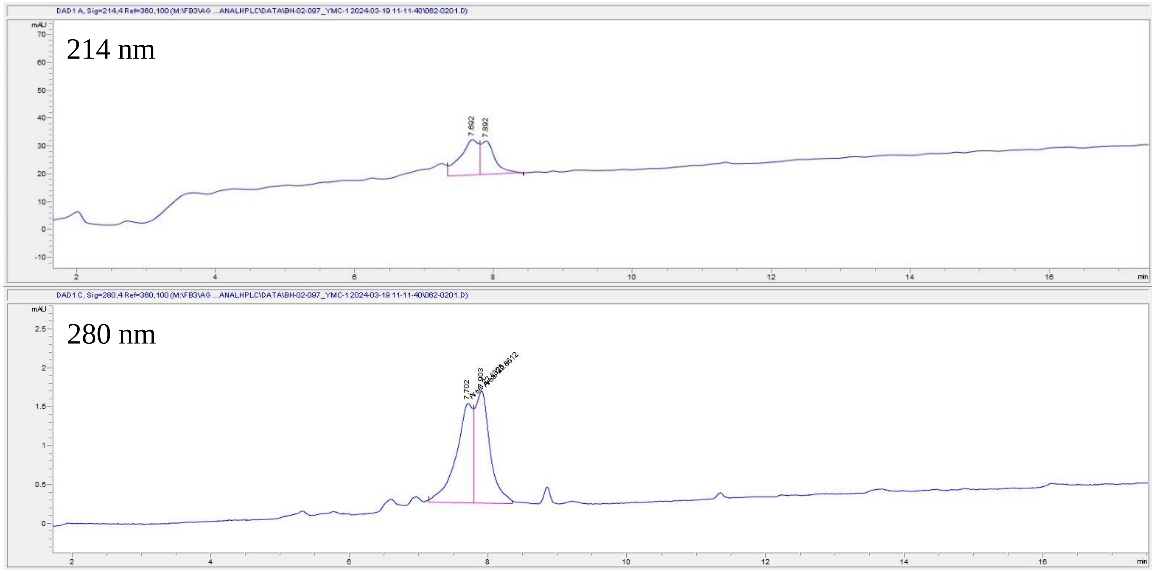

Figure S128: HPLC-UV traces of purified **NPE-p6-Pep4**.

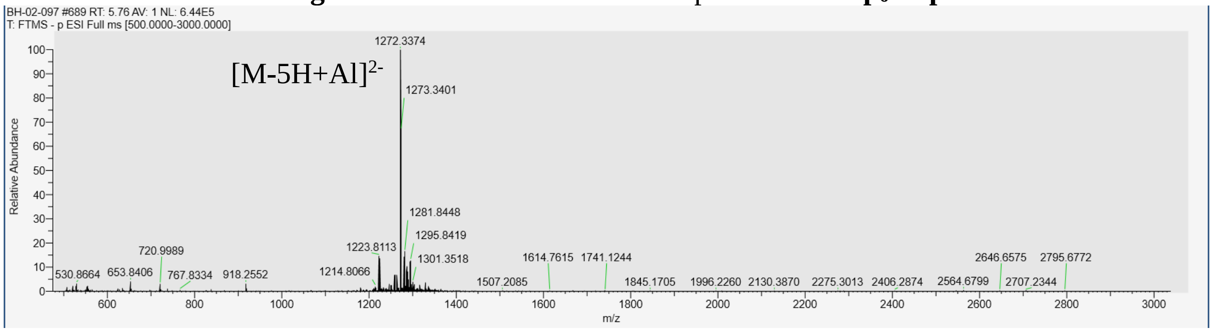

Figure S129: HRMS spectrum of **NPE-p6-Pep4**.

p_6-Pep4

H_2N-Ser-Pro-Thr-Pro-[ppppppSer]-Pro-Ser-Pro-Pro-Arg-Asn-Ser-Asp-Gln-Glu-Gly-Gly-Gly-Lys-COOH

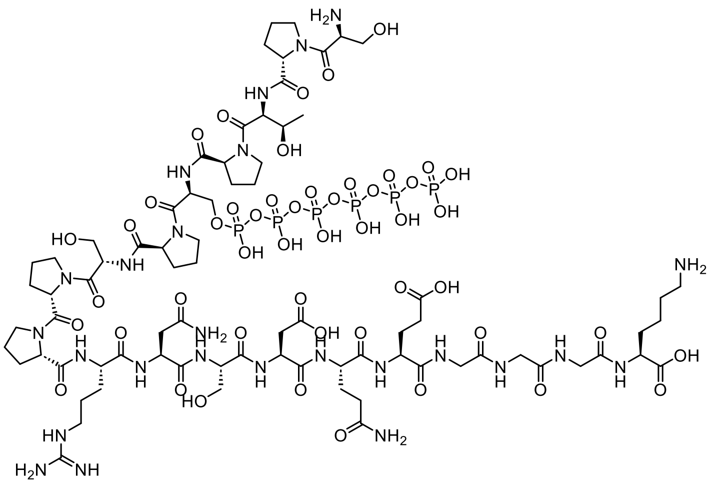

HRMS (ESI): m/z calc'd. for $C_{77}H_{124}AlN_{25}O_{49}P_6^{2-}$ $[M-5H+Al]^{2-}$ 1197.8116, found 1197.6.

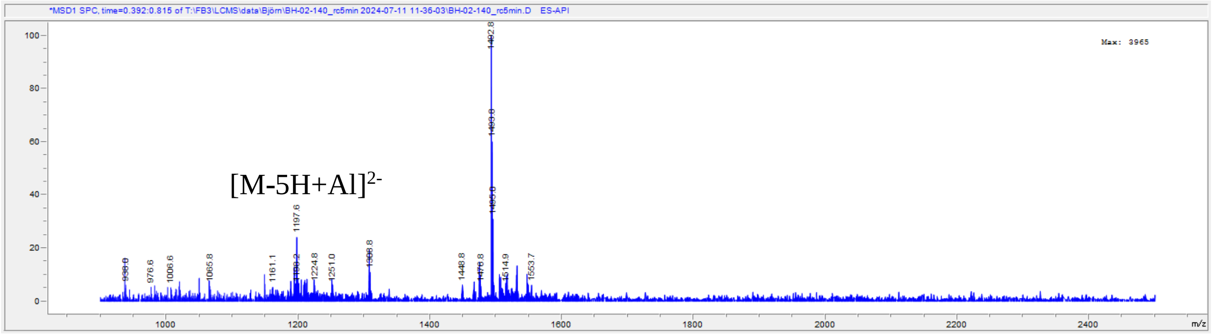

Figure S130: LR-MS spectrum of **p_6-Pep4**. Product ion not sufficiently visible on QTof or Q Exactive.

6.5 DEK (Ser303) Peptides

p-Pep5

H_2N-Lys-Glu-Ser-Glu-[pSer]-Glu-Asp-Ser-Ser-Asp-Asp-Glu-Pro-Leu-Ile-Lys-COOH

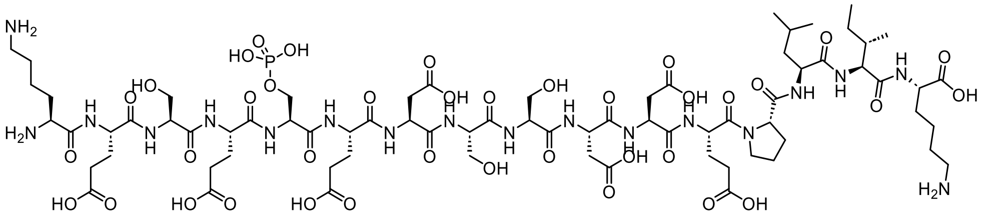

HRMS (ESI): m/z calc'd. for $C_{73}H_{117}N_{18}O_{38}P^{2-}$ $[M-2H]^{2-}$ 942.3762, found 942.3735.
T_R (Method **A**) = 9.059 min.

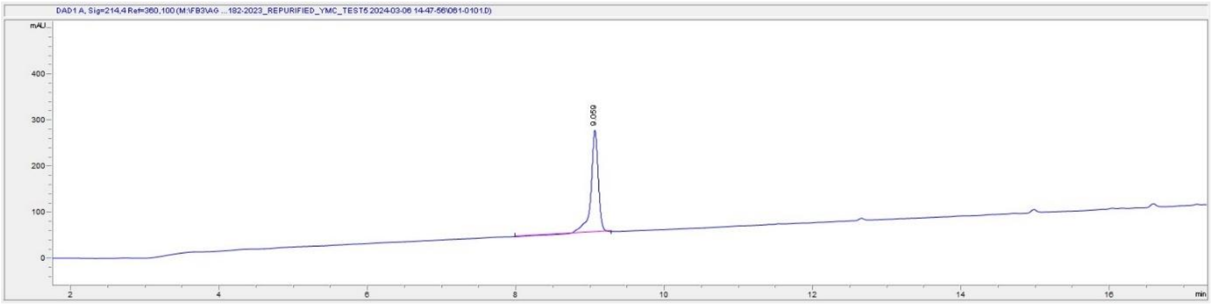

Figure S131: HPLC-UV trace (214 nm) of purified **p-Pep5**.

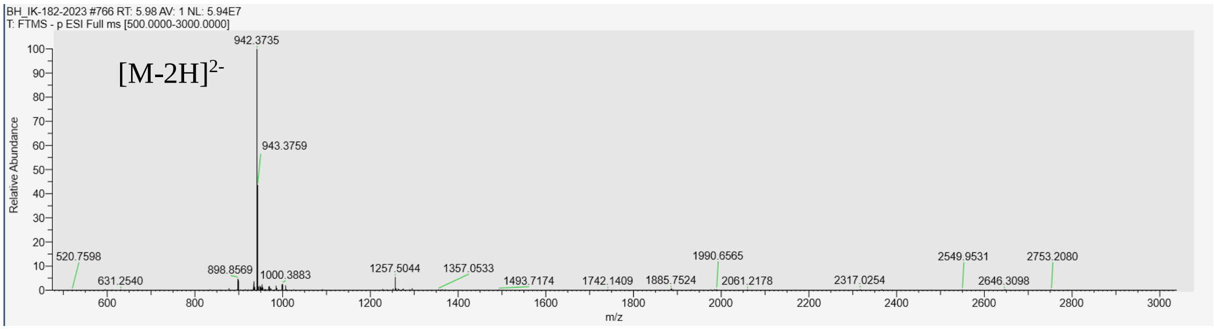

Figure S132: HRMS spectrum of **p-Pep5**.

NPE-p$_2$-Pep5

H_2N-Lys-Glu-Ser-Glu-[NPE-ppSer]-Glu-Asp-Ser-Ser-Asp-Asp-Glu-Pro-Leu-Ile-Lys-COOH

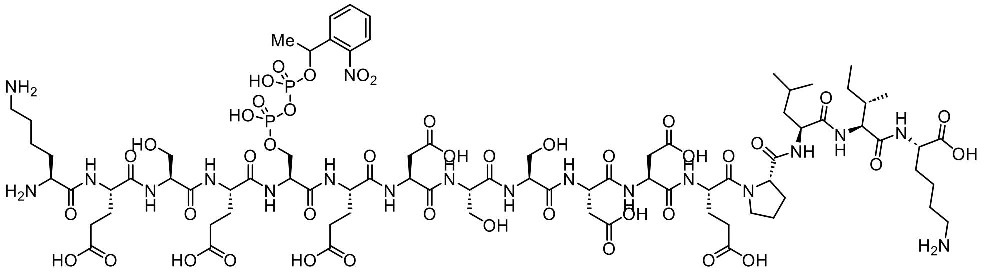

HRMS (ESI): m/z calc'd. for $C_{81}H_{125}N_{19}O_{43}P_2^{2-}$ $[M-2H]^{2-}$ 1056.8832, found 1056.8809.
T_R (Method **A**) = 10.741 min.

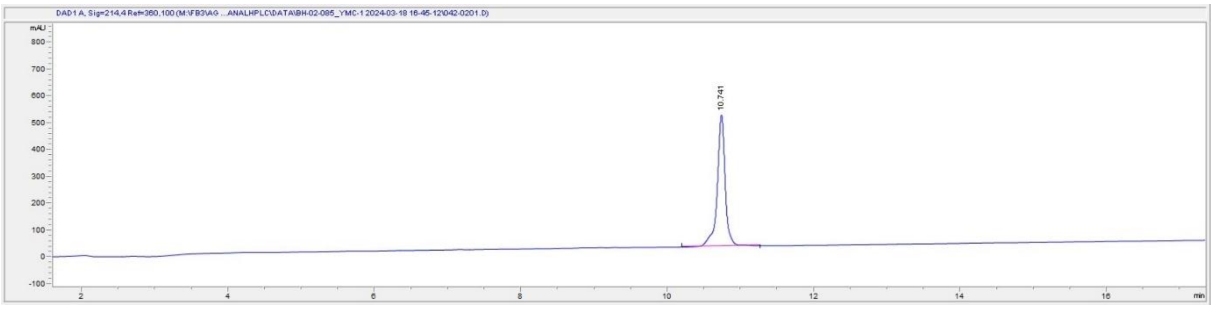

Figure S133: HPLC-UV trace (214 nm) of purified **NPE-p$_2$-Pep5**.

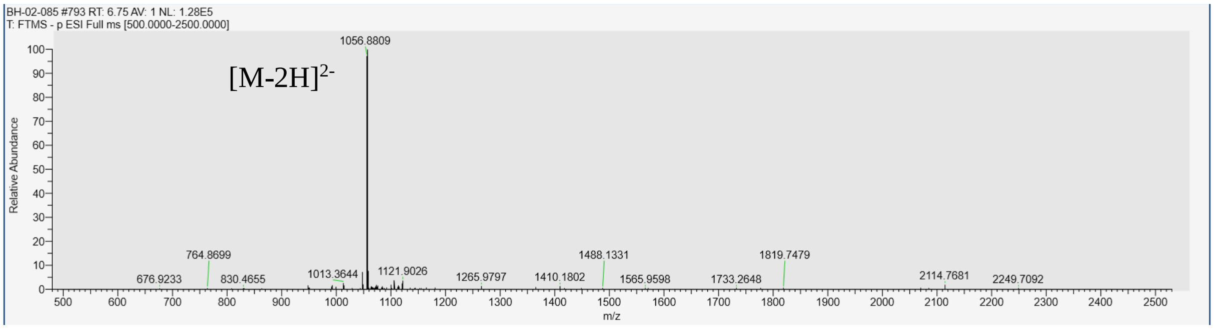

Figure S134: HRMS spectrum of **NPE-p$_2$-Pep5**.

p_2-Pep5
H_2N-Lys-Glu-Ser-Glu-[ppSer]-Glu-Asp-Ser-Ser-Asp-Asp-Glu-Pro-Leu-Ile-Lys-COOH

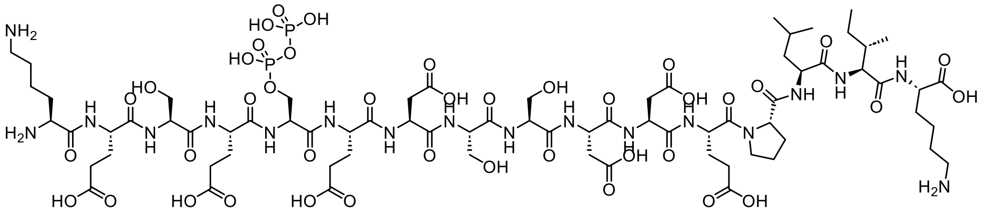

HRMS (ESI): m/z calc'd. for $C_{73}H_{118}N_{18}O_{41}P_2^{2-}$ $[M-2H]^{2-}$ 982.3594, found 982.3602.
T_R (Method **A**) = 8.796 min.

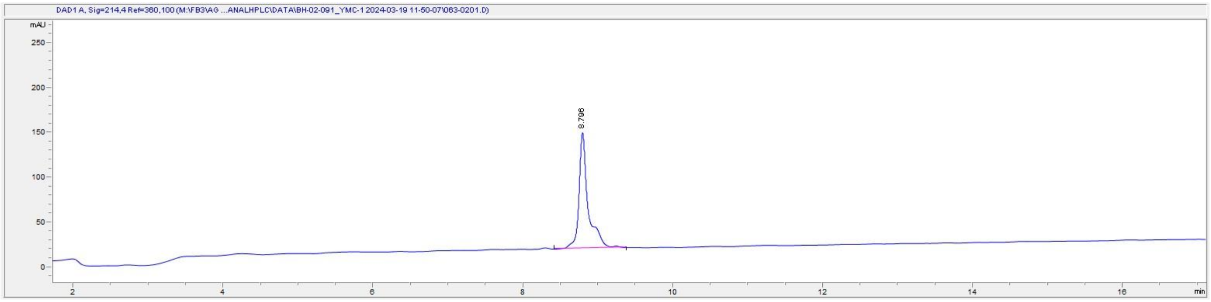

Figure S135: HPLC-UV trace (214 nm) of purified **p_2-Pep5**.

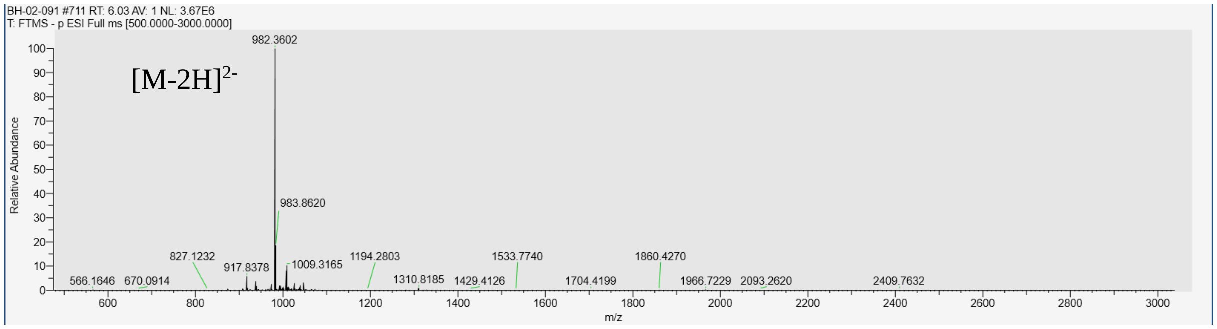

Figure S136: HRMS spectrum of **p_2-Pep5**.

Bn-p$_3$-Pep5

H_2N-Lys-Glu-Ser-Glu-[Bn-pppSer]-Glu-Asp-Ser-Ser-Asp-Asp-Glu-Pro-Leu-Ile-Lys-COOH

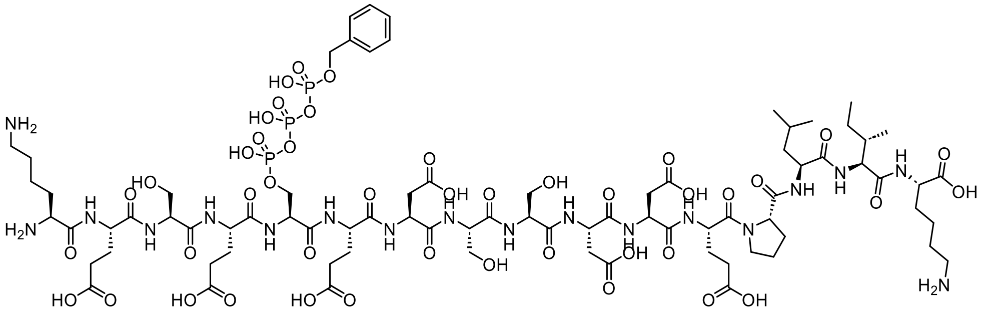

HRMS (ESI): m/z calc'd. for $C_{80}H_{125}N_{18}O_{44}P_3^{2-}$ $[M-2H]^{2-}$ 1067.3660, found 1067.3671.

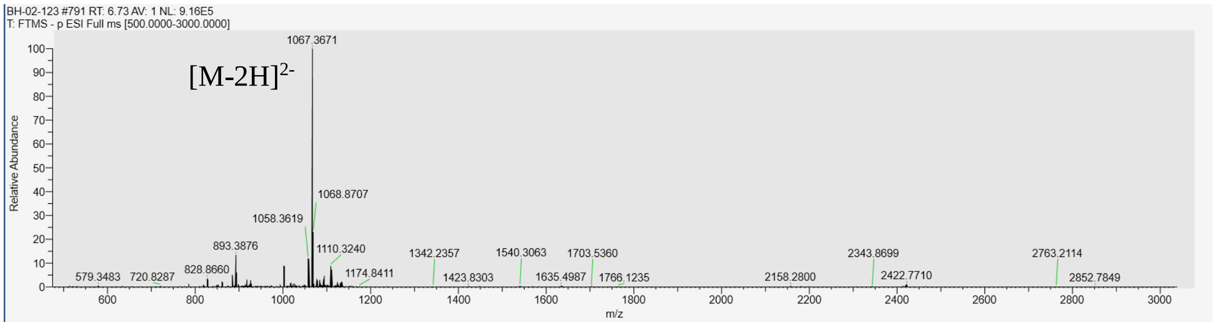

Figure S137: HRMS spectrum of **Bn-p$_3$-Pep5**.

p_3-Pep5

H_2N-Lys-Glu-Ser-Glu-[pppSer]-Glu-Asp-Ser-Ser-Asp-Asp-Glu-Pro-Leu-Ile-Lys-COOH

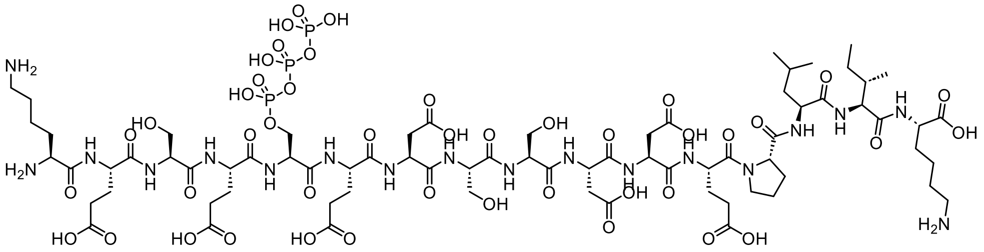

HRMS (ESI): m/z calc'd. for $C_{73}H_{116}AlN_{18}O_{44}P_3^{2-}$ $[M-5H+Al]^{2-}$ 1034.3216, found 1034.3220.

T_R (Method **A**) = 8.750 min.

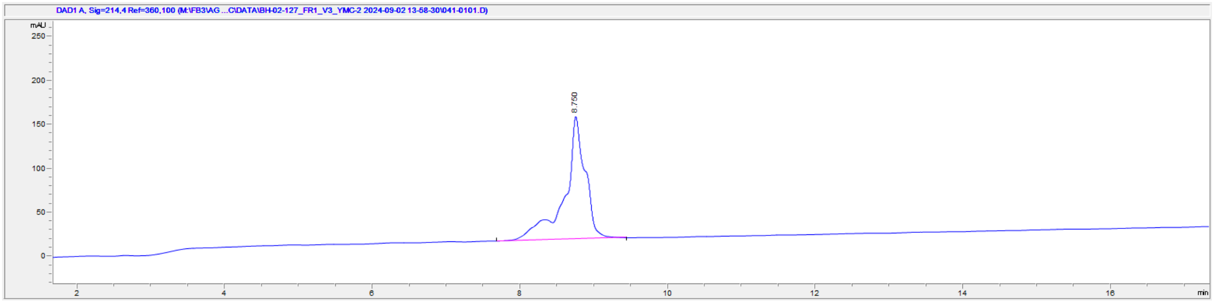

Figure S138: HPLC-UV trace (214 nm) of purified **p_3-Pep5**.

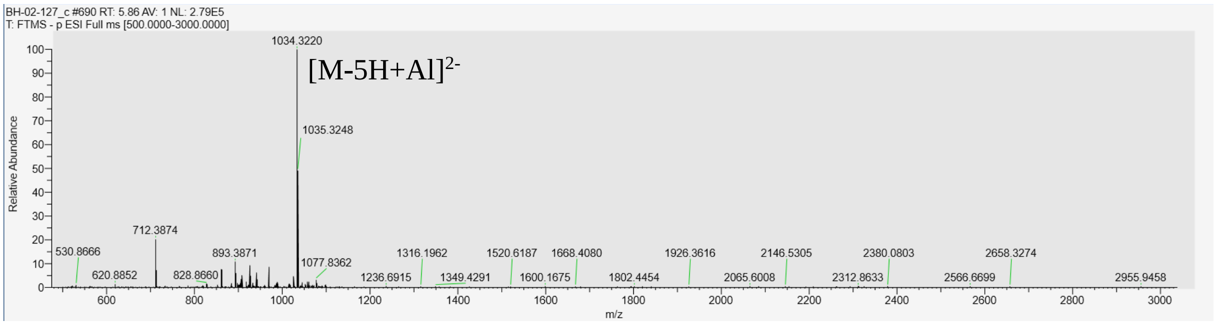

Figure S139: HRMS spectrum of **p_3-Pep5**.

NPE-p$_4$-Pep5

H_2N-Lys-Glu-Ser-Glu-[NPE-ppppSer]-Glu-Asp-Ser-Ser-Asp-Asp-Glu-Pro-Leu-Ile-Lys-COOH

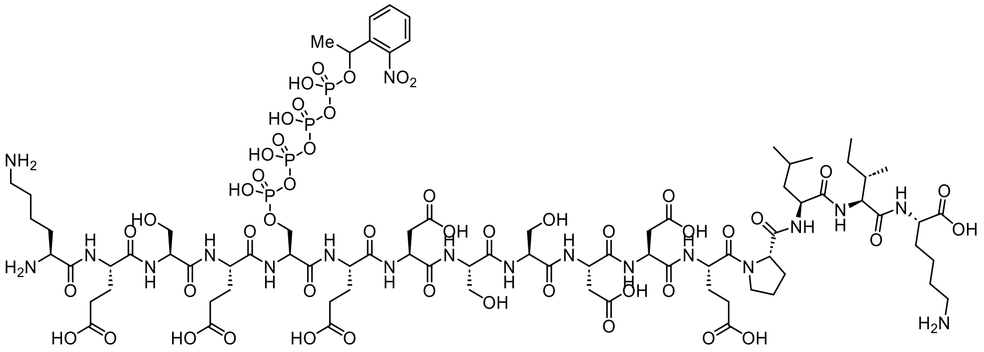

HRMS (ESI): m/z calc'd. for $C_{81}H_{124}AlN_{19}O_{49}P_4^{2-}$ [M-5H+Al]$^{2-}$ 1148.8286, found 1148.8287.
T_R (Method **A**) = 11.045 min.

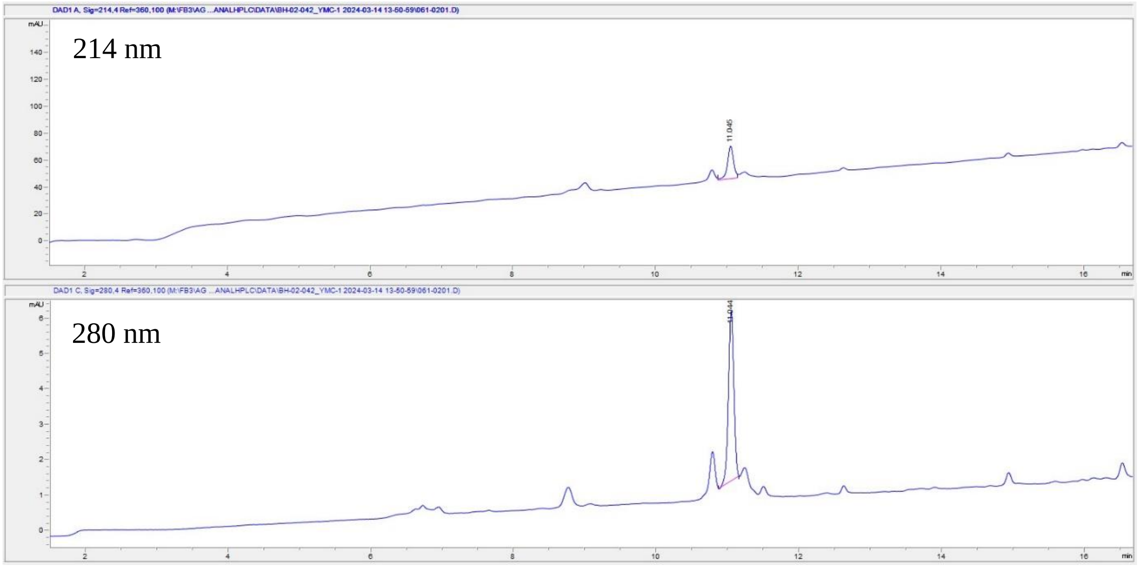

Figure S140: HPLC-UV traces of purified **NPE-p$_4$-Pep5**.

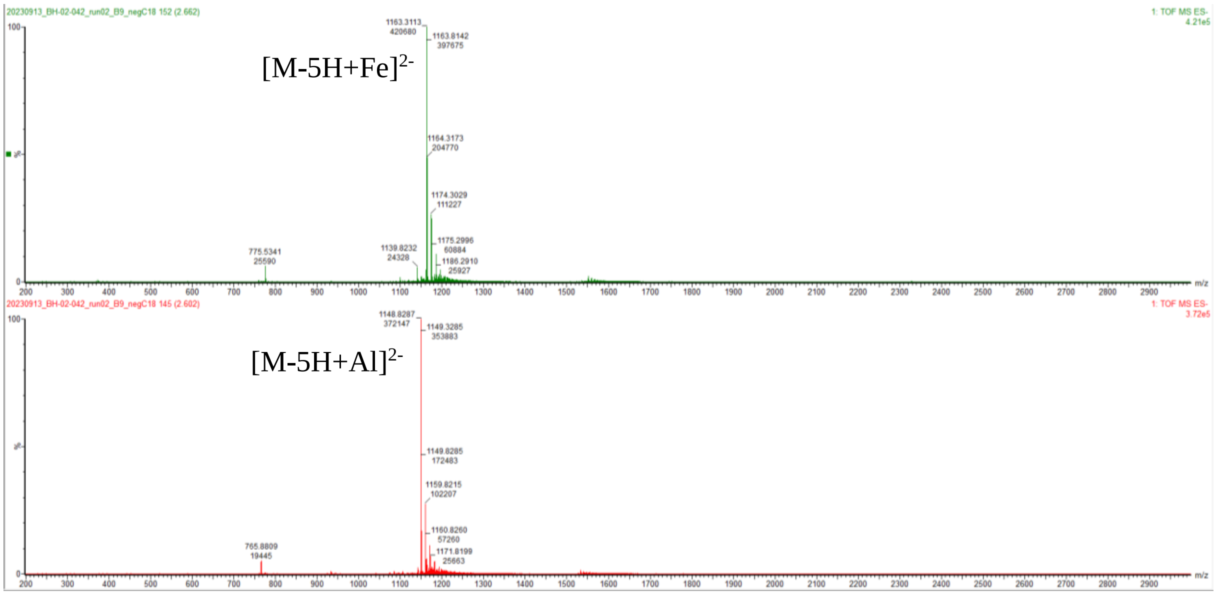

Figure S141: HRMS spectrum of **NPE-p$_4$-Pep5**.

p_4-Pep5

H_2N-Lys-Glu-Ser-Glu-[ppppSer]-Glu-Asp-Ser-Ser-Asp-Asp-Glu-Pro-Leu-Ile-Lys-COOH

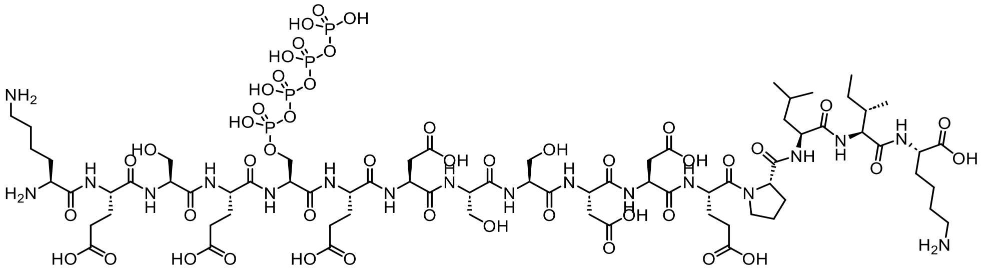

HRMS (ESI): m/z calc'd. for $C_{73}H_{117}AlN_{18}O_{47}P_4^{2-}$ $[M-5H+Al]^{2-}$ 1074.3047, found 1074.3108.
T_R (Method **A**) = 8.978 min.

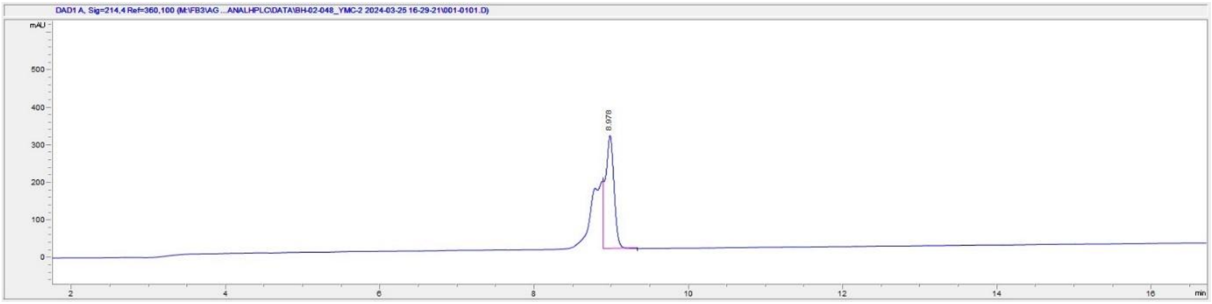

Figure S142: HPLC-UV trace (214 nm) of purified **p_4-Pep5**.

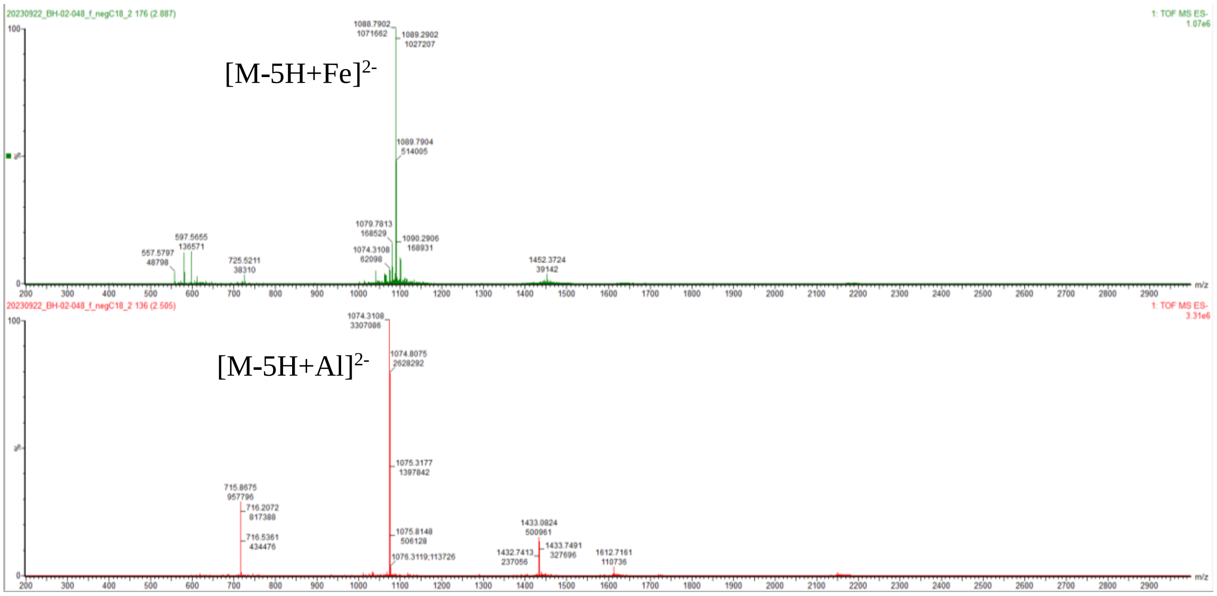

Figure S143: HRMS spectrum of **p_4-Pep5**.

NPE-p$_5$-Pep5

H_2N-Lys-Glu-Ser-Glu-[NPE-pppppSer]-Glu-Asp-Ser-Ser-Asp-Asp-Glu-Pro-Leu-Ile-Lys-COOH

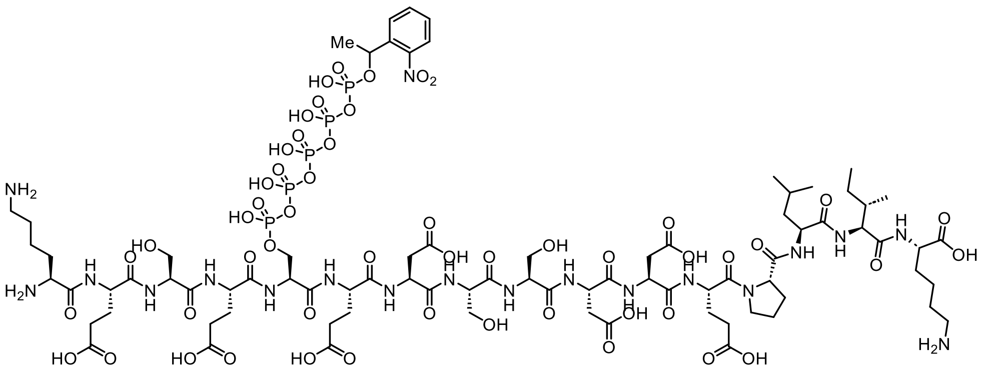

HRMS (ESI): m/z calc'd. for $C_{81}H_{125}AlN_{19}O_{52}P_5$2- $[M-5H+Al]^{2-}$ 1188.8117, found 1188.7987.
T_R (Method **A**) = 10.616 min (Al), 10.692 (Fe).

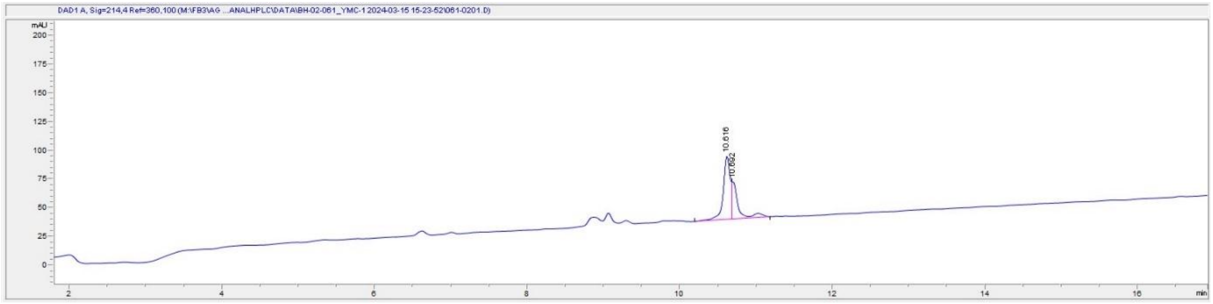

Figure S144: HPLC-UV trace (214 nm) of purified **NPE-p$_5$-Pep5**.

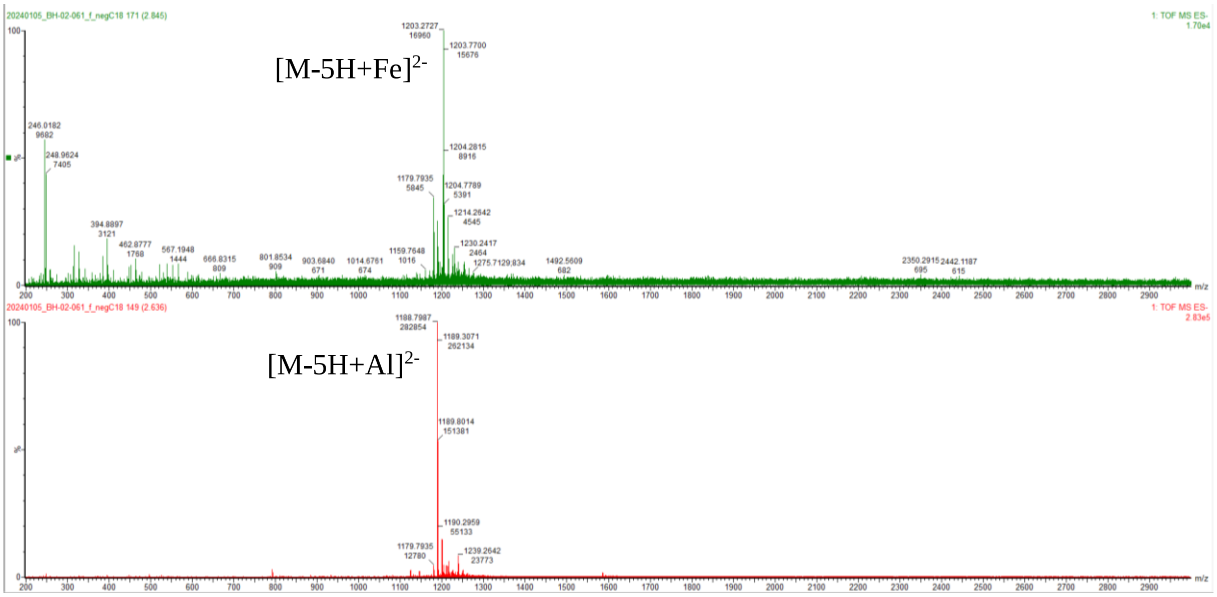

Figure S145: HRMS spectrum of **NPE-p$_5$-Pep5**.

p_5-Pep5

H_2N-Lys-Glu-Ser-Glu-[pppppSer]-Glu-Asp-Ser-Ser-Asp-Asp-Glu-Pro-Leu-Ile-Lys-COOH

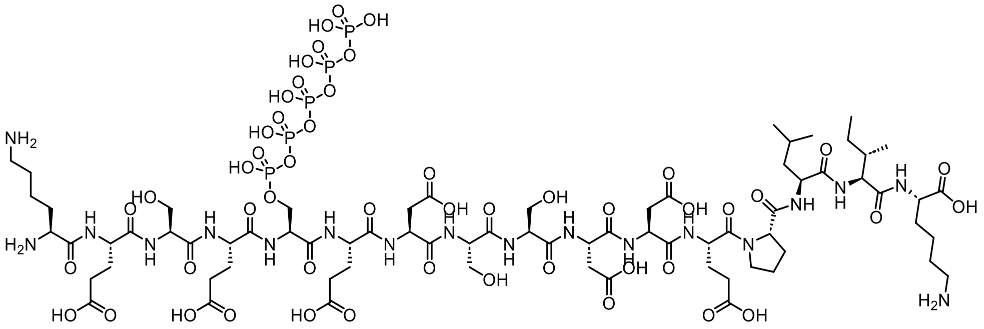

HRMS (ESI): m/z calc'd. for $C_{73}H_{118}AlN_{18}O_{50}P_5^{2-}$ $[M\text{-}5H\text{+}Al]^{2-}$ 1114.2879, found 1114.2813.
T_R (Method **A**) = 8.834 min (Al), 8.998 (Fe).

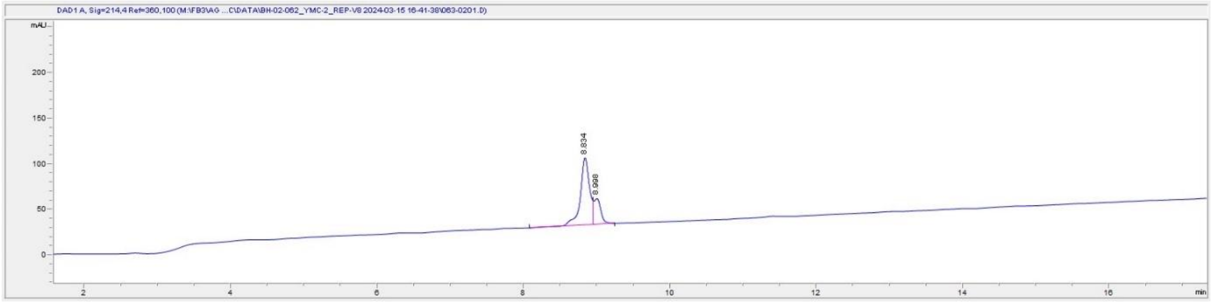

Figure S146: HPLC-UV trace (214 nm) of purified **p_5-Pep5**.

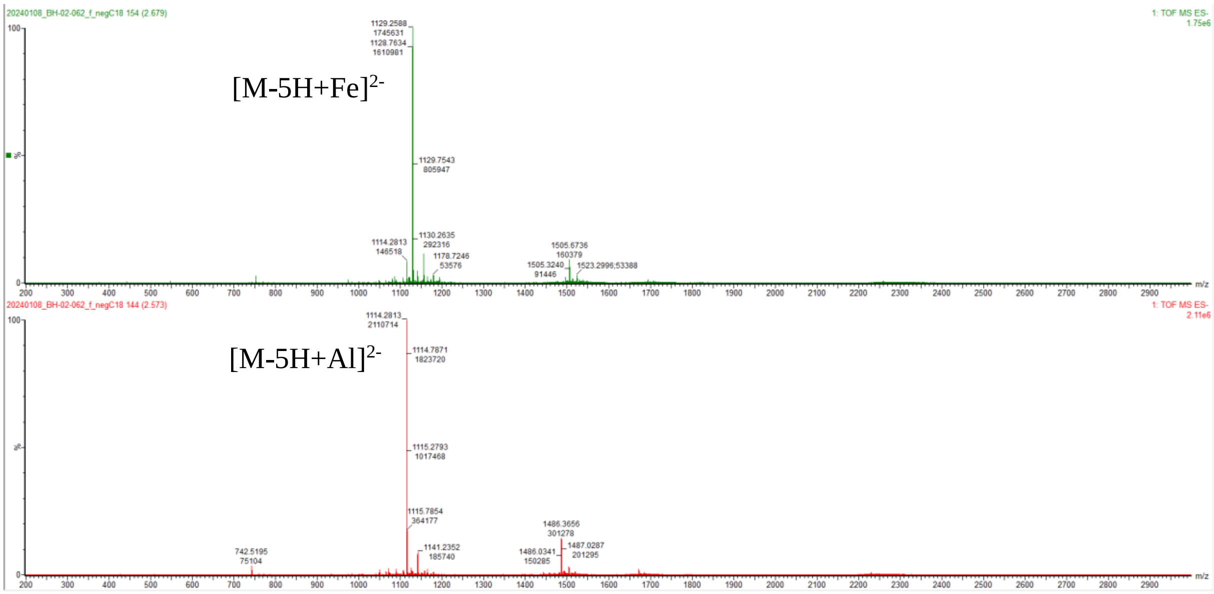

Figure S147: HRMS spectrum of **p_5-Pep5**.

NPE-p$_6$-Pep5

H_2N-Lys-Glu-Ser-Glu-[NPE-ppppppSer]-Glu-Asp-Ser-Ser-Asp-Asp-Glu-Pro-Leu-Ile-Lys-COOH

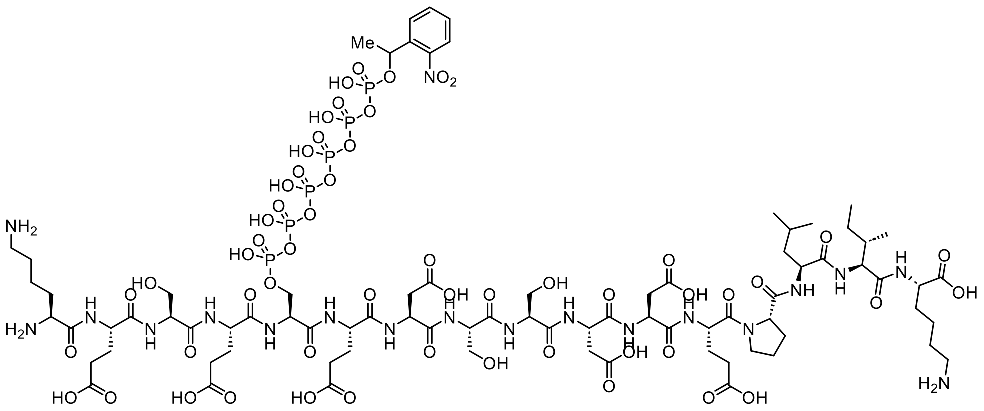

HRMS (ESI): m/z calc'd. for $C_{81}H_{126}AlN_{19}O_{55}P_6^{2-}$ $[M\text{-}5H+Al]^{2-}$ 1228.7949, found 1228.7939.

T_R (Method **A**) = 10.314 min.

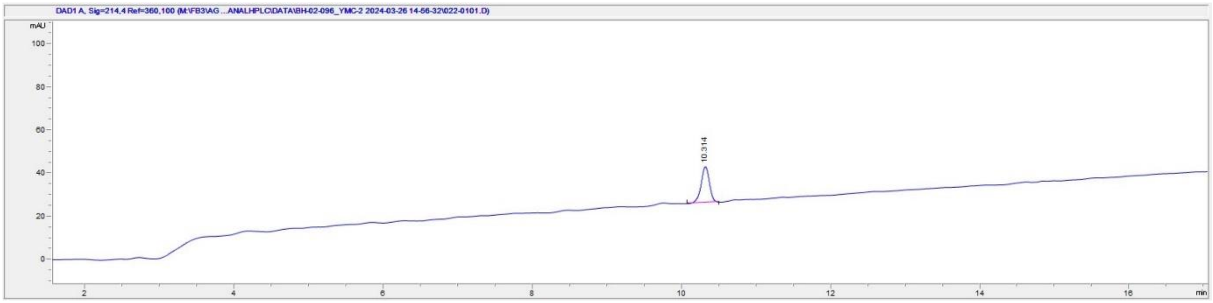

Figure S148: HPLC-UV trace (214 nm) of purified **NPE-p$_6$-Pep5**.

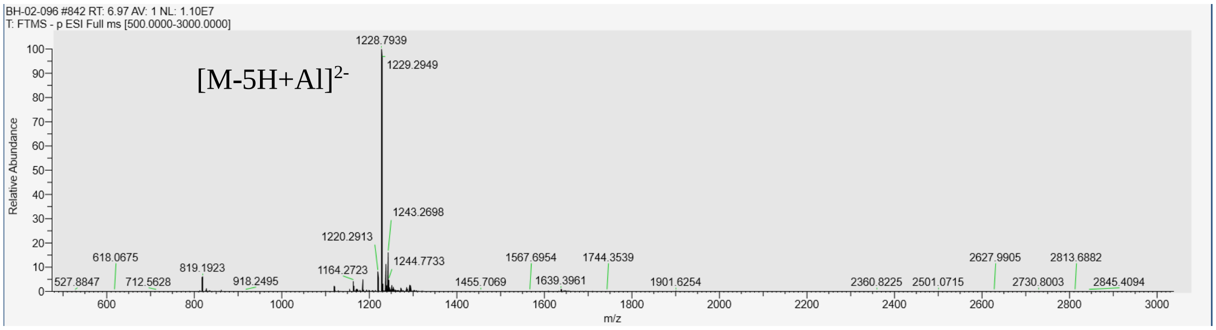

Figure S149: HRMS spectrum of **NPE-p$_6$-Pep5**.

p_6-Pep5
H_2N-Lys-Glu-Ser-Glu-[ppppppSer]-Glu-Asp-Ser-Ser-Asp-Asp-Glu-Pro-Leu-Ile-Lys-COOH

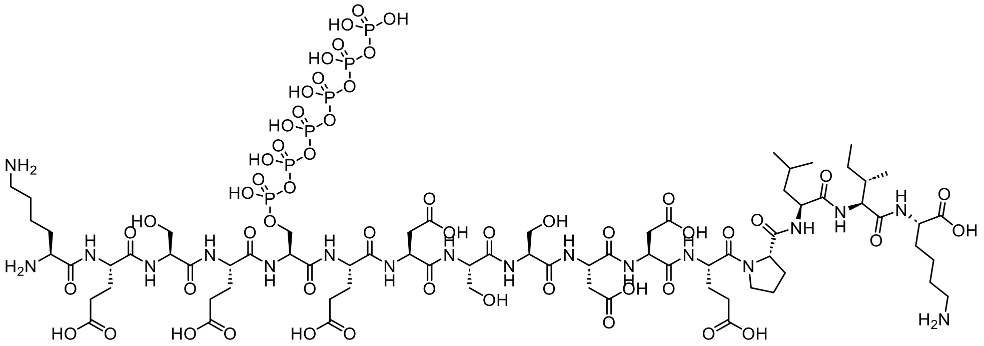

HRMS (ESI): m/z calc'd. for $C_{73}H_{119}AlN_{18}O_{53}P_6^{2-}$ $[M\text{-}5H+Al]^{2-}$ 1154.2711, found 1154.2690.
T_R (Method **A**) = 8.352 min.

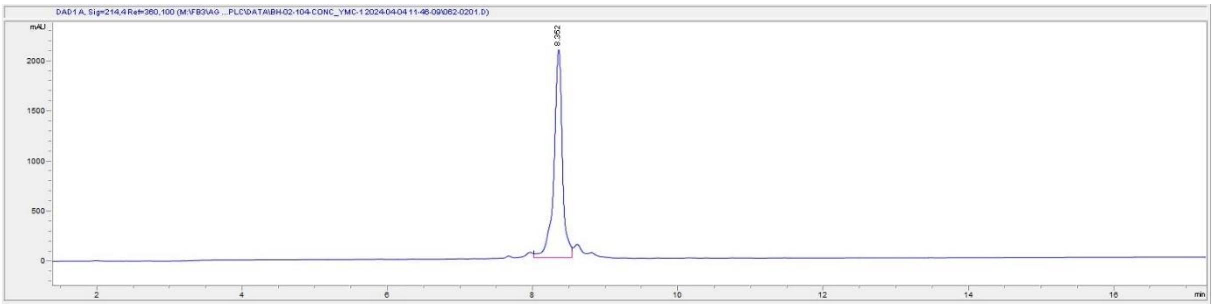

Figure S150: HPLC-UV trace (214 nm) of purified **p_6-Pep5**.

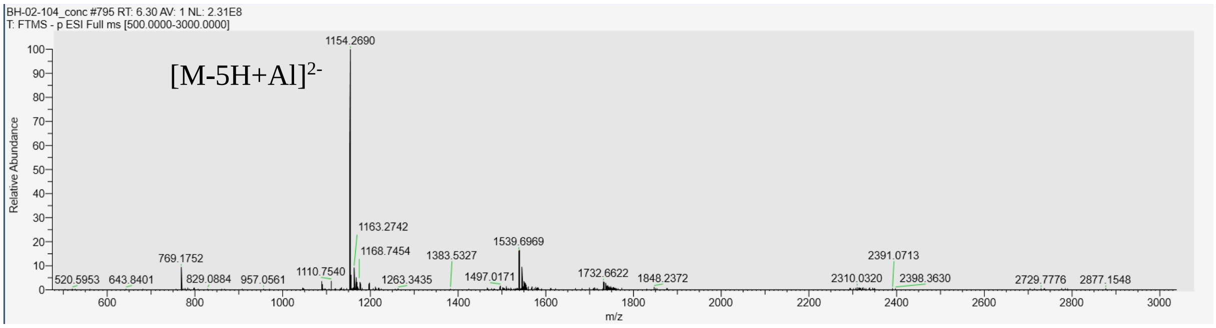

Figure S151: HRMS spectrum of **p_6-Pep5**.

6.6 NPM1 (Ser125) Peptides

p-Pep6

H_2N-Val-Glu-Glu-Asp-Ala-Glu-[pSer]-Glu-Asp-Glu-Glu-Glu-Glu-Asp-Val-Lys-COOH

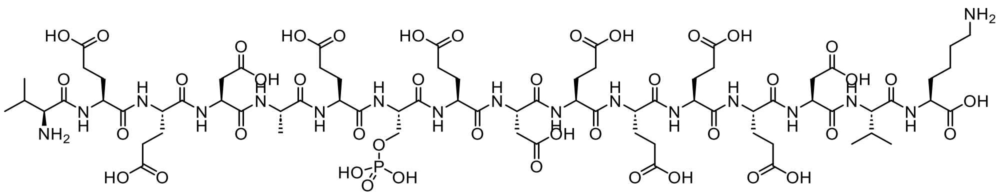

HRMS (ESI): m/z calc'd. for $C_{74}H_{112}N_{17}O_{43}P^{2-}$ $[M-2H]^{2-}$ 978.8424, found 978.8442.
T_R (Method **A**) = 6.750 min.

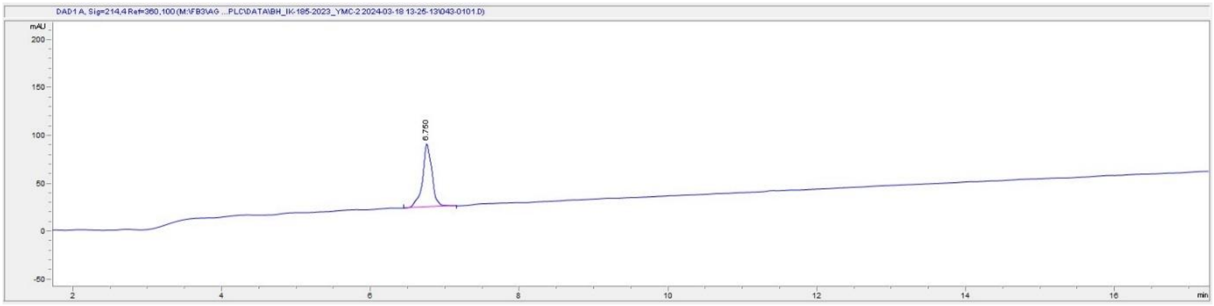

Figure S152: HPLC-UV trace (214 nm) of purified **p-Pep6**.

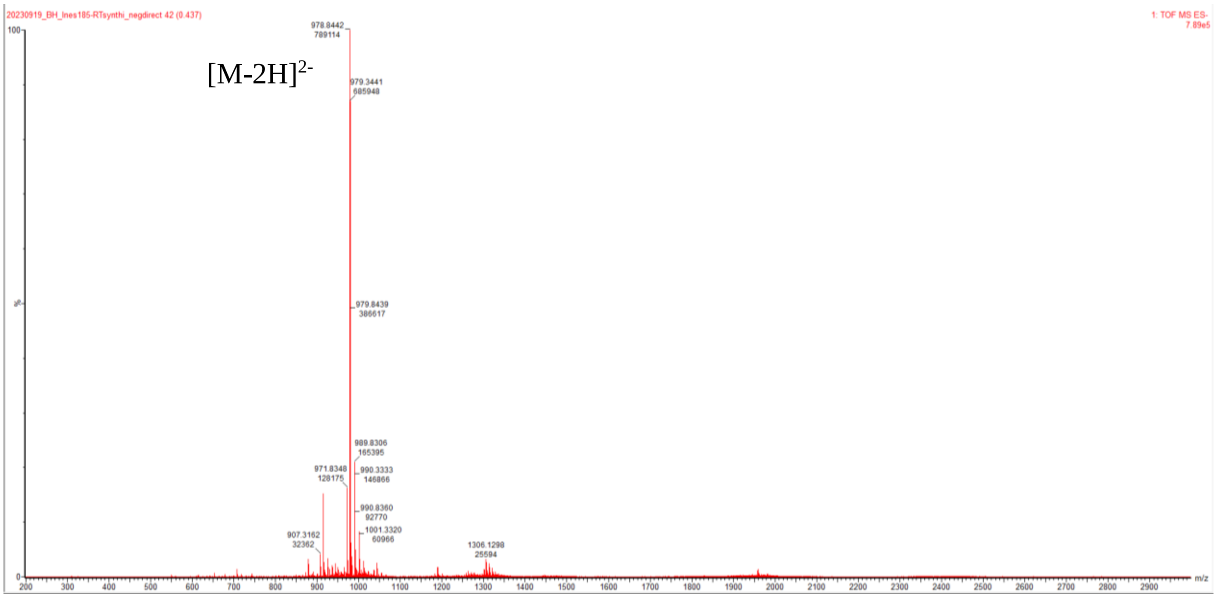

Figure S153: HRMS spectrum of **p-Pep6**.

NPE-p$_2$-Pep6

H_2N-Val-Glu-Glu-Asp-Ala-Glu-[NPE-ppSer]-Glu-Asp-Glu-Glu-Glu-Glu-Asp-Val-Lys-COOH

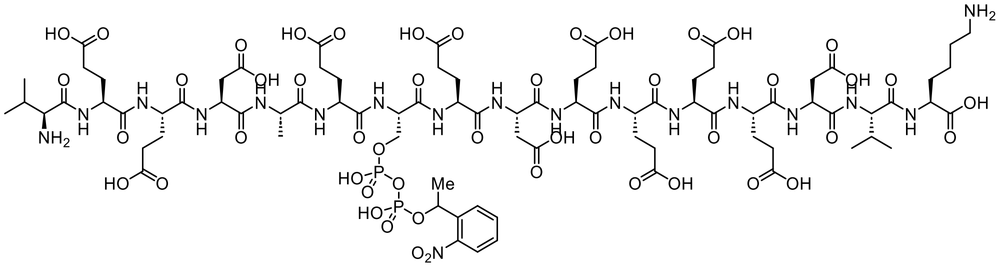

HRMS (ESI): m/z calc'd. for $C_{82}H_{120}N_{18}O_{48}P_2^{2-}$ $[M-2H]^{2-}$ 1093.3494, found 1093.3491.
T_R (Method **A**) = 8.892 min.

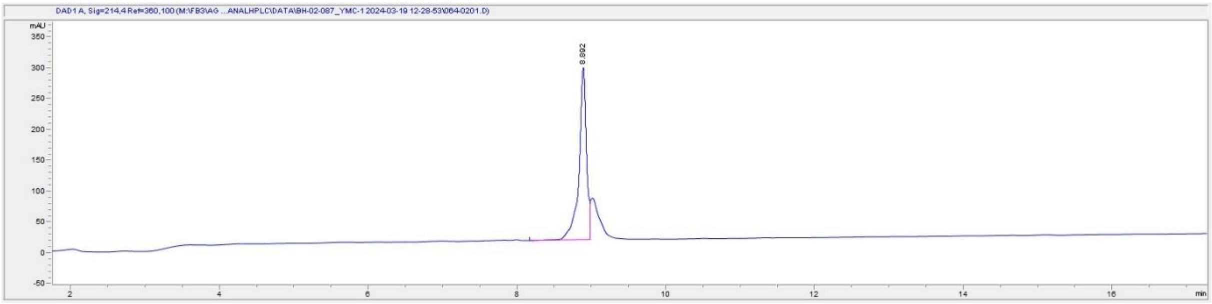

Figure S154: HPLC-UV trace (214 nm) of purified **NPE-p$_2$-Pep6**.

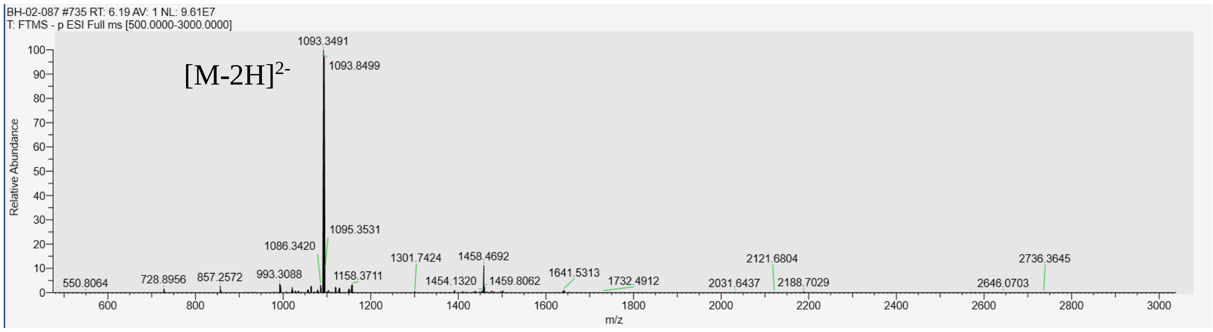

Figure S155: HRMS spectrum of **NPE-p$_2$-Pep6**.

p_2-Pep6
H_2N-Val-Glu-Glu-Asp-Ala-Glu-[ppSer]-Glu-Asp-Glu-Glu-Glu-Glu-Asp-Val-Lys-COOH

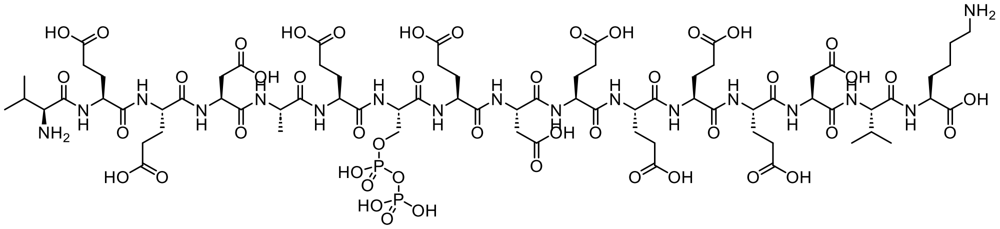

HRMS (ESI): m/z calc'd. for $C_{74}H_{113}N_{17}O_{46}P_2^{2-}$ $[M-2H]^{2-}$ 1018.8256, found 1018.8260.
T_R (Method **A**) = 6.446 min.

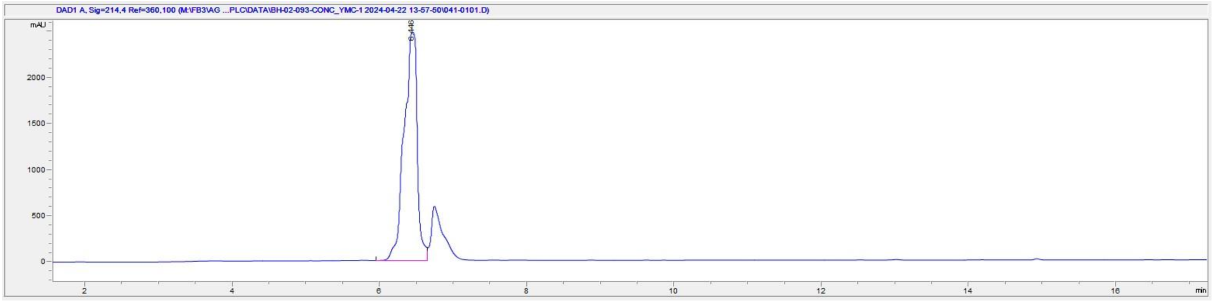

Figure S156: HPLC-UV trace (214 nm) of purified **p_2-Pep6**.

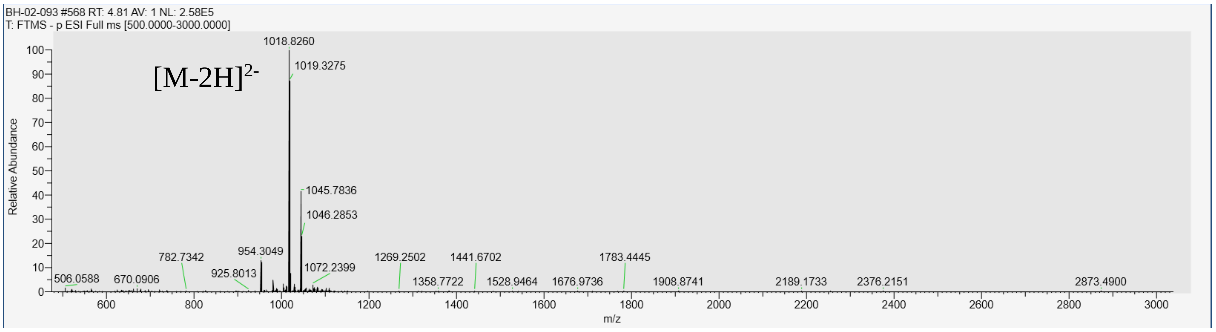

Figure S157: HRMS spectrum of **p_2-Pep6**.

Bn-p$_3$-Pep6

H$_2$N-Val-Glu-Glu-Asp-Ala-Glu-[Bn-pppSer]-Glu-Asp-Glu-Glu-Glu-Glu-Asp-Val-Lys-COOH

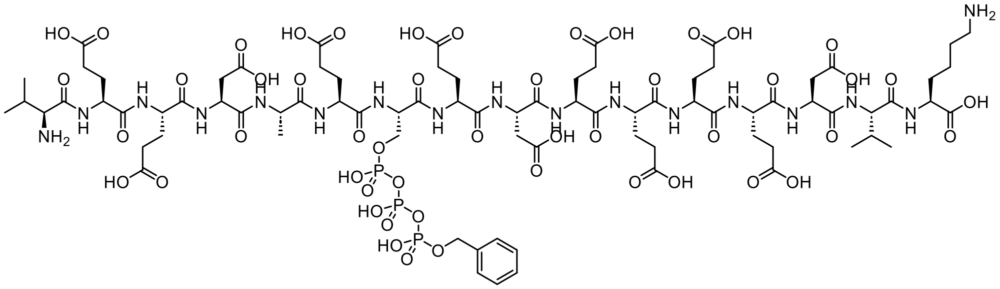

HRMS (ESI): m/z calc'd. for $C_{81}H_{117}FeN_{17}O_{49}P_3^{2-}$ $[M-5H+Fe]^{2-}$ 1130.2876, found 1130.2882.

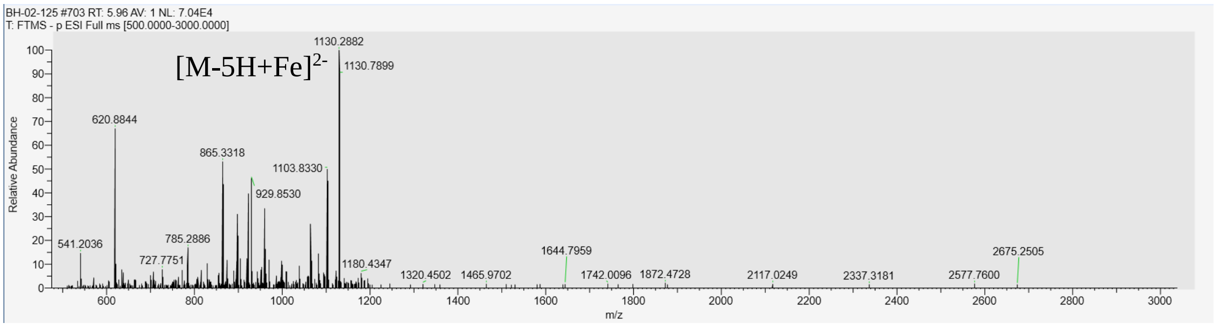

Figure S158: HRMS spectrum of **Bn-p$_3$-Pep6**.

p_3-Pep6
H_2N-Val-Glu-Glu-Asp-Ala-Glu-[pppSer]-Glu-Asp-Glu-Glu-Glu-Glu-Asp-Val-Lys-COOH

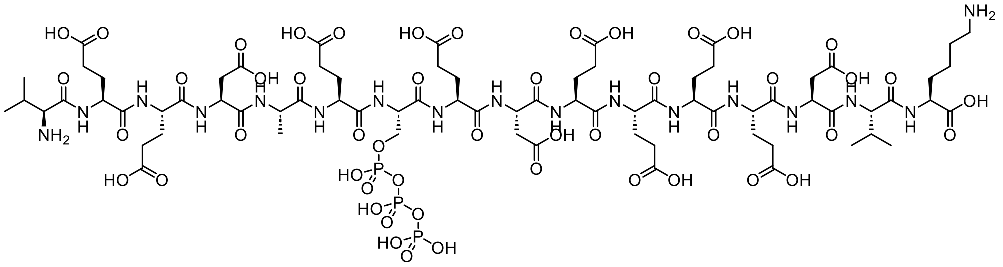

HRMS (ESI): m/z calc'd. for $C_{74}H_{111}AlN_{17}O_{49}P_3^{2-}$ $[M-5H+Al]^{2-}$ 1070.7878, found 1070.7858.
T_R (Method **A**) = 7.411 min.

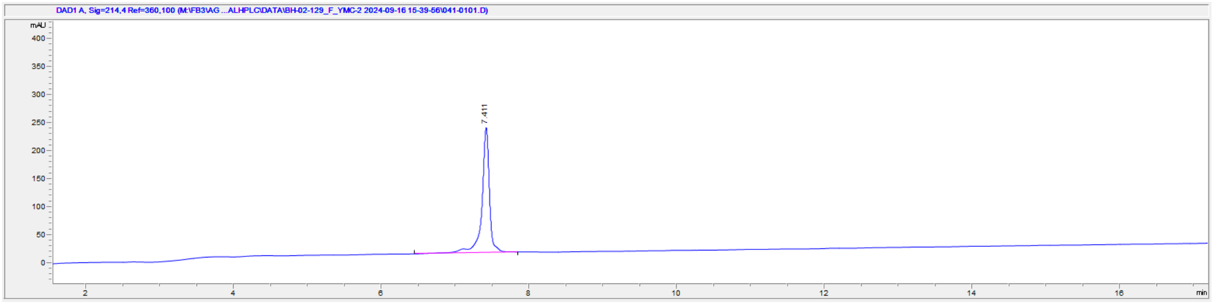

Figure S159: HPLC-UV trace (214 nm) of purified **p_3-Pep6**.

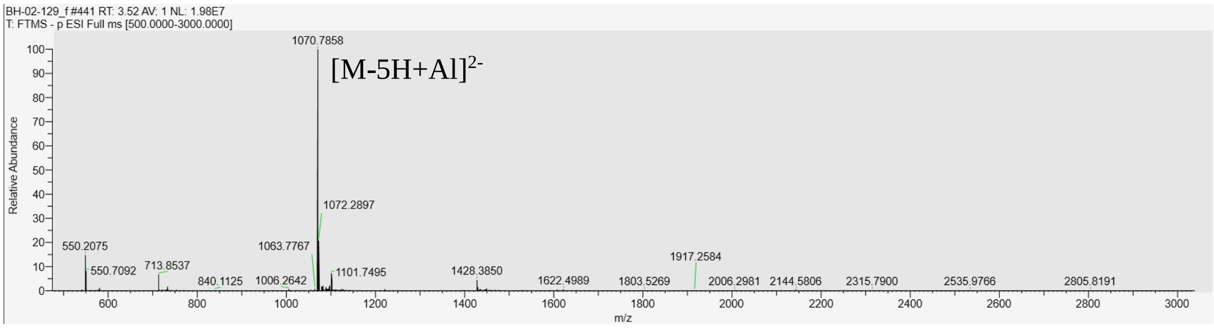

Figure S160: HRMS spectrum of **p_3-Pep6**.

NPE-p$_4$-Pep6

H_2N-Val-Glu-Glu-Asp-Ala-Glu-[NPE-ppppSer]-Glu-Asp-Glu-Glu-Glu-Glu-Asp-Val-Lys-COOH

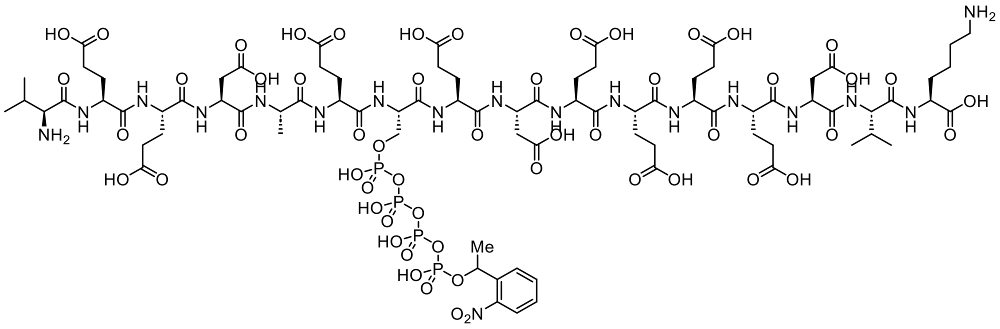

HRMS (ESI): m/z calc'd. for $C_{82}H_{119}FeN_{18}O_{54}P_4^{2-}$ $[M-5H+Fe]^{2-}$ 1199.7712, found 1199.7720.
T_R (Method **A**) = 9.286 min.

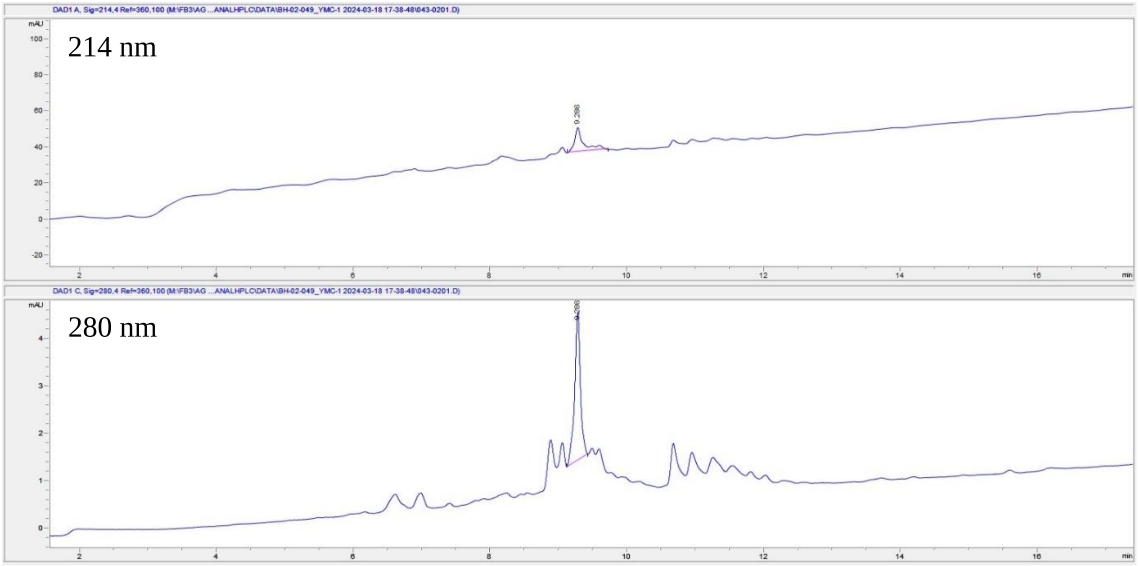

Figure S161: HPLC-UV traces of purified **NPE-p$_4$-Pep6**.

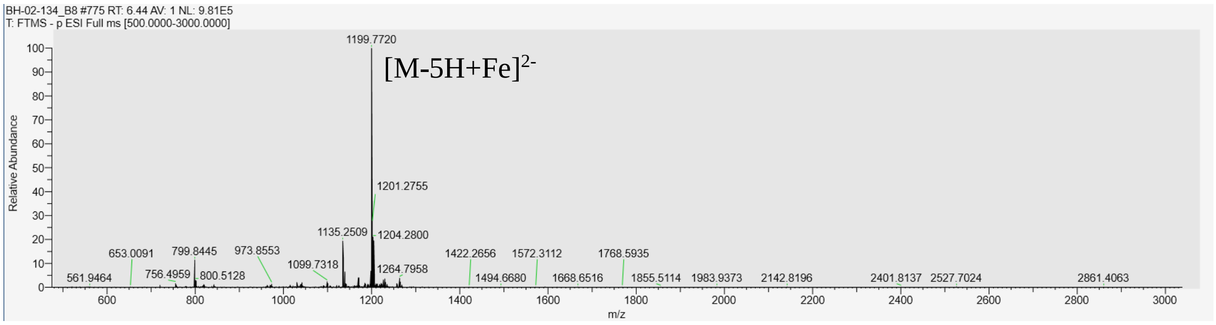

Figure S162: HRMS spectrum of **NPE-p$_4$-Pep6**.

p_4-Pep6

H_2N-Val-Glu-Glu-Asp-Ala-Glu-[ppppSer]-Glu-Asp-Glu-Glu-Glu-Glu-Asp-Val-Lys-COOH

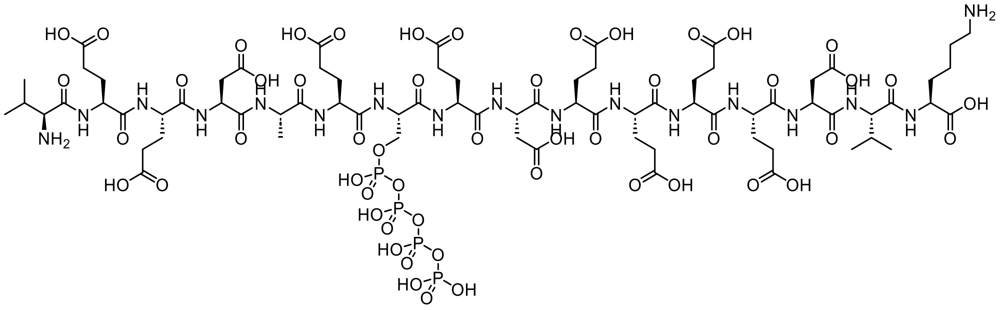

HRMS (ESI): m/z calc'd. for $C_{74}H_{112}AlN_{17}O_{52}P_4^{2-}$ $[M-5H+Al]^{2-}$ 1110.7709, found 1110.7700.

T_R (Method **A**) = 6.599 min.

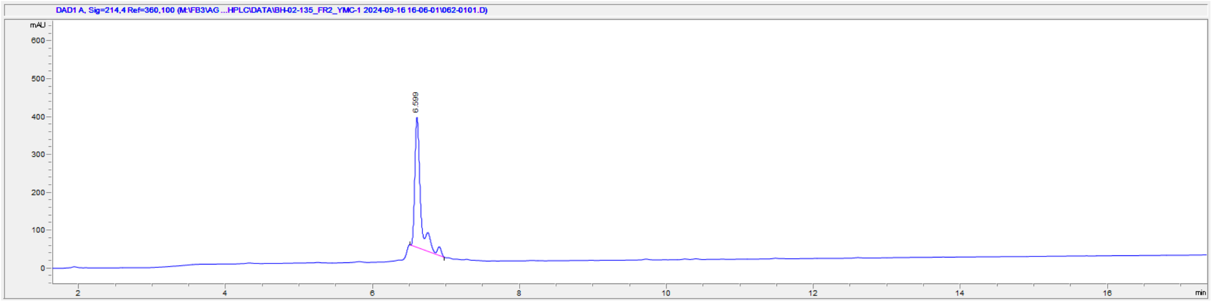

Figure S163: HPLC-UV trace (214 nm) of purified **p_4-Pep6**.

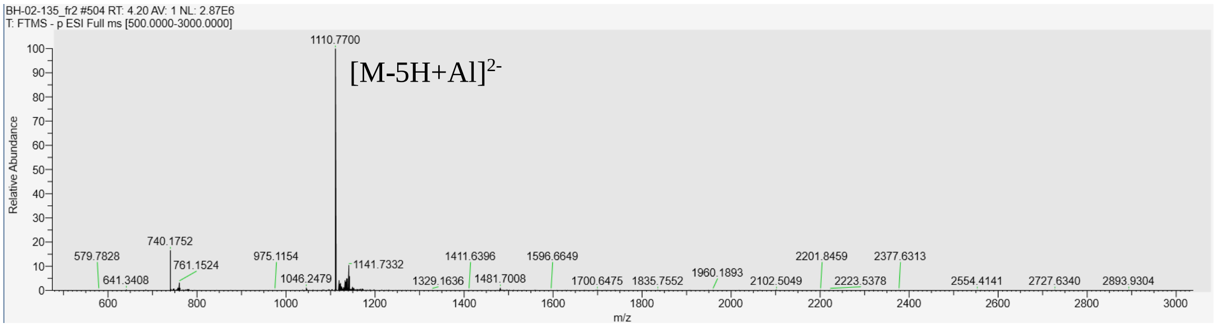

Figure S164: HRMS spectrum of **p_4-Pep6**.

NPE-p$_5$-Pep6

H_2N-Val-Glu-Glu-Asp-Ala-Glu-[NPE-pppppSer]-Glu-Asp-Glu-Glu-Glu-Glu-Asp-Val-Lys-COOH

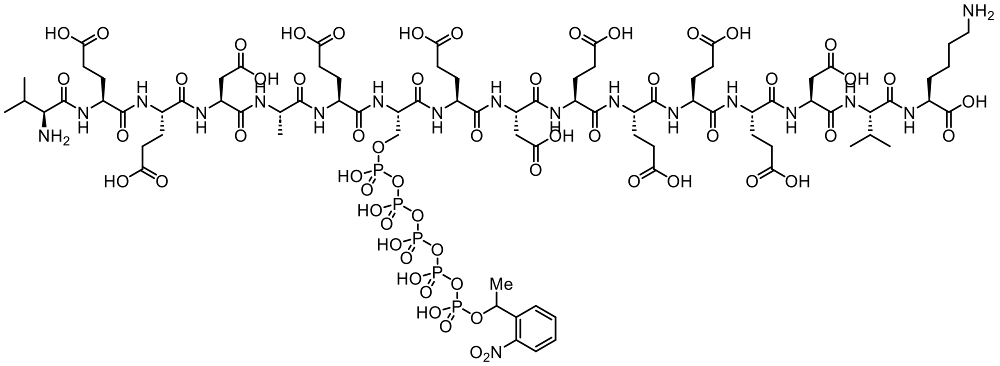

HRMS (ESI): m/z calc'd. for $C_{82}H_{120}AlN_{18}O_{57}P_5^{2-}$ $[M\text{-}5H\text{+}Al]^{2-}$ 1225.2779, found 1225.2627.

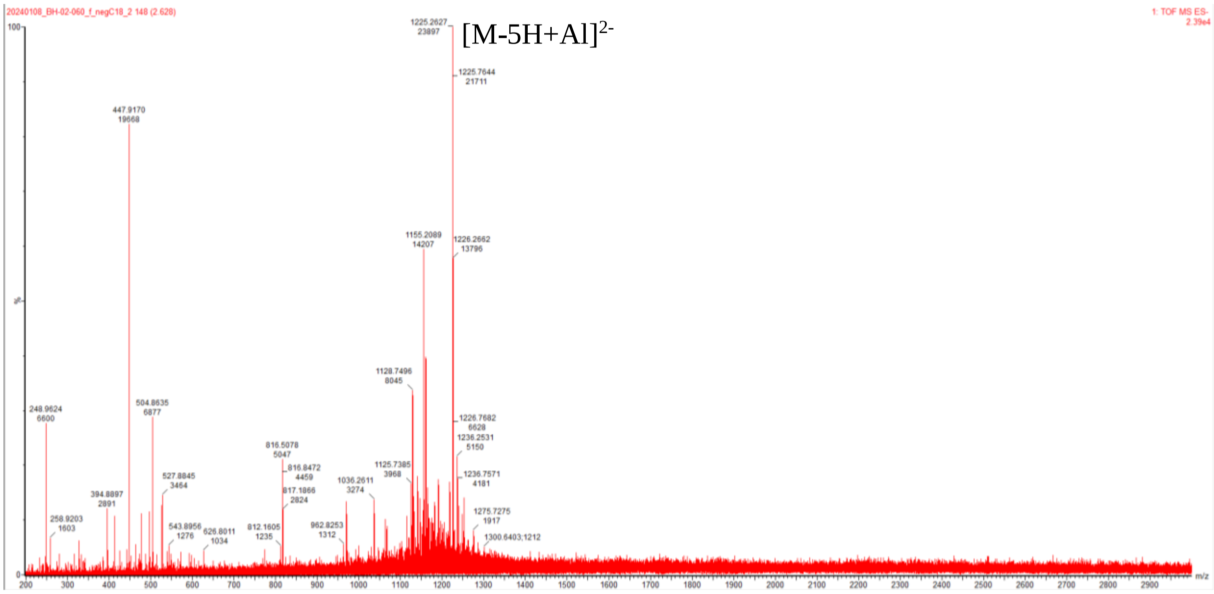

Figure S165: HRMS spectrum of **NPE-p$_5$-Pep6**.

p_5-Pep6

H_2N-Val-Glu-Glu-Asp-Ala-Glu-[pppppSer]-Glu-Asp-Glu-Glu-Glu-Glu-Asp-Val-Lys-COOH

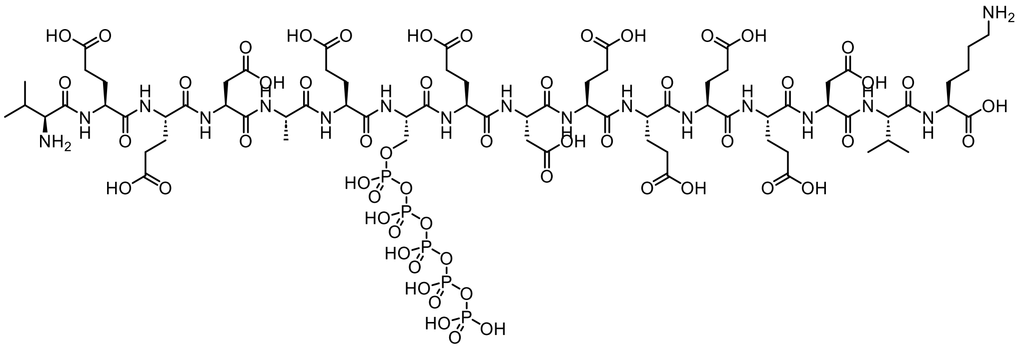

HRMS (ESI): m/z calc'd. for $C_{74}H_{113}AlN_{17}O_{55}P_5^{2-}$ $[M-5H+Al]^{2-}$ 1150.7541, found 1150.7454.
T_R (Method **A**) = 6.543 min.

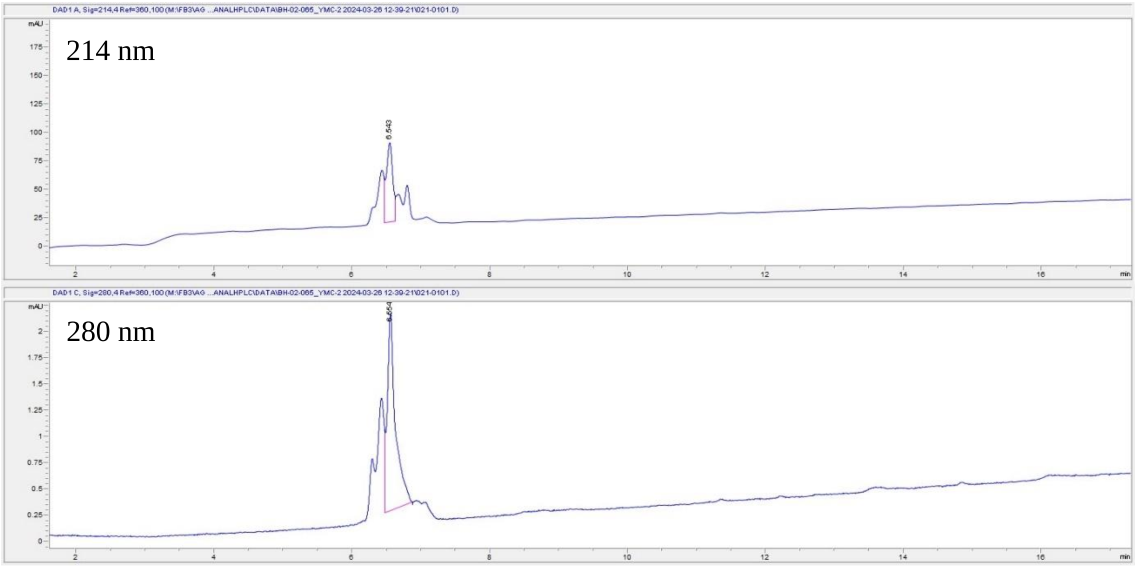

Figure S166: HPLC-UV traces of purified **p_5-Pep6**.

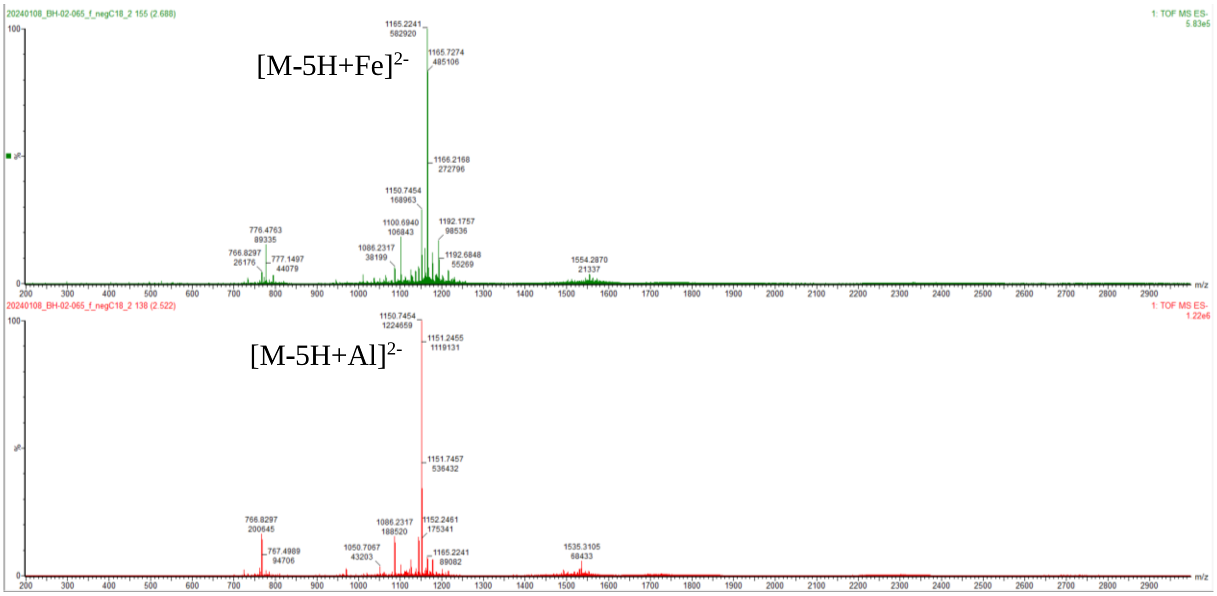

Figure S167: HRMS spectrum of **p_5-Pep6**.

NPE-p$_6$-Pep6
H_2N-Val-Glu-Glu-Asp-Ala-Glu-[NPE-ppppppSer]-Glu-Asp-Glu-Glu-Glu-Glu-Asp-Val-Lys-COOH

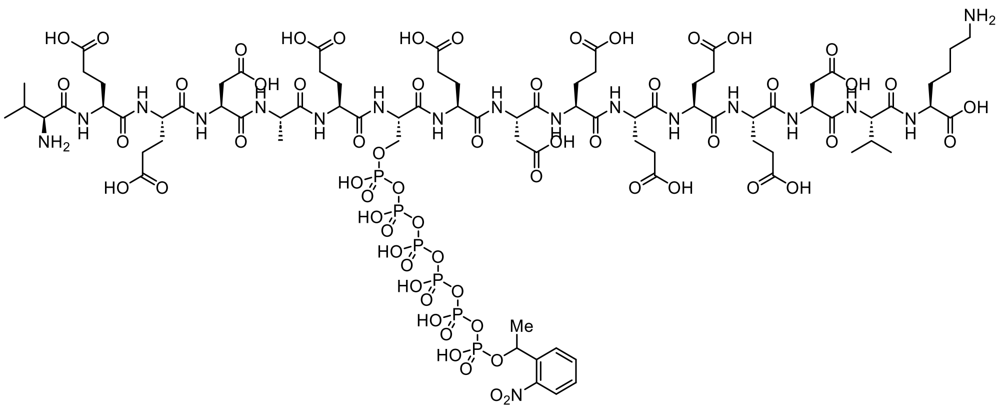

HRMS (ESI): m/z calc'd. for $C_{82}H_{121}AlN_{18}O_{60}P_6^{2-}$ $[M-5H+Al]^{2-}$ 1265.2611, found 1265.2621.
T_R (Method **A**) = 8.073 min.

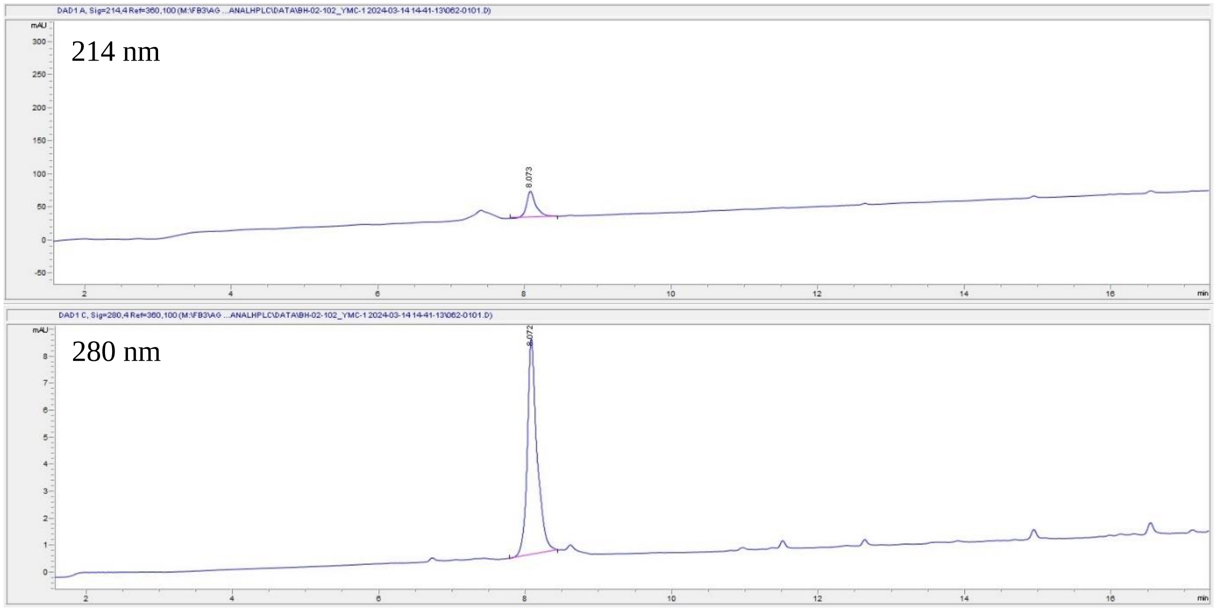

Figure S168: HPLC-UV traces of purified **NPE-p$_6$-Pep6**.

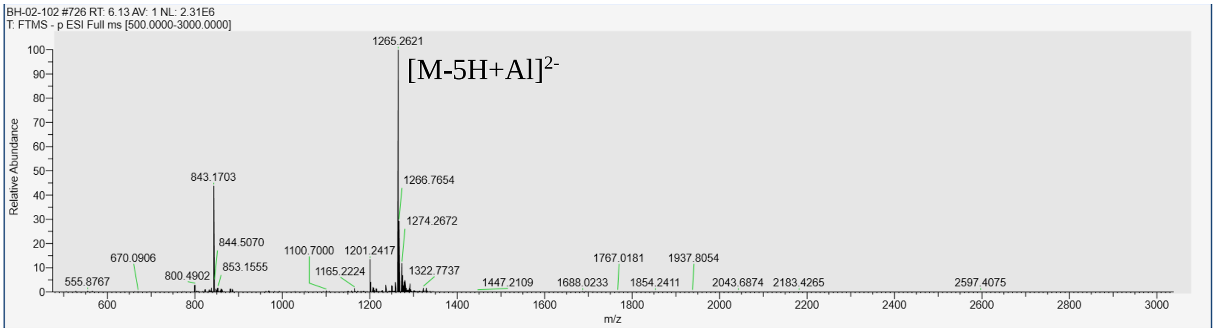

Figure S169: HRMS spectrum of **NPE-p$_6$-Pep6**.

p_6-Pep6

H_2N-Val-Glu-Glu-Asp-Ala-Glu-[ppppppSer]-Glu-Asp-Glu-Glu-Glu-Glu-Asp-Val-Lys-COOH

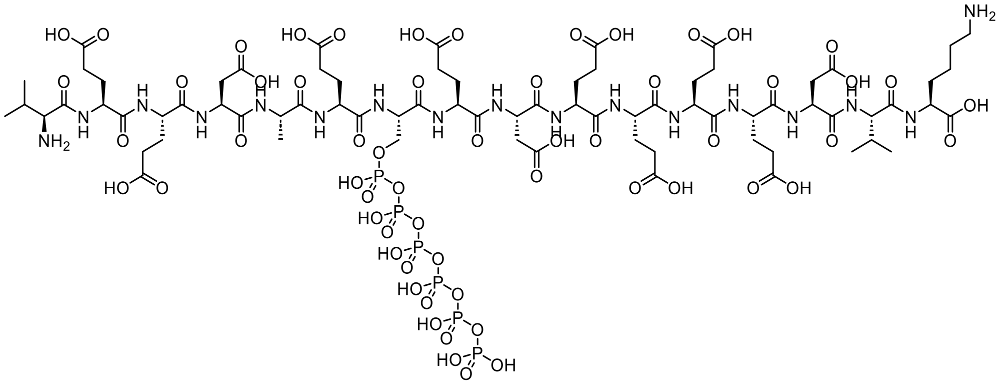

HRMS (ESI): m/z calc'd. for $C_{74}H_{114}AlN_{17}O_{58}P_6^{2-}$ $[M-5H+Al]^{2-}$ 1190.7373, found 1190.7355.
T_R (Method **A**) = 6.025 min (Al), 6.452 (Fe).

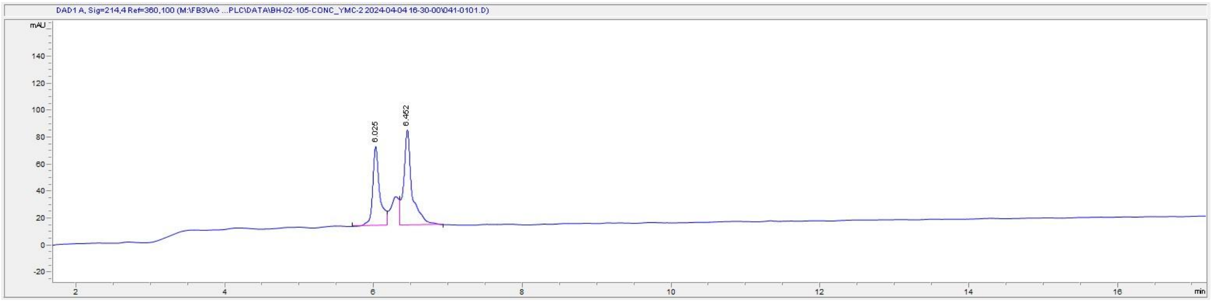

Figure S170: HPLC-UV trace (214 nm) of purified **p_6-Pep6**.

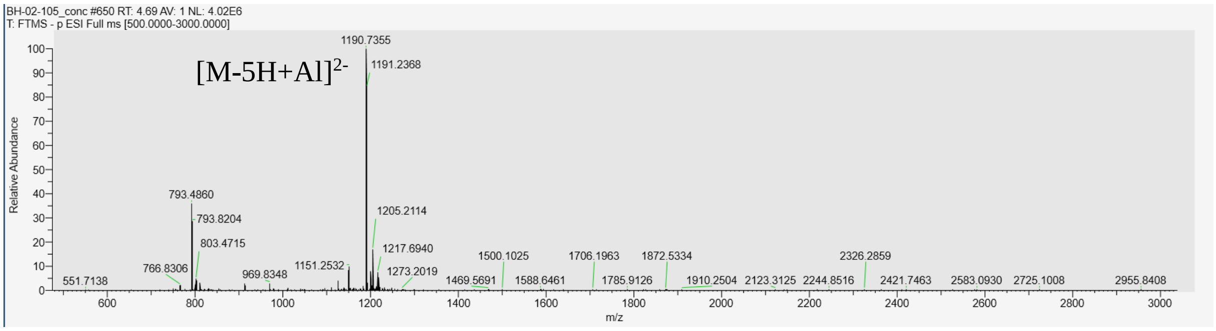

Figure S171: HRMS spectrum of **p_6-Pep6**.

6.7 NOLC1 (Ser84) Peptides

p-Pep7

H_2N-Lys-Lys-Ala-Ser-[pSer]-Ser-Asp-Ser-Glu-Asp-Ser-Ser-Glu-Glu-Glu-COOH

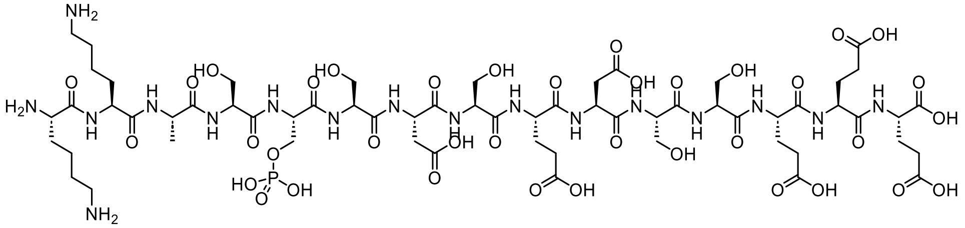

HRMS (ESI): m/z calc'd. for $C_{61}H_{98}N_{17}O_{37}P^{2-}$ $[M-2H]^{2-}$ 845.8029, found 845.7994.
T_R (Method **A**) = 4.292 min.

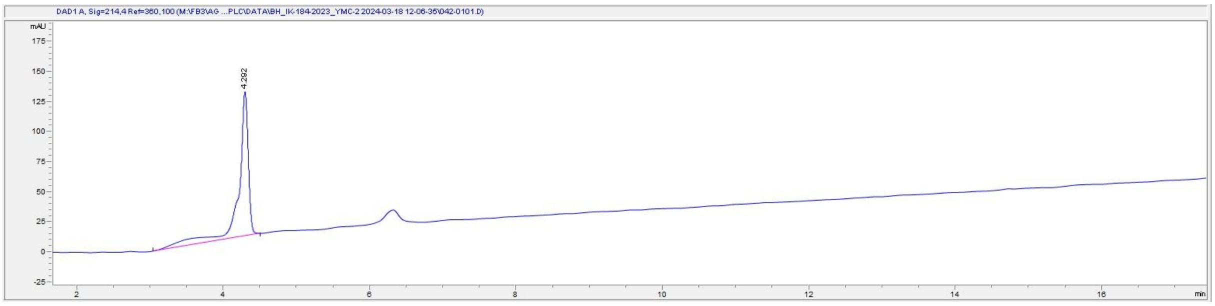

Figure S172: HPLC-UV trace (214 nm) of purified **p-Pep7**.

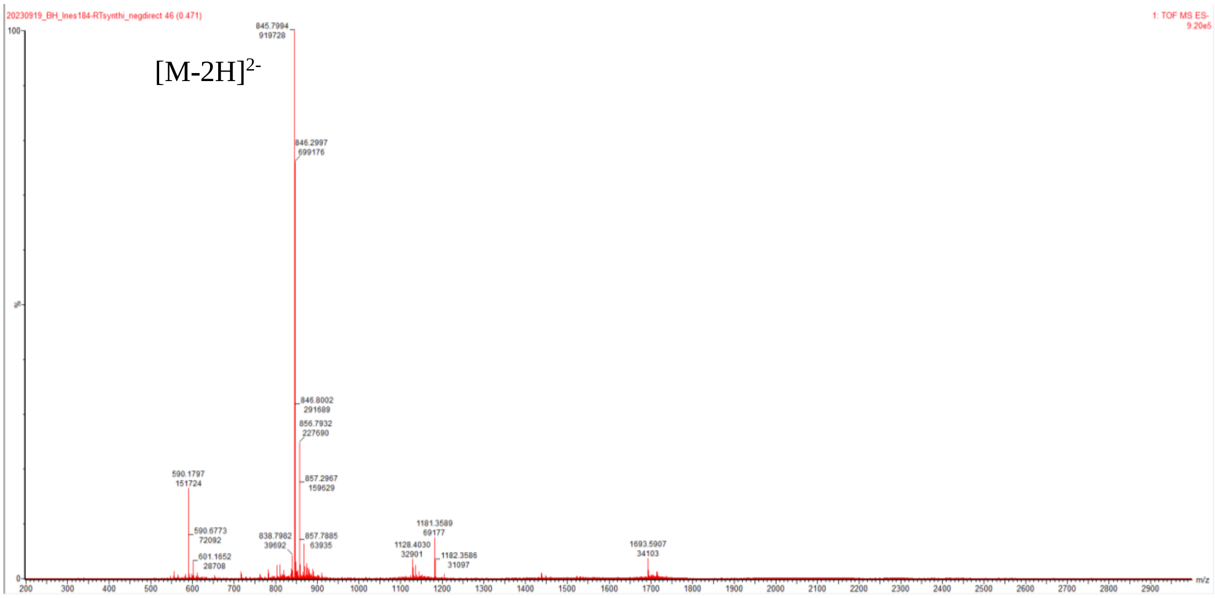

Figure S173: HRMS spectrum of **p-Pep7**.

NPE-p₂-Pep7
H_2N-Lys-Lys-Ala-Ser-[NPE-ppSer]-Ser-Asp-Ser-Glu-Asp-Ser-Ser-Glu-Glu-Glu-COOH

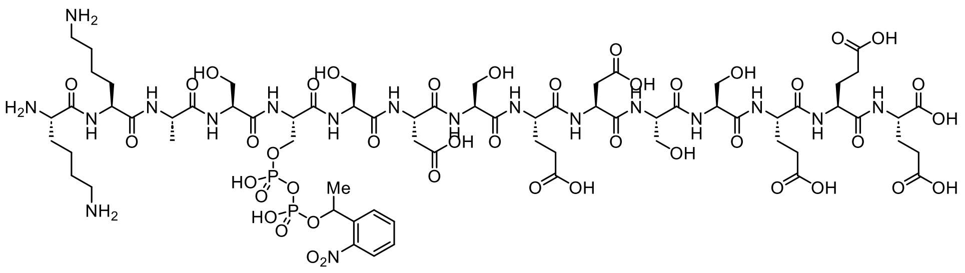

HRMS (ESI): m/z calc'd. for $C_{69}H_{106}N_{18}O_{42}P_2^{2-}$ $[M-2H]^{2-}$ 960.3099, found 960.3098.
T_R (Method **A**) = 7.077 min.

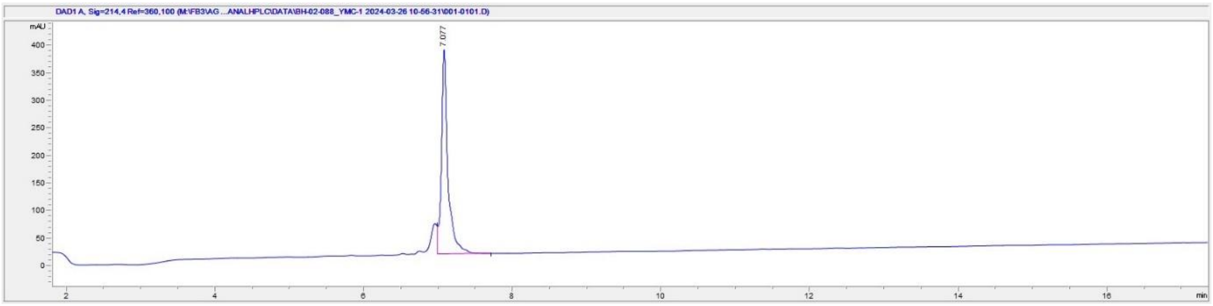

Figure S174: HPLC-UV trace (214 nm) of purified **NPE-p$_2$-Pep7**.

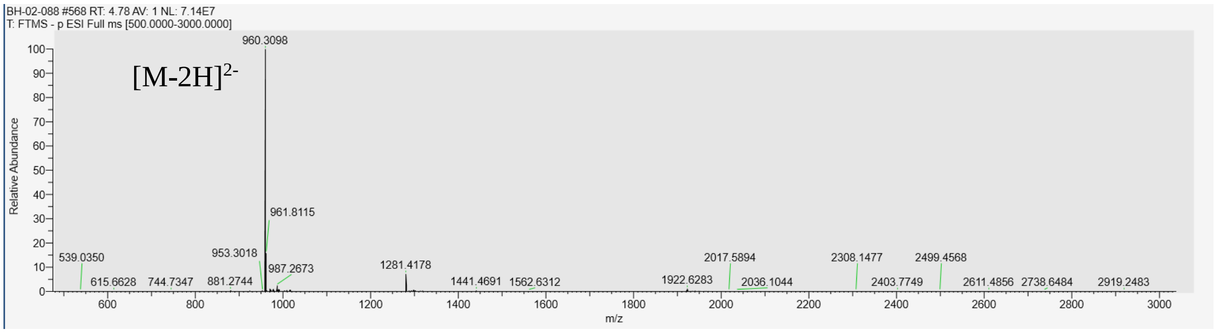

Figure S175: HRMS spectrum of **NPE-p$_2$-Pep7**.

p_2-Pep7

H_2N-Lys-Lys-Ala-Ser-[ppSer]-Ser-Asp-Ser-Glu-Asp-Ser-Ser-Glu-Glu-Glu-COOH

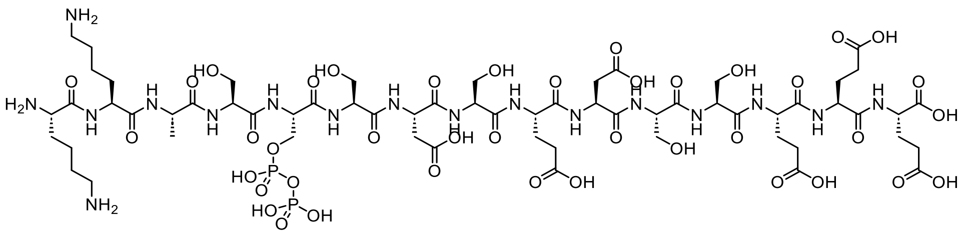

HRMS (ESI): m/z calc'd. for $C_{61}H_{99}N_{17}O_{40}P_2^{2-}$ $[M-2H]^{2-}$ 885.7860, found 885.7864.

T_R (Method **A**) = 6.569 min.

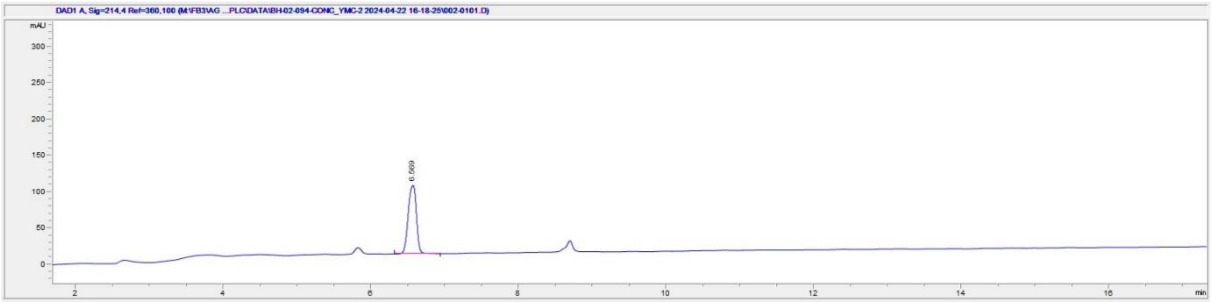

Figure S176: HPLC-UV trace (214 nm) of purified **p_2-Pep7**.

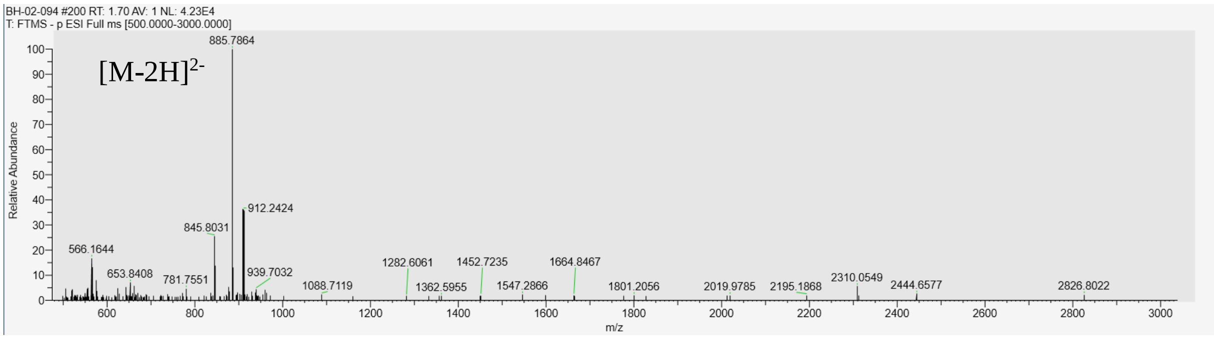

Figure S177: HRMS spectrum of **p_2-Pep7**.

Bn-p$_3$-Pep7

H_2N-Lys-Lys-Ala-Ser-[Bn-pppSer]-Ser-Asp-Ser-Glu-Asp-Ser-Ser-Glu-Glu-Glu-COOH

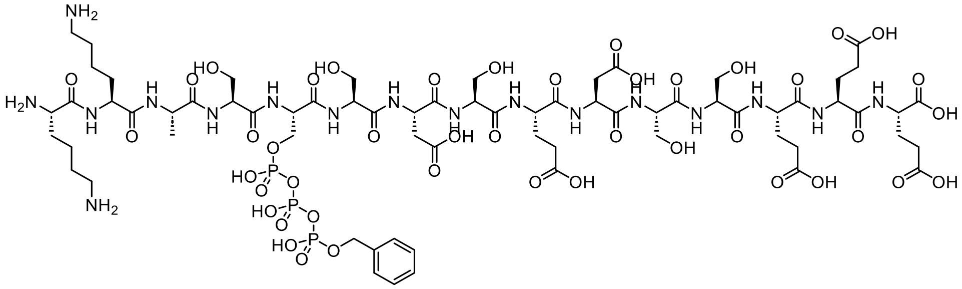

HRMS (ESI): m/z calc'd. for $C_{68}H_{106}N_{17}O_{43}P_3^{2-}$ $[M-2H]^{2-}$ 970.7927, found 970.7927.

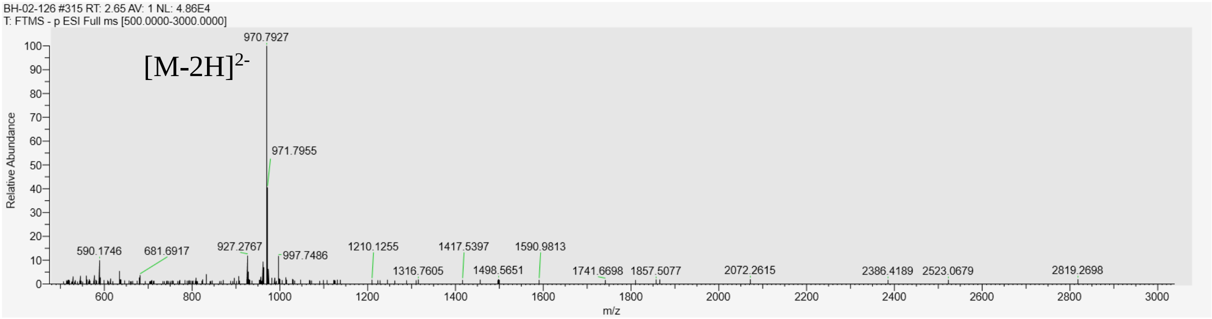

Figure S178: HRMS spectrum of **Bn-p$_3$-Pep7**.

p_3-Pep7

H_2N-Lys-Lys-Ala-Ser-[pppSer]-Ser-Asp-Ser-Glu-Asp-Ser-Ser-Glu-Glu-Glu-COOH

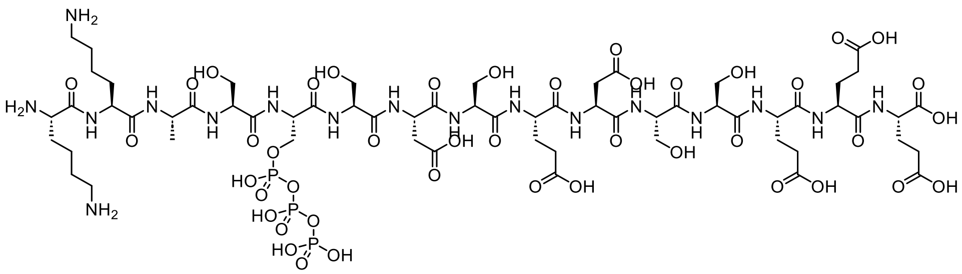

HRMS (ESI): m/z calc'd. for $C_{61}H_{97}AlN_{17}O_{43}P_3^{2-}$ $[M-5H+Al]^{2-}$ 937.7482, found 937.7471.
T_R (Method **A**) = 4.303 min.

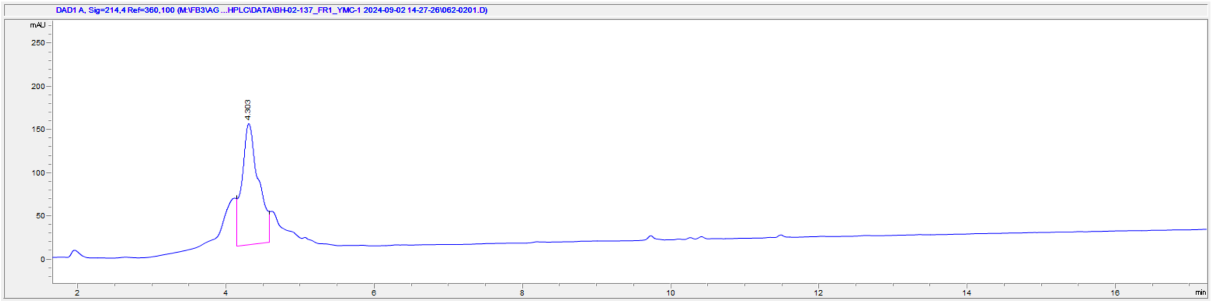

Figure S179: HPLC-UV trace (214 nm) of purified **p_3-Pep7**.

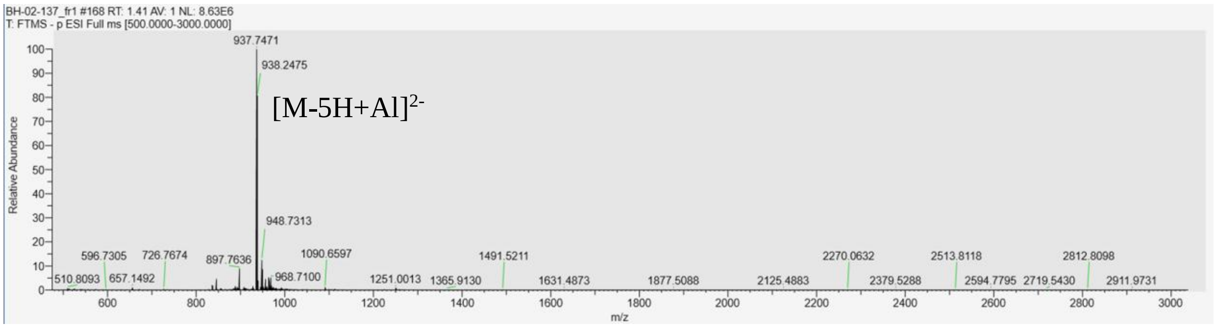

Figure S180: HRMS spectrum of **p_3-Pep7**.

NPE-p$_4$-Pep7

H_2N-Lys-Lys-Ala-Ser-[NPE-ppppSer]-Ser-Asp-Ser-Glu-Asp-Ser-Ser-Glu-Glu-Glu-COOH

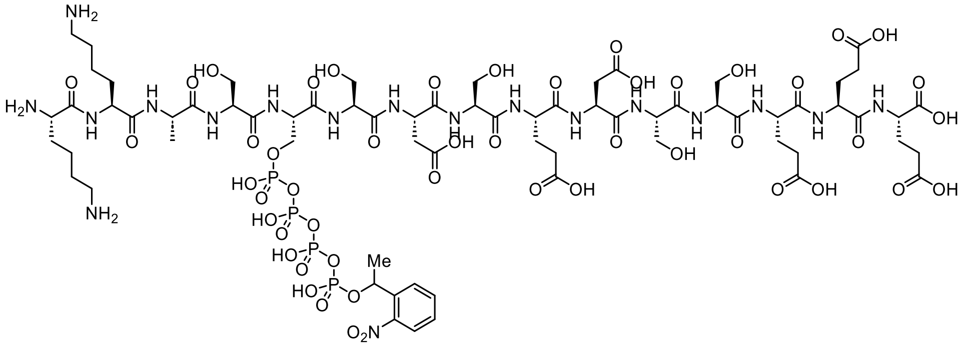

HRMS (ESI): m/z calc'd. for $C_{69}H_{108}N_{18}O_{48}P_4^{2-}$ $[M-2H]^{2-}$ 1040.2762, found 1040.2727.

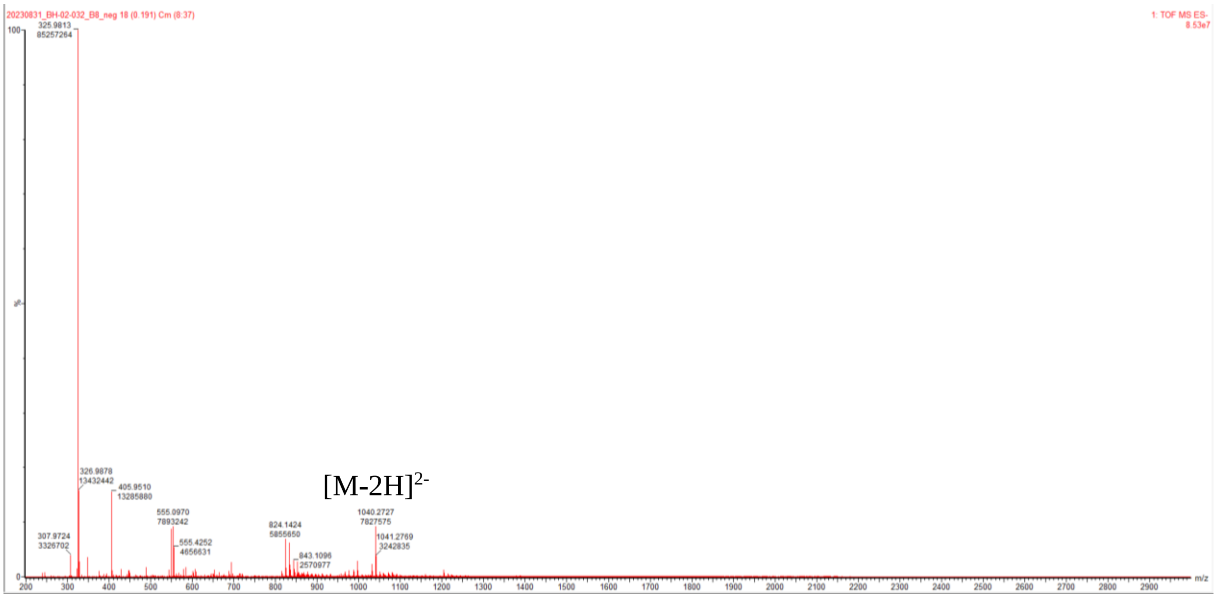

Figure S181: HRMS spectrum of **NPE-p$_4$-Pep7**.

p_4-Pep7

H_2N-Lys-Lys-Ala-Ser-[ppppSer]-Ser-Asp-Ser-Glu-Asp-Ser-Ser-Glu-Glu-Glu-COOH

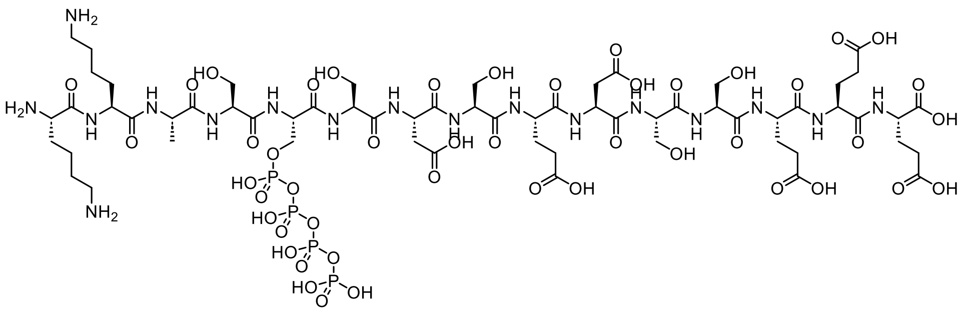

HRMS (ESI): m/z calc'd. for $C_{61}H_{98}AlN_{17}O_{46}P_4^{2-}$ $[M-5H+Al]^{2-}$ 977.7314, found 977.7297.
T_R (Method **A**) = 4.213 min.

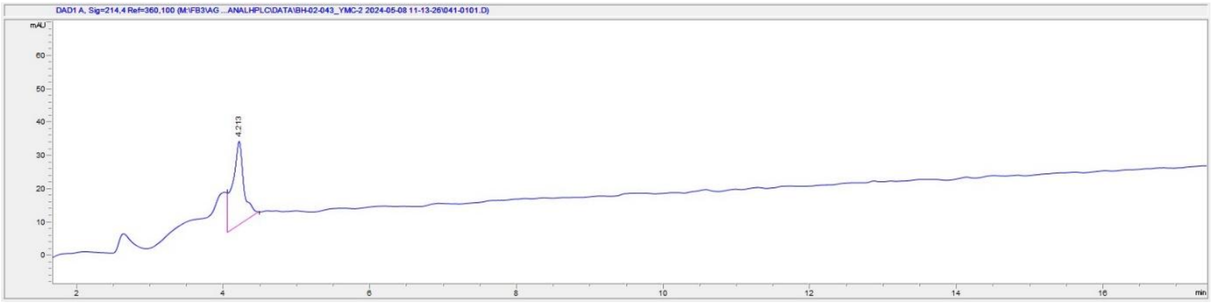

Figure S182: HPLC-UV trace (214 nm) of purified **p_4-Pep7**.

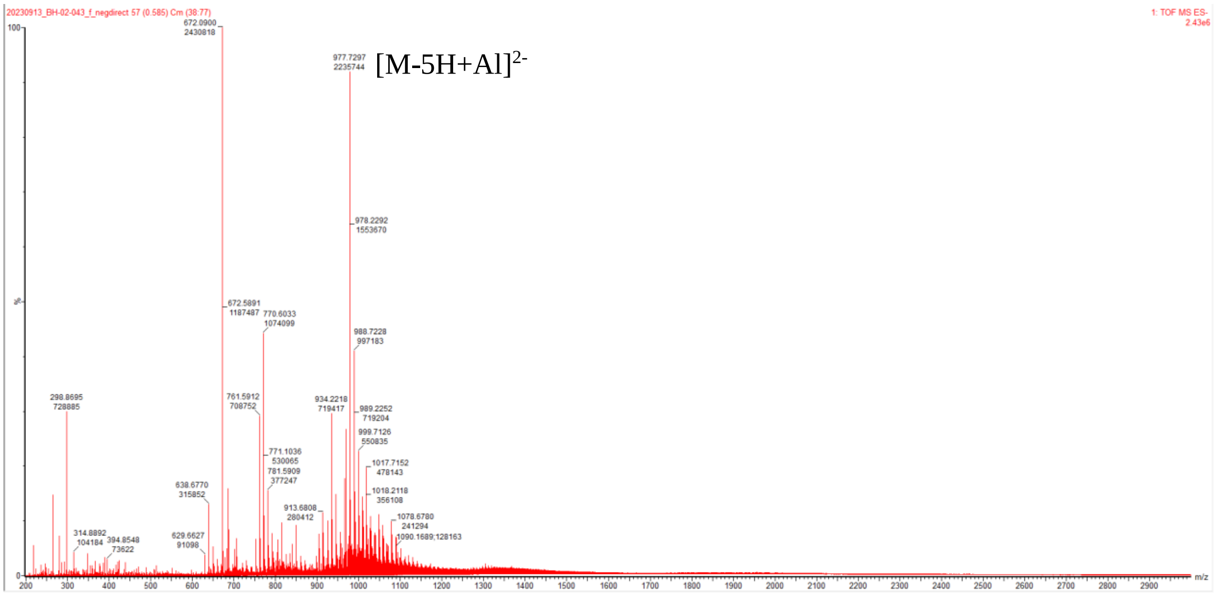

Figure S183: HRMS spectrum of **p_4-Pep7**.

NPE-p5-Pep7

H_2N-Lys-Lys-Ala-Ser-[NPE-pppppSer]-Ser-Asp-Ser-Glu-Asp-Ser-Ser-Glu-Glu-Glu-COOH

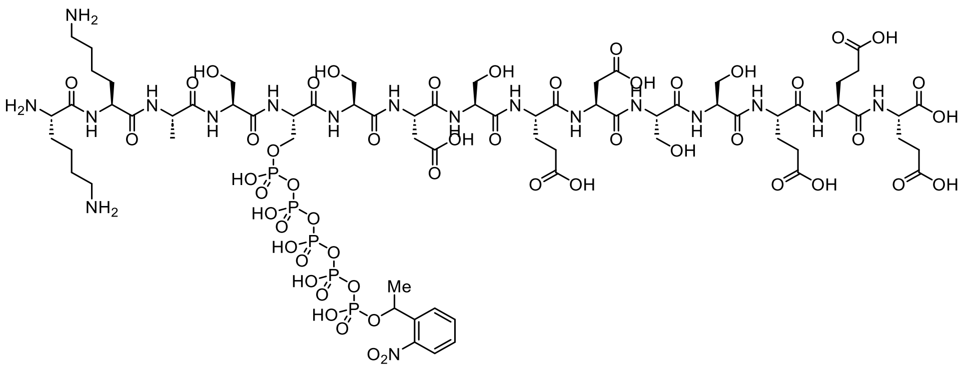

HRMS (ESI): m/z calc'd. for $C_{69}H_{106}AlN_{18}O_{51}P_5^{2-}$ $[M-5H+Al]^{2-}$ 1092.2384, found 1092.2384.
T_R (Method **A**) = 7.218 min (Al), 7.338 min (Fe).

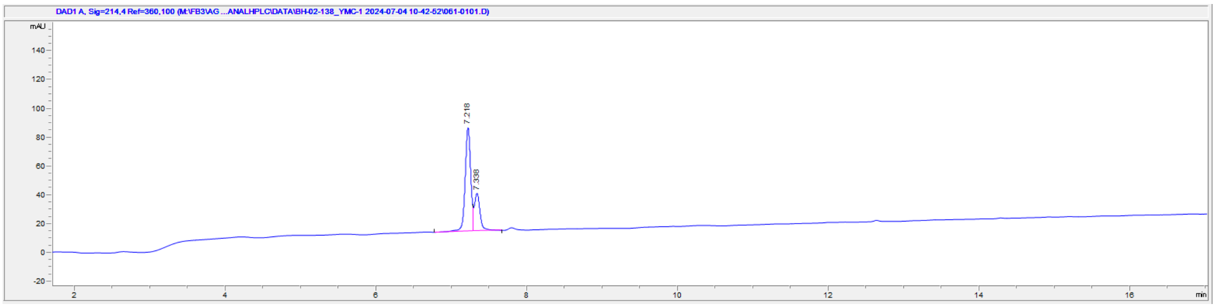

Figure S184: HPLC-UV trace (214 nm) of purified **NPE-p5-Pep7**.

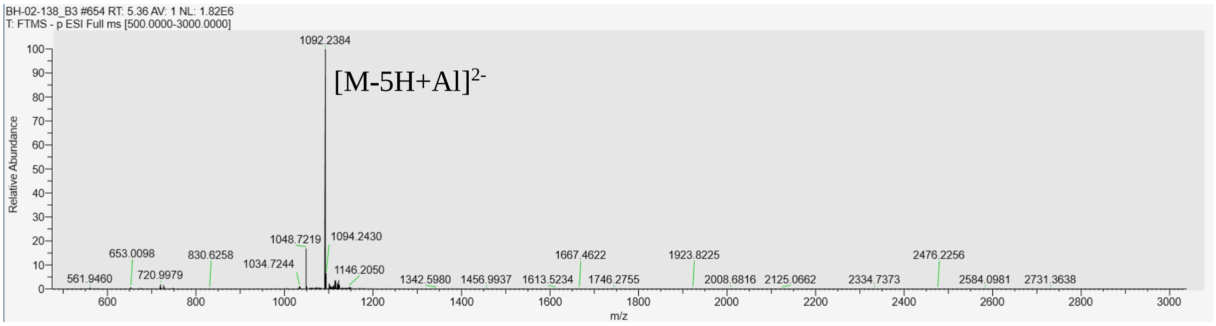

Figure S185: HRMS spectrum of **NPE-p5-Pep7**.

p_5-Pep7

H_2N-Lys-Lys-Ala-Ser-[pppppSer]-Ser-Asp-Ser-Glu-Asp-Ser-Ser-Glu-Glu-Glu-COOH

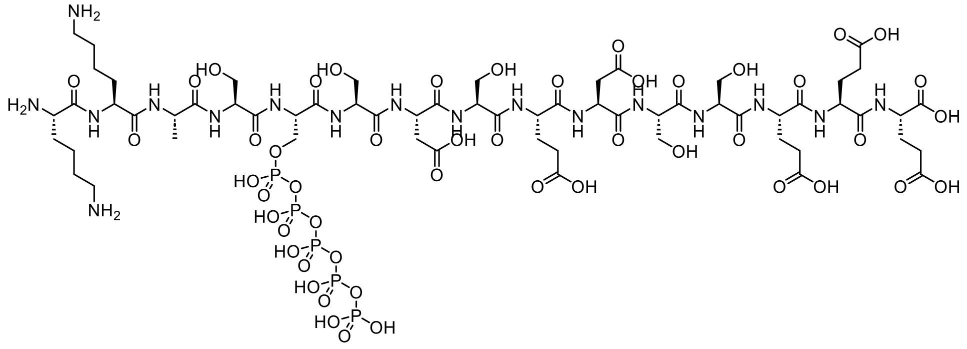

HRMS (ESI): m/z calc'd. for $C_{61}H_{99}AlN_{17}O_{49}P_5^{2-}$ $[M-5H+Al]^{2-}$ 1017.7146, found 1017.7141.

T_R (Method **A**) = 3.994 min (Al), 4.239 (Fe).

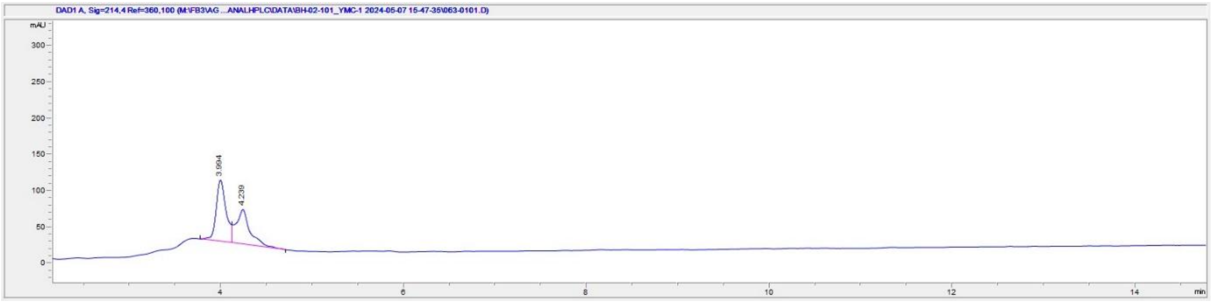

Figure S186: HPLC-UV trace (214 nm) of purified **p_5-Pep7**.

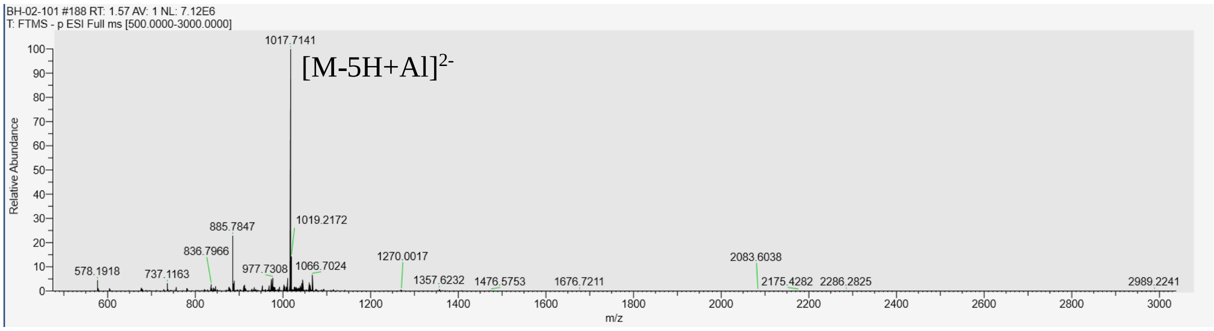

Figure S187: HRMS spectrum of **p_5-Pep7**.

NPE-p$_6$-Pep7

H_2N-Lys-Lys-Ala-Ser-[NPE-ppppppSer]-Ser-Asp-Ser-Glu-Asp-Ser-Ser-Glu-Glu-Glu-COOH

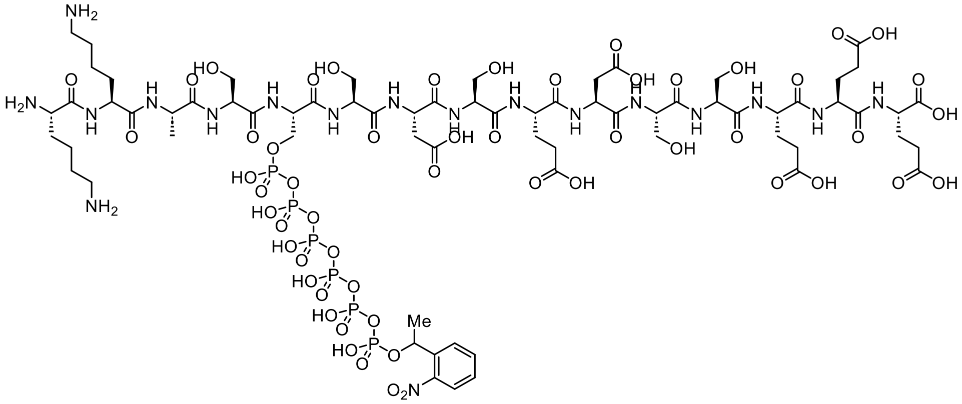

HRMS (ESI): m/z calc'd. for $C_{69}H_{107}AlN_{18}O_{54}P_6^{2-}$ $[M\text{-}5H\text{+}Al]^{2-}$ 1132.2216, found 1132.2208.
T_R (Method **A**) = 6.857 min.

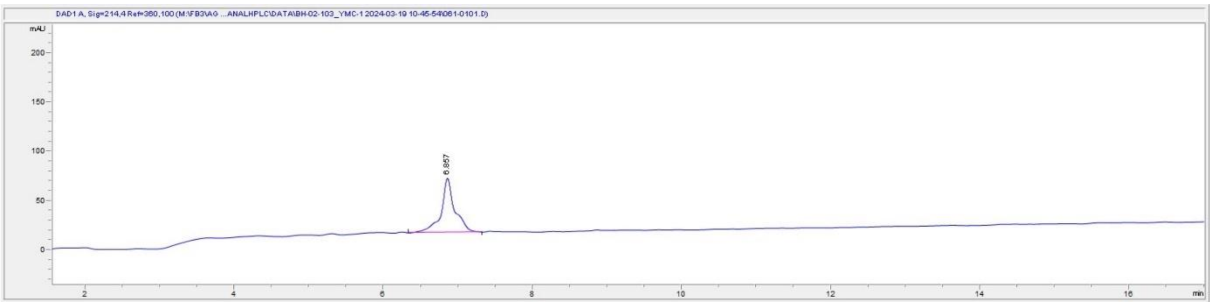

Figure S188: HPLC-UV trace (214 nm) of purified **NPE-p$_6$-Pep7**.

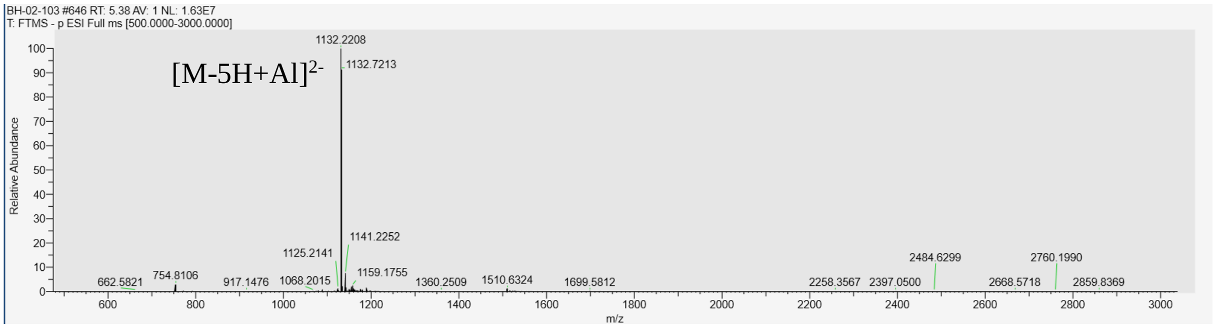

Figure S189: HRMS spectrum of **NPE-p$_6$-Pep7**.

p_6-Pep7

H_2N-Lys-Lys-Ala-Ser-[ppppppSer]-Ser-Asp-Ser-Glu-Asp-Ser-Ser-Glu-Glu-Glu-COOH

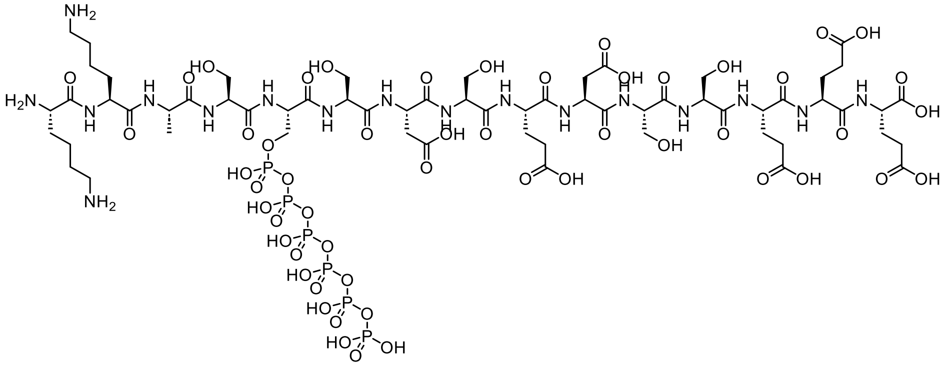

HRMS (ESI): m/z calc'd. for $C_{61}H_{100}AlN_{17}O_{52}P_6^{2-}$ $[M\text{-}5H\text{+}Al]^{2-}$ 1057.6977, found 1057.6938.

T_R (Method **A**) = 3.668 min.

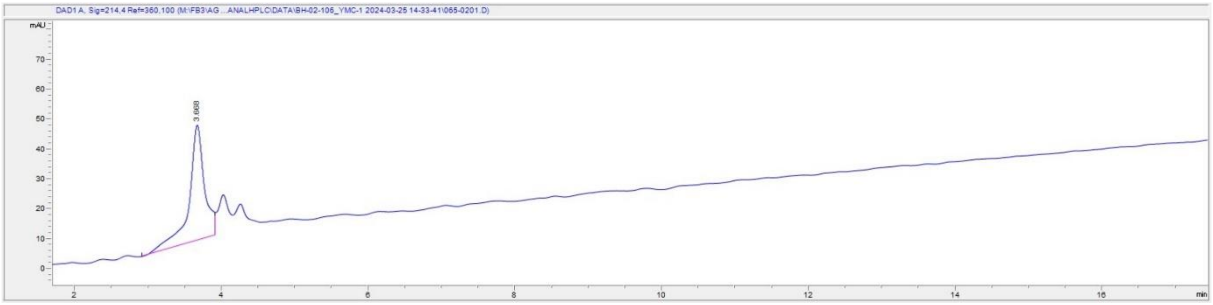

Figure S190: HPLC-UV trace (214 nm) of purified **p_6-Pep7**.

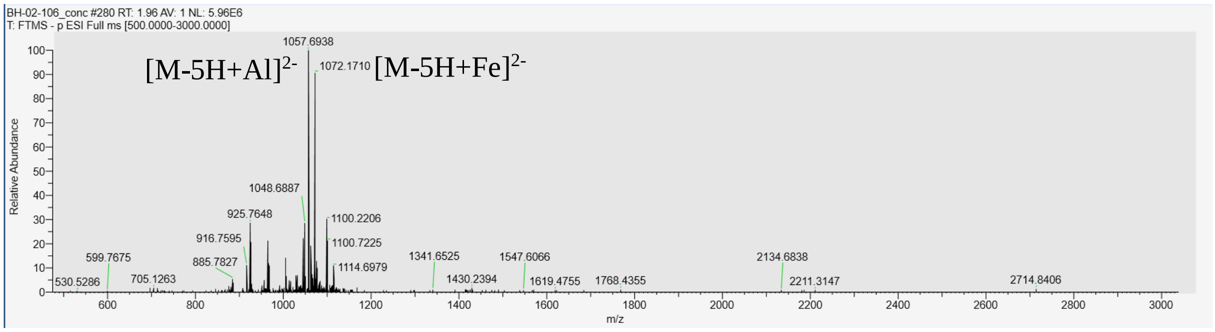

Figure S191: HRMS spectrum of **p_6-Pep7**.

7 Reactions of p-Pep1 with OligoP-Diimidazolides 3c and 4c

7.1 Reaction with 3c

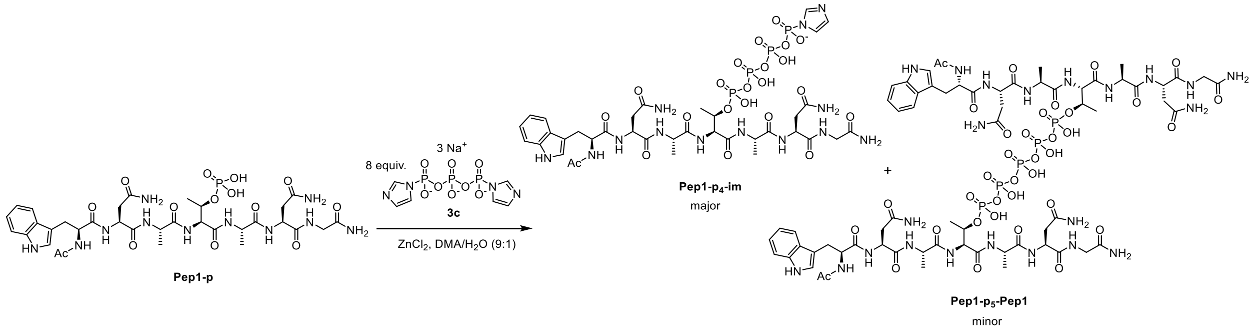

Synthetic procedure: Phosphopeptide **p-Pep1** (1.00 mg, 1.00 equiv.) was dissolved in DMA (500 µL) with the assistance of sonication. In parallel, oligoP-diimidazolide reagent **3c** (8.00 equiv.) and zinc chloride (80.0 equiv.) were suspended in DMA (400 µL) and Milli-Q water (100 µL) and dispersed with the assistance of sonication. Both solutions were combined and the resulting cloudy colorless reaction mixture was stirred at 45 °C for 40 min. Afterwards, the reaction mixture was diluted with Milli-Q water (4 mL), filtered through a syringe filter (Acrodisc, 0.2 µm), and submitted for high-resolution LC-MS (**Method X**) and preparative HPLC purification (**Method II**).

Method X: High resolution LC-MS data was collected on an Agilent 6545 Q-ToF mass spectrometer coupled to an Agilent Infinity 1260 LC system fitted with isocratic and binary solvent delivery systems (module no. G7110B and G7112B), an autosampler (module no. G7167A), and a column oven (module no. G7116A), using a Zorbax 300SB-C3 5 µm column (150 x 2.1 mm). Solvents: A = Milli-Q water + 0.1% FA, B = acetonitrile + 0.1% FA, with a gradient of: 1% B for 2 min, 1–61% B for 9 min, 61–95% B for 1 min, and a flow rate of 0.8 mL/min. Temperature: 40 °C;

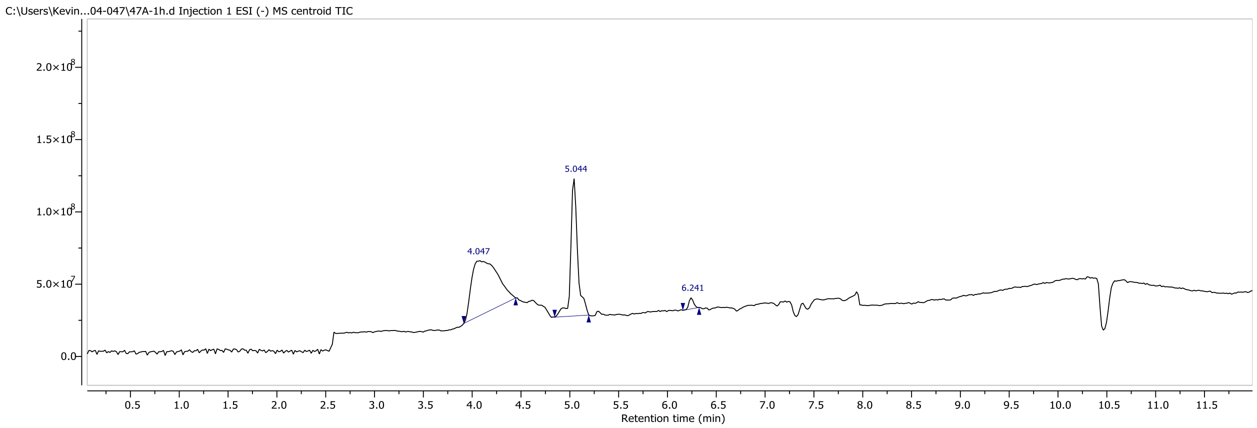

Figure S192: Total-ion-chromatogram (TIC) of aliquot from reaction mixture containing **p-Pep1**, **im-p$_4$-Pep1**, and **Pep1-p$_5$-Pep1** (**Method X**).

im-p₄-Pep1
AcHN-Trp-Asn-Ala-[im-ppppThr]-Ala-Asn-Gly-CONH$_2$

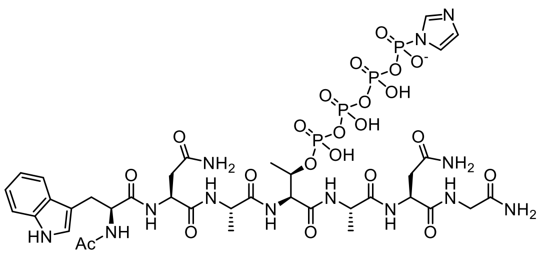

HRMS (ESI): m/z calc'd. for $C_{36}H_{52}N_{13}O_{22}P_4^-$ $[M-H]^-$ 1142.2306, found 1142.2202.
HRMS (ESI): m/z calc'd. for $C_{33}H_{48}N_{11}O_{22}P_4^-$ $[M-C_3H_4N_2]^-$ 1074.1931, found 1074.1830.
T_R (Method **X**) = 4.047 min.

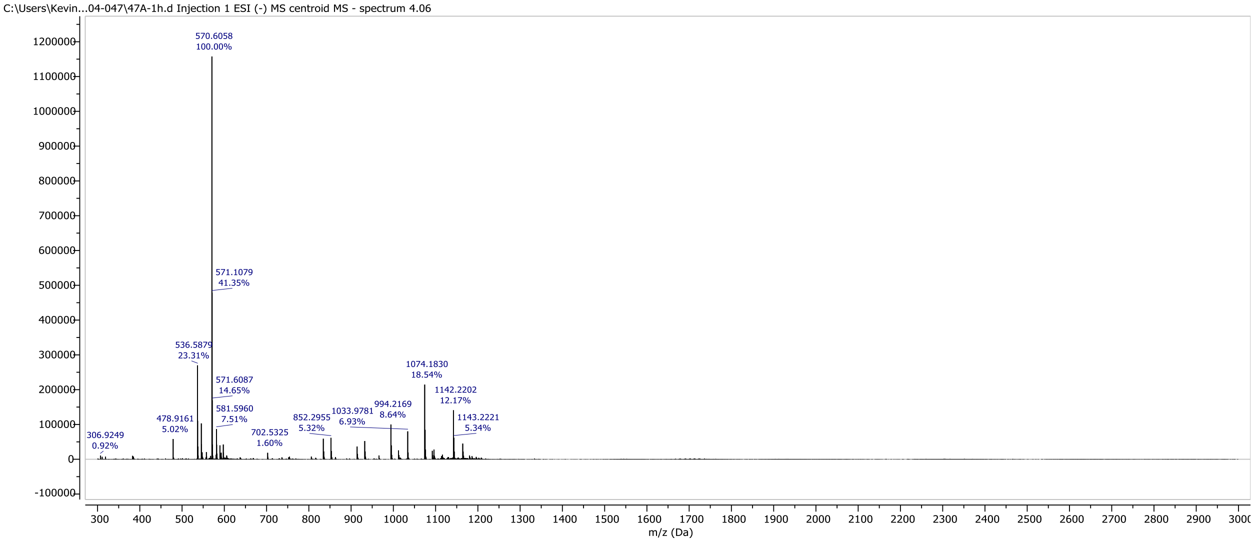

Figure S193: HRMS spectrum of **im-p₄-Pep1**.

Pep1-p$_5$-Pep1

AcHN-Trp-Asn-Ala-[Thr]-Ala-Asn-Gly-CONH$_2$

p$_5$

AcHN-Trp-Asn-Ala-[Thr]-Ala-Asn-Gly-CONH$_2$

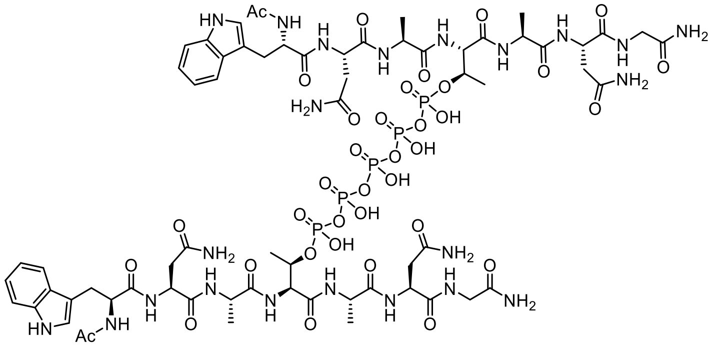

HRMS (ESI): m/z calc'd. for $C_{66}H_{92}AlN_{22}O_{36}P_5^{2-}$ $[M-5H+Al]^{2-}$ 975.2280, found 975.2253.

T_R (Method **X**) = 6.241 min.

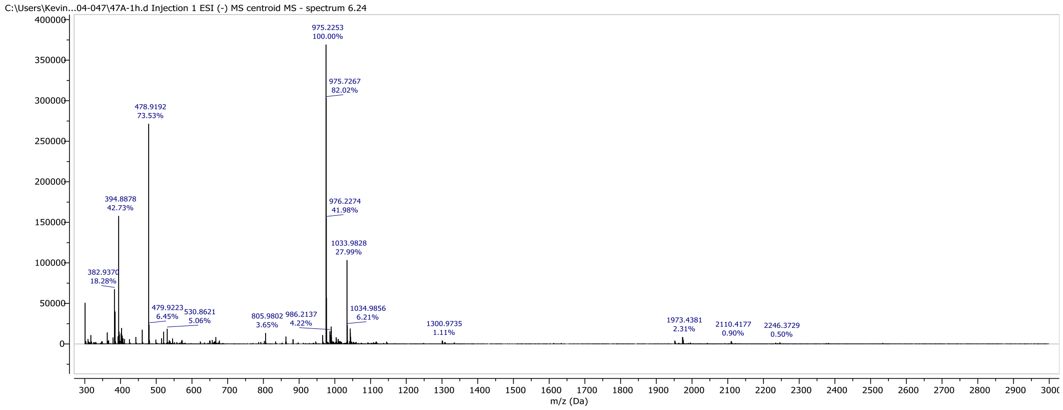

Figure S194: HRMS spectrum of **Pep1-p$_5$-Pep1**.

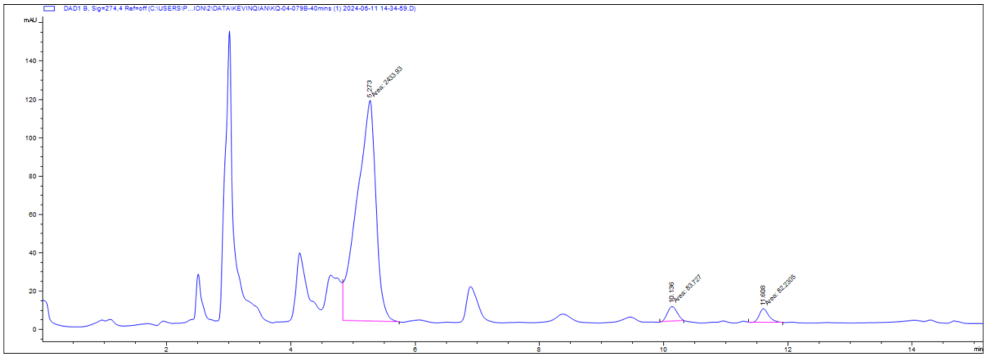

Figure S195: HPLC-UV trace (274 nm) of the reaction mixture resulting from **p-Pep1** and **3c**.

In the preparative RP-HPLC chromatogram, three peptidic species are observed: the reactant **p-Pep1** (T_R = 10.136 min), and products **Pep1-p$_5$-Pep1** (T_R = 11.608 min) and **p$_4$-Pep1** (T_R = 5.273 min). Under the preparative HPLC conditions detailed in **Method II**, the **im-p$_4$-Pep1** species completely hydrolyzes to **p$_4$-Pep1**. Therefore, conversion to **im-p$_4$-Pep1** was determined by quantifying the hydrolysis product **p$_4$-Pep1** as a proxy.

7.2 Reaction with 4c

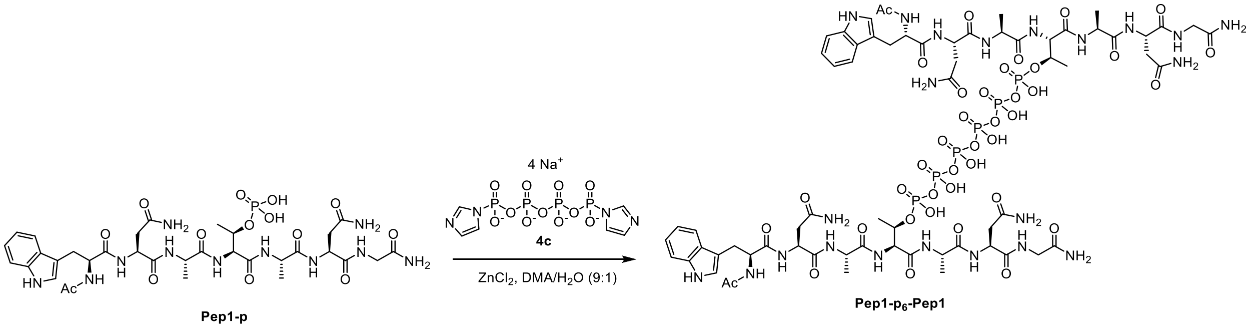

Synthetic procedure: Phosphopeptide **p-Pep1** (4.00 mg, 1.00 equiv.) was dissolved in DMA (2 mL) with the assistance of sonication. In parallel, oligoP-diimidazolide reagent **4c** (8.00 equiv.) and zinc chloride (80.0 equiv.) were suspended in DMA (1.6 mL) and Milli-Q water (400 µL) and dispersed with the assistance of sonication. Both solutions were combined and the resulting cloudy colorless reaction mixture was stirred at 45 °C for 1 h. Afterwards, the reaction mixture was diluted with Milli-Q water (6 mL), filtered through a syringe filter (Acrodisc, 0.2 µm), and submitted to preparative HPLC (Method **II**). After lyophilization of the product containing fractions, the product **Pep1-p$_6$-Pep1** was obtained as a colorless solid (1.1 mg, 23% yield). The purified product was characterized by HPLC-UV (Method **B**) and high resolution ESI-MS (Method **X**).

Pep1-p$_6$-Pep1

AcHN-Trp-Asn-Ala-[Thr]-Ala-Asn-Gly-CONH$_2$

p$_6$

AcHN-Trp-Asn-Ala-[Thr]-Ala-Asn-Gly-CONH$_2$

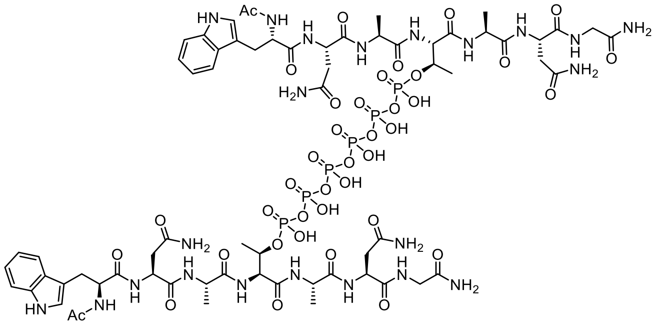

HRMS (ESI): m/z calc'd. for $C_{66}H_{93}AlN_{22}O_{39}P_6^{2-}$ [M-5H+Al]$^{2-}$ 1015.2111, found 1015.2180.
T$_R$ (Method **B**) = 8.458 min; T$_R$ (Method **X**) = 5.894 min.

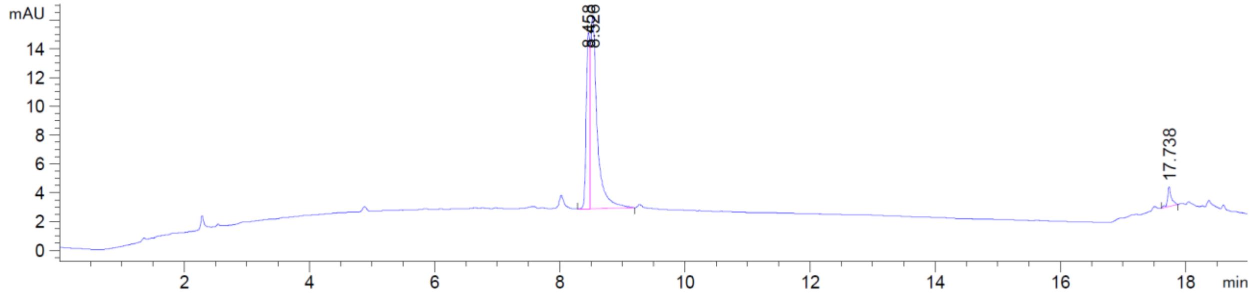

Figure S196: HPLC-UV trace (274 nm) of purified **Pep1-p$_6$-Pep1**.

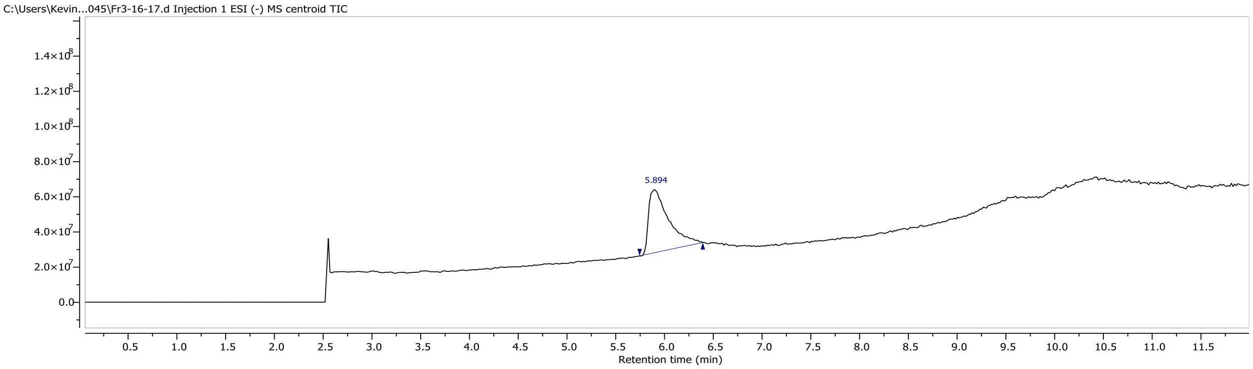

Figure S197: Total-ion-chromatogram (TIC) of purified **Pep1-p$_6$-Pep1**.

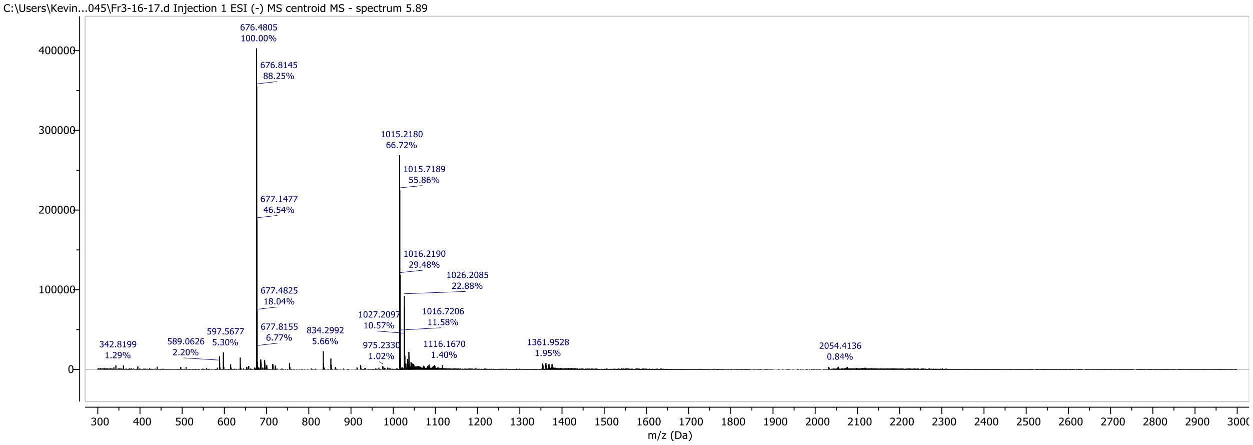

Figure S198: HRMS spectrum of **Pep1-p$_6$-Pep1**.

8 NMR spectra of Pentaphosphopeptide Bn-p5-Pep1

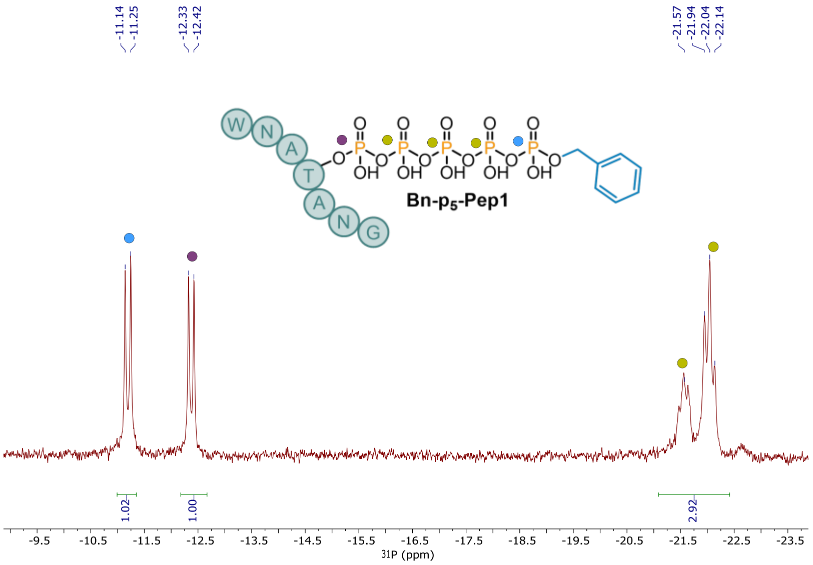

Figure S199: $^{31}P\{^1H\}$ NMR spectrum of **Bn-p5-Pep1**.

$^{31}P\{^1H\}$ NMR (162 MHz, D_2O) δ -11.19 (d, J = 16.7 Hz), -12.37 (d, J = 15.8 Hz), -21.57 (t, J = 14.4 Hz), -22.04 (t, J = 15.8 Hz).

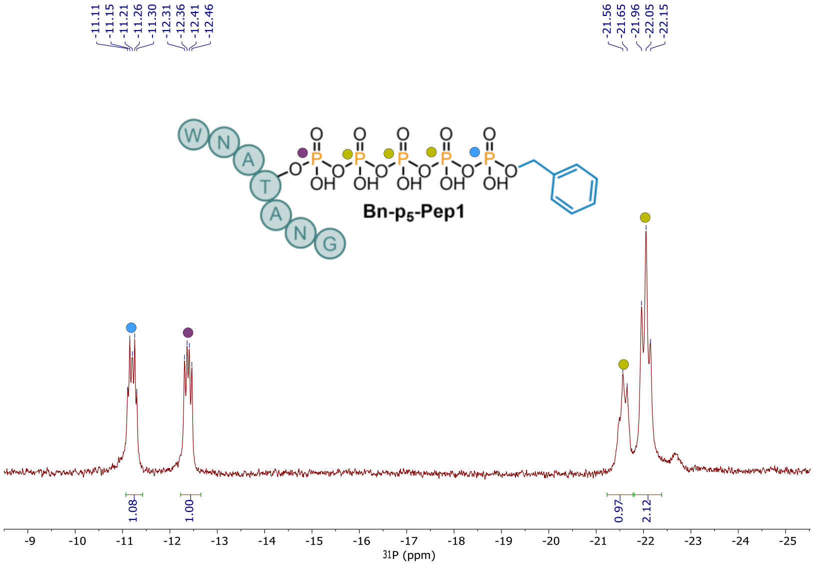

Figure S200: ^{31}P NMR spectrum of **Bn-p5-Pep1**.

^{31}P NMR (162 MHz, D_2O) δ -11.20 (dt, J = 15.2, 7.5 Hz), -12.39 (dd, J = 16.4, 8.8 Hz), -21.61 (t, J = 14.8 Hz), -22.05 (t, J = 15.5 Hz).

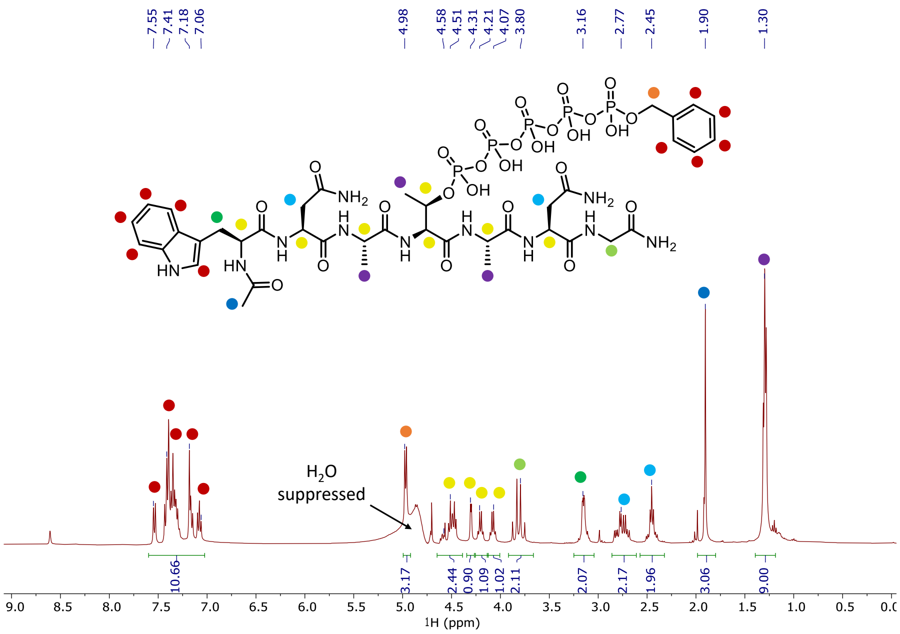

Figure S201: Solvent suppressed ^{1}H NMR spectrum of **Bn-p$_5$-Pep1**.

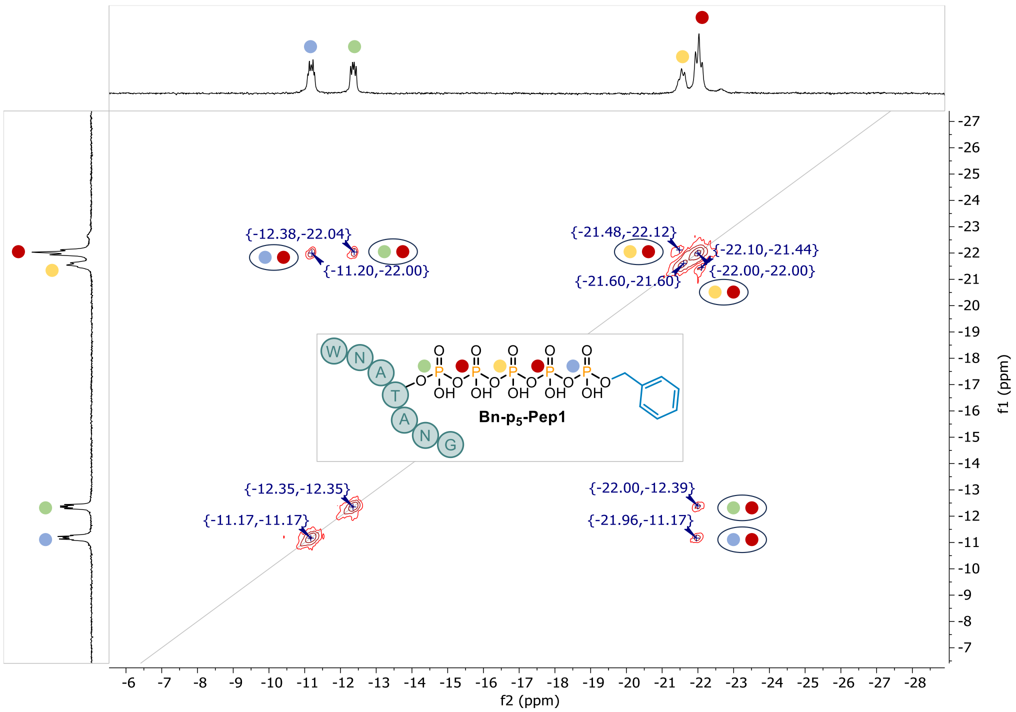

Figure S202: ^{31}P-^{31}P COSY NMR spectrum of **Bn-p$_5$-Pep1**.

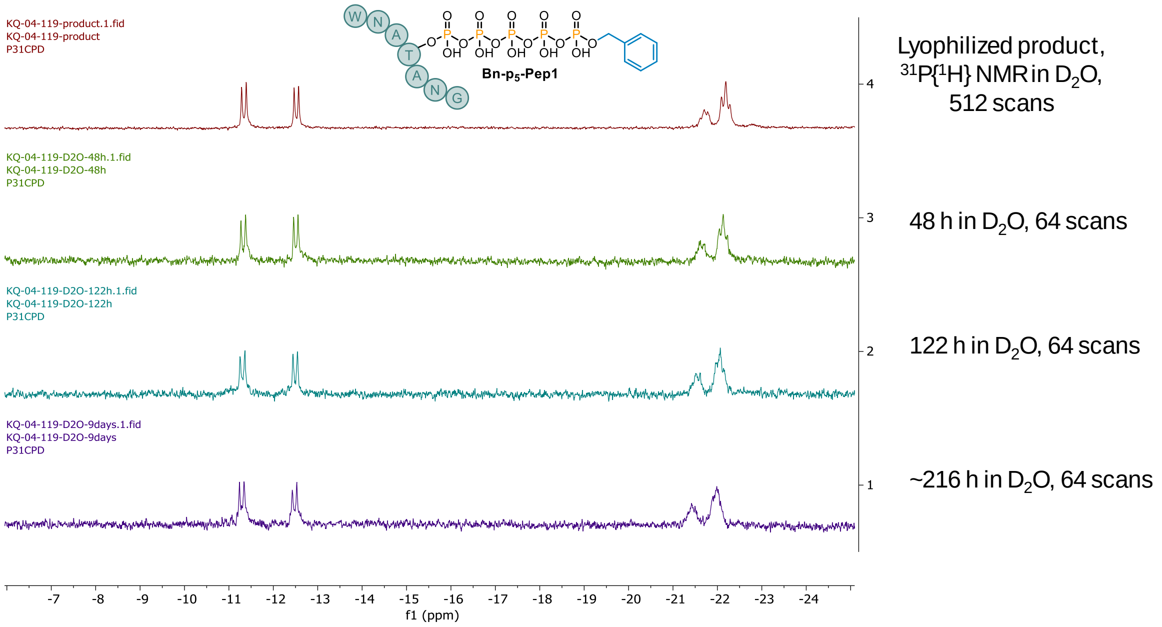

Figure S203: Stacked $^{31}P\{^{1}H\}$ NMR spectra of Bn-p_5-Pep1 in D_2O at various time points, indicating no discernible hydrolysis occurs after 9 days.

9 OligoP-imidazolide Hydrolysis Kinetics Study

Four solutions of P-imidazolide reagents in D_2O were prepared at the following concentrations:

(1) 33 mM **1b**
(2) 25 mM **4a**
(3) 25 mM **4a** + 120 mM $ZnCl_2$
(4) 11 mM **4b**

We monitored the hydrolysis of **1b**, **4a**, and **4b** at room temperature by ^{31}P NMR spectroscopy by integrating the P-imidazolide peaks with the hydrolysis product peaks. For each spectrum, 32 scans were collected with a delay time of 10 s. The concentrations of **1b**, **4a**, and **4b** were plotted and fitted according pseudo first-order reaction kinetics. To clarify, the hydrolysis reactions were conducted in D_2O but it was not our intention to measure the kinetic isotope effect, k_H/k_D. We aimed to compare the relative rates of hydrolysis between the different P-imidazolides, and D_2O was used for convenient locking and shimming.

P-imidazolide **1b** hydrolyzed in D_2O with an observed first order rate constant of 1.45 x 10^{-6} s^{-1} and a half-life of 132.8 h. Compared to monoP-imidazolides, the oligoP-imidazolides appeared to be significantly more reactive; the rate of hydrolysis of **4a** is more than an order-of-magnitude higher than that for **1b**, with a rate constant of 1.97 x 10^{-5} s^{-1}. As expected, Zn^{2+} activates the oligoP-imidazolide, accelerating the hydrolysis reaction. Replacing the benzyl group with NPE has a slight effect on the rate of hydrolysis, as **4b** hydrolyzes with a rate constant of 3.25 x 10^{-5} s^{-1}.

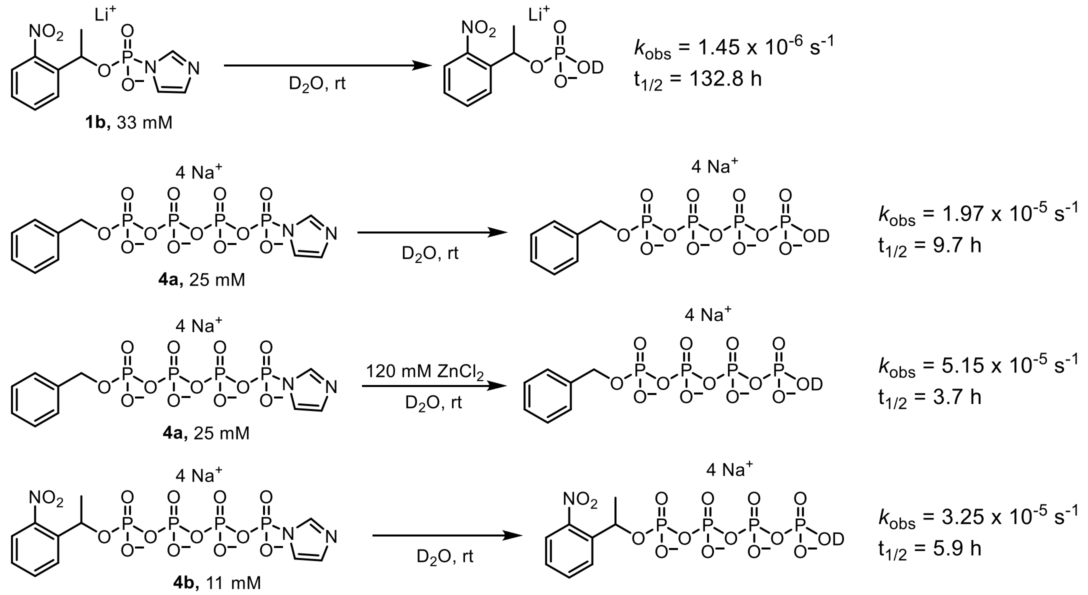

Figure S204: Summary of P-imidazolide and oligoP-imidazolide hydrolysis kinetics.

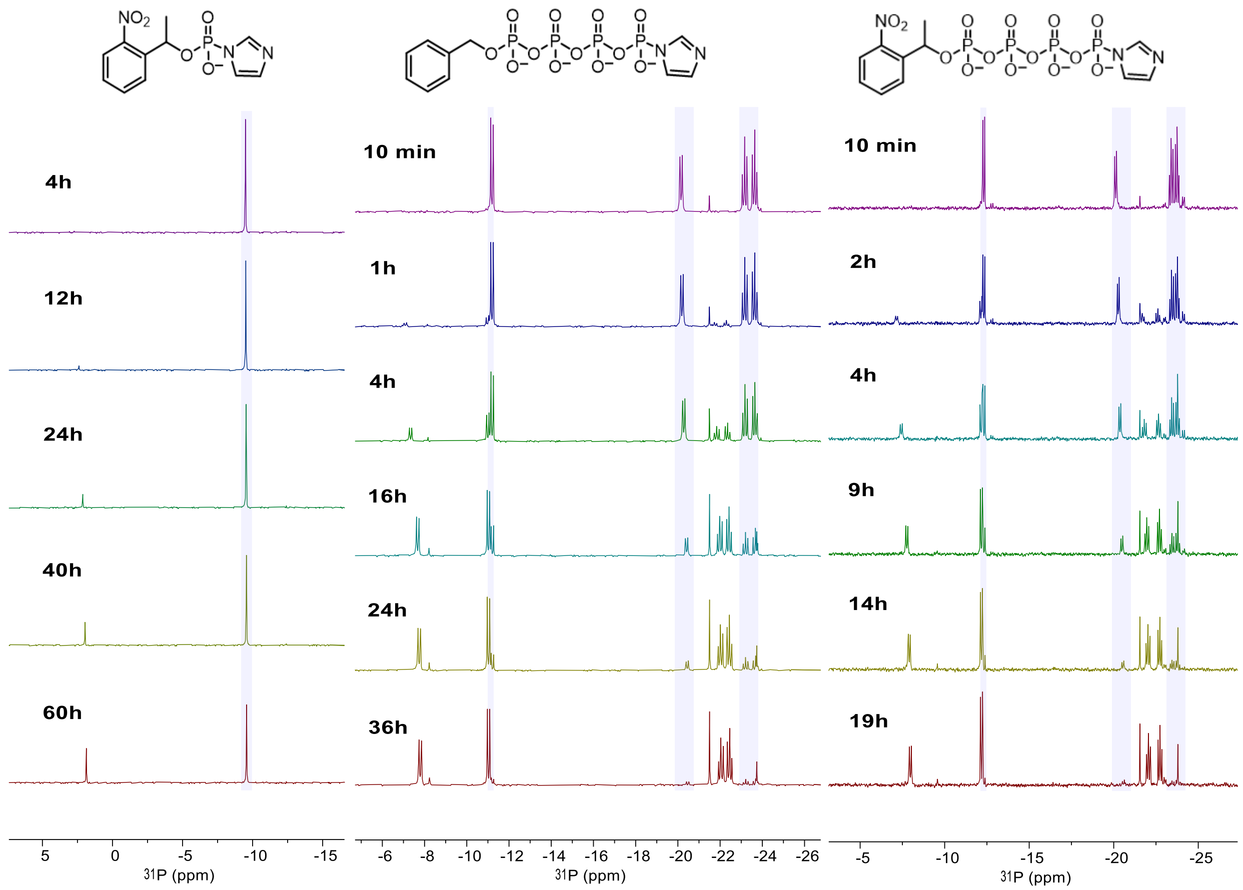

Figure S205: Monitoring of P-imidazolide and oligoP-imidazolide hydrolysis by ^{31}P NMR spectroscopy.

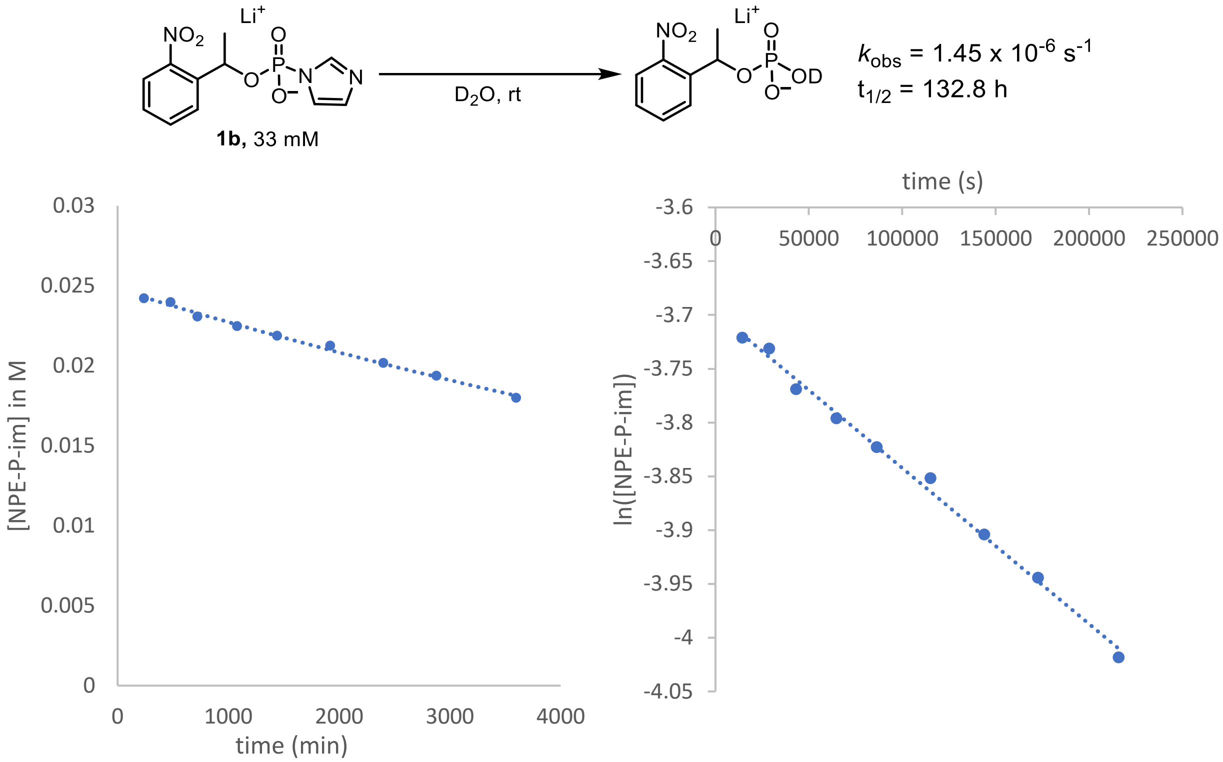

Figure S206: First-order decay fit for the hydrolysis of **1b**.

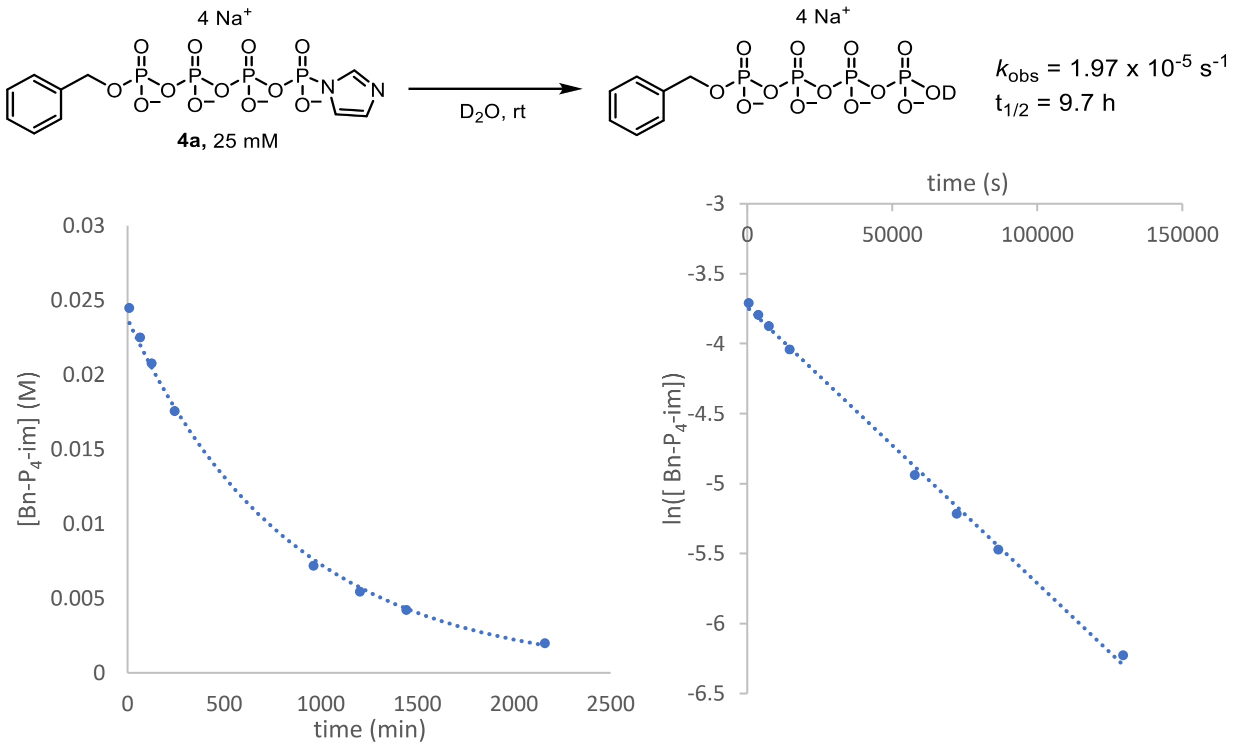

Figure S207: First-order decay fit for the hydrolysis of **4a**.

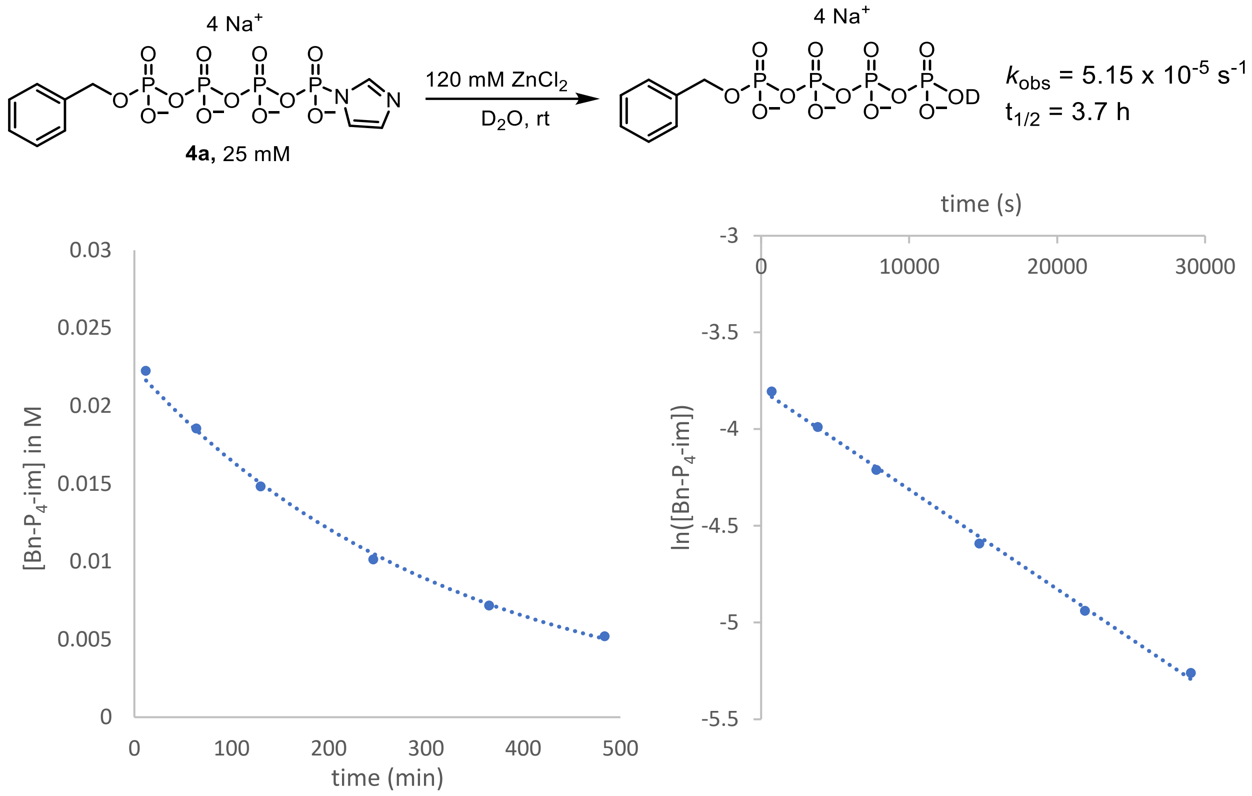

Figure S208: First-order decay fit for the hydrolysis of **4a** with ~5 equiv. of $ZnCl_2$.

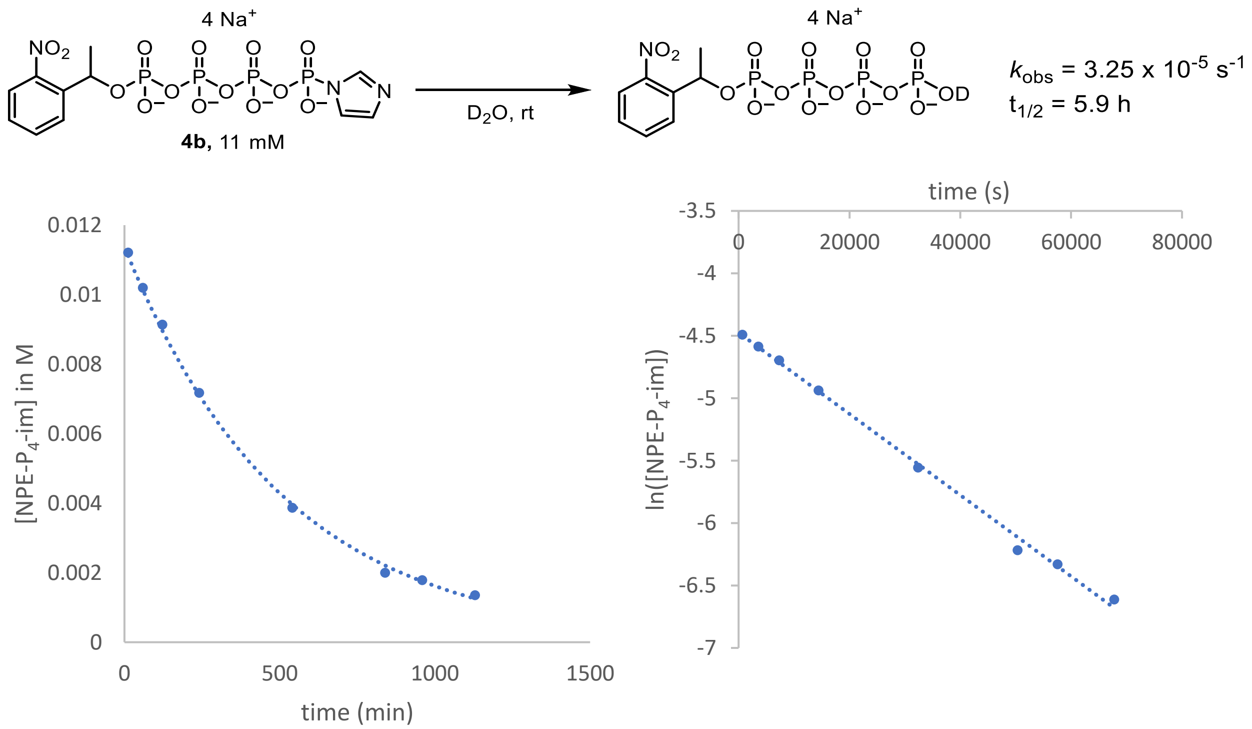

Figure S209: First-order decay fit for the hydrolysis of **4b**.

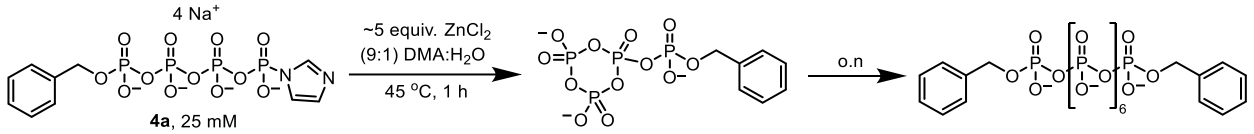

In an NMR tube, a solution of **4a** (25 mM) and $ZnCl_2$ (120 mM) in a 9:1 mixture of DMA/water was heated to 45 °C in a water bath. Within 1 h, ^{31}P NMR spectroscopy reveals the unambiguous formation of a benzyl phosphoryl-trimetaphosphate as the major species, presumably formed from the intramolecular cyclization of the oligoP-imidazolide.

After allowing the reaction mixture to incubate at this temperature overnight, a species which we assign as dibenzyl octaphosphate was observed as the major product.

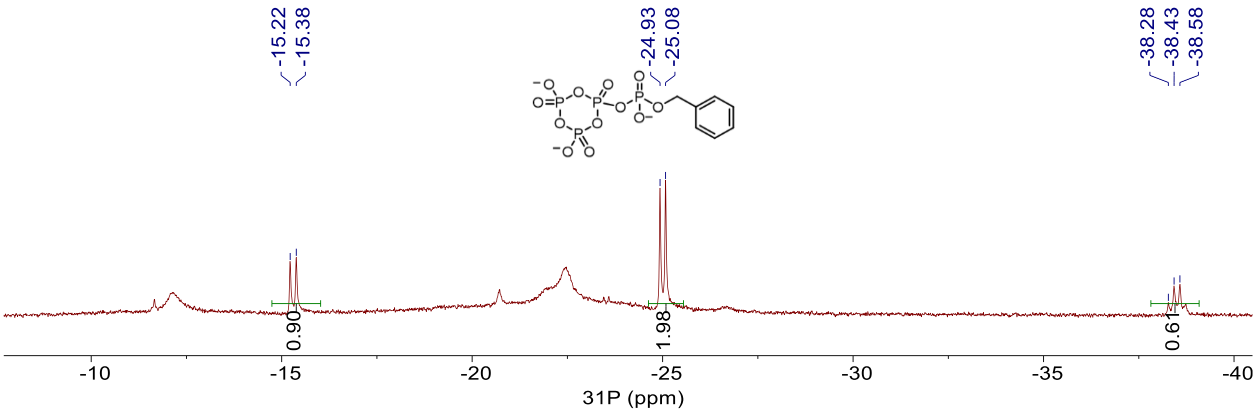

Figure S210: $^{31}P\{^1H\}$ NMR spectrum of cyclization product of **4a**.

$^{31}P\{^1H\}$ NMR (162 MHz, DMA/H_2O) δ -15.30 (d, J = 26.0 Hz, 1P), -25.00 (d, J = 23.4 Hz, 2P), -38.53 (dd, J = 26.0, 23.4 Hz, 1P).

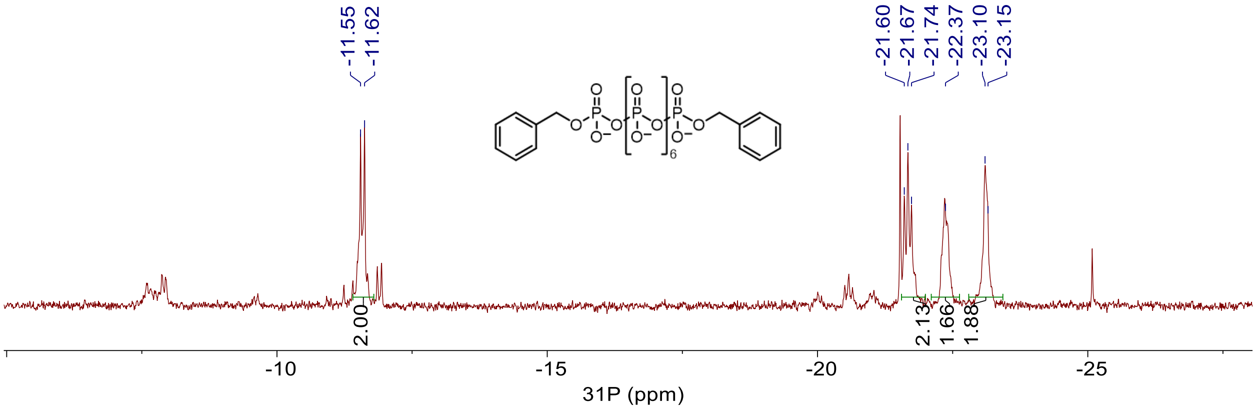

Figure S211: $^{31}P\{^1H\}$ NMR spectrum of dimerization product of **4a**.

$^{31}P\{^1H\}$ NMR (202 MHz, DMA/H_2O) δ -11.58 (d, J = 15.4 Hz, 2P), -21.67 (t, J = 13.6 Hz, 2P), -22.37 (br, 2P), -23.13 (br, 2P).

10 Tandem MS/MS Spectrometry

Tandem MS/MS experiments on purified samples of **p-Pep1** and **Bn-p$_5$-Pep1** were performed on an Agilent Ultivo triple-quadrupole QQQ mass spectrometer with an ESI source coupled to an Agilent Infinity 1260 LC system operating on Agilent Chemstation and MassHunter software. A Zorbax 300SB-C3 5 µm column (2.1 x 150 mm) was used for these two phosphopeptides. Mobile phase solvents consisted of: A = Milli-Q water + 0.1% FA, B = acetonitrile + 0.1% FA, with a gradient of: 1% B for 2 min, 1–61% B for 9 min, 61–95% B for 1 min, a flow rate of 0.8 mL/min, and a column temperature of 40 °C. Parent ions were fragmented by collision-induced dissociation (CID).

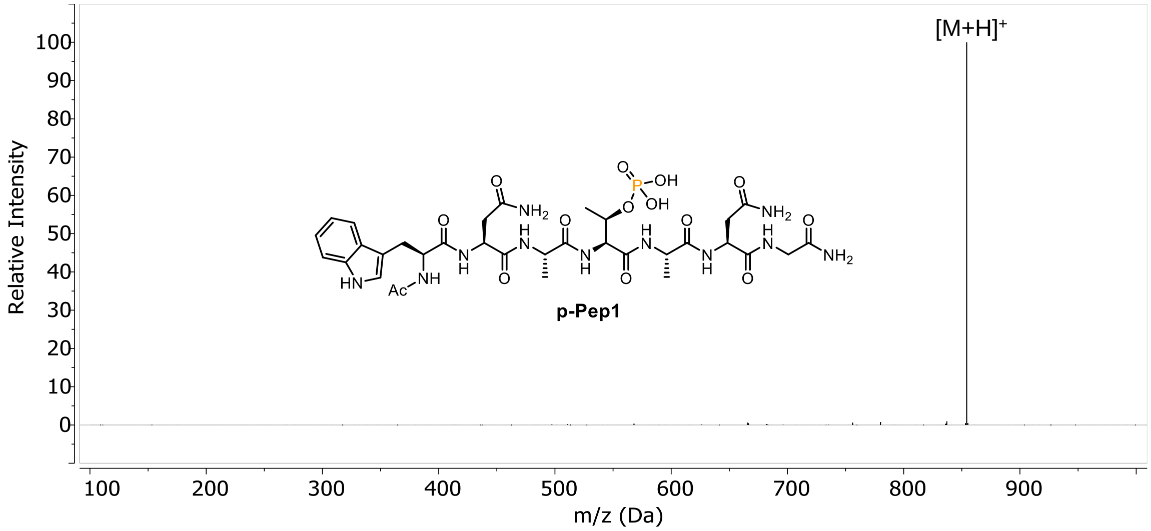

Figure S212: CID MS/MS spectrum of **p-Pep1**. With a collision energy of 0 V in positive ion mode, the parent ion $[M+H]^+$ of the phosphopeptide substrate **p-Pep1** was the only prominent species observed.

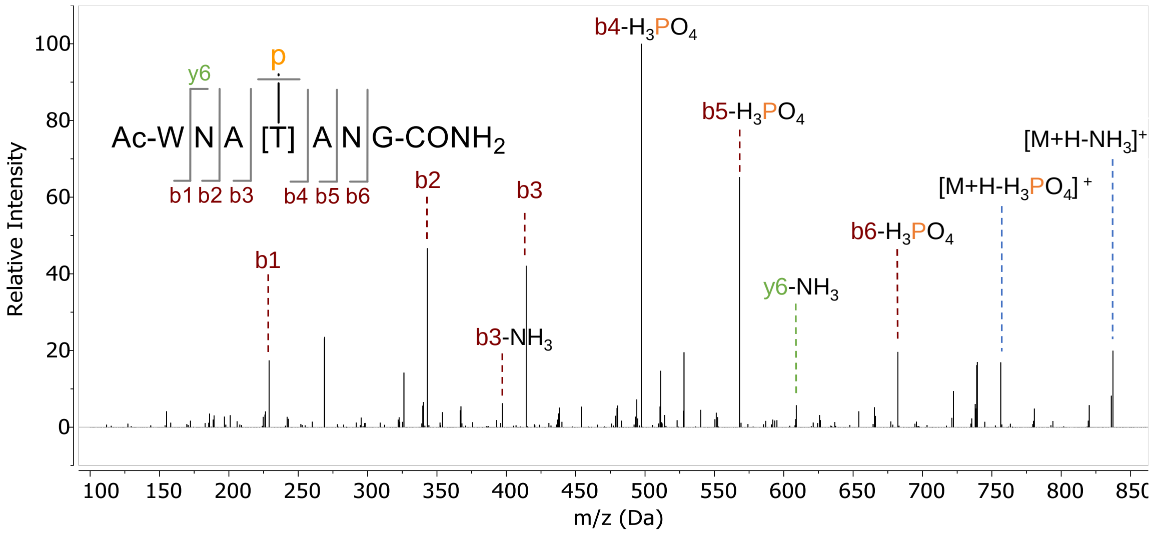

Figure S213: CID MS/MS spectrum of **p-Pep1**. The parent ion $[M+H]^+$ of **p-Pep1** at 854 m/z was fragmented with a collision energy of 30 V in positive ion mode. The combination of observed b and y ions confirmed the sequence of the **p-Pep1** phosphopeptide substrate.

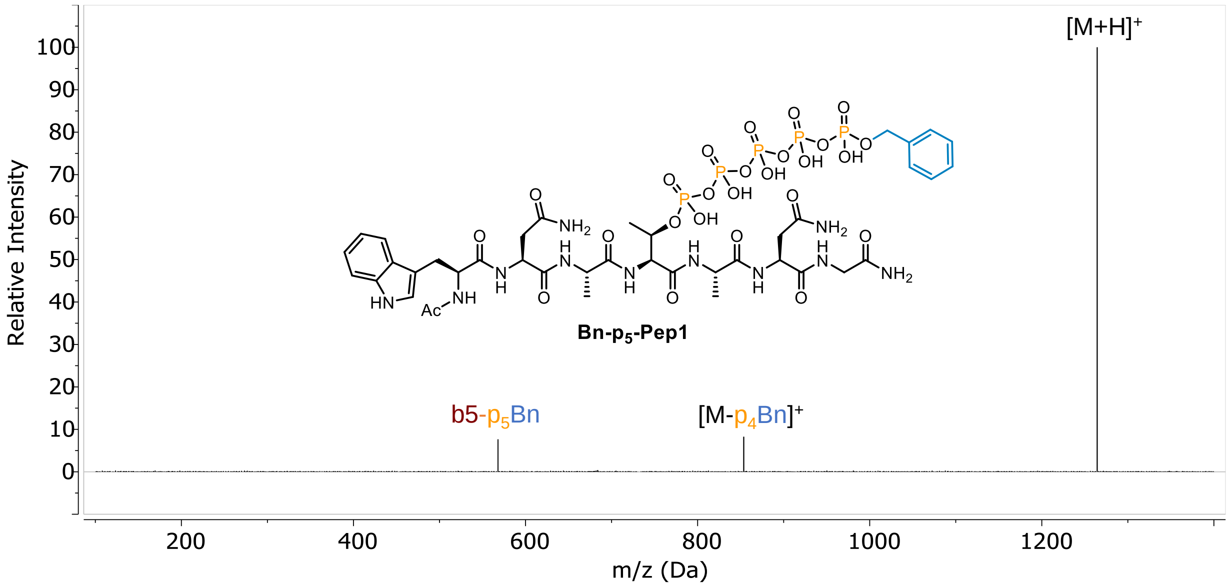

Figure S214: CID MS/MS spectrum of **Bn-p$_5$-Pep1**. With a collision energy of 0 V in positive ion mode, the parent $[M+H]^+$ of **Bn-p$_5$-Pep1** was observed as the predominant species, along with some minor fragment ions.

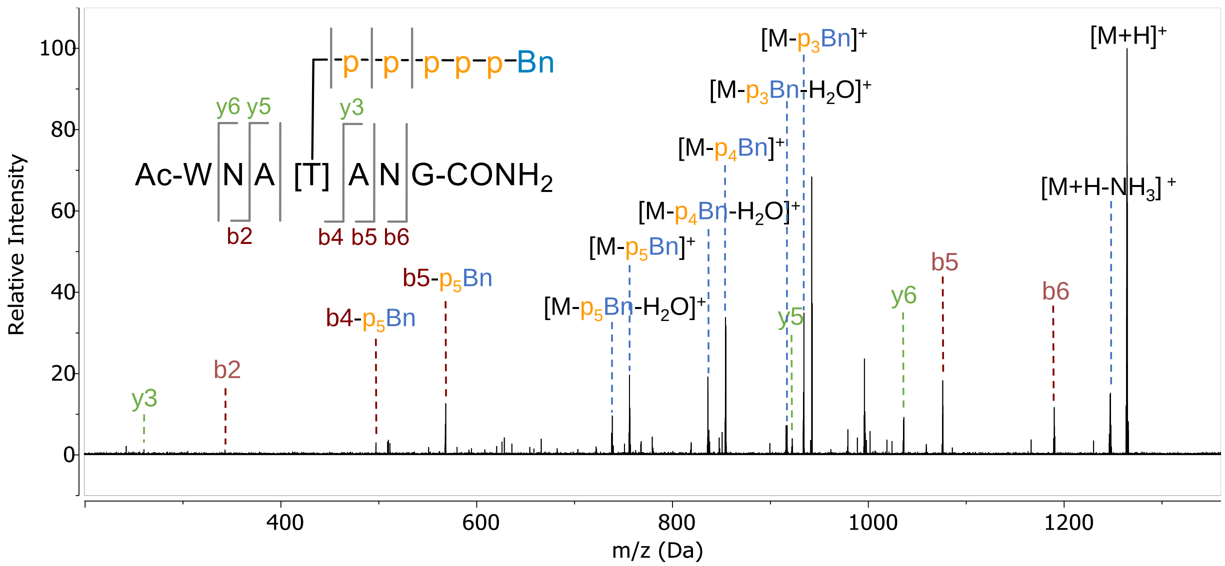

Figure S215: CID MS/MS spectrum of **Bn-p$_5$-Pep1**. The parent ion of **Bn-p$_5$-Pep1** at 1264 m/z was fragmented with a collision energy of 15 V in positive ion mode. A number of daughter ions corresponding to fragmentation along the peptide and/or oligophosphate chain were observed. The combination of observed b and y ions confirmed the sequence of the **Bn-p$_5$-Pep1** oligophosphopeptide product.

11 Protein Oligophosphorylation

Intact proteins were analyzed on an Agilent 6545 Q-ToF high-resolution mass spectrometer coupled to an Agilent Infinity 1260 LC system running on Agilent Chemstation, MassHunter, and BioConfirm software. A Zorbax 300SB-C3 5 µm column (2.1 x 150 mm) was used for intact protein analysis. Mobile phase solvents consisted of: A = Milli-Q water + 0.1% FA, B = acetonitrile + 0.1% FA, with a gradient of: 1% B for 2 min, 1–61% B for 9 min, 61–95% B for 1 min, and a flow rate of 0.8 mL/min. ESI-QToF-MS data was collected in positive mode with extended dynamic range of 300–3000 m/z. Typical MS conditions are as follows: gas temperature = 350 °C, drying gas flow rate = 11 L/min, nebulizer pressure = 60 psi, the capillary and fragmentor voltages were set at 4000 V and 175 V, respectively

Deconvoluted masses were obtained in BioConfirm B.09.00. using a maximum entropy algorithm with a mass range of 8000–12000 Da, a mass step of 1.0000 Da, a limited m/z range of 700–3000 m/z, and a baseline subtraction factor of 3.00. All deconvoluted masses are reported to the nearest integer value.

Recombinant human ubiquitin **wt-Ub** was purchased from Bio-Techne® (Catalog #: U-100H-10M) as a lyophilized powder. Phospho-ubiquitin **pS65-Ub** was also purchased from Bio-Techne® (Catalog #: U-102-150) as a 2.170 µg/µL solution in 10 mM HEPES buffer (pH 7.5) and was used as provided.

11.1 wt-Ub

Sequence:
MQIFVKTLTG KTITLEVEPS DTIENVKAKI QDKEGIPPDQ QRLIFAGKQL EDGRTLSDYN IQKESTLHLV LRLRGG

Calculated average molecular weight for **wt-Ub**: 8564.8 Da
Deconvoluted mass found: 8565 Da

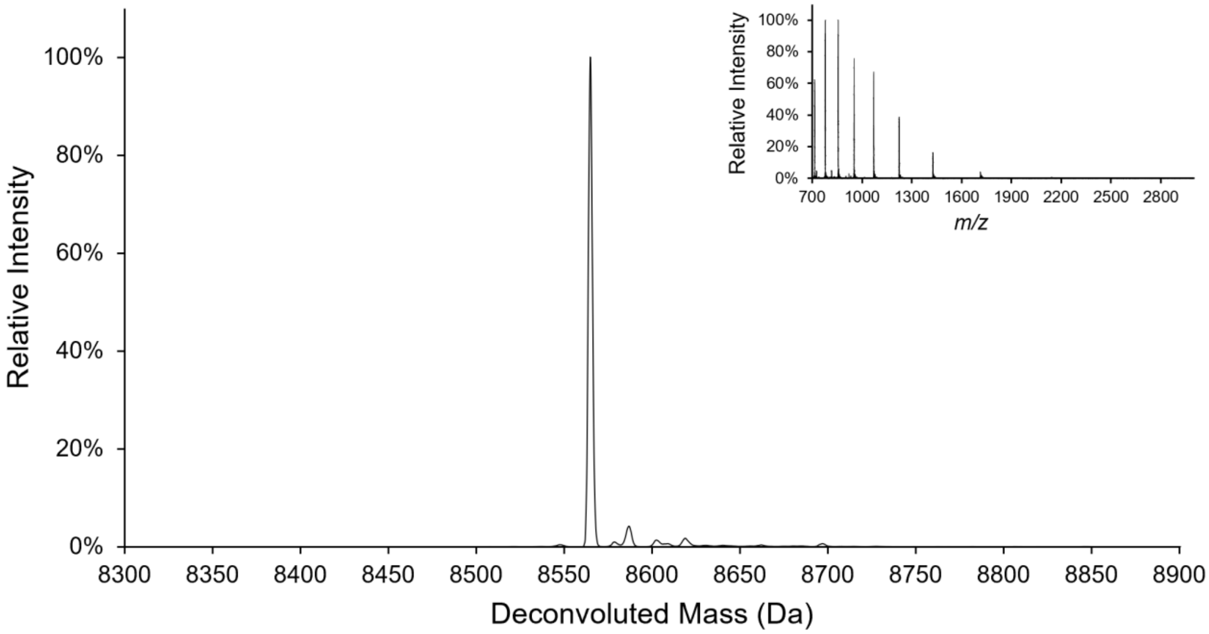

Figure S216: Deconvoluted mass spectrum of **wt-Ub**.

11.2 pS65-Ub

Sequence:
MQIFVKTLTG KTITLEVEPS DTIENVKAKI QDKEGIPPDQ QRLIFAGKQL EDGRTLSDYN IQKE(**pS**)TLHLV LRLRGG

Calculated average molecular weight for **pS65-Ub**: 8644.8 Da
Deconvoluted mass found: 8645 Da

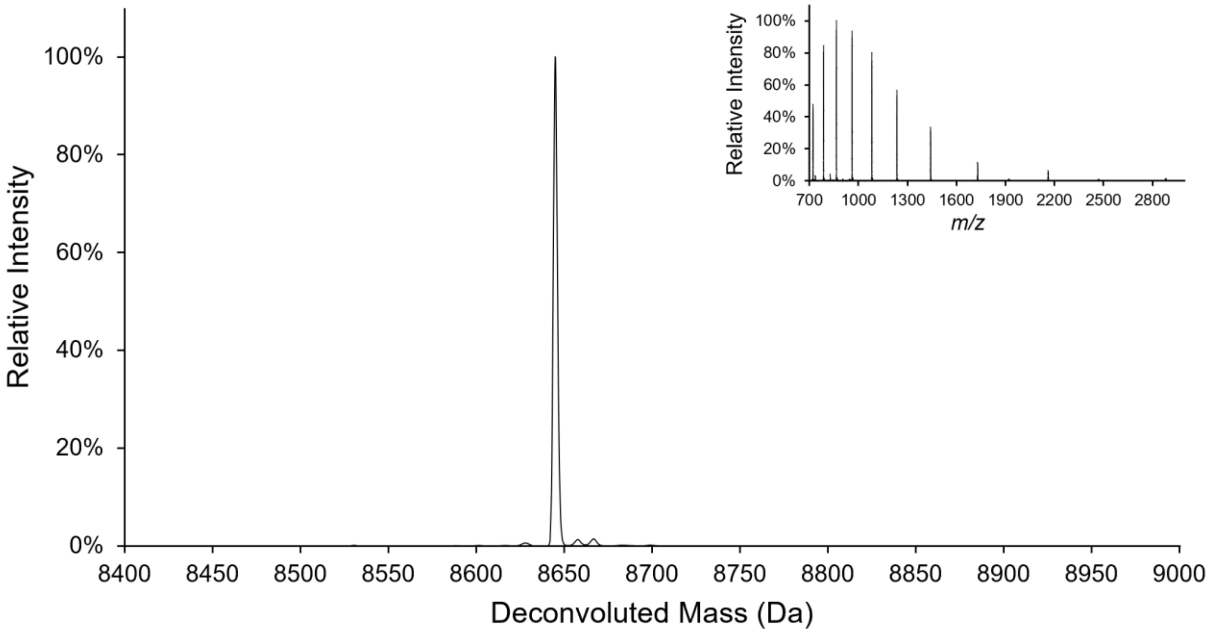

Figure S217: Deconvoluted mass spectrum of **pS65-Ub**.

11.3 NPE-p$_4$S65-Ub

Reactions with **pS65-Ub** were conducted following a modified literature procedure.[4] A 1.5 mL Eppendorf® tube was charged with solid reagent **3b** (7.2 mg, 13.8 µmol), and to it was added 200 µL of a 340 mM $ZnCl_2$ solution in DMA. This suspension was sonicated briefly. A 22 µL aliquot of a 2.170 µg/µL stock solution of **pS65-Ub** (48 µg, 5.5 nmol) in 10 mM aqueous HEPES buffer was added to the Eppendorf® tube, resulting in a 9:1 DMA/aqueous buffer mixture. The contents in the tube were thoroughly mixed *via* vortexing, and the solution was incubated at 45 ˚C for 2 h, interrupted by occasional mixing by inversion. As the reaction progressed at this elevated temperature, the cloudy suspension transformed into a colorless homogeneous mixture. After the allotted time, the reaction mixture was quenched with 220 µL of aqueous 0.1 M EDTA (pH 8.5), transferred to an 0.5 mL Amicon® Ultra 3 kDa MWCO spin filter, and centrifuged at 14,000 rcf for 20 min. The concentrated sample (~50 µL) was diluted to a volume of 500 µL with Milli-Q water, and analyzed by ESI-MS.

Sequence:
MQIFVKTLTG KTITLEVEPS DTIENVKAKI QDKEGIPPDQ QRLIFAGKQL EDGRTLSDYN IQKE(**NPE-ppppS**)TLHLV LRLRGG

Calculated average molecular weight for **NPE-p_4S65-Ub**: 9033.7 Da
Deconvoluted mass found: 9034 Da

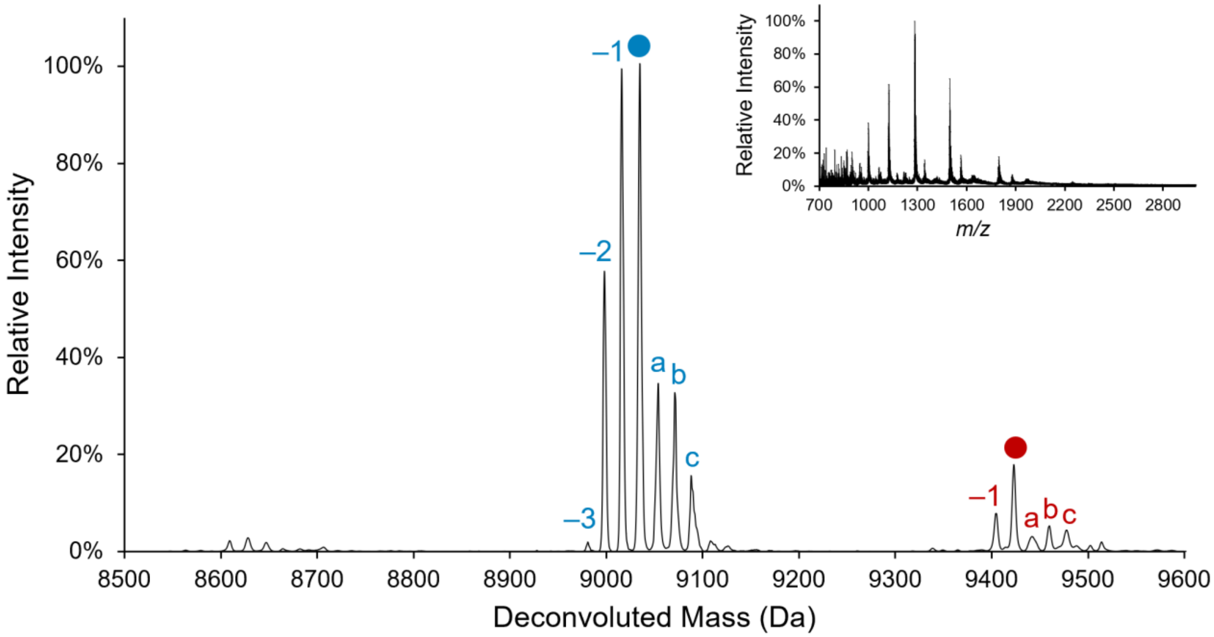

Figure S218: Deconvoluted mass spectrum of **NPE-p_4S65-Ub**.

Supporting Table 10: Deconvoluted mass values of labeled peaks for **NPE-p_4S65-Ub**.

Peak Label	Calculated m/z (Da)	Found m/z (Da)	Assignment
–3	8980	8981	–3 H_2O
–2	8998	8998	–2 H_2O
–1	9016	9016	–1 H_2O
●	9034	9034	**NPE-p_4S65-Ub**
a	9056	9054	Na^+ adduct
b	9072	9072	K^+ adduct
c	9087	9088	Fe^{3+} adduct

Peak Label	Calculated m/z (Da)	Found m/z (Da)	Assignment
–1	9405	9405	–1 H_2O
●	9423	9423	**(NPE-p_4S65-Ub)-(p_3-NPE)**
a	9445	9443	Na^+ adduct
b	9461	9461	K^+ adduct
c	9476	9478	Fe^{3+} adduct

11.4 p4S65-Ub

The sample vial containing **NPE-p4S65-Ub** was placed on ice and irradiated with a consumer grade UV LED lamp (Everbeam UV-LED Black Light [λ = 365 nm, 50 W, AC 240 V]). for 1 hour. The colorless solution gradually turned yellow-brownish, indicating successful photodeprotection of the NPE group.

Sequence:
MQIFVKTLTG KTITLEVEPS DTIENVKAKI QDKEGIPPDQ QRLIFAGKQL EDGRTLSDYN IQKE(**ppppS**)TLHLV LRLRGG

Calculated average molecular weight for **p4S65-Ub**: 8884.7
Deconvoluted mass found: 8885 Da

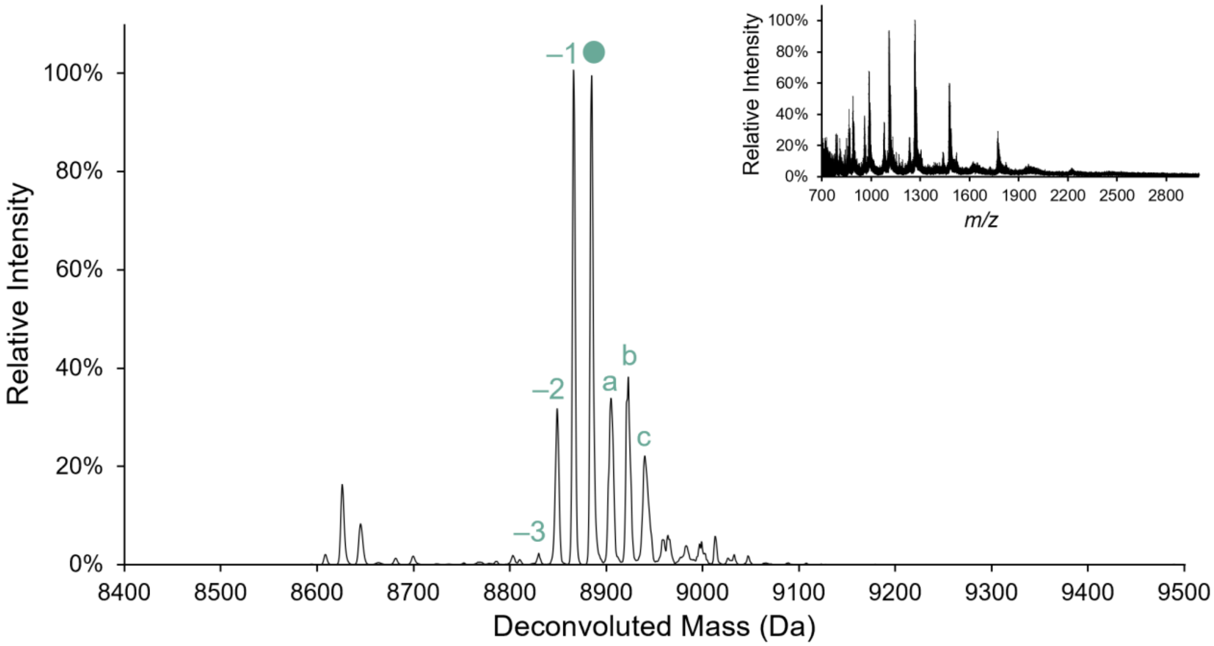

Figure S219: Deconvoluted mass spectrum of **p4S65-Ub**.

Supporting Table 11: Deconvoluted mass values of labeled peaks for **p4S65-Ub**.

Peak Label	Calculated m/z (Da)	Found m/z (Da)	Assignment
–3	8831	8832	–3 H_2O
–2	8849	8849	–2 H_2O
–1	8867	8866	–1 H_2O
●	8885	8885	**p4S65-Ub**
a	8907	8905	Na^+ adduct
b	8923	8923	K^+ adduct
c	8938	8940	Fe^{3+} adduct

11.5 wt-Ub + Reagent 3b

A 1.5 mL Eppendorf® tube was charged with solid reagent **3b** (7.2 mg, 13.8 µmol), and to it was added 200 µL of a 340 mM $ZnCl_2$ solution in DMA. This suspension was sonicated briefly. A 20 µL aliquot of a 2.0 µg/µL aqueous stock solution of **wt-Ub** (40 µg, 4.7 nmol) was added to the Eppendorf® tube, resulting in a 9:1 DMA/H_2O mixture. The contents in the tube were thoroughly mixed *via* vortexing, and the solution was incubated at 45 °C in a water bath for 1.5 h, interrupted by occasional mixing by inversion. After the allotted time, the reaction mixture was quenched with 220 µL of aqueous 0.1 M EDTA (pH 8.5), transferred to an 0.5 mL Amicon® Ultra 3 kDa MWCO centrifuge filter, and centrifuged at 14,000 rcf for 20 min. The concentrated sample (~50 µL) was diluted to a volume of 500 µL with Milli-Q water, and analyzed by ESI-MS.

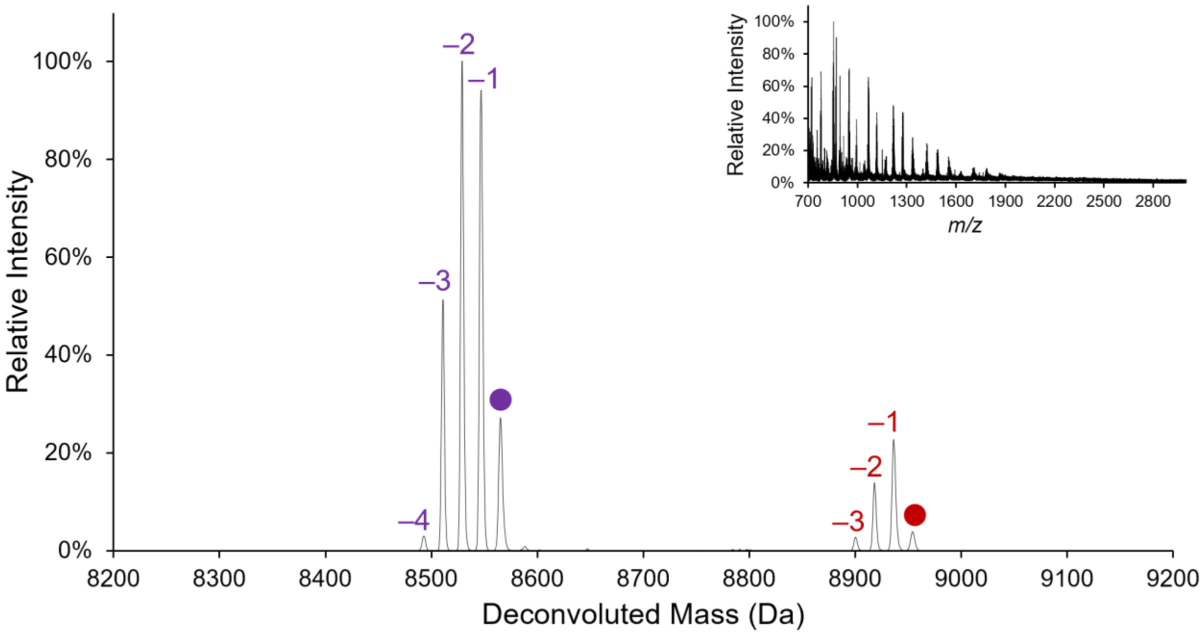

Figure S220: Deconvoluted mass spectrum of products resulting from **wt-Ub** and **3b**.

Supporting Table 12: Deconvoluted mass values of labeled peaks for **wt-Ub** control experiment.

Peak Label	Calculated m/z (Da)	Found m/z (Da)	Assignment
–4	8493	8494	–4 H_2O
–3	8511	8511	–3 H_2O
–2	8529	8529	–2 H_2O
–1	8547	8547	–1 H_2O
●	8565	8565	**wt-Ub**

Peak Label	Calculated m/z (Da)	Found m/z (Da)	Assignment
–3	8900	8902	–3 H_2O
–2	8918	8918	–2 H_2O
–1	8936	8937	–1 H_2O
●	8954	8956	**Ub-(p_3-NPE)**

To confirm that the observed dehydration signals (consecutive losses of 18 Da) were not simply artifacts arising from the deconvolution algorithm, matrix-assisted laser desorption/ionization-time of flight mass spectrometry (MALDI-ToF) was also employed to analyze the reaction products between **wt-Ub** and **3b**. Following the reaction conditions described previously for a duration of 1.5 h at 45 °C, the sample was concentrated in a 0.5 mL 3 kDa MWCO centrifuge filter and diluted to a final volume of 500 µL with Milli-Q water. An aliquot (1 µL) of this reaction mixture was spotted on a Bruker MTP 384 ground steel target plate, followed by 1 µL of internal standard solution (0.1 mg/mL of Ribonuclease A in Milli-Q water) and 1 µL of matrix solution (saturated solution of sinapinic acid in a 1:1 mixture of 0.1% TFA in water and 0.1% TFA in acetonitrile). The spots were *not* mixed and were allowed to dry completely before loading into the spectrometer. MALDI-ToF spectra were collected on a high-resolution Bruker Autoflex LRF Speed mass spectrometer in positive reflector mode in a 5–20 kDa mass range. The data was processed in Bruker FlexAnalysis software, and the spectrum was referenced to the internal standard (Ribonuclease A at 13682 Da). Indeed, the signals corresponding to dehydration were also observed by MALDI-ToF, indicating that such peaks are not artifacts from the deconvolution of the ESI-MS data.

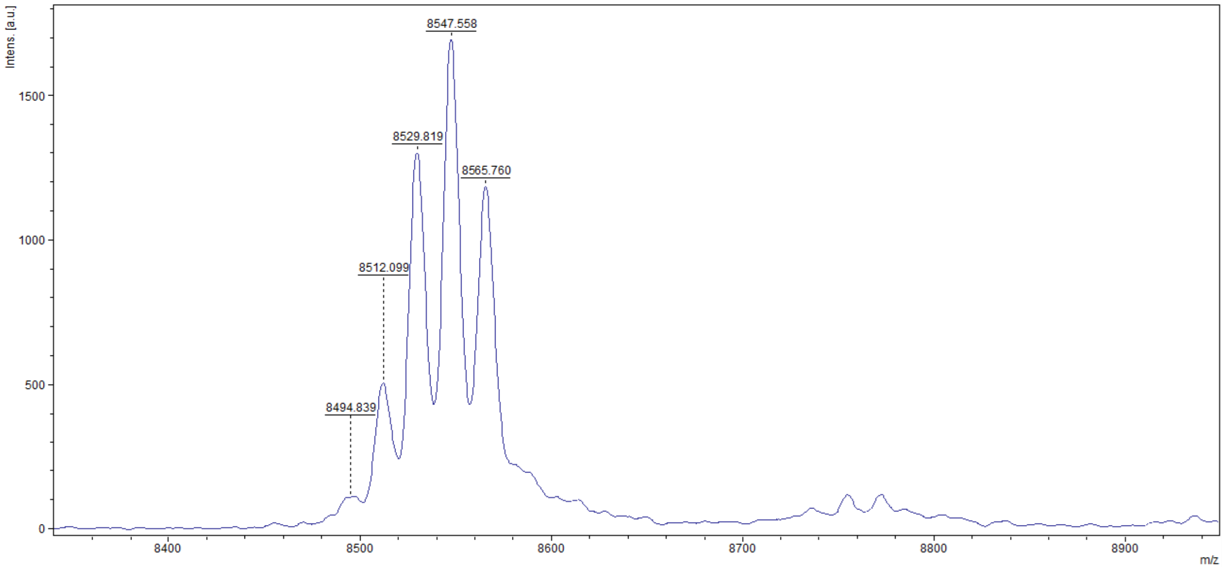

Figure S221: MALDI-ToF spectrum of dehydration products resulting from **wt-Ub** and **3b**.

12 Bibliography

[1] A. B. Pangborn, M. A. Giardello, R. H. Grubbs, R. K. Rosen, F. J. Timmers, *Organometallics* **1996**, *15*, 1518–1520.

[2] T. Schneider, K. Schwedtmann, J. Fidelius, J. J. Weigand, *Nat. Synth.* **2023**, *2*, 972–979.

[3] M. Meisel, H. Bock, B. Solouki, M. Kremer, *Angew. Chem. Int. Ed.* **1989**, *28*, 1373–1376.

[4] A. M. Marmelstein, J. A. M. Morgan, M. Penkert, D. T. Rogerson, J. W. Chin, E. Krause, D. Fiedler, *Chem. Sci.* **2018**, *9*, 5929–5936.

[5] S. M. Shepard, C. C. Cummins, *J. Am. Chem. Soc.* **2019**, *141*, 1852–1856.

[6] J. Singh, A. Ripp, T. M. Haas, D. Qiu, M. Keller, P. A. Wender, J. S. Siegel, K. K. Baldridge, H. J. Jessen, *J. Am. Chem. Soc.* **2019**, *141*, 15013–15017.

[7] J. Singh, N. Steck, D. De, A. Hofer, A. Ripp, I. Captain, M. Keller, P. A. Wender, R. Bhandari, H. J. Jessen, *Angew. Chem. Int. Ed.* **2019**, *58*, 3928–3933.

[8] K. Qian, S. M. Shepard, T. Xin, G. Park, C. C. Cummins, *J. Am. Chem. Soc.* **2023**, *145*, 6045–6050.

[9] S. M. Shepard, H. Kim, Q. X. Bang, N. Alhokbany, C. C. Cummins, *J. Am. Chem. Soc.* **2021**, *143*, 463–470.

[10] G. Park, E. C. Wralstad, N. Faginas-Lago, K. Qian, R. T. Raines, G. Bistoni, C. C. Cummins, *ACS Cent. Sci.* **2024**, *10*, 1415–1422.

[11] T. L. Parker, *Novel Bis(Phosphoranylidene) Ammonium Salts*, **1991**, US5045632A.

[12] V. Balasanthiran, M. H. Chisholm, K. Choojun, C. B. Durr, P. M. Wambua, *J. Organomet. Chem* **2016**, *812*, 56–65.

[13] D. Grunhaus, E. R. Molina, R. Cohen, T. Stein, A. Friedler, M. Hurevich, *Org. Process. Res. Dev.* **2022**, *26*, 2492–2497.

[14] A. M. Marmelstein, L. M. Yates, J. H. Conway, D. Fiedler, *J. Am. Chem. Soc.* **2014**, *136*, 108–111.
